# A Suitable Functionalization of Nitroindazoles with Triazolyl and Pyrazolyl Moieties via Cycloaddition Reactions

**DOI:** 10.3390/molecules25010126

**Published:** 2019-12-28

**Authors:** Mohammed Eddahmi, Nuno M. M. Moura, Latifa Bouissane, Ouafa Amiri, M. Amparo F. Faustino, José A. S. Cavaleiro, Ricardo F. Mendes, Filipe A. A. Paz, Maria G. P. M. S. Neves, El Mostapha Rakib

**Affiliations:** 1 Laboratory of Organic and Analytic Chemistry, Faculty of Sciences and Technics, Sultan Moulay Slimane University, BP 523, 2300 Beni-Mellal, Morocco; eddahmi.med@gmail.com (M.E.); latifa_bouissane@yahoo.fr (L.B.); amiri.ouafa@gmail.com (O.A.); 2 QOPNA & LAQV-REQUIMTE, Chemistry Department, University of Aveiro, 3810-193 Aveiro, Portugal; faustino@ua.pt (M.A.F.F.); jcavaleiro@ua.pt (J.A.S.C.); 3 CICECO - Aveiro Institute of Materials, Chemistry Department, University of Aveiro, 3810-193 Aveiro, Portugal; rfmendes@ua.pt (R.F.M.); filipe.paz@ua.pt (F.A.A.P.)

**Keywords:** nitrogen heterocycles, nitroindazole, alkylation, click chemistry, 1,3-dipolar cycloaddition, nitrile imines

## Abstract

The alkylation of a series of nitroindazole derivatives with 1,2-dibromoethane afforded the corresponding *N*-(2-bromoethyl)- and *N*-vinyl-nitro-1*H*-indazoles. The Cu(I)-catalysed azide- alkyne 1,3-dipolar cycloaddition was selected to substitute the nitroindazole core with 1,4-disubstituted triazole units after converting one of the *N*-(2-bromoethyl)nitroindazoles into the corresponding azide. The reactivity in 1,3-dipolar cycloaddition reactions with nitrile imines generated in situ from ethyl hydrazono-α-bromoglyoxylates was studied with nitroindazoles bearing a vinyl unit. The corresponding nitroindazole-pyrazoline derivatives were obtained in good to excellent yields.

## 1. Introduction

1,3-Dipolar cycloadditions have been successfully exploited as a facile route for the construction of five-membered heterocyclic rings [1,2]. These reactions are considered relevant alternatives to heterocyclic classic routes and allows one to install biologically significant functionalities like triazole and pyrazoline units in different scaffolds in a single step [3,4,5,6,7,8,9]. An attractive approach giving rise to substituted triazole derivatives is based on the 1,3-dipolar cycloaddition of azides to terminal alkynes catalysed by copper(I), the so-called Copper(I)-catalysed azide-alkyne (CuAAC) reaction [10,11,12]. In this click chemistry approach the presence of copper(I) is a crucial requirement to afford selectively only one of the regioisomers - the 1,4-disubstituted 1,2,3-triazole [11,13,14].

On the other hand, different cycloaddition procedures have been established to afford pyrazoline/pyrazole derivatives [4,15,16]. Among them, it is the one based on the reaction of *N*-aryl-*C*-ethoxycarbonylnitrile imines, generated in situ by base-induced dehydrobromination of ethyl hydrazono-α-bromoglyoxylates, with templates bearing vinyl units [17,18,19,20].

Pyrazoline and pyrazole derivatives have demonstrated a broad spectrum of interesting biological properties, and some of them were shown to have analgesic, anti-hyperglycemic, hypotensive, antipyretic, antioxidant, antiparasitic, antimicrobial, antitumoral and anti-inflammatory activities, among others [5,6,7,21,22,23]. 

Indazoles are another important group of *N*-heterocycles with significant biological activities as nitric oxide synthase (NOS) inhibitors, kinase inhibitors, anti-inflammatory, anticancer, antimicrobial, antifungal, antimalarial, and antileishmanial agents, among others. Some anticancer and anti-inflammatory drugs based in indazole scaffolds are commercially available [24,25,26,27,28,29,30]. Besides the biological properties presented by indazole derivatives, this family of *N*-heterocycles also showed potential to be used in other fields as corrosion inhibitors, components for OLEDs and battery applications, and as copolymerizing molecules for new materials [31,32,33,34]. 

Following our interest on developing synthetic approaches to functionalize the indazole core [35,36], we decided to follow the 1,3-dipolar cycloaddition methodology to substitute nitroindazoles with triazole or pyrazoline moieties, aiming in such way to obtain new compounds with improved biological features for different applications. In the strategy envisaged it was considered that the alkylation of indazole with 1,2-dibromoethane using liquid-liquid phase transfer catalysis, could afford not only the *N*-1- and *N*-*2*-bromoethylindazoles, but also the corresponding *N*-1- and *N*-2-vinylindazoles [37]. The annular tautomerisation of two nitrogen atoms (*N*-1, *N*-2) present in the indazole ring, has been explored in synthetic and theoretical studies concerning the substitution of *N-H*-indazole [38]. More recently, it was reported by our group that the reactivity of *N*-1 and *N*-2 alkylated indazole isomers is strongly dependent on the reaction conditions, namely, solvent proticity and pH, as well as, electronic and steric effects [39,40].

Herein it is described the synthesis of a series of *N*-bromoethyl-nitroindazoles and *N*-vinyl-nitroindazoles reacting the corresponding nitroindazoles with 1,2-dibromoethane. A detailed analysis of the reaction conditions allowed to develop a simple, fast and inexpensive protocol to obtain both the vinyl and the bromoethyl derivatives in just one step in reasonable amounts. One of the bromoethyl derivatives was used to afford the corresponding azide, and its reactivity in CuAAC reactions with different alkynes was studied. Additionally, the reactivity of one of the vinyl derivatives as dipolarophile was studied in the presence of *N*-aryl-*C*-ethoxycarbonylnitrile-imines.

## 2. Results

### 2.1. Reaction of Nitroindazoles ***1a**–**1d*** with 1,2-Dibromoethane

**Scheme 1:**
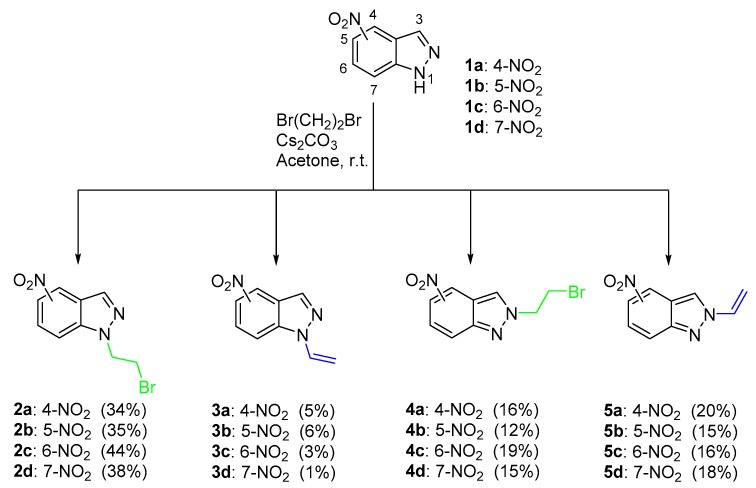
Synthetic access to *N*-bromoethyl-nitro-indazoles and *N*-vinyl-nitro-indazoles.

The reaction of the nitroindazoles **1a**–**1d** with 1,2-dibromoethane performed at room temperature, in acetone and in the presence of Cs_2_CO_3_ (1.1 equiv.) afforded the corresponding *N*-bromoethylnitroindazoles **2** and **4** and the *N*-vinyl-nitroindazoles **3** and **5** (Scheme 1) in overall yields ranging from 68% to 82%. The starting nitroindazoles **1** were obtained in excellent yields by diazotization of the adequate 2-methyl-nitroanilines according to the procedure described by Noelting (Scheme 1) [41].

The conditions indicated in Scheme 1 (cesium carbonate/acetone, room temperature) were selected considering that in the optimisation studies performed with derivative **1b** (see Table 1), the *N*-bromoethylnitroindazoles **2b** and **4b** and the elimination products **3b** and **5b** were obtained in just 1 h of reaction in reasonable amounts. From the results summarised in Table 1, it is patent that the distribution of the products **2b**–**5b** is strongly dependent on the experimental conditions used. 

The first experiments were performed with **1b** and using KOH (1 equiv.) in acetone at room temperature; such conditions gave rise, after 48 h, to the synthesis of compound **2b** and to **3b**, respectively, in 39% and 9% yields (entry 1). Two other products **4b** and **5b** were obtained; those are due to the reaction of the *N*-2 of the indazole moiety, the vinyl derivative **5b** being obtained in higher yield than **4b** (25% *versus* 12%).

In a previous study performed with 4-nitroindazole **1a** at low temperature (0 °C), only the substituted compounds **2a** and **4a** were obtained in a total yield of 58% [39]. Therefore, the elimination process with higher activation energy than the substitution route [42] is favoured by the higher temperature used in the reactions reported in the present work. It was also observed (entries 2 and 3) that the formation of the elimination products was strongly favoured when the reactions were performed in the presence of three equiv. of KOH either at room temperature (4 h of reaction) or at reflux (2 h of reaction). In fact, under these conditions, only traces of **4b** were detected, and the yield of **3b** increased to 25% and 21%, respectively. The change of the solvent to tetrahydrofuran (THF) or methanol had no positive effect on the yield/distribution of the products obtained. When methanol was used (entry 5) it was not observed the full consumption of the starting material and a significant amount was recovered (*ca.* 35%).

In the reactions performed in acetone and 1 equiv. of Cs_2_CO_3_ (entry 6), compound **2b** was isolated in 35% in just 1 h of reaction time and its elimination product **3b** in 6% yield; under those conditions, the vinyl derivative **5b** was isolated in slightly better yield (15%) than compound **4b** (12%). Interestingly, the preferential formation of the *N*-bromoethylnitroindazole **4b** towards its elimination product **5b** (20% vs. 8%) can be obtained in the reaction performed with K_2_CO_3_ at room temperature, although a longer reaction time was required (68 h) until the full consumption of the starting nitroindazole **1b** has been detected (entry 7). With this base, an improvement in the yield of **2b** to 47% was observed, and **3b** was isolated in 10% yield. 

The conditions of entry 6 were extended to the other nitroindazoles since these conditions showed the best relationship between the reaction time and total yield. In general, the product distribution and the moderate selectivity towards *N*-1 was maintained (Scheme 1). The *N*-bromoethylnitroindazoles **2a**–**2d** (34–44%) were always isolated as the major products and in much higher yields than the corresponding elimination derivatives **3a**–**3d** (1-6%), while the yield values of **4a**–**4d** (12-19%) were obtained in the same range of the vinyl derivatives **5a**–**5d** (15-20%). The elimination reaction leading to the *N*-vinyl substituted derivatives is strongly favoured in the *N*-2 alkylated derivatives, probably due to higher instability of the corresponding *N*-bromoethyl-nitroindazole [43,44]. 

The structures of all derivatives were unambiguously confirmed by using 1D (^1^H and ^13^C spectra) and 2D [(^1^H,^1^H) COSY, (^1^H,^13^C) HSQC and (^1^H,^13^C) HMBC] NMR techniques, and by mass spectrometry (see experimental section and supporting information SI, Figures S1–S67). 

The ^1^H-NMR spectra of derivatives **2a**–**2d** and **4a**–**4d** are consistent with *N*-alkylated indazole derivatives with a bromoethyl moiety showing, in the aliphatic region, two characteristic triplets at ca. δ 5.0 ppm and δ 4.0 ppm due to the resonances of the methylene protons. The ^13^C-NMR spectra of isomers **2a**–**2d** and **4a**–**4d** showed two signals at ca. δ 50 and δ 32 ppm due to the resonances of the two methylene carbons from the bromoethyl moiety. The assignments of these signals were confirmed by DEPT 135 studies.

The ^1^H-NMR spectra of *N*-substituted vinylic products **3a**–**3d** and **5a**–**5d** present in the aliphatic region two doublets of doublets due to the resonances of the methylene protons from the vinylic units at ca. δ 5.8 ppm and δ 5.1 ppm for derivatives **3a**–**3d**, while for compounds **5a**–**5d** these signals are slightly deshielded to ca. δ 6.2 ppm and δ 5.4 ppm. The resonance of the methinic proton from the vinylic group generates a doublet of doublets signal ranging from δ 7.9 to δ 7.3 ppm. These three NMR signals present characteristic constant couplings due to the *geminal* (*J*_gem_ ≈ 1 Hz), *cis* (*J*_cis_ ≈ 9 Hz) and *trans* (*J*_trans_ ≈ 15 Hz) correlations between the three protons from the vinyl unit. The resonances of all the remaining protons from the nitroindazole moiety generate signals in the aromatic region, as expected, being the proton from the position 3 of the pyrazolic ring (δ 9.2-8.2 ppm) the most deshielded one.

Additionally, the ^13^C-NMR spectra of the compounds **3a**–**3d** and **5a**–**5d** present the signals due to the resonance of the methinic carbons from the alkene groups at ca. δ 130 ppm. The signals corresponding to the resonances of the secondary carbons from the vinylic double bonds were easily identified by DEPT 135 NMR spectra ranging from δ 107.4 to δ 100.5 ppm.

The structures of all compounds isolated from the alkylation/elimination reactions of nitroindazoles **1a**–**1d** with 1,2-dibromoethane were confirmed by ESI(+) mass spectrometry, showing the peaks corresponding to the expected [M]^+^^•^ or [M + H]^+^ molecular ions.

### 2.2. CuAAC of Azidoethyl-Nitroindazoles with Terminal Alkynes

The CuAAC of organic azides and terminal alkynes is considered to be a versatile and promising approach to prepare new bioactive compounds, pharmaceutical lead compounds, bio-probes, soft materials among others [45,46,47,48,49,50].

**Scheme 2:**
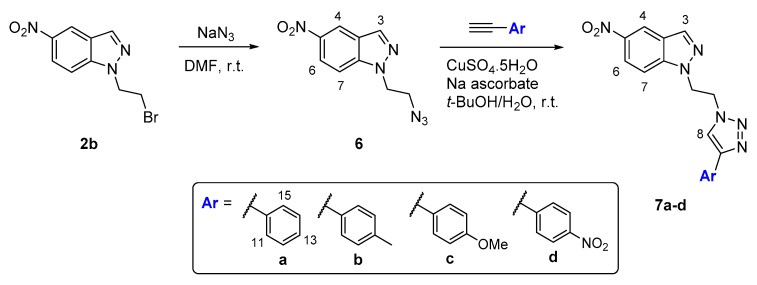
Synthetic access to new *N*-substituted triazolonitroindazoles by the copper(I)-catalysed azide-alkyne cycloaddition approach.

For the incorporation of triazole units in the nitroindazole core (Scheme 2) it was selected the *N*-bromoethylnitroindazole **2b** that was efficiently converted into the required 5-nitroindazole azide **6** (88%) by reaction with an excess of sodium azide in DMF. The structure of this synthon was confirmed by mass spectrometry and ^1^H-NMR and ^13^C data (see experimental data and Figures S68–S71 in SI).

The copper(I)-catalysed 1,3-dipolar cycloaddition reactions involving the azide **6** were performed in the presence of the terminal alkynes, 1-ethynylbenzene, 1-ethynyl-4-methylbenzene, 1-ethynyl-4-metoxybenzene and 1-ethynyl-4-nitrobenzene (Scheme 2). All these reactions were accomplished at room temperature in a mixture of *tert*-butyl alcohol-water (1:1) using sodium ascorbate as the reducing agent and CuSO_4_ as the copper source. After reaction times ranging from 12 to 16 h, the desired adducts **7a**–**7d** were isolated in excellent yields (71–87%) (Table 2). The yield of the CuAAC reaction and the consequent formation of the 1,4-disubstituted triazole ring is favoured by the presence of electron donor groups instead of electron-withdrawing groups in the *para* position of the phenyl ring from the alkyne reagent.

**Scheme 3:**
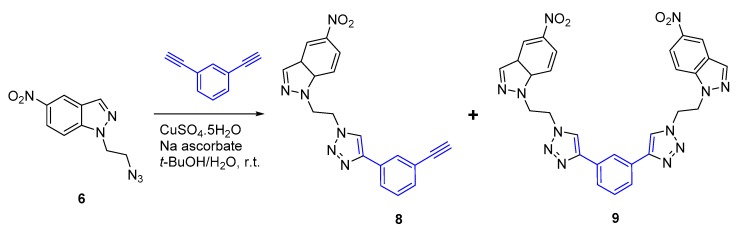
CuAAC reaction of derivative **6** with 1,3-diethynylbenzene.

The excellent performance of **6** in this CuAAC reactions prompted us to extend our study to 1,3-diethynylbenzene (Scheme 3). With this terminal alkyne, the reaction leads to the formation of 1,4-disubstituted triazole **8** and the bis-nitroindazolyl-triazole **9** in 23% and 58% yields, respectively. These two adducts were easily separated by column chromatography using hexane-ethyl acetate as solvent (Scheme 3).

The structure of all the newly synthesised compounds were unambiguously confirmed by using 1D (^1^H and ^13^C spectra) and 2D [(^1^H,^1^H) COSY, (^1^H,^13^C) HSQC and (^1^H,^13^C) HMBC] NMR techniques, and by mass spectrometry (see experimental section and supporting information SI, Figures S72–S97).

In this series of compounds, the success of the reaction was easily confirmed from the ^1^H-NMR spectra analysis. It shows the resonance of the protons from the ethylenic bridge as two multiplets at δ 5.10–5.01 ppm and δ 5.00–4.92 ppm and a remarkable singlet in the aromatic region due to the resonance of the proton from the triazole ring. In the ^1^H-NMR spectrum of compound **8**, an additional peak at δ 4.25 ppm due to the resonance of the proton from the alkyne unit was observed. The resonance of the two carbons from the triple bond generates two characteristic peaks at δ 83.1 and δ 81.2 ppm in the ^13^C-NMR spectrum.

The structure of each the indazole-triazole derivatives **7b** and **9** were unambiguously established by single-crystal X-ray diffraction studies (*vide infra*).

### 2.3. 1,3-Dipolar Cycloaddition Reactions of N-Vinyl-Nitroindazoles with Nitrile Imines

**Scheme 4:**
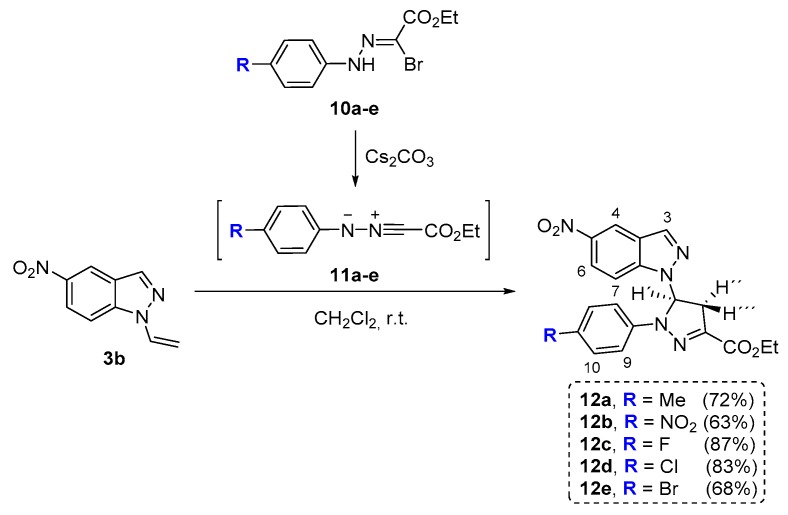
Reaction of *N*-vinyl-nitroindazole **3b** with nitrile imines **11a-e**.

Considering the easy accessibility to *N*-vinylnitroindazoles we envisaged an extra functionalization of the indazole nucleus with pyrazoline units by using the vinyl moiety to trap nitrile imines generated from the ethyl hydrazono-α-bromoglyoxylates (Scheme 4).

The ethyl hydrazono-α-bromoglyoxylates **10a**–**10e** selected to generate in situ the corresponding *N*-aryl-*C*-ethoxycarbonylnitrile imines **11a**–**11e** were obtained using the approach developed by Hamilton and co-workers. The preparation of the nitrile imine precursors was carried out by the reaction of ethyl acetoacetate with the adequate diazonium salts followed by bromination of the resulting azoacetoacetic esters [51].

The cycloaddition reactions involving the indazole **3b** and the *N*-aryl-*C*-ethoxycarbonylnitrile imines **11a**–**11e** were performed in dichloromethane at room temperature, in the presence of Cs_2_CO_3_ (2 equiv.). After 24 h of reaction, it was observed by TLC the total, or almost total, consumption of the starting indazole and this being accompanied by the formation of the main product. After the workup and purification of the reaction mixture by column chromatography, we were able to conclude by a detailed spectroscopic analysis that the major products were the pyrazoline cycloadducts **12a**–**12e** which were isolated in yields ranging from 63% to 83%. It is worth to refer that in neither case was isolated the corresponding pyrazole derivative from the dehydrogenation of the pyrazoline ring from derivatives **12a**–**12e**.

**Figure 1:**
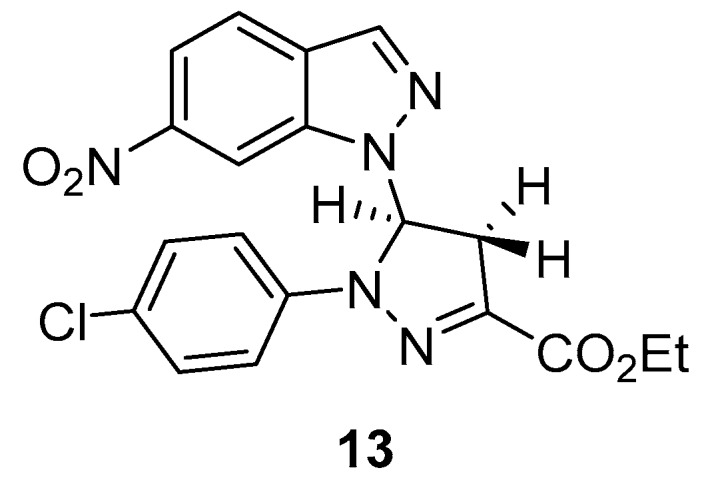
Structure of the pyrazoline-indazole cycloadduct **13** obtained from **3c**.

When the reaction was performed with the vinyl-nitroindazole **3c** and the nitrile imine **11d**, obtained in situ from the corresponding ethyl hydrazono-α-bromoglyoxylates **10d**, the expected pyrazoline derivative **13** (Figure 1) was obtained in 81% yield. This yield is similar to the one obtained with the indazole **3b**, showing that the position of the electron-withdrawing nitro group in the indazole moiety does not have a significant influence in the reaction yield. 

The structural assignments of the new cycloadducts **12a**–**12e** and **13** relied on 1D (^1^H and^13^C spectra) and 2D [(^1^H,^1^H) COSY, NOESY, (^1^H,^13^C) HSQC and (^1^H,^13^C) HMBC] NMR spectra and on their mass spectra (see experimental section and Figures S98–S119 in the SI).

The resonances due to the protons from the indazole core were not significantly affected by the presence of the introduced pyrazoline unit. Important diagnostic peaks confirming the presence of the pyrazoline unit are the two double doublets at ca. δ 7.58–7.76 ppm and δ 3.82–3.85 ppm and a multiplet at ca. δ 3.33–3.38 ppm, corresponding respectively to the pyrazoline protons H’, H′′ and H′′′. In the aliphatic region, there is also the expected quartet (ca. δ 4.3 ppm) and triplet (ca. δ 1.3 ppm) due to the resonance of the protons from the ethyl ester group. The ^13^C-NMR spectra show a distinctive signal near δ 161 ppm corresponding to the resonance of the carbonyl carbon from the ethyl ester group. The structure of the cycloadduct **13** was also unequivocally established by single-crystal X-ray diffraction studies (*vide infra*).

### 2.4. X-Ray Diffraction

**Figure 2:**
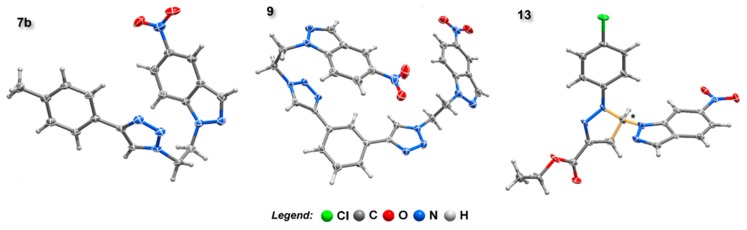
Schematic representation of molecular units presents in the crystal structures of compounds **7b**, **9** and **13**. Non-hydrogen atoms are represented as thermal ellipsoids drawn at the 50% probability level and hydrogen atoms as small spheres with arbitrary radii. The chiral carbon present in **13** is denoted by an asterisk.

Single-crystal X-ray diffraction analysis was used to study the structural features of three of the reported compounds in this paper, namely compounds **7b**, **9** and **13**. The *N*-substituted triazolo-nitroindazole **7b** crystallises in the centrosymmetric monoclinic space group *P*2_1_/n with the asymmetric unit being composed of a whole molecular unit as depicted in Figure 2. The small number of hydrogen bonding donors and acceptors in this compound leads to a close packing in the solid-state achieved, mainly, by weak hydrogen bonding interactions of the C‒H···N and C‒H···O types, having typical geometrical parameters: *d*_C···N_ distances found in the 3.195(3)-3.325(3) Å range with <(CHN) interaction angles ranging from 114 to139°; the *d*_C···O_ distances were found instead in the 3.277(3)–3.411(4) Å range with the corresponding <(CHO) interaction angles in the range 123–142°. Despite the presence of several aromatic moieties, only weak π···π contacts are present, most of them between aromatic rings composing the molecular unit itself [intercentroid distances of *d*_π···π_ = 3.9112(14)–3.9511(14) Å]. 

From the reaction of derivative **6** with 1,3-diethynylbenzene, we were able to crystallise the *bis*-nitroindazolyl-triazole **9**. Despite its intrinsic molecular symmetry, **9** crystallises in the triclinic *P*-1 space group, with the asymmetric unit being composed of a complete molecular unit (Figure 2). As for **7b**, the close packing in the solid-state is achieved mainly by weak C‒H···N and C‒H···O hydrogen bonding interactions [*d*_C···N_ distances found in the 3.237(3)–3.530(3) Å range with <(CHN) interaction angles ranging from 123 to158°; the *d*_C···O_ distances were found instead in the 3.291(4)–3.594(3) Å range with the corresponding <(CHO) interaction angles in the range 132-168°]. Interestingly, despite the presence of a high number of aromatic moieties, only a few highly offset π···π contacts are observed, all between aromatic rings within the molecular unit [intercentroid distances of *d*_π···π_ = 3.5320(14)–3.7007(15) Å]. 

The pyrazoline-indazole cycloadduct **13** crystallises in the noncentrosymmetric monoclinic space group *P*2_1_. The asymmetric unit is composed of a whole molecular unit containing one asymmetric carbon (C14, denoted with an asterisk in Figure 2). The crystal quality and the absence of heavy atomic elements did not allow a precise calculation of the Flack parameter. However, we believe that the crystal should consist of a racemic mixture since there is no reason for the performed reaction to exhibit any enantiomeric excess. As for the previous compounds, the close packing of **13** is achieved by weak C‒H···N and C‒H···O hydrogen-bonding interactions [*d*_C···N_ distances found in the 3.160(6)–3.329(6) Å range with <(CHN) interaction angles ranging from 115 to164°; *d*_C···O_ distance of 3.288(6) Å with the corresponding <(CHO) interaction angle of 128°]. No π···π contacts are observed. 

## 3. Materials and Methods

### 3.1. General Remarks

Melting points were measured using a B-540 melting point apparatus (Buchi, Flawil, Switzerland). Electrospray ionization mass spectra (ESI) were acquired with a Micromass Q-Tof 2 (Micromass, Manchester, UK), operating in the positive ion mode, equipped with a Z-spray source, an electrospray probe and a syringe pump. Source and desolvation temperatures were 80 °C and 150 °C, respectively. Capillary voltage was 3000 V. The spectra were acquired at a nominal resolution of 9000 and at cone voltages of 30 V. Nebulisation and collision gases were N_2_ and Ar, respectively. Compound solutions in methanol were introduced at a 10 μL min^−1^ flow rate. ^1^H and ^13^C solution NMR spectra were recorded on an Avance 300 spectrometer at 300.13 and 75.47 MHz, respectively (Bruker, Wissembourg, France). DMSO-*d*_6_ was used as solvent and tetramethylsilane (TMS) as the internal reference; the chemical shifts are expressed in δ (ppm) and the coupling constants (*J*) in Hertz (Hz). Unequivocal ^1^H assignments were made using 2D COSY (^1^H/^1^H), while ^13^C assignments were made on the basis of 2D HSQC (^1^H/^13^C) and HMBC (delay for long-range *J* C/H couplings were optimized for 7 Hz) experiments. Elemental analyses were performed on a CHNS-932 apparatus (LECO, Madrid, Spain). Column chromatography was carried out using silica gel (Merck, 35–70 mesh). Analytical TLC was carried out on 0.2 mm thick sheets precoated with silica gel 60 (Merck, city, Darmstadt, Germany). All chemicals were used as supplied. Solvents were purified or dried according to the literature procedures [52].

### 3.2. N-alkylation of Nitroindazole Derivatives 1a-d with 1,2-Dibromoethane. General Procedure 

To a solution of the appropriate nitroindazole **1a**–**1d** (100 mg, 0.62 mmol) in acetone (10.0 mL) it was added a small excess of cesium carbonate (1.1 equiv., 0.67 mmol, 218 mg). Then, the 1,2-dibromoethane alkylating agent (1.1 equiv., 0.67 mmol, 58 μL) was added dropwise and the resulting reactional mixture was maintained under stirring at room temperature until the TLC control showed the total consumption of the starting material (1 h). Then, the solvent was evaporated and the crude product was purified by column chromatography (silica gel) using hexane:toluene (1:1) as the eluent.

*1-(2-Bromoethyl)-4-nitro-1H-indazole*, **2a**. Yield: 34% (56.3 mg), orange solid, R*_f_*: 0.17, m.p.: 92–94 °C. ^1^H-NMR (DMSO-*d*_6_): δ 8.60 (1H, s, H-3), 8.35 (1H, d, *J* = 8.0 Hz, 1H-5), 8.19 (1H, d, *J* = 8.0 Hz, H-7), 7.68 (1H, t, *J* = 8.0 Hz, H-6), 5.00 (2H, t, *J* = 5.8 Hz, *N*-CH_2_), 4.00 (2H, t, *J* = 5.8 Hz, Br-CH_2_). ^13^C-NMR (DMSO-*d*_6_): δ 141.6 (C7a), 139.7 (C4), 132.4 (C3), 125.9 (C6), 118.6 (C5), 118.2 (C7), 115.8 (C3a), 50.0 (*N*-CH_2_), 32.0 (Br-CH_2_) ppm. MS-ESI(+): *m/z* 270.1 [M]^+^^•^. Elemental analysis calcd (%) for C_9_H_8_BrN_3_O_2_.1/20 PhCH_3_ C 40.98, H 3.09, N 15.27; found C 40.93, H 3.15, N 15.64.

*1-(2-Bromoethyl)-5-nitro-1H-indazole*, **2b**. Yield: 35% (57.9 mg), yellow solid, R*_f_*: 0.18, m.p.: 109–111 °C. ^1^H-NMR (DMSO-*d*_6_): δ 8.84 (1H, d, *J* = 2.2 Hz, H-4), 8.48 (1H, s, H-3), 8.25 (H-6, dd, *J* = 9.3 and 2.2 Hz, 1H), 7.97 (1H, d, *J* = 9.3 Hz, H-7), 4.95 (2H, t, *J* = 5.8 Hz, *N*-CH_2_), 3.99 (2H, t, *J* = 5.8 Hz, Br-CH_2_) ppm. ^13^C-NMR (DMSO-*d*_6_): δ 141.9 (C7a), 141.8 (C5), 136.9 (C3), 122.6 (C3a), 121.1 (C6), 119.1 (C4), 110.9 (C7), 49.9 (*N*-CH_2_), 32.1 (Br-CH_2_) ppm. MS-ESI(+): *m/z* (^81^Br) 272.0 [M + H]^+^. Elemental analysis calcd (%) for C_9_H_8_BrN_3_O_2_.1/20 PhCH_3_ C 40.98, H 3.09, N 15.27; found C 40.61, H 3.17, N 15.64.

*1-(2-Bromoethyl)-6-nitro-1H-indazole*, **2c**. Yield: 44% (72.8 mg), orange solid, R*_f_*: 0.20, m.p.: 117–119 °C. ^1^H-NMR (CDCl_3_): δ 8.46 (1H, t, *J* = 1.0 Hz, H-7), 8.19 (1H, d, *J* = 1.0 Hz, H-3), 8.05 (1H, dd, *J* = 8.9 and 1.1 Hz, H-5), 7.86 (1H, dd, *J* = 8.9 and 1.1 Hz, H-4), 4.85 (*N*-CH_2_, t, *J* = 6.3 Hz, 2H), 3.87 (Br-CH_2_, t, *J* = 6.3 Hz, 2H) ppm. ^13^C-NMR (CDCl_3_); δ 146.8 (C7a), 139.0 (C6), 134.6 (C3), 127.0 (C3a), 122.1 (C4), 115.8 (C5), 105.9 (C7), 50.6 (N-CH_2_), 29.6 (Br-CH_2_) ppm. MS-ESI(+): *m/z* (^81^Br) 272.0 [M + H]^+^. Elemental analysis calcd (%) for C_9_H_8_BrN_3_O_2_.1/40 PhCH_3_ C 40.48, H 3.04, N 15.42; found C 40.70, H 3.05, N 15.85.

*1-(2-Bromoethyl)-7-nitro-1H-indazole*, **2d**.Yield: 38% (62.9), yellow solid, R*_f_*: 0.37, m.p.: 77–79 °C. ^1^H-NMR (CDCl_3_): δ 8.25 (1H, s, H-3), 8.19 (1H, dd, *J* = 7.9 and 1.1 Hz, H-6), 8.06 (1H, dd, *J* = 7.9 and 1.1 Hz, H-4), 7.28 (1H, t, *J* = 7.9 Hz, H-5), 5.07 (2H, t, *J* = 6.6 Hz, *N*-CH_2_), 3.74 (2H, t, *J* = 6.6 Hz, Br-CH_2_) ppm. ^13^C-NMR (CDCl_3_): δ 135.6 (C3), 135.4 (C7a), 131.0 (C7), 129.0 (C3a), 128.4 (C4), 125.4 (C6), 120.3 (C5), 54.0 (N-CH_2_), 30.2 (Br-CH_2_) ppm. 

MS-ESI(+): *m/z* 270.0 [M]^+^^•^. Elemental analysis calcd (%) for C_9_H_8_BrN_3_O_2_ C 40.02, H 2.99, N 15.56; found C 40.24, H 3.06, N 15.48.

*4-Nitro-1-vinyl-1H-indazole*, **3a**. Yield: 5% (5.8 mg), orange solid, R*_f_*: 0.40, m.p.: 135–137 °C. ^1^H-NMR (DMSO-*d*_6_): δ 8.71 (1H, s, H-3), 8.46 (1H, d, *J* = 8.1 Hz, H-5), 8.23 (1H, d, *J* = 8.1 Hz, H-7), 7.87 (1H, dd, *J* = 15.2, 8.8 Hz, *N*-CH=), 7.74 (1H, t, *J* = 8.1 Hz, H-6), 5.80 (1H, d, *J* = 15.2 Hz, H′′), 5.08 (1H, d, *J* = 8.8 Hz, H′).^13^C-NMR (DMSO-*d*_6_): δ 139.93 (C7a),139.86 (C4), 134.4 (C3), 129.9 (*N*-CH=), 127.2 (C6), 119.4 (C5), 117.9 (C7), 116.7 (C3a), 100.5 (CH_2_) ppm. MS-ESI(+): *m/z* 190.1 [M + H]^+^. Elemental analysis calcd (%) for C_9_H_7_N_3_O_2_.1/10 H_2_O C 56.60, H 3.80, N 22.00; found C 56.30, H 3.79, N 22.29.

*5-Nitro-1-vinyl-1H-indazole*, **3b**. Yield: 6% (7.0 mg), yellow solid, R*_f_*: 0.42, m.p.: 160–162 °C. ^1^H-NMR (DMSO-*d*_6_): δ 8.85 (1H, d, *J* = 2.2 Hz, H-4), 8.59 (1H, s, H-3), 8.31 (1H, dd, *J* = 9.2 and 2.2 Hz, H-6), 8.10 (1H, d, *J* = 9.2 Hz, H-7), 7.81 (1H, dd, *J* = 15.2 Hz and 8.8 Hz, *N*-CH=), 5.77 (1H, d, *J* = 15.2 Hz, H′′), 5.05 (1H, d, *J* = 8.8 Hz, H′) ppm. ^13^C-NMR (DMSO-*d*_6_): δ 142.5 (C7a), 140.0 (C4), 138.7 (C3), 130.0 (*N*-CH=), 123.6 (C3a), 122.1 (C6), 119.3 (C4), 110.8 (C7), 100.6 (CH_2_) ppm. MS-ESI(+): *m/z* 190.1 [M + H]^+^. Elemental analysis calcd (%) for C_9_H_7_N_3_O_2_.1/11 H_2_O C 56.65, H 3.79, N 22.02; found C 57.06, H 4.18, N 21.63.

*6-Nitro-1-vinyl-1H-indazole*, **3c**. Yield: 3% (3.5 mg), yellow solid, R*_f_*: 0.33, m.p.: 127–129 °C. ^1^H-NMR (CDCl_3_): δ 8.53 (1H, d, *J* = 1.6 Hz, H-7), 8.25 (1H, s, H-3), 8.09 (1H, dd, *J* = 8.8 and 1.6 Hz, H-5), 7.88 (1H, d, *J* = 8.8 Hz, H-4), 7.40 (1H, dd, *J* = 15.4 and 8.9 Hz, *N*-CH=), 5.87 (1H, dd, *J* = 15.4 and 1.2 Hz, H′′), 5.10 (1H, dd, *J* = 8.9 and 1.2 Hz, H’) ppm. ^13^C-NMR (CDCl_3_): δ 147.1 (C7a), 137.3 (C6), 135.7 (C3), 129.3 (*N*-CH=), 127.9 (C3a), 122.1 (C4), 116.6 (C5), 106.1 (C7), 101.4 (CH_2_) ppm. MS-ESI(+): *m/z* 190.1 [M + H]^+^. Elemental analysis calcd (%) C_9_H_7_N_3_O_2_.1/5 H_2_O C 56.07, H 3.87, N 21.80; found C 55.70, H 3.49, N 22.14.

*7-Nitro-1-vinyl-1H-indazole*, **3d**. Yield: 1% (1.2 mg), orange solid, R*_f_*: 0.46, m.p.: 97–99 °C. ^1^H-NMR (CDCl_3_): δ 8.30 (1H, s, H-3),8.13 (1H, dd, *J* = 7.8 and 1.0 Hz, H-6), 8.05 (1H, dd, *J* = 7.8 and 1.0 Hz, H-4), 7.46 (1H, dd, *J* = 15.0 and 8.6 Hz, *N*-CH=), 7.30 (1H, t, *J* = 7.8 Hz, H-5), 5.80 (1H, dd, *J* = 15.0 and 0.6 Hz, H′′), 5.04 (1H, dd, *J* = 8.6 and 0.6 Hz, H’) ppm. ^13^C-NMR (CDCl_3_): δ 136.6 (C3), 132.1 (*N*-CH=), 129.4 (C7a), 129.1 (C7), 128.0 (C3a), 1278.0 (C4), 125.4 (C6), 120.9 (C5), 103.3 (CH_2_) ppm. MS-ESI(+): *m/z* 190.1 [M + H]^+^. Elemental analysis calcd (%) C_9_H_7_N_3_O_2_.1/11 H_2_O C 56.65, H 3.79, N 22.02; found C 56.96, H 4.11, N 22.23.

*2-(2-Bromoethyl)-4-nitro-2H-indazole*, **4a**. Yield: 16% (26.5 mg), orange solid, R*_f_*: 0.12, m.p.: 93–95 °C. ^1^H-NMR (DMSO-*d*_6_): δ 9.00 (1H, s, H-3), 8.22 (2H, d, *J* = 8.0 Hz, H-5 and H-7), 7.53 (1H, t, *J* = 8.0 Hz, H-6), 5.00 (2H, t, *J* = 5.9 Hz, *N*-CH_2_), 4.10 (2H, t, *J* = 5.9 Hz, Br-CH_2_) ppm. ^13^C-NMR (DMSO-*d*_6_): δ 149.3 (C7a),140.1 (C4), 126.3 (C5), 125.8 (C3), 124.9 (C6), 120.7 (C7), 113.7 (C3a), 54.5 (*N*-CH_2_), 31.8 (Br-CH_2_) ppm. MS-ESI(+): *m/z* 270.1 [M]^+^^•^. Elemental analysis calcd (%) for C_9_H_8_BrN_3_O_2_.1/15 PhCH_3_ C 41.16, H 3.11, N 15.21; found C 41.04, H 3.09, N 15.65. 

*2-(2-Bromoethyl)-5-nitro-2H-indazole*, **4b**. Yield: 12% (19.9 mg), yellow solid, R*_f_*: 0.07, m.p.: 136–138 °C. ^1^H-NMR (DMSO-*d*_6_): δ 8.94 (1H, dd, *J* = 2.2 and 0.9 Hz, H-4), 8.89 (1H, d, *J* = 0.9, H-3), 8.04 (1H, dd, *J* = 9.5 and 2.2 Hz, H-6), 7.81 (1H, dt, *J* = 9.5 and 0.9 Hz, H-7), 4.96 (2H, t, *J* = 5.8 Hz, *N*-CH_2_), 4.07 (2H, t, *J* = 5.8 Hz, Br-CH_2_) ppm. ^13^C-NMR (DMSO-*d*_6_): δ 149.3 (C7a), 142.1 (C5), 130.4 (C3), 120.7 (C6), 119.9 (C4), 119.6 (C3a), 118.2 (C7), 54.6 (*N*-CH_2_), 31.9 (Br-CH_2_) ppm. MS-ESI(+): *m/z* (^81^Br) 272.4 [M + H]^+^. Elemental analysis calcd (%) for C_9_H_8_BrN_3_O_2_.1/20 PhCH_3_ C 40.98, H 3.09, N 15.27; found C 40.94, H 3.19, N 15.67.

*2-(2-Bromoethyl)-6-nitro-2H-indazole*, **4c**. Yield: 19% (31.5 mg), orange solid, R*_f_*: 0.08, m.p.: 161-163 °C.^1^H-NMR (CDCl_3_): δ 8.73 (1H, d, *J* = 1.0 Hz, H-3), 8.66-8.65 (1H, m, H-7), 8.02 (1H, dd, *J* = 9.2 and 1.0 Hz, H-5), 7.83 (1H, dd, *J* = 9.2 and 2.0 Hz, H-4), 4.98 (2H, t, *J* = 5.8 Hz, *N*-CH_2_), 4.08 (2H, t, *J* = 5.8 Hz, Br-CH_2_) ppm. ^13^C-NMR (CDCl_3_): δ 146.2 (C7a), 146.1 (C6), 126.7 (C3), 123.8 (C3a), 122.9 (C4), 114.9 (C5), 114.8 (C7), 54.8 (*N*-CH_2_), 31.9 (Br-CH_2_) ppm. MS-ESI(+): *m/z* 270.1 [M]^+^^•^. Elemental analysis calcd (%) for C_9_H_8_BrN_3_O_2_.1/20 PhCH_3_ C 40.98, H 3.09, N 15.27; found C 40.61, H 3.25, N 15.43.

*2-(2-Bromoethyl)-7-nitro-2H-indazole*, **4d**. Yield: 15% (24.8 mg), orange solid, R*_f_*: 0.05, m.p.: 149–151 °C. ^1^H-NMR (DMSO-*d*_6_): δ 8.89 (1H, s, H-3), 8.47-8.18 (2H, m, H-6 and H-4), 7.29 (1H, t, *J* = 7.8 Hz, H-5), 4.99 (2H, t, *J* = 5.8 Hz, N-CH_2_), 4.07 (2H, t, *J* = 5.8 Hz, Br-CH_2_) ppm. ^13^C-NMR (DMSO-*d*_6_): δ 139.9 (C7a), 136.6 (C7), 130.4 (C3), 128.2 (C4), 125.2 (C6), 124.9 (C3a), 120.0 (C5), 54.6 (N-CH_2_), 32.0 (Br-CH_2_). MS-ESI(+): *m/z* 270.0 [M]^+^^•^. Elemental analysis calcd (%) for C_9_H_8_BrN_3_O_2_.1/20 PhCH_3_ C 40.98, H 3.09, N 15.27; found C 40.99, H 2.89, N 15.60.

*4-Nitro-2-vinyl-2H-indazole*, **5a**. Yield: 20% (23.2 mg), yellow solid, R*_f_*: 0.25, m.p.: 133–135 °C. ^1^H-NMR (DMSO-*d*_6_): δ 9.16 (1H, d, *J* = 1.0 Hz, H-3), 8.34-8.05 (2H, m, H-5 and H-7), 7.73 (1H, dd, *J* = 15.5 and 8.7 Hz, *N*-CH=), 7.56 (1H, dd, *J* = 8.6 Hz and 7.5 Hz, H-6), 6.22 (1H, dd, *J* = 15.5 Hz and 1.1 Hz, H′′), 5.38 (1H, dd, J = 8.7 and 1.1 Hz, H′) ppm. ^13^C-NMR (DMSO-*d*_6_): δ 149.6 (C7a), 140.3 (C4), 133.7 (C3), 126.4 (*N*-CH=), 126.0 (C6), 124.3 (C5), 121.5 (C7), 114.3 (C3a), 107.2 (CH_2_) ppm. MS-ESI(+): *m/z* 190.1 [M + H]^+^. Elemental analysis calcd (%) for C_9_H_7_N_3_O_2_.1/10 H_2_O C 56.60, H 3.80, N 22.00; found C 56.44, H 3.51, N 22.27.

*5-Nitro-2-vinyl-2H-indazole*, **5b**. Yield: 15% (17.4 mg), yellow solid, R*_f_*: 0.13, m.p.: 128–130 °C. ^1^H-NMR (DMSO-*d*_6_): δ 9.03 (1H, s, H-3), 8.91 (1H, d, *J* = 1.7 Hz, H-4), 8.05 (1H, dd, *J* = 9.5 and 1.7 Hz, H-6), 7.83 (1H, d, *J* = 9.5 Hz, H-7), 7.70 (1H, dd, *J* = 15.4 and 8.7 Hz, *N*-CH=), 6.16 (1H, dd, *J* = 15.4 and 1.1 Hz, H′′), 5.37 (1H, dd, *J* = 8.7 and 1.1 Hz, H′) ppm. ^13^C-NMR (DMSO-*d*_6_): δ 149.4 (C7a), 142.5 (C4), 133.7 (C3), 128.6 (*N*-CH=), 120.9 (C6), 120.9 (C4), 120.1 (C3a), 118.4 (C7), 107.2 (CH_2_) ppm. MS-ESI(+): *m/z* 190.1 [M + H]^+^. Elemental analysis calcd (%) for C_9_H_7_N_3_O_2_.1/11 H_2_O C 56.65, H 3.79, N 22.02; found C 56.23, H 3.78, N 22.41.

*6-Nitro-2-vinyl-2H-indazole*, **5c**. Yield: 16% (18.6 mg), yellow solid, R*_f_*: 0.17, m.p.: 109–111 °C. ^1^H-NMR (CDCl_3_): δ 8.72 (1H, d, *J* = 1.9 Hz, H-7), 8.22 (1H, d, *J* = 0.8 Hz, H-3), 7.91 (1H, dd, *J* = 9.2 and 1.9 Hz, H-5), 7.77 (1H, dd, *J* = 9.2 and 0.8 Hz, H-4), 7.36 (1H, dd, *J* = 15.6 and 8.7 Hz, *N*-CH=), 6.11 (1H, dd, *J* = 15.6 and 1.7 Hz, H′′), 5.33 (1H, dd, *J* = 8.7 and 1.7 Hz, H′) ppm. ^13^C-NMR (CDCl_3_): δ 147.5 (C7a), 147.3 (C6), 133.5 (*N*-CH=), 124.4 (C3a), 122.0 (C3), 121.8 (C4), 116.56(C5), 115.9 (C7), 107.3 (CH_2_) ppm. MS-ESI(+): *m/z* 190.1 [M + H]^+^. Elemental analysis calcd (%) C_9_H_7_N_3_O_2_.1/6 H_2_O C 56.25, H 3.85, N 21.87; found C 56.15, H 4.08, N 22.01.

*7-Nitro-2-vinyl-2H-indazole*, **5d**. Yield: 15% (17.4 mg), yellow solid, R*_f_*: 0.07, m.p.: 149–151 °C. ^1^H-NMR (CDCl_3_): δ 8.39 (1H, s, H-3), 8.37 (1H, dd, *J* = 7.9 and 1.0 Hz, H-6), 8.06 (1H, dd, *J* = 7.9 and 1.0 Hz, H-4), 7.46 (1H, dd, *J* = 15.7 and 8.8 Hz, *N*-CH=), 7.22 (1H, t, *J* = 7.9 Hz, H-5), 6.12 (1H, dd, *J* = 15.7 and 1.8 Hz, H′′), 5.35 (1H, dd, *J* = 8.8 and 1.8 Hz, H’) ppm. ^13^C-NMR (CDCl_3_): δ 141.3 (C7a), 137.9 (C7), 133.7 (C3), 128.9 (HC-N *N*-CH=), 126.2 (C4), 125.6 (C3a), 122.8 (C6), 121.0 (C5), 107.4 (CH_2_) ppm. MS-ESI(+): *m/z* 190.1 [M + H]^+^. Elemental analysis calcd (%).C_9_H_7_N_3_O_2_.2/5 H_2_O C 55.05, H 4.00, N 21.40; found C 54.97, H 3.76, N 21.33.

### 3.3. Procedure for the Preparation of 1-(2-Azidoethyl)-5-nitro-1H-indazole Intermediate ***6***

A mixture of 1-(2-bromoethyl)-5-nitro-1*H*-indazole (**2b**, 0.1 g, 0.37 mmol) and sodium azide NaN_3_ (10 equiv., 3.7 mmol, 130 μL) in 5 mL of DMF was maintained under stirring at room temperature for 24 h. After this period, the TLC control confirmed the disappearance of the starting material and the formation of a main product. Then, the reaction mixture was washed with water and the desired product was extracted with diethyl ether. The organic layer was separated, dried under Na_2_SO_4_ and the solvent evaporated under reduced pressure. The residue was crystallized in hexane affording compound **6** pure in 88% yield. Yield: 88% (78.2 mg), yellow solid, R*_f_*: 0.57, m.p.: 100–102 °C. ^1^H-NMR (DMSO-*d*_6_): δ 8.85 (1H, dd, *J* = 2.2 and 0.8 Hz, H-4), 8.48 (1H, d, *J* = 0.8 Hz, H-3), 8.27 (1H, dd, *J* = 9.3 and 2.2 Hz, H-6), 7.96 (1H, dt, *J* = 9.3 and 0.8 Hz, H-7), 4.71 (2H, t, *J* = 5.4 Hz, *N*-CH_2_), 3.83 (2H, t, *J* = 5.4 Hz, N_3_-CH_2_) ppm. ^13^C-NMR (DMSO-*d*_6_): δ 141.8 (C7a), 141.7 (C5), 136.8 (C3), 122.8 (C3a), 121.1 (C6), 119.1 (C4), 110.7 (C7), 50.2 (*N*-CH_2_), 48.00 (CH_2_-N_3_) ppm. MS-ESI(+): *m/z* 233.1 [M + H]^+^. Elemental analysis calcd (%) for C_9_H_8_N_6_O_2_.1/3 H_2_O C 45.38, H 3.67, N 35.28; found C 45.37, H 3.48, N 35.08.

### 3.4. General Procedure for 1,3-Dipolar Cycloaddition of Azides with Terminal Alkynes

To a stirred solution of azide **6** (0.1 g, 0.43 mmol) and the appropriate terminal alkynes (0.64 mmol) in 5 mL of a mixture H_2_O/*t*-BuOH (1:1), it was added copper sulphate (0.02 mmol) and sodium ascorbate (0.04 mmol). The reaction mixture was stirred at room temperature until the TLC control showed the total consumption of the starting material (12–16 h). After this period, the reaction mixture was washed with water and the organic phase was extracted with dichloromethane. The combined organic layers were dried with anhydrous sodium sulfate and the solvent was removed under reduced pressure. The desired products **7a**–**7d** were obtained pure after crystallization in ethanol. The compounds **8** and **9** were purified by column chromatography (silica gel) using hexane:ethyl acetate (1:1) as the eluent.

*5-Nitro-1-(2-(4-phenyl-1H-1,2,3-triazol-1-yl)ethyl)-1H-indazole*, **7a**. Yield: 74% (106.5 mg), white solid, R*_f_*: 0.30, m.p.: 182–184 °C. ^1^H-NMR (DMSO-*d*_6_): δ 8.78 (1H, d, *J* = 2.2 Hz, H-4), 8.41 (1H, d, *J* = 0.9 Hz, H-3), 8.39 (1H, s, H-8), 8.13 (1H, dd, *J* = 9.3 and 2.2 Hz, H-6), 7.79–7.59 (3H, m, H-7, H-11 and H-15), 7.50-–7.36 (2H, m, H-12 and H-14), 7.34–7.25 (1H, m, H-13), 5.08–5.04 (2H, m, *N*-CH_2_), 4.96-4.92 (2H, m, CH_2_-triazole) ppm. ^13^C-NMR (DMSO-*d*_6_): δ 146.3 (C9), 141.7 (C7a), 141,5 (C5), 137.0 (C3), 130.5 (C10), 128.9 (C12 and C12′), 127.9 (C13), 125.0 (C11 and C11′), 122.6 (C3a), 121.8 (C8), 120.9 (C6), 119.1 (C4), 110.2 (C7), 49.2 (*N*-CH_2_), 48.5 (CH_2_-triazole) ppm. MS-ESI(+): *m/z* 335.2 [M + H]^+^. Elemental analysis calcd (%) for C_17_H_14_N_6_O_2_.9/8 H_2_O C 57.58, H 4.62, N 23.70; found C 57.16, H 4.53, N 24.21.

*5-Nitro-1-(2-(4-p-tolyl-1H-1,2,3-triazol-1-yl)ethyl)-1H-indazole*, **7b**. Yield: 82% (123.0 mg), white solid, R*_f_*: 0.34, m.p.: 188–190 °C. MS-ESI(+): *m/z* 349.2 [M + H]^+^. ^1^H-NMR (CDCl_3_): δ 8.66 (1H, dd, *J* = 2.1 and 0.8 Hz, H-4), 8.28 (1H, d, *J* = 0.8 Hz, H-3), 8.13 (1H, dd, *J* = 9.2 and 2.1 Hz, H-6), 7.44 (2H, dd, *J* = 6.6 Hz, H-11 and H-15), 7.21–7.09 (4H, m, H-7, H-8, H-12 and H-14), 4.98 (4H, s, *N*-CH_2_-CH_2_-triazole), 2.33 (3H, s, CH_3_) ppm. ^13^C-NMR (CDCl_3_): δ 142.7 (C7a), 142.0 (C5), 138.3 (C9), 137.2 (C3), 129.5 (C12 and C12′), 127.0 (C13), 126.7 (C10), 125.5 (C11 and C11′), 122.8 (C3a), 122.1 (C6), 120.2 (C8), 118.8 (C4), 108.8 (C7), 49.5 (*N*-CH_2_), 48.9 (CH_2_-triazole), 21.3 (CH_3_) ppm.

*1-(2-(4-(4-Methoxyphenyl)-1H-1,2,3-triazol-1-yl)ethyl)-5-nitro-1H-indazole*, **7c**. Yield: 87% (136.5 mg), yellow solid, R*_f_*: 0.22, m.p.: 210–212 °C. ^1^H-NMR (DMSO-*d*_6_): δ 8.78 (1H, dd, *J* = 2.2 and 0.8 Hz, H-4), 8.41 (1H, d, *J* = 0.8 Hz, H-3), 8.28 (1H, s, H-8), 8.13 (1H, dd, *J* = 9.3 and 2.2 Hz, H-6), 7.69 (1H, dt, *J* = 9.3 and 0.8 Hz, H-7), 7.64–7.56 (2H, m, H-11 and H-15), 7.00–6.93 (2H, m, H-12 and H-14), 5.07–5.01 (2H, m, *N*-CH_2_), 4.98–4.90 (2H, m, CH_2_-trizole), 3.76 (CH_3_, s, 3H) ppm. ^13^C-NMR (DMSO-*d*_6_): δ 159.0 (C13), 146.2 (C9), 141.7 (C7a), 141,5 (C5), 137.0 (C3), 126.4 (C12 and C12′), 123.1 (C3a), 122.7 (C10), 120.9 (C8), 120.8 (C6), 119.1 (C4), 114.3 (C11 and C11′), 110.2 (C7), 55.2 (CH_3_), 49.2 (*N*-CH_2_), 48.5 (CH_2_-triazole) ppm. MS-ESI(+): *m/z* 365.2 [M + H]^+^. Elemental analysis calcd (%) for C_18_H_16_N_6_O_3_.3/2 H_2_O C 57.44, H 4.64, N 22.33; found C 57.75, H 4.52, N 21.97.

*5-Nitro-1-(2-(4-(4-nitrophenyl)-1H-1,2,3-triazol-1-yl)ethyl)-1H-indazole*, **7d**. Yield: 71% (116.0 mg), orange solid, R*_f_*: 0.15, m.p.: 281–283 °C. ^1^H-NMR (DMSO-*d*_6_): δ 8.79 (1H, dd, *J* = 2.2 and 0.8 Hz, H-4), 8.68 (1H, s, H-8), 8.40 (1H, d, *J* = 0.8 Hz, H-3), 8.35–8.25 (2H, m, H-12 and H-14), 8.14 (2H, dd, *J* = 9.3 and 2.2 Hz, H-6), 8.02-–7.93 (2H, m, H-11 and H-15), 7.73 (1H, dt, *J* = 9.3 and 0.8 Hz, H-7), 5.10-5.06 (2H, m, *N*-CH_2_), 5.00-4.96 (CH_2_-triazole) ppm. ^13^C-NMR (DMSO-*d*_6_): δ 146.6 (C13), 144.3 (C9), 141.7 (C7a), 141,5 (C5), 137.0 (C10), 136.9 (C3), 125.8 (C12 and C12′), 124.4 (C11 and C11′), 123.9 (C8), 122.7 (C3a), 120.9 (C6), 119.1 (C4), 110.2 (C7), 49.4 (*N*-CH_2_), 48.5 (CH_2_-triazole) ppm. MS-ESI(+): *m/z* 380.1 [M + H]^+^. Elemental analysis calcd (%) for C_17_H_13_N_7_O_4_.1/2 H_2_O C 52.58, H 3.63, N 25.25; found C 52.68, H 3.24, N 24.91.

*1-(2-(4-(3-Ethynylphenyl)-1H-1,2,3-triazol-1-yl)ethyl)-5-nitro-1H-indazole*, **8**. Yield: 23% (35.5 mg), white solid, R*_f_*: 0.32, m.p.: 176–178 °C. ^1^H-NMR (DMSO-*d*_6_): δ 8.79 (1H, d, *J* = 2.2 Hz, H-4), 8.48 (1H, s, H-8), 8.41 (1H, s, H-3), 8.13 (1H, dd, *J* = 9.3 and 2.2 Hz, H-6), 7.78–7.69 (3H, m, H-7, H-13 and H-15), 7.476–7.38 (2H, m, H-11 and H-14), 5.08–5.04 (2H, m, *N*-CH_2_), 4.96–4.92 (2H, m, CH_2_-triazole), 4.25 (1H, s, -C≡CH) ppm. ^13^C-NMR (DMSO-*d*_6_): δ 145.3 (C9), 141.7 (C7a), 141,5 (C5), 137.0 (C3), 131.00 (C11), 130.99 (C10), 129.4 (C14), 128.0 (C13), 125.5 (C15), 122.7 (C12), 122.5 (C3a), 122.3 (C8), 120.9 (C6), 119.1 (C4), 110.2 (C7), 83.1 (Ph-C≡), 81.2 (≡CH), 49.3 (*N*-CH_2_), 48.5 (CH_2_-triazole) ppm. MS-ESI(+): *m/z* 359.2 [M + H]^+^. Elemental analysis calcd (%) for C_19_H_14_N_6_O_2_.3/4 H_2_O C 61.37, H 4.20, N 22.60; found C 61.39, H 3.80, N 22.25.

*1,3-bis(1-(2-(5-Nitro-1H-indazol-1-yl)ethyl)-1H-1,2,3-triazol-4-yl)benzene*, **9**. Yield: 58% (73.9 mg), yellow solid, R*_f_*: 0.08, m.p.: 173–175 °C. ^1^H-NMR (DMSO-*d*_6_): δ 8.79 (2H, d, *J* = 2.1 Hz, H-4), 8.49 (2H, s, H-8), 8.41 (2H, s, H-3), 8.14 (2H, dd, *J* = 9.3 and 2.1 Hz, H-6), 8.10 (1H, t, *J* = 1.6 Hz, H-11), 7.73 (2H, d, *J* = 9.3 Hz, H-7), 7.61 (2H, dd, *J* = 7.7 and 1.6 Hz, 2H H-12 and H-14), 7.44 (1H, t, *J* = 7.7 Hz, H-13), 5.10–5.06 (4H, m, *N*-CH_2_), 4.97-4.94 (2H, m, CH_2_-triazole) ppm. ^13^C-NMR (DMSO-*d*_6_): δ 146.0 (C9), 141.7 (C7a), 141.5 (C5), 137.0 (C3), 131.1 (C10), 129.5 (C13), 124.5 (C12 and C14), 122.6 (C3a), 122.0 (C8), 121.5 (C11), 120.9 (C6), 119.1 (C4), 110.2 (C7), 49.2 (*N*-CH_2_), 48.5 (CH_2_-triazole) ppm. MS-ESI(+): *m/z* 591.3 [M + H]^+^. Elemental analysis calcd (%) for C_28_H_22_N_12_O_4_.2/3 H_2_O.1/3 C_4_H_10_O C 56.17, H 4.28, N 26.80; found C 56.17, H 3.96, N 26.46.

### 3.5. General Procedure for 1,3-Dipolar Cycloaddition Reactions of N-Vinyl-Nitroindazoles with Nitrile Imines to Give Access to Compounds ***12a**–**12e***

A solution of 5-nitro-1-vinyl-1*H*-indazole **3b** or 6-nitro-1-vinyl-1H-indazole, **3** (0.02 g, 0.10 mmol) and the appropriate hydrazonyl bromide **10a**–**10e** (1.5 equiv., 0.15 mmol) in dichloromethane (5 mL) was treated with cesium carbonate (0.20 mmol) and then stirred for 24 h at room temperature. After this period, the solvent was removed, and the crude product was purified by column chromatography on silica gel (EtOAc:hexane 2:8) to afford the corresponding products **12a**–**12e.**

*Ethyl 5-(5-nitro-1H-indazol-1-yl)-1-(p-tolyl)-4,5-dihydro-1H-pyrazole-3-carboxylate*, **12a**. Yield: 72% (29.9 mg), yellow solid, R*_f_*: 0.38, m.p.: 167–169 °C. ^1^H-NMR (DMSO-*d*_6_): δ 8.78 (1H, d, *J* = 2.0 Hz, H-4), 8.39 (1H, s, H-3), 8.34 (1H, dd, *J* = 9.3 and 2.0 Hz, H-6), 8.09 (1H, d, *J* = 9.3 Hz, H-7), 7.59 (1H, dd, *J* = 11.3 Hz and 3.1 Hz, H’), 7.06–7.98 (2H, m, H-9 and H-9′), 6.97–6.91 (2H, m, H-10 and H-10′), 4.31 (2H, q, *J* = 7.1 Hz, -OC*H*_2_CH_3_), 3.82 (1H, dd, *J* = 18.9 and 11.2 Hz, H′′′), 3.39–3.31 (1H, m, H′′), 2.09 (3H, s, Ph-CH_3_), 1.32 (3H, t, *J* = 7.1 Hz, -OCH_2_C*H*_3_) ppm. ^13^C-NMR (DMSO-*d*_6_): δ 161.6 (C=O), 142.1 (C7a), 140.8 (C12), 139.5 (C5), 138.6 (C3), 138.4 (C8), 131.0 (C11), 129.6 (C9 and C9′), 122.8 (C3a), 122.0 (C6), 119.4 (C4), 114.9 (C10 and C10′), 110.3 (C7), 72.1 (CH’), 60.8 (O-*C*H_2_CH_3_), 39.1 (CH_2_), 20.1 (Ph-CH_3_),14.2 (O-CH_2_*C*H_3_) ppm. MS-ESI(+): *m/z* 416.2 [M + Na]^+^. Elemental **analysis** calcd (%) for C_20_H_19_N_5_O_4_.1/2 H_2_O C 59.69, H 5.01, N 17.40; found C 59.30, H 4.71, N 17.83.

*Ethyl 5-(5-nitro-1H-indazol-1-yl)-1-(4-nitrophenyl)-4,5-dihydro-1H-pyrazole-3-carboxylate*, **12b**. Yield: 63% (28.3 mg), orange solid, R*_f_*: 0.22, m.p.: 183–185 °C. ^1^H-NMR (DMSO-*d*_6_): δ 8.82 (1H, d, *J* = 2.2 Hz, H-4), 8.44 (1H, s, H-3), 8.39 (1H, dd, *J* = 9.3 and 2.2 Hz, H-6), 8.17 (1H, d, *J* = 9.3 Hz, H-7), 8.08 (2H, d, *J* = 9.3 Hz, H-10 and H-10′), 7.76 (1H, dd, *J* = 10.9 Hz and 2.9 Hz, H’), 7.25 (H-9 and H-9′, d, *J* = 9.3 Hz, 2H), 4.35 (2H, q, *J* = 7.1 Hz, -OC*H*_2_CH_3_), 3.94 (1H, dd, *J* = 19.2 and 10.9 Hz, H′′′), 3.45–3071 (1H, m, H′′), 1.34 (3H, t, *J* = 7.1 Hz, -OCH_2_C*H*_3_) ppm. ^13^C-NMR (DMSO-*d*_6_): δ 161.0 (C=O), 146.2 (C11), 144.4 (C8), 142.4 (C7a), 141.0 (C12), 140.9 (C5), 138.9 (C3), 125.7 (C9 and C9′), 123.0 (C3a), 122.4 (C6), 119.5 (C4), 114.0 (C10 and C10′), 110.4 (C7), 71.4 (CH’), 61.4 (O-*C*H_2_CH_3_), 40.0 (CH_2_), 14.2 (O-CH_2_*C*H_3_) ppm. MS-ESI(+): *m/z* 447.1 [M + Na]^+^. Elemental analysis calcd (%) for C_19_H_16_N_6_O_6_ C 53.78, H 3.80, N 19.80; found C 53.84, H 4.17, N 20.07.

*Ethyl 1-(4-fluorophenyl)-5-(5-nitro-1H-indazol-1-yl)-4,5-dihydro-1H-pyrazole-3-carboxylate*, **12c**. Yield: 87% (36.5 mg), white solid, R*_f_*: 0.27, m.p.: 177–179 °C. ^1^H-NMR (DMSO-*d*_6_): δ 8.78 (1H, d, *J* = 2.2 Hz, H-4), 8.40 (1H, s, H-3), 8.34 (1H, dd, *J* = 9.3 and 2.2 Hz, H-6), 8.10 (1H, d, *J* = 9.3 Hz, H-7,), 7.60 (1H, H’, dd, *J* = 11.2 Hz and 3.0 Hz), 7.15–7.11 (2H, m, H-9 and H-9′), 7.07–6.97 (2H, m, H-10 and H-10′), 4.31 (2H, q, *J* = 7.1 Hz, -OC*H*_2_CH_3_), 3.83 (1H, dd, *J* = 18.9 and 11.2 Hz, H′′′), 3.41-3.40 (1H, m, H′′), 1.32 (3H, t, *J* = 7.1 Hz, -OCH_2_C*H*_3_) ppm. ^13^C-NMR (DMSO-*d*_6_): δ 161.5 (C=O), 142.1 (C7a), 140.9 (C12), 140.5 (C5), 138.5 (C3), 137.6 (C8), 125.7 (C11), 122.8 (C3a), 122.1 (C6), 119.4 (C4), 116.7, 116.6 (C9 and C9′), 116.0, 115.7 (C10 and C10′), 110.3 (C7), 72.4 (CH’), 60.9 (O-*C*H_2_CH_3_), 39.3 (CH_2_), 14.2 (O-CH_2_*C*H_3_) ppm. MS-ESI(+): *m/z* 420.2 [M + Na]^+^. Elemental analysis calcd (%) for C_19_H_16_FN_5_O_4_.1/2 H_2_O C 56.16, H 4.24, N 17.23; found C 56.48, H 4.24, N 17.06. 

*Ethyl 1-(4-chlorophenyl)- 5-(5-nitro-1H-indazol-1-yl)- 4,5-dihydro-1H-pyrazole-3-carboxylate*, **12d**. Yield: 83% (36.3 mg), white solid, R*_f_*: 0.30, m.p.: 179–181 °C. ^1^H-NMR (DMSO-*d*_6_): δ 8.80 (1H, d, *J* = 2.2 Hz, H-4), 8.41 (1H, s, H-3), 8.35 (1H, dd, *J* = 9.3 and 2.2 Hz, H-6), 8.11 (1H, d, *J* = 9.3 Hz, H-7), 7.62 (1H, dd, *J* = 11.0 Hz and 3.0 Hz, H’), 7.23–7.18 (2H, m, H-9 and H-9′), 7.15–7.10 (2H, m, H-10 and H-10′), 4.32 (2H, q, *J* = 7.1 Hz, -OC*H*_2_CH_3_), 3.85 (1H, dd, *J* = 19.1 Hz and 11.0 Hz, H′′′), 3.40 (1H, dd, *J* = 19.1 and 3.0 Hz, H′′), 1.33 (3H, t, *J* = 7.1 Hz, -OCH_2_C*H*_3_) ppm. ^13^C-NMR (DMSO-*d*_6_): δ 161.3 (C=O), 142.2 (C7a), 141.1 (C12), 140.8 (C5), 139.9 (C8), 138.6 (C3), 129.1 (C9 and C9′), 125.7 C11), 122.9 (C3a), 122.2 (C6), 119.4 (C4), 116.3 (C10 and C10′), 110.3 (C7), 72.0 (CH’), 61.0 (O-*C*H_2_CH_3_), 39.4 (CH_2_), 14.2 (O-CH_2_*C*H_3_) ppm. MS-ESI(+): *m/z* 436.1 [M + Na]^+^. Elemental analysis calcd (%) for C_19_H_16_ClN_5_O_4_.1/6 H_2_O C 54.75, H 3.95, N 16.80; found C 54.28, H 4.27, N 17.30.

*Ethyl 1-(4-bromophenyl)-5-(5-nitro-1H-indazol-1-yl)-4,5-dihydro-1H-pyrazole-3-carboxylate*, **12e**. Yield: 68% (32.9 mg), white solid, R*_f_*: 0.32, m.p.: 152–154 °C. ^1^H-NMR (DMSO-*d*_6_): δ 8.80 (1H, d, *J* = 2.2 Hz, H-4), 8.41 (1H, s, H-3), 8.35 (1H, dd, *J* = 9.3 and 2.2 Hz, H-6), 8.10 (1H, d, *J* = 9.3 Hz, H-7), 7.62 (1H, dd, *J* = 11.1 Hz and 3.0 Hz, H’), 7.37–7.27 (2H, m, H-9 and H-9′), 7.13-7.01 (2H, m, H-10 and H-10′), 4.32 (2H, q, *J* = 7.1 Hz, -OC*H*_2_CH_3_), 3.85 (1H, dd, *J* = 19.0 and 11.1 Hz, H′′′), 3.40–3.39 (1H, m, H′′), 1.33 (3H, t, *J* = 7.1 Hz, -OCH_2_C*H*_3_) ppm. ^13^C-NMR (DMSO-*d*_6_): δ 161.3 (C=O), 142.2 (C7a), 141.1 (C12), 140.8 (C5), 140.3 (C8), 138.6 (C3), 131.9 (C9 and C9′), 122.9 (C3a), 122.2 (C6), 119.4 (C4), 116.7 (C10 and C10′), 113.6 (C11), 110.3 (C7), 71.9 (CH’), 61.1 (O-*C*H_2_CH_3_), 39.4 (CH_2_), 14.2 (O-CH_2_*C*H_3_) ppm. MS-ESI(+): *m/z* 482.1 [M + Na]^+^. Elemental analysis calcd (%) for C_19_H_16_BrN_5_O_4_ C 49.80, H 3.52, N 15.28; found C 50.08, H 3.64, N 15.31.

*Ethyl 1-(4-chlorophenyl)-5-(6-nitro-1H-indazol-1-yl)-4,5-dihydro-1H-pyrazole-3-carboxylate*, **13**. Yield: 81% (35.4 mg), white solid, R*_f_*: 0.35, m.p.: 150–152 °C. ^1^H-NMR (DMSO-*d*_6_); δ 9.05 (1H, s, H-7), 8.33 (1H, s, H-3), 8.00 (1H, s, H-5 and H-4), 7.77 (1H, dd, *J* = 11.0, 2.7 Hz, H’), 7.20 (1H, d, *J* = 8.9 Hz, H-9 and H-9′), 7.15 (1H, d, *J* = 8.9 Hz, H-10 and H-10′), 4.32 (2H, q, *J* = 7.1 Hz, -OC*H*_2_CH_3_), 3.83 (1H, dd, *J* = 19.0 and 10.9 Hz, H′′′), 3.32–3.31 (1H, m, H′′), 1.32 (3H, t, *J* = 7.1 Hz, -OCH_2_C*H*_3_) ppm. ^13^C-NMR (DMSO-*d*_6_): δ 161.4 (C=O), 146.7 (C7a), 141.0 (C12), 140.0 (C8), 138.0 (C6), 136.4 (C3), 129.1 (C9 and C9′), 126.7 (C11), 125.7 (C3a), 122.8 (C4), 116.4 (C10 and C10′), 115.9 (C5), 106.6 (C7), 71.8 (CH’), 61.0 (O-CH_2_), 39.6 (CH_2_), 14.2 (CH_3_) ppm. MS-ESI(+): *m/z* 436.1 [M + Na]^+^. Elemental analysis calcd (%) for C_19_H_16_ClN_5_O_4_ C 55.12, H 3.90, N 16.92; found C 54.88, H 4.03, N 16.60.

### 3.6. Single-Crystal X-Ray Diffraction Studies

Single crystals of compounds **7b**, **9** and **13** were manually harvested from the crystallization vials and immersed in highly viscous FOMBLIN Y perfluoropolyether vacuum oil (LVAC 140/13, Sigma-Aldrich) to avoid degradation caused by the evaporation of the solvent [53]. Crystals were mounted on either Hampton Research CryoLoops or MiTeGen MicroLoops, typically with the help of a Stemi 2000 stereomicroscope equipped with Carl Zeiss lenses (San Francisco, CA, USA).

X-ray diffraction data for **9** and **13** were collected at 150(2)K on a D8 QUEST system (Bruker, Flawil, Switzerland) equipped with a Mo Kα sealed tube (λ = 0.71073 Å), a multilayer TRIUMPH X-ray mirror (Concord, CA, USA), a PHOTON 100 CMOS detector, and an Oxford Instruments Cryostrem 700+ Series low temperature device (Grenoble, Switzerland). Crystal data for **7b** was instead collected at 150(2)K on a Bruker X8 Kappa APEX II CCD area-detector diffractometer (Mo Kα graphite-monochromated radiation, λ = 0.71073 Å, controlled by the APEX3 software package (Bruker, Wissembourg, France) [54] and equipped with an Oxford Cryosystems Series 700 cryostream monitored remotely using the software interface Cryopad [55]. In both cases, diffraction images were processed using the software package SAINT+ [56], and data were corrected for absorption by the multiscan semi-empirical method implemented in SADABS 2016/2 [57].

Structures were solved using the algorithm implemented in SHELXT-2014/5 [58], which allowed the immediate location of almost all of the heaviest atoms composing the asymmetric unit of the three compounds. The remaining missing and misplaced non-hydrogen atoms were located from difference Fourier maps calculated from successive full-matrix least-squares refinement cycles on *F*^2^ using the latest SHELXL from the 2018/3 release [59], All structural refinements were performed using the graphical interface ShelXle [60]. 

Hydrogen atoms bound to carbon and oxygen were placed at their idealized positions using appropriate *HFIX* instructions in SHELXL: *43* (aromatic carbon atoms), *13* (tertiary carbon atoms), *23* (–CH_2_– carbon atoms) and *137* (for terminal methyl groups). These hydrogen atoms were included in subsequent refinement cycles with isotropic thermal displacements parameters (*U*_iso_) fixed at 1.2 (for the three first families of groups) or 1.5×*U*_eq_ (for the methyl groups) of the parent non-hydrogen atoms. Structural drawings have been created using the software package Crystal Impact Diamond [61]. 

The last difference Fourier map synthesis showed: for **7b**, the highest peak (0.199 eÅ^−3^) and the deepest hole (-0.191 eÅ^−3^) located at 0.91 and 0.83 Å from C13 and C11, respectively; for **9**, the highest peak (0.225 eÅ^−3^) and the deepest hole (-0.256 eÅ^−3^) located at 0.87 and 0.41 Å from C24 and H19, respectively; and for **13**, the highest peak (0.310 eÅ^−3^) and the deepest hole (-0.631 eÅ^−3^) located at 1.00 and 1.01 Å from Cl1.

Crystal data for **7b**: C_18_H_16_N_6_O_2_, *M* = 348.37, monoclinic, space group *P*2_1_/n, *Z* = 4, *a* = 9.4190(7) Å, *b* = 5.4701(4) Å, *c* = 32.403(3) Å, β = 95.259(5)°, *V* = 1662.5(2) Å^3^, μ(Mo-Kα) = 0.096 mm^−1^, *D*_c_ = 1.392 g cm^−3^, colourless plate with crystal size of 0.16 × 0.06 × 0.04 mm^3^. Of a total of 26,545 reflections collected, 3038 were independent (*R*_int_ = 0.0761). Final *R*1 = 0.0504 [*I* > 2*σ(I)*] and *wR*2 = 0.1242 (all data). Data completeness to theta = 25.24°, 99.8%. CCDC 1957399.

Crystal data for **9**: C_28_H_22_N_12_O_4_, *M* = 590.57, triclinic, space group *P*-1, *Z* = 2, *a* = 5.2677(7) Å, *b* = 13.2391(17) Å, *c* = 19.680(3) Å, α = 73.232(4)º, β = 87.663(4)°, *γ* = 86.022(4)°, *V* = 1310.6(3) Å^3^, *μ*(Mo-Kα) = 0.107 mm^−1^, *D*_c_ = 1.496 g cm^−3^, colourless needle with crystal size of 0.27 × 0.09 × 0.06 mm^3^. Of a total of 21,961 reflections collected, 4765 were independent (*R*_int_ = 0.0507). Final *R*1 = 0.0619 [*I* > 2*σ(I)*] and *wR*2 = 0.1275 (all data). Data completeness to theta = 25.24°, 99.7%. CCDC 1957400.

Crystal data for **13**: C_19_H_16_ClN_5_O_4_, *M* = 413.82, monoclinic, space group *P*2_1_, *Z* = 2, *a* = 12.6613(17) Å, *b* = 4.9988(6) Å, *c* = 14.716(2) Å, β = 102.355(4)°, *V* = 909.8(2) Å^3^, *μ*(Mo-Kα) = 0.249 mm^−1^, *D*_c_ = 1.511 g cm^−3^, colourless needle with crystal size of 0.21 × 0.07 × 0.03 mm^3^. Of a total of 12,618 reflections collected, 3298 were independent (*R*_int_ = 0.0520). Final *R*1 = 0.0550 [*I* > 2*σ(I)* and *wR*2 = 0.1331 (all data). Data completeness to theta = 25.24°, 99.3%. CCDC 1957398.

## 4. Conclusions

In summary, the *N*-alkylation of nitroindazole derivatives with dibromoethane afforded *N*-(2-bromoethyl)- and *N*-vinyl-nitro-1*H*-indazoles. The distribution of the *N*-substituted derivatives depends on the experimental conditions although the *N*-1 bromoethyl derivatives are always isolated as the major components. The elimination reaction at *N*-2 seems to be more favourable than the elimination reaction at the *N*-1 position. Both types of derivatives showed to be excellent templates for further functionalization via 1,3-dipolar cycloaddition approaches. 

The *N*-bromoethylnitroindazole derivative **2c** after being efficiently converted into the corresponding azide **6** afforded, in the presence of terminal ethynylbenzene derivatives and under CuAAC conditions, the expected triazolo derivatives in yields ranging from 71 to 87%. This reaction seems to be favoured by the presence of electron-donating groups in the ethynylbenzene derivative. 

The reaction of the *N*-vinyl-nitroindazole **3b** with *N*-aryl-*C*-ethoxycarbonylnitrile imines generated in situ from ethyl hydrazono-α-bromoglyoxylates, afforded the corresponding nitroindazole-pyrazoline derivatives **12** with yields ranging from 63 to 87%. The presence of the nitro group in a different position of the indazole core did not seem to affect its reactivity as dipolarophile since derivative **13** was also obtained in excellent yield from the nitroindazole **3c**. The expected regioselectivity in these cycloaddition reactions was further supported by single-crystal X-ray diffraction analysis with some single crystals of the compounds obtained. 

## Figures and Tables

**Table 1:** Conditions studied for the optimization of the *N*-alkylation reaction of compound **1b** and yields of compounds **2b**–**4b**.

Entry	Solvent	Base	Time (h)	Yield (%)			
				2b	3b	4b	5b
1	Acetone	KOH	48	39	9	12	25
2	Acetone	KOH ^a^	4	29	25	Traces	17
3	Acetone	KOH ^b^	2	27	21	Traces	19
4	THF	KOH ^b^	72	30	11	14	10
5	MeOH	KOH ^b^	120 ^c^	15	Traces	7	Traces
6	Acetone	Cs_2_CO_3_	1	35	6	12	15
7	Acetone	K_2_CO_3_	68	47	10	20	8

^a^ 3 equiv. KOH; ^b^ 3 equiv. KOH, reflux. ^c^ Recovery of a significant amount of starting material **1b.**

**Table 2:** Copper(I)-catalysed azide-alkyne cycloaddition reactions of compound **6** with a series of ethynylbenzene derivatives.

Entry	Product	Ar	Time (h)	Yield (%)
1	**7a**	Ph	12	74
2	**7b**	*p*-Me-Ph	12	82
3	**7c**	*p*-OMe-Ph	14	87
4	**7d**	*p*-NO_2_-Ph	16	71

## Supplementary Materials

The following are available online at https://www.mdpi.com/1420-3049/25/1/126/s1, Copies Figures S1 to S124: Copies of ^1^H, ^13^C, 2D NMR and MS spectra of compounds **2a**–**2d**, **3a**–**2d**, **4a**–**4d**, **5a**–**5d**, **6**, **7a**–**5d**, **8**, **9**, **12a**–**12e** and **13**.
