# Supplementary material for: A Suitable Functionalization of Nitroindazoles with Triazolyl and Pyrazolyl Moieties via Cycloaddition Reactions
Source: Molecules. 2019 Dec 28;25(1):126. doi: 10.3390/molecules25010126 (PMC6983193; doi:10.3390/molecules25010126)
Supplement: Supplementary file 1 [file molecules-25-00126-s001.pdf]

Supporting Information

A suitable functionalization of nitroindazoles with triazolyl and pyrazolyl moieties *via* cycloaddition reactions

Mohammed Eddahmi,[1,2] Nuno M. M. Moura,[2] Latifa Bouissane,[1] Ouafa Amiri,[1] Maria A. F. Faustino,[2] José A. S. Cavaleiro,[2] Ricardo F. Mendes,[3] Filipe Paz,[3] Maria G. P. M. S. Neves,[2] El Mostapha Rakib[1]

[1] Laboratory of Organic and Analytic Chemistry, Faculty of Sciences and Technics, Sultan Moulay Slimane University, BP 523, 2300 Beni-Mellal, Morocco.

[2] QOPNA & LAQV-REQUIMTE, Chemistry Department, University of Aveiro, 3810-193 Aveiro, Portugal.

[3] CICECO - Aveiro Institute of Materials, Chemistry Department, University of Aveiro, 3810-193 Aveiro, Portugal.

Table of contents

I - NMR and mass spectrum of compounds **2a-d**, **3a-d, 4a-d** and **5a-d**……………………... **S2**

II - NMR and mass spectrum of compounds **6**, **7a-d**, **8** and **9**……………........................... **S36**

III - NMR and mass spectrum of compounds **12a-e**…………………………………….……... **S51**

IV - NMR and mass spectrum of compound **13**……..…………….………………….……... **S62**

I - NMR and mass spectrum of compounds 2a-d, 3a-d, 4a-d and 5a-d

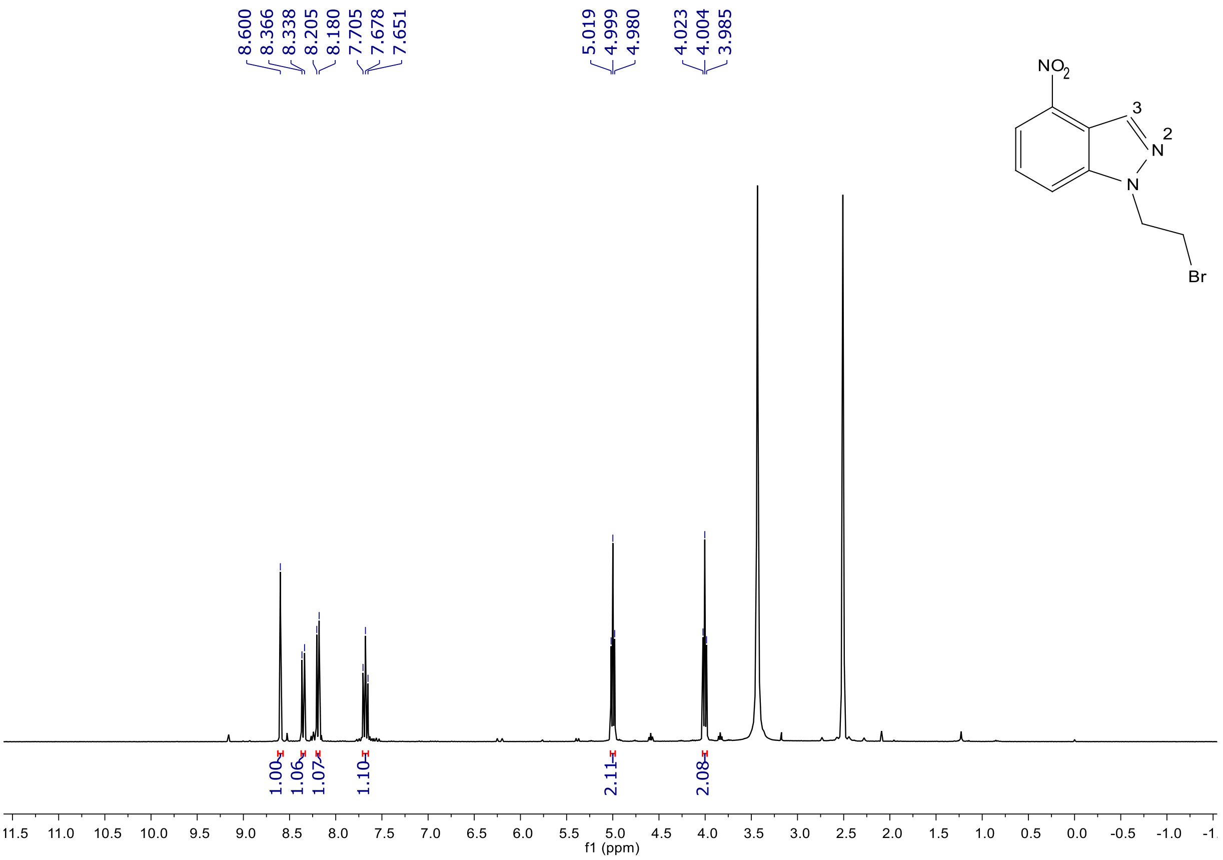

Figure S1. ^{1}H NMR spectrum of compound **2a** in DMSO-d6.

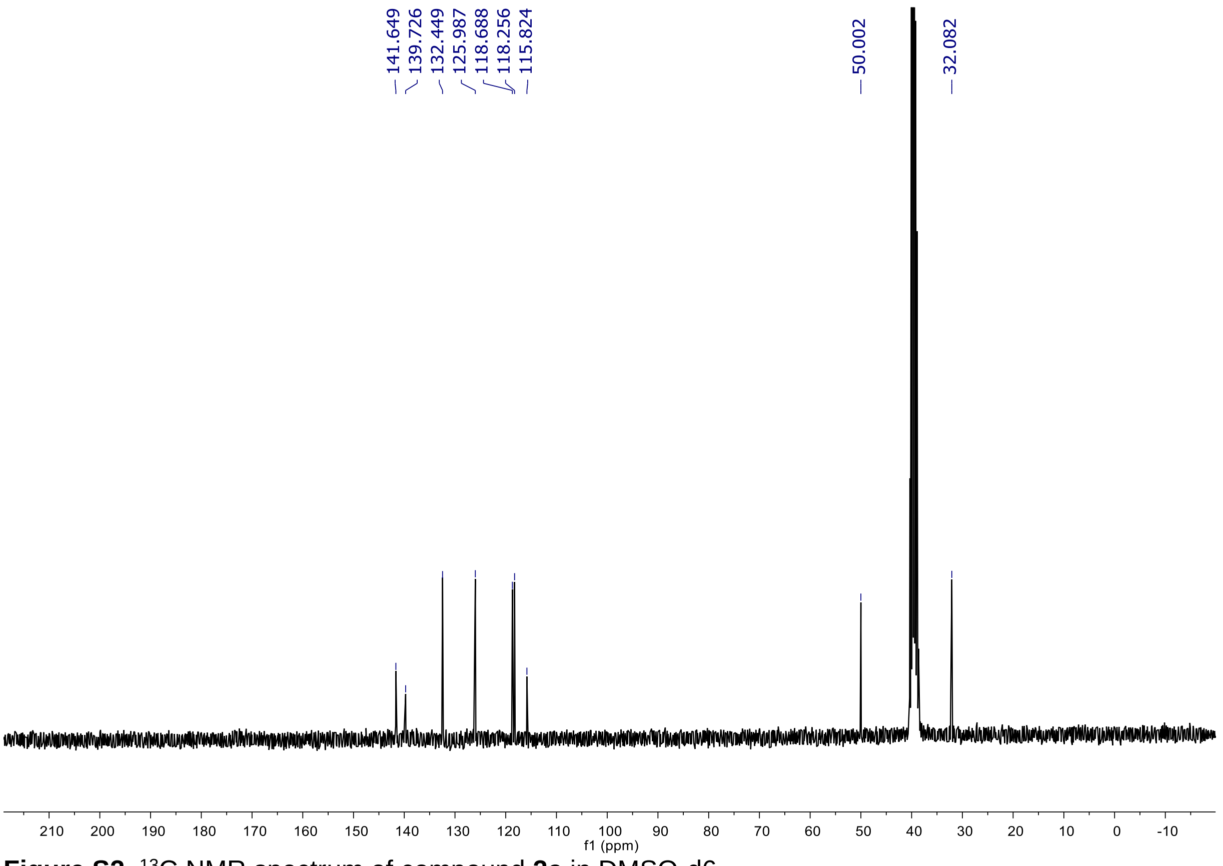

Figure S2. ^{13}C NMR spectrum of compound **2a** in DMSO-d6.

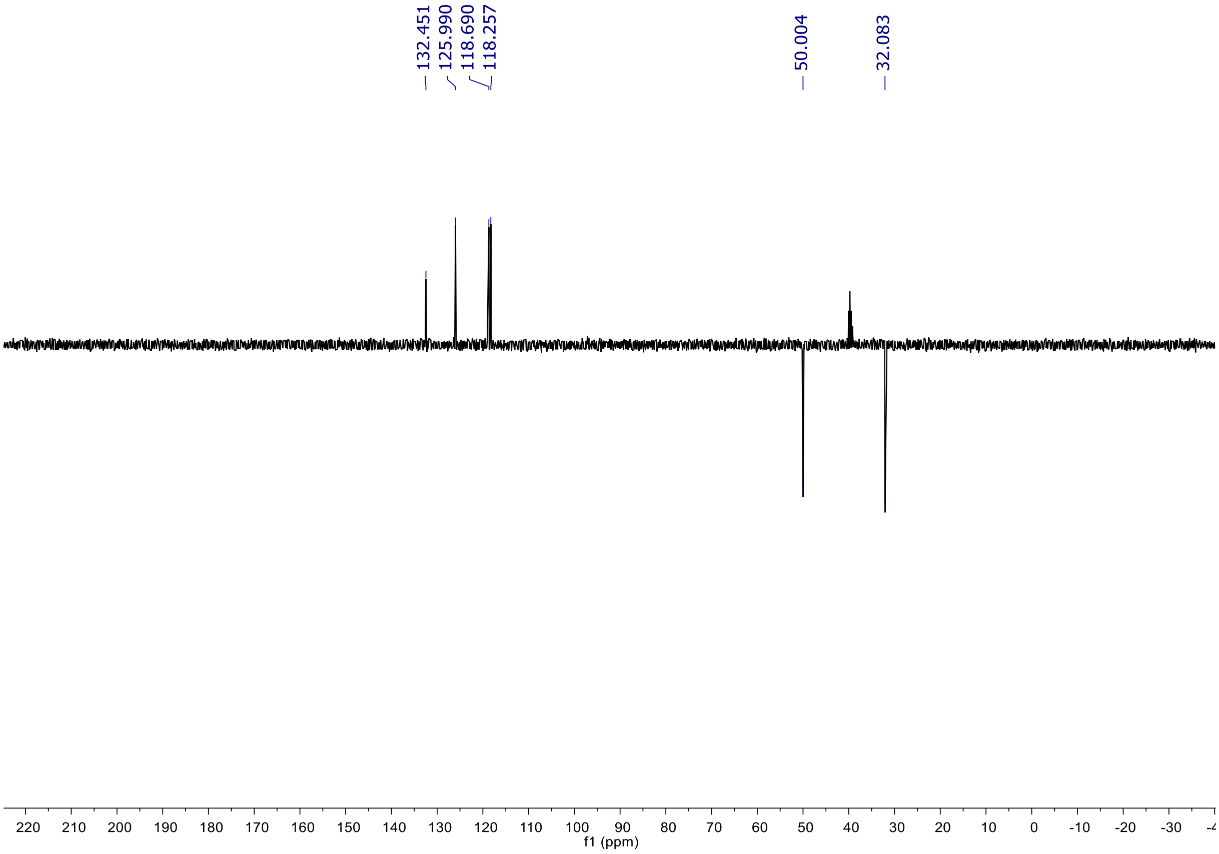

Figure S3. ^{13}C NMR DEPT 135 spectrum of compound **2a** in DMSO-d_6.

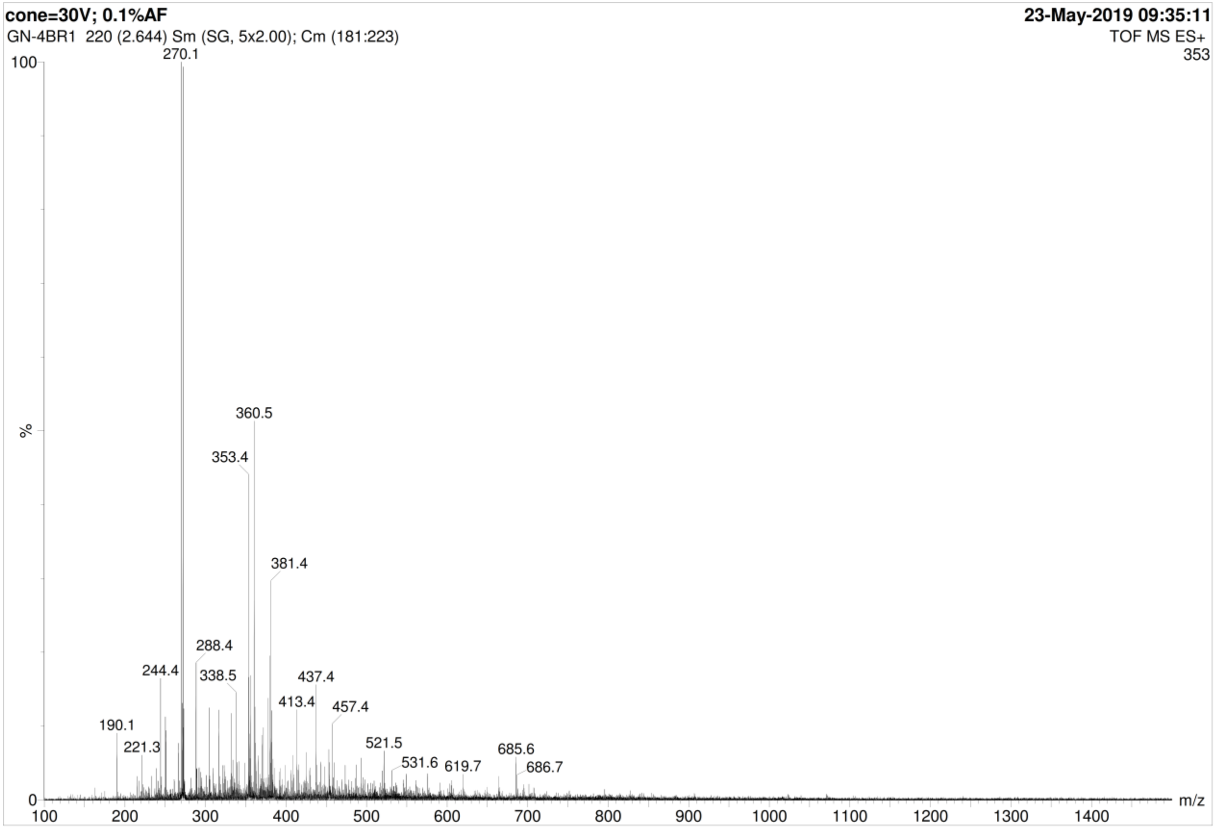

Figure S4. MS-ESI(+) spectrum of compound **2a.**

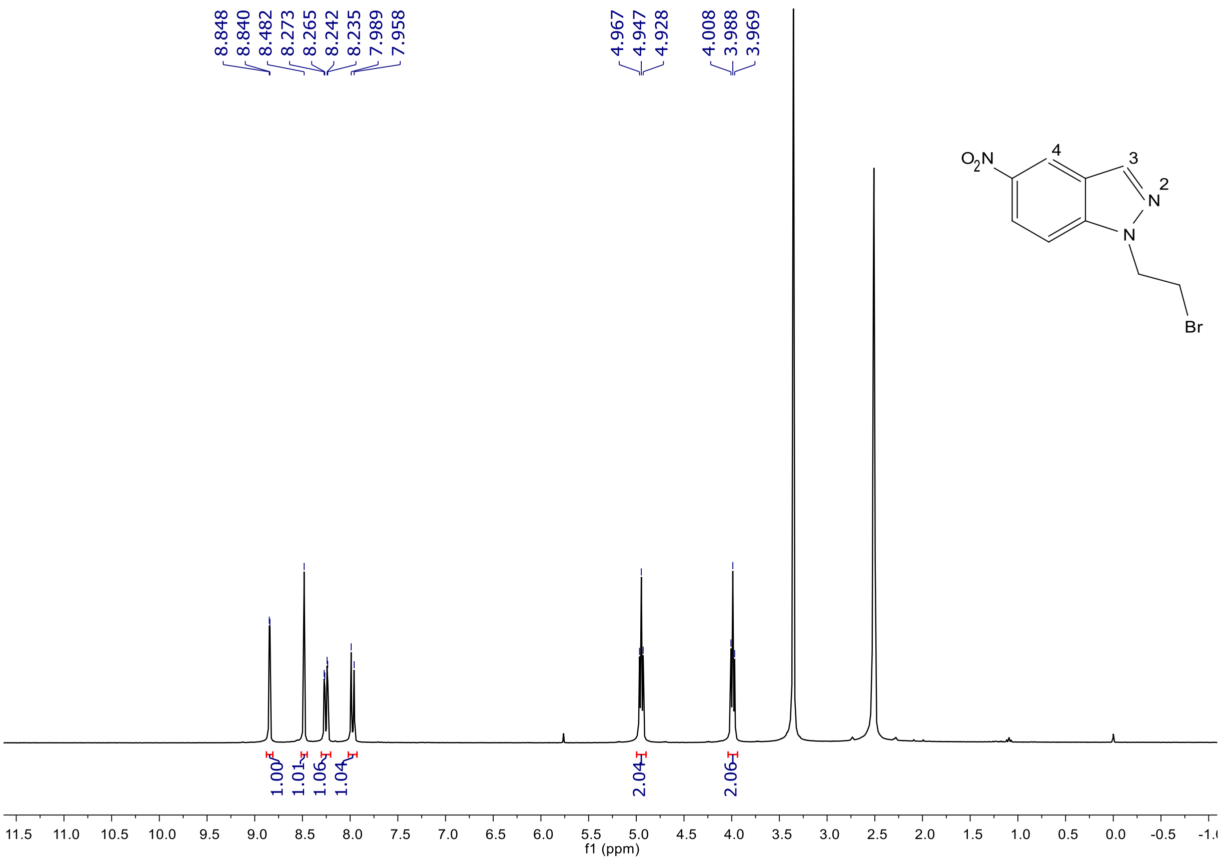

Figure S5. ¹H NMR spectrum of compound **2b** in DMSO-d6.

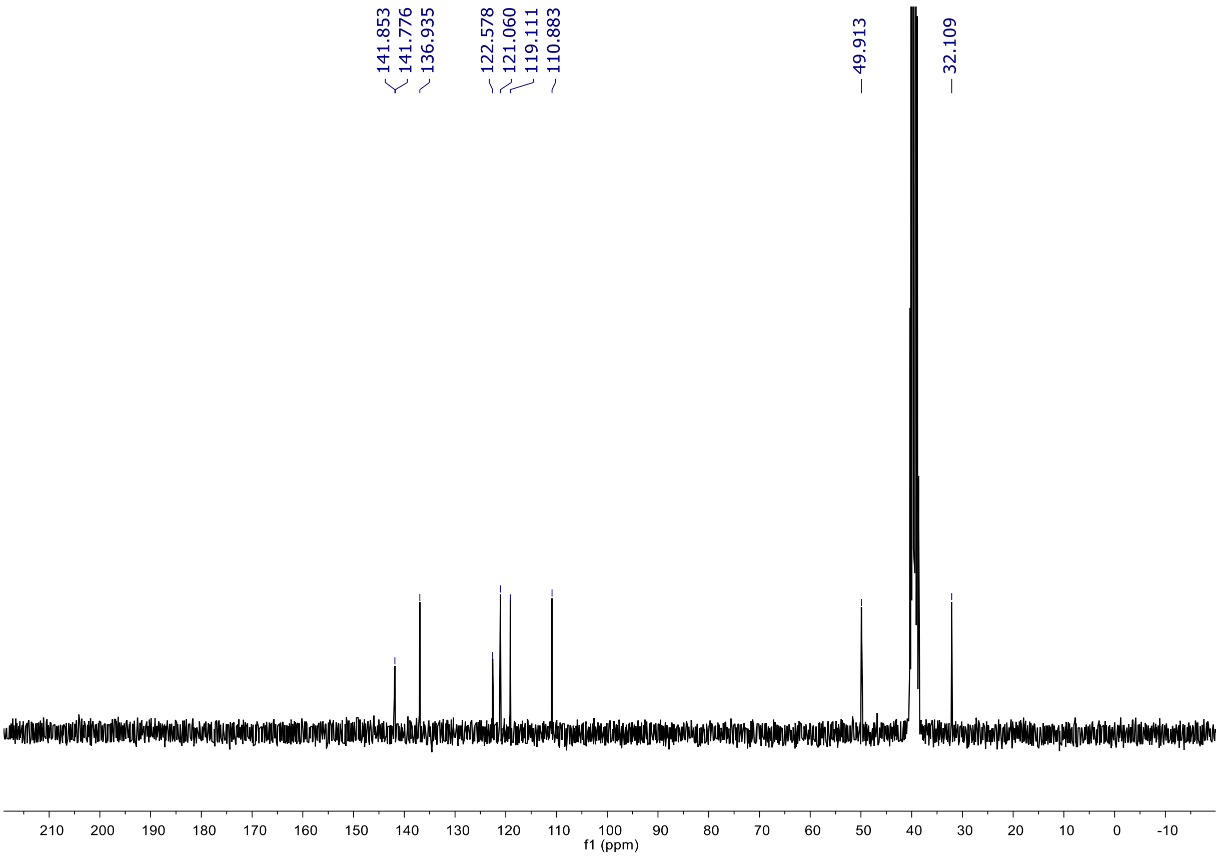

Figure S6. ¹³C NMR spectrum of compound **2b** in DMSO-d6.

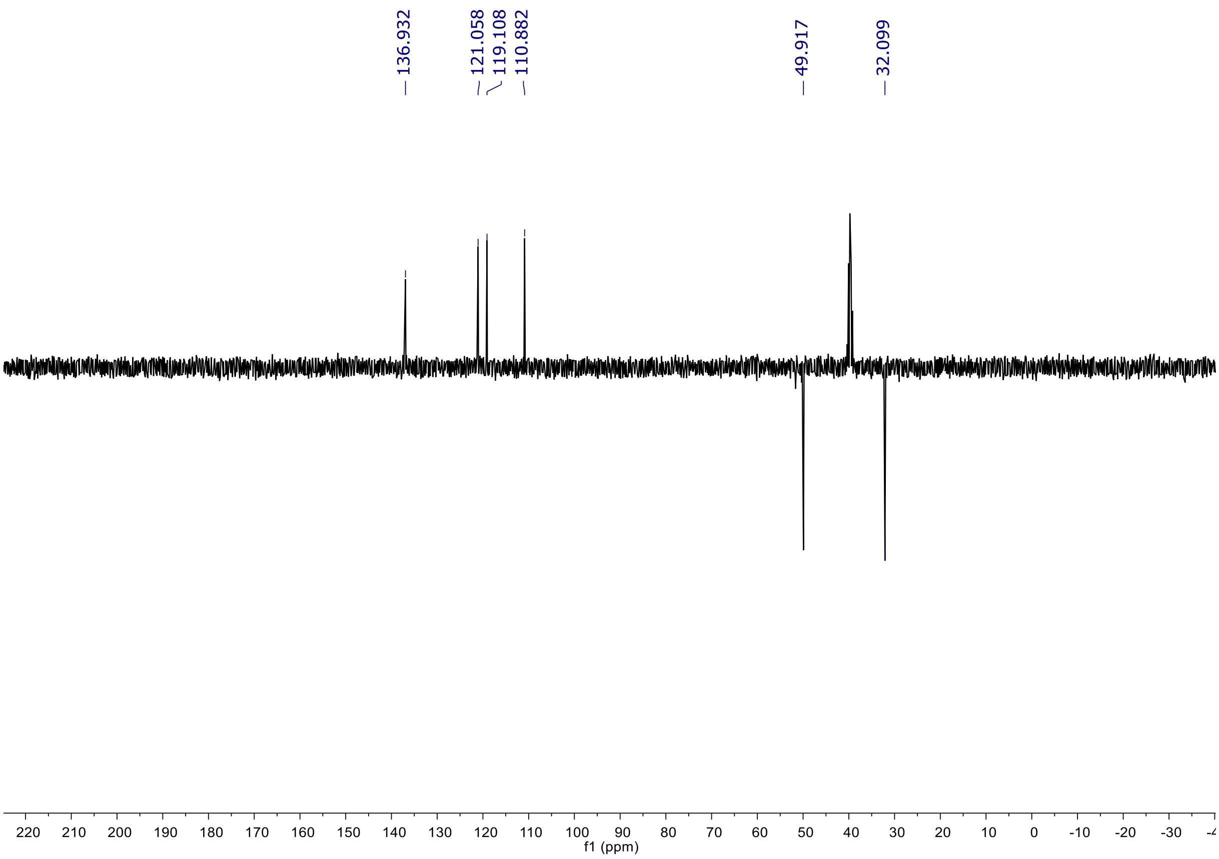

Figure S7. ^{13}C NMR DEPT 135 spectrum of compound **2b** in DMSO-d_6.

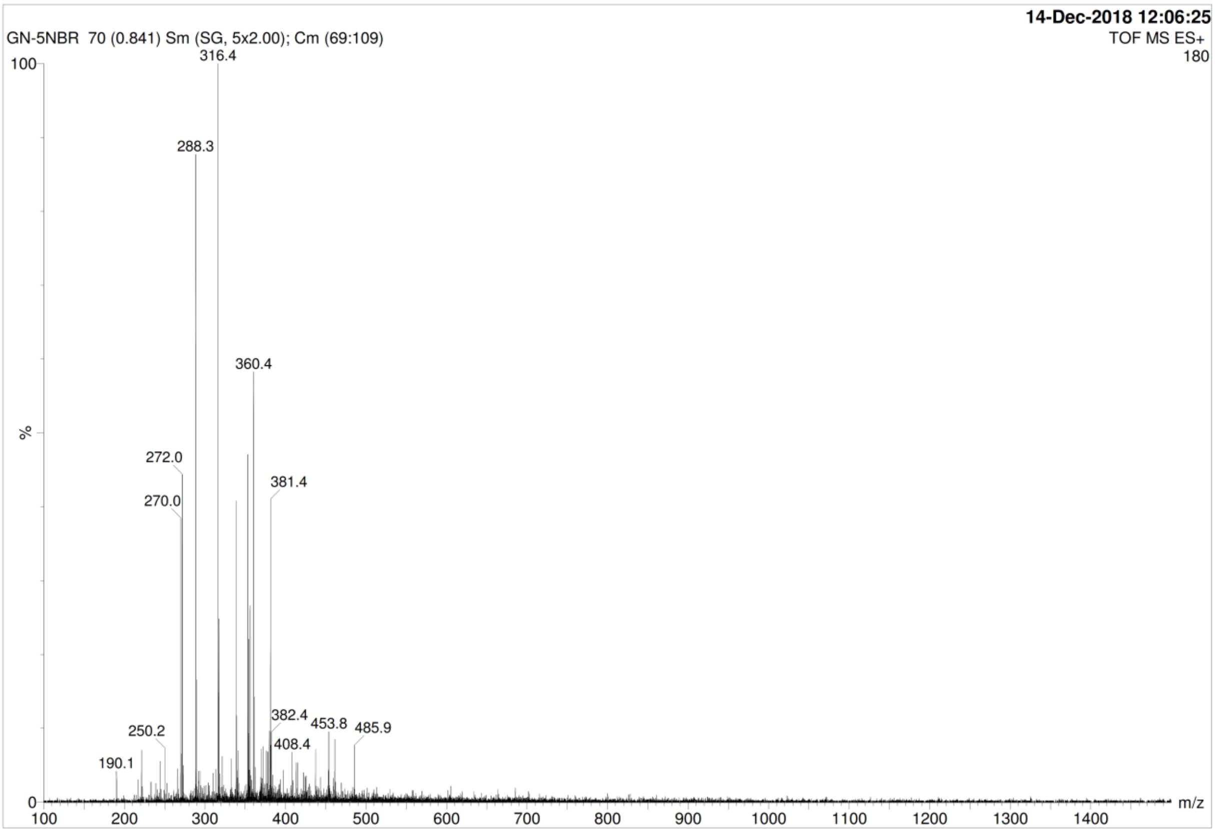

Figure S8. MS-ESI(+) spectrum of compound **2b**.

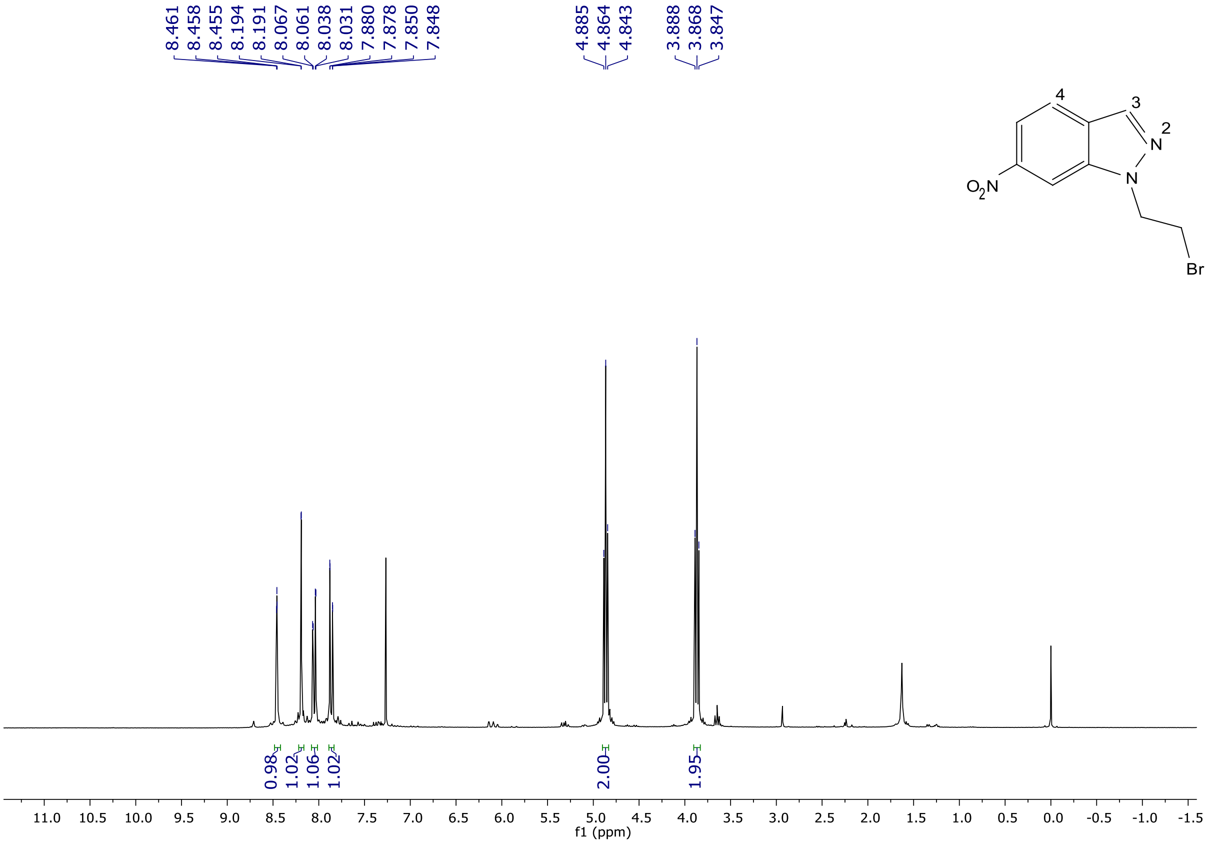

Figure S9. ^{1}H NMR spectrum of compound **2c** in $CDCl_3$.

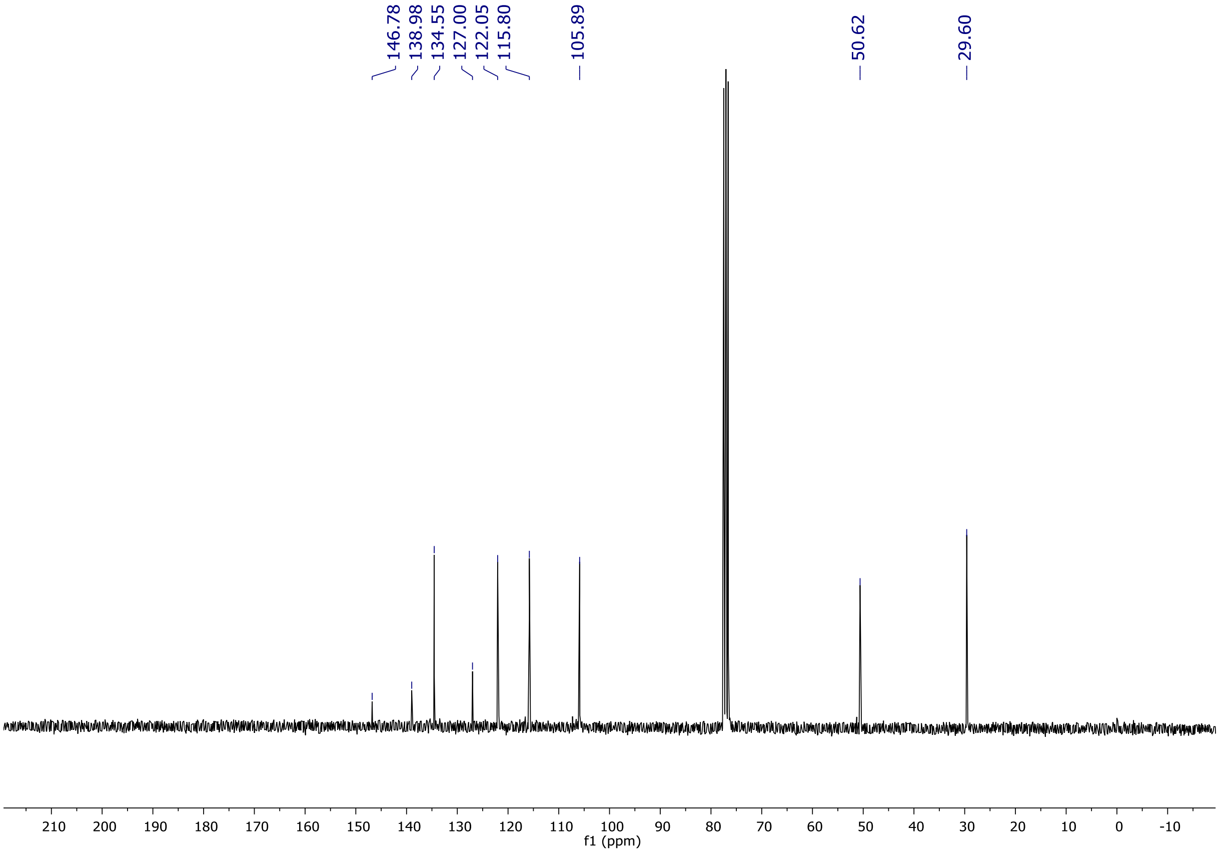

Figure S10. ^{13}C NMR spectrum of compound **2c** in $CDCl_3$.

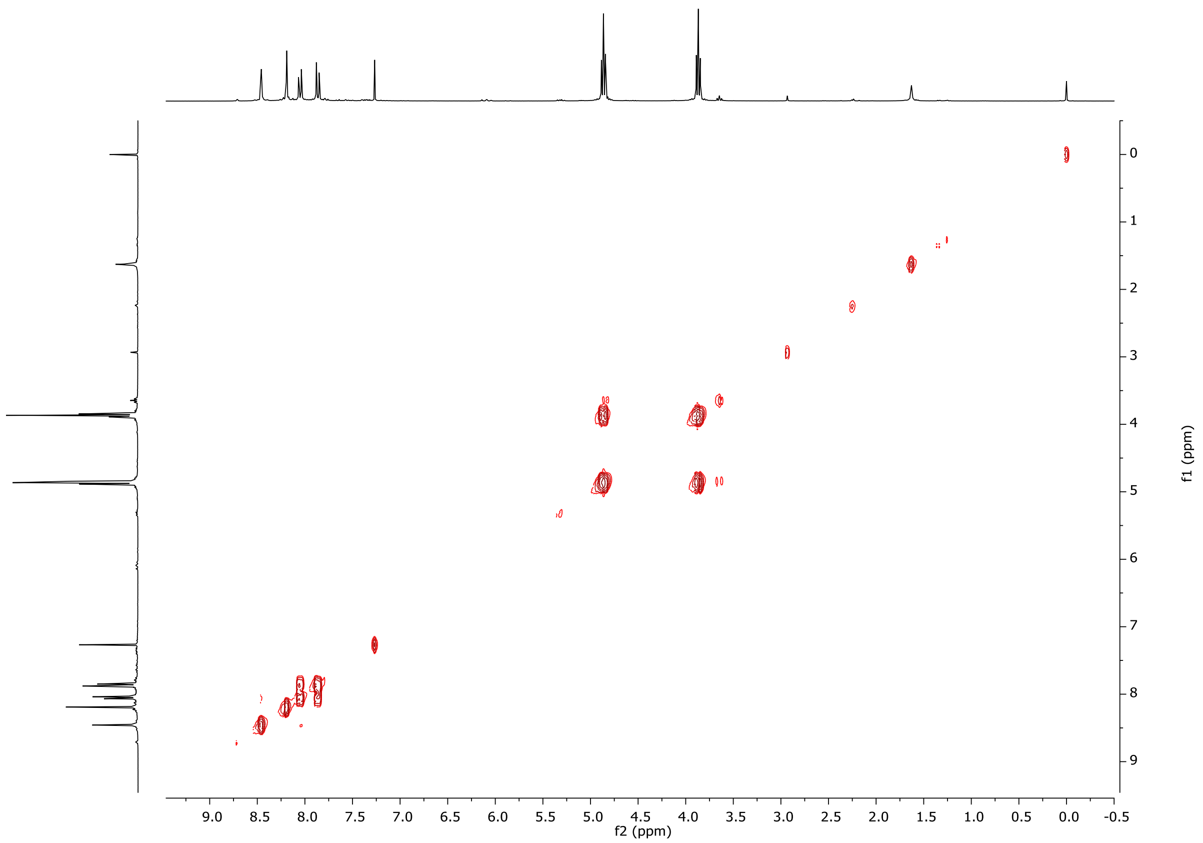

Figure S11. COSY ($^1H/^1H$) spectrum of compound **2c** in $CDCl_3$.

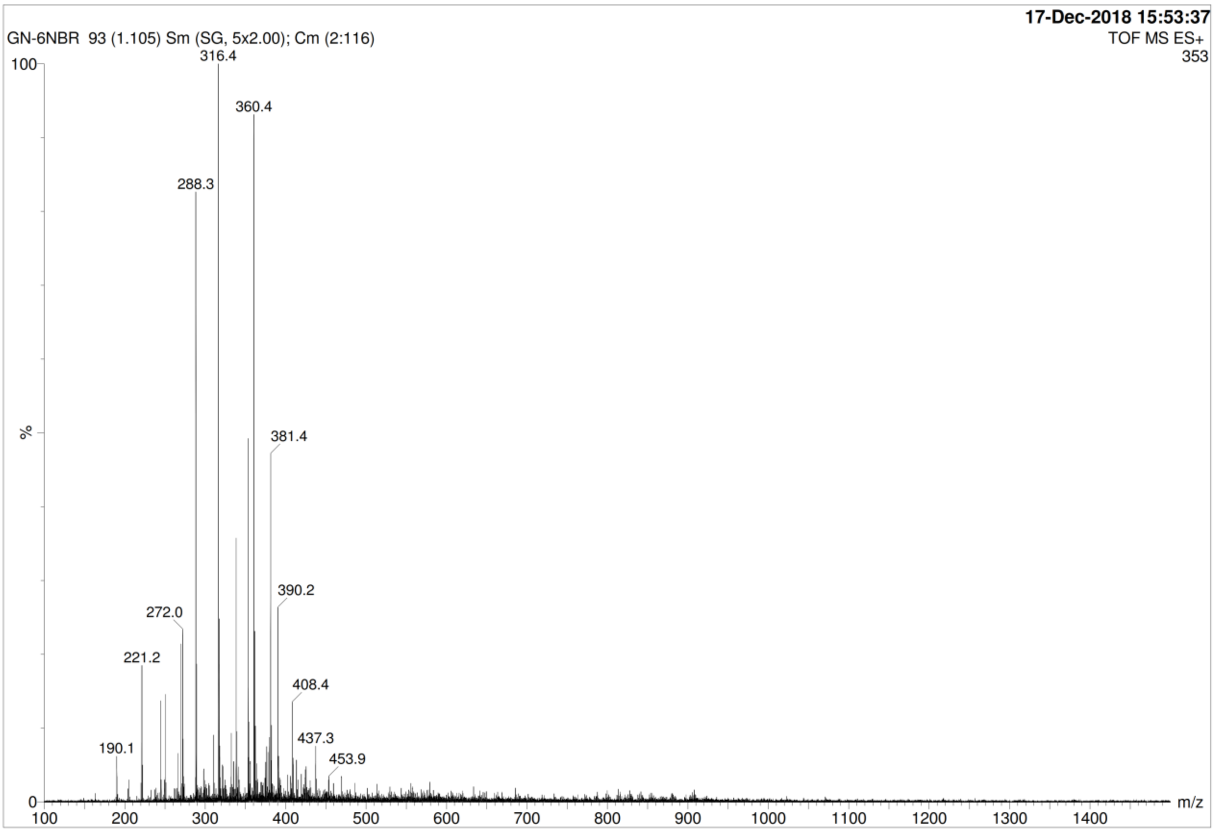

Figure S12. MS-ESI(+) spectrum of compound **2c**.

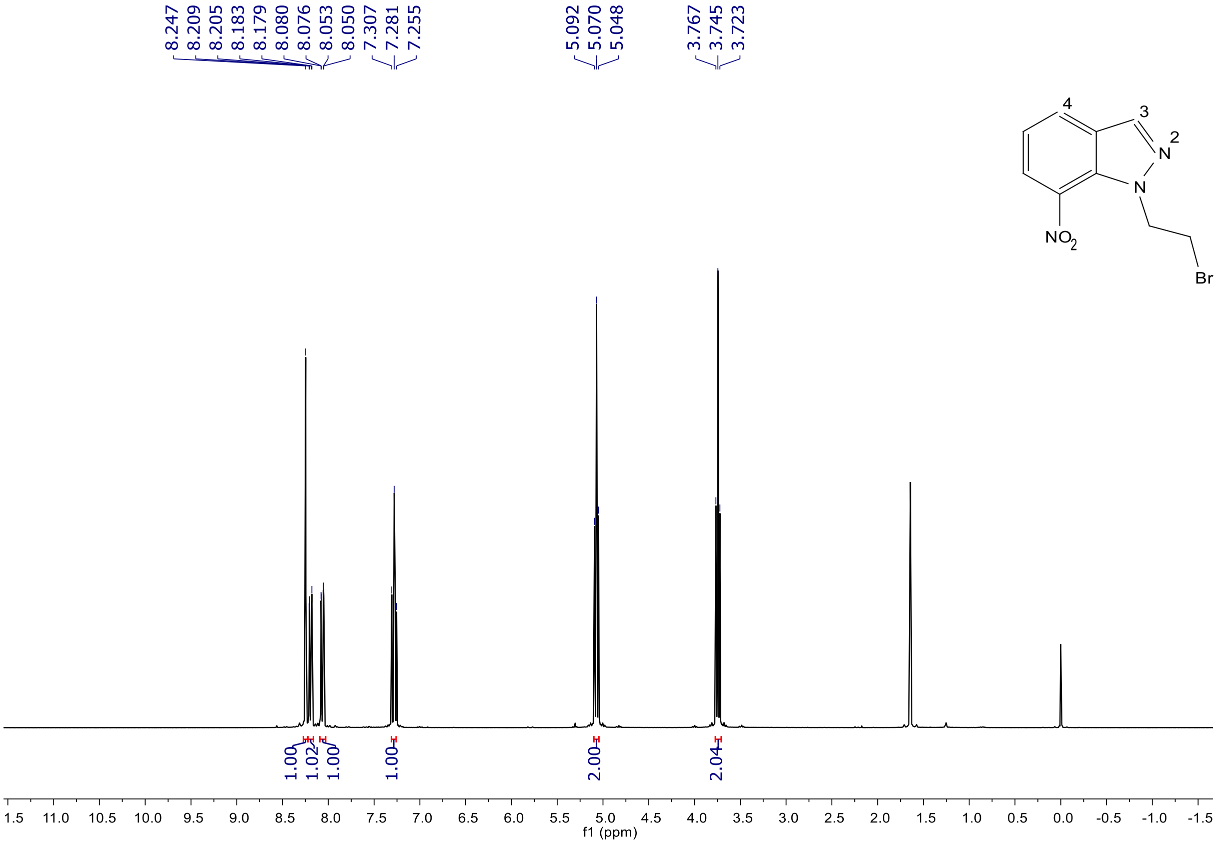

Figure S13. ^{1}H NMR spectrum of compound **2d** in $CDCl_3$.

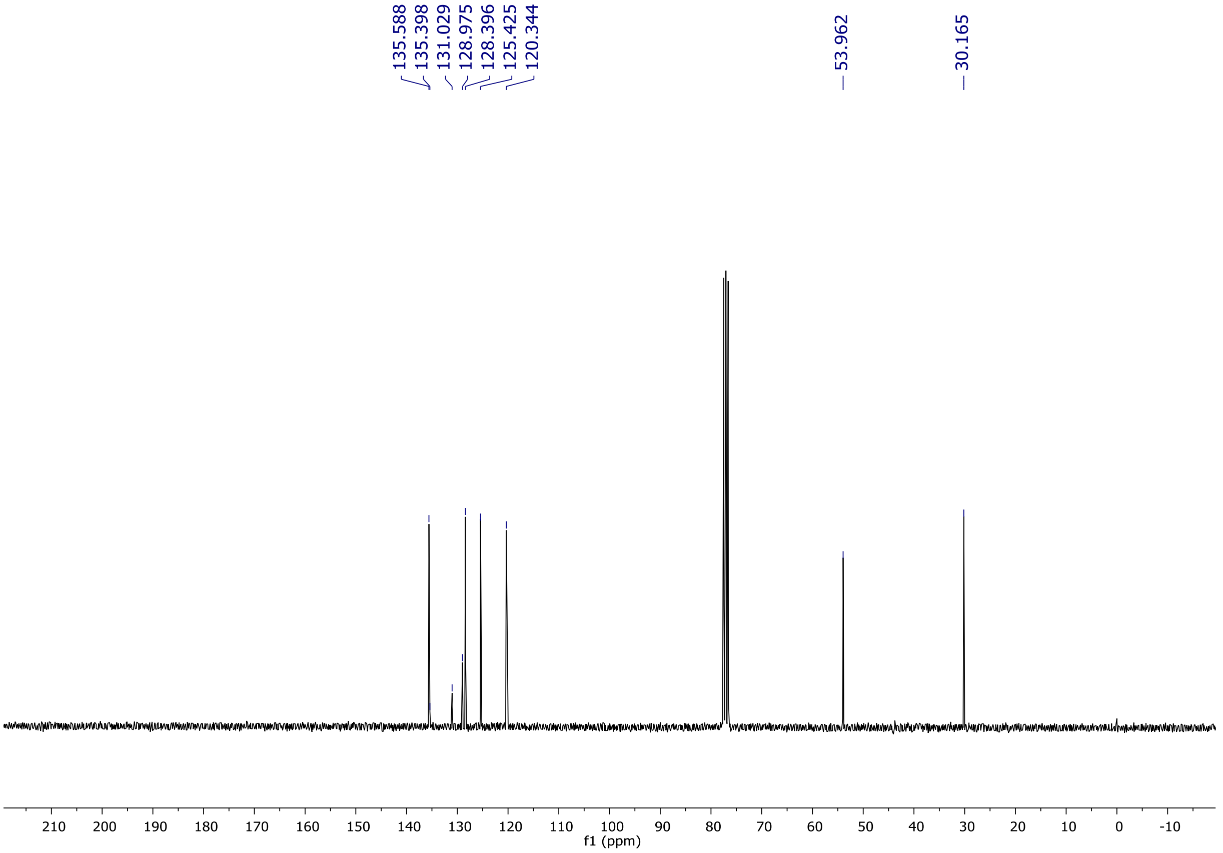

Figure S14. ^{13}C NMR spectrum of compound **2d** in $CDCl_3$.

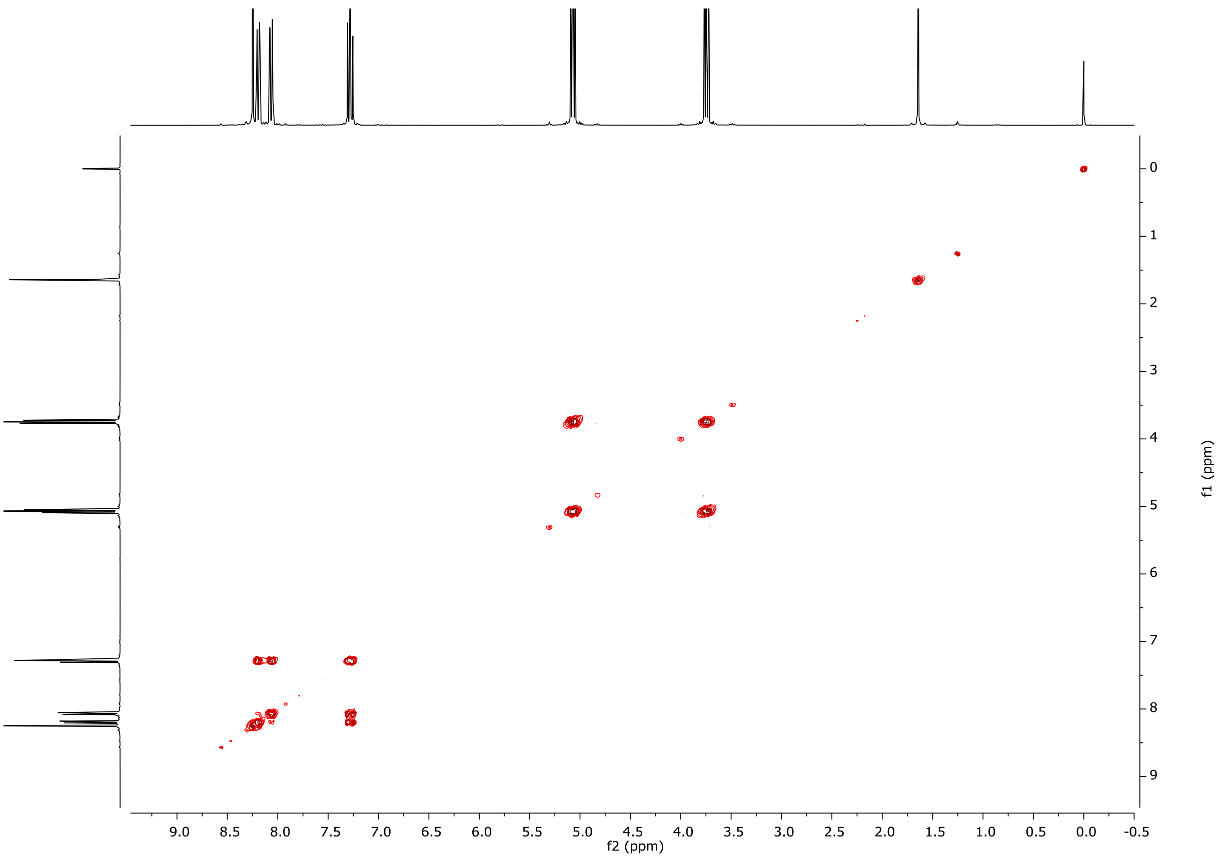

Figure S15. COSY ($^1H/^1H$) spectrum of compound **2d** in $CDCl_3$.

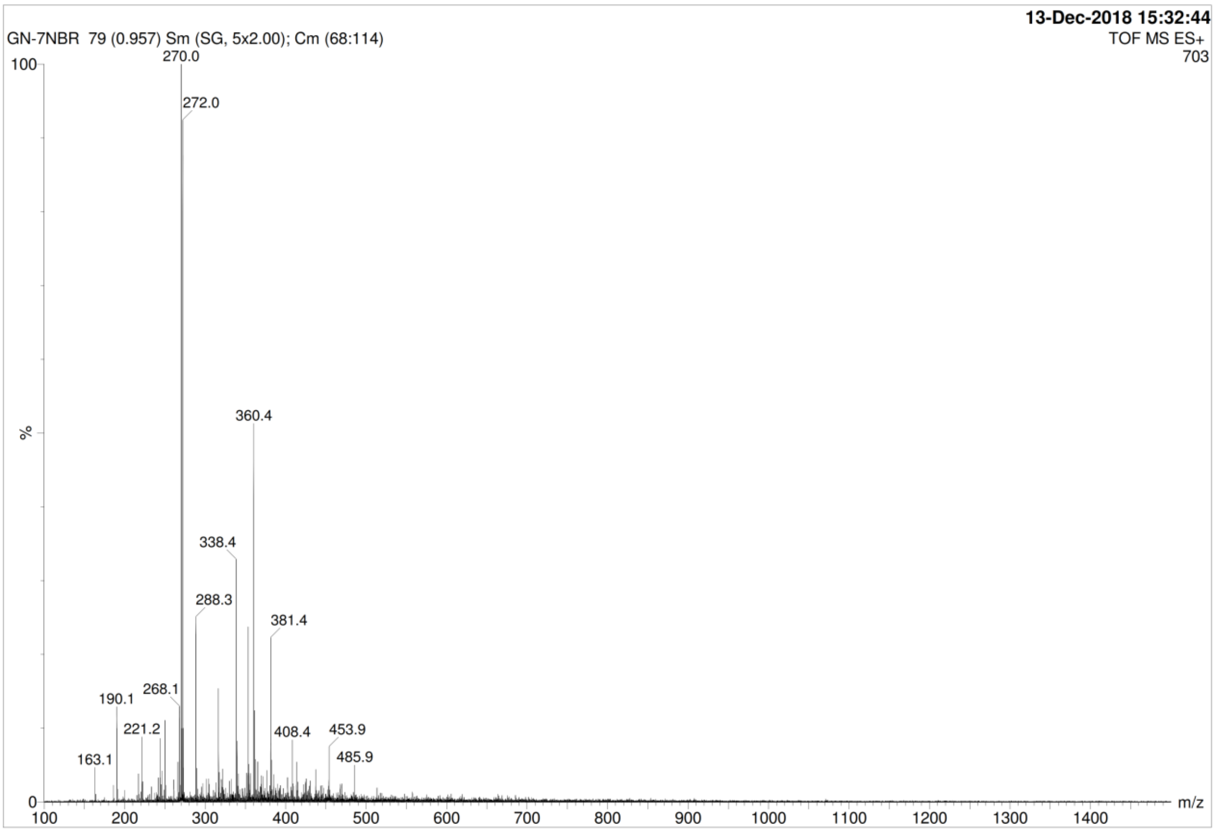

Figure S16. MS-ESI(+) spectrum of compound **2d**.

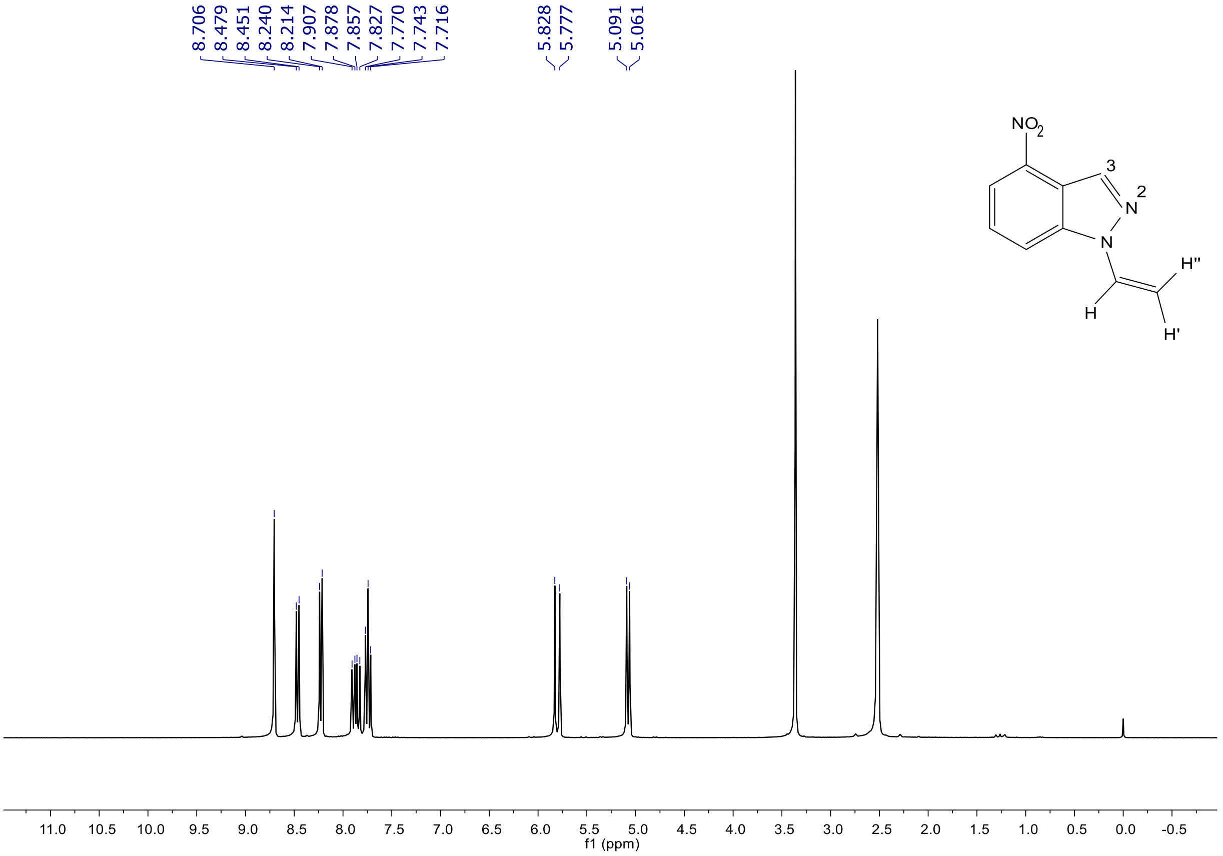

Figure S17. ^{1}H NMR spectrum of compound **3a** in DMSO-d$_6$.

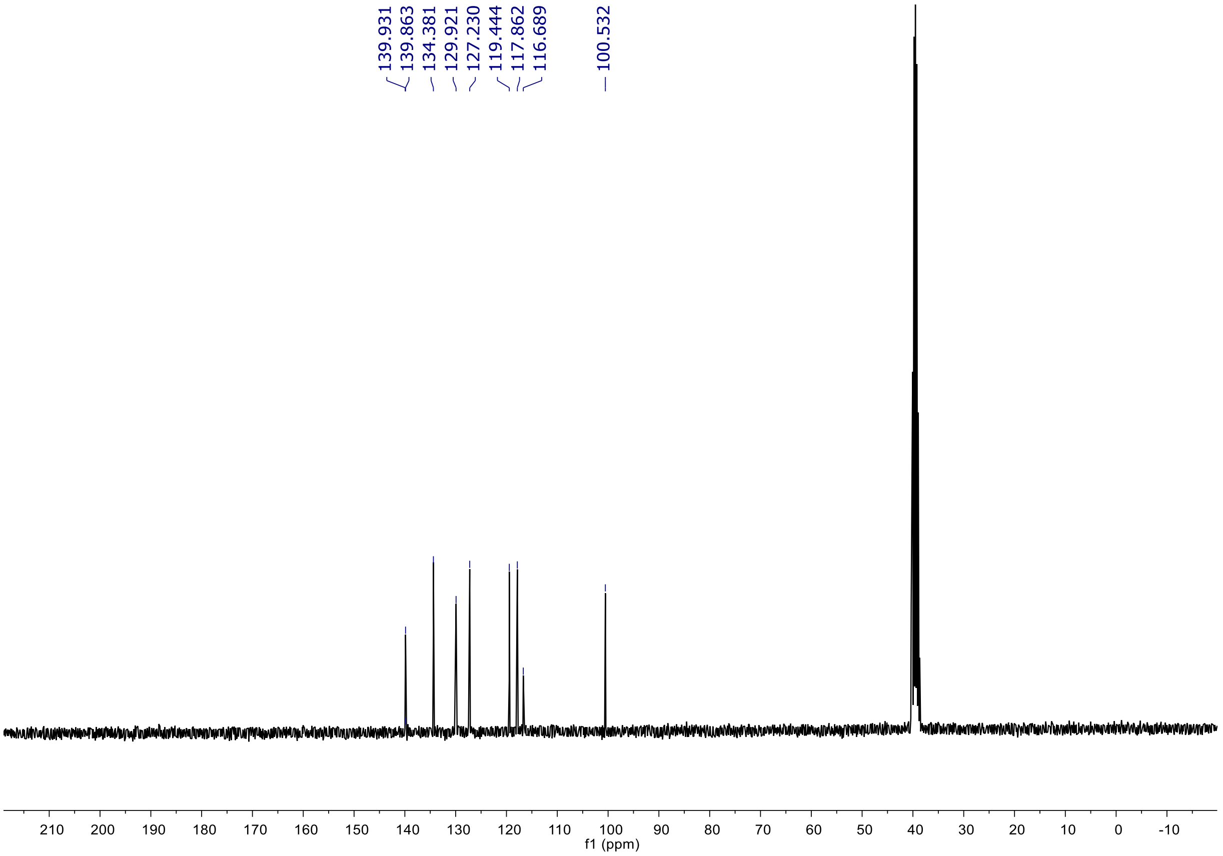

Figure S18. ^{13}C NMR spectrum of compound **3a** in DMSO-d$_6$.

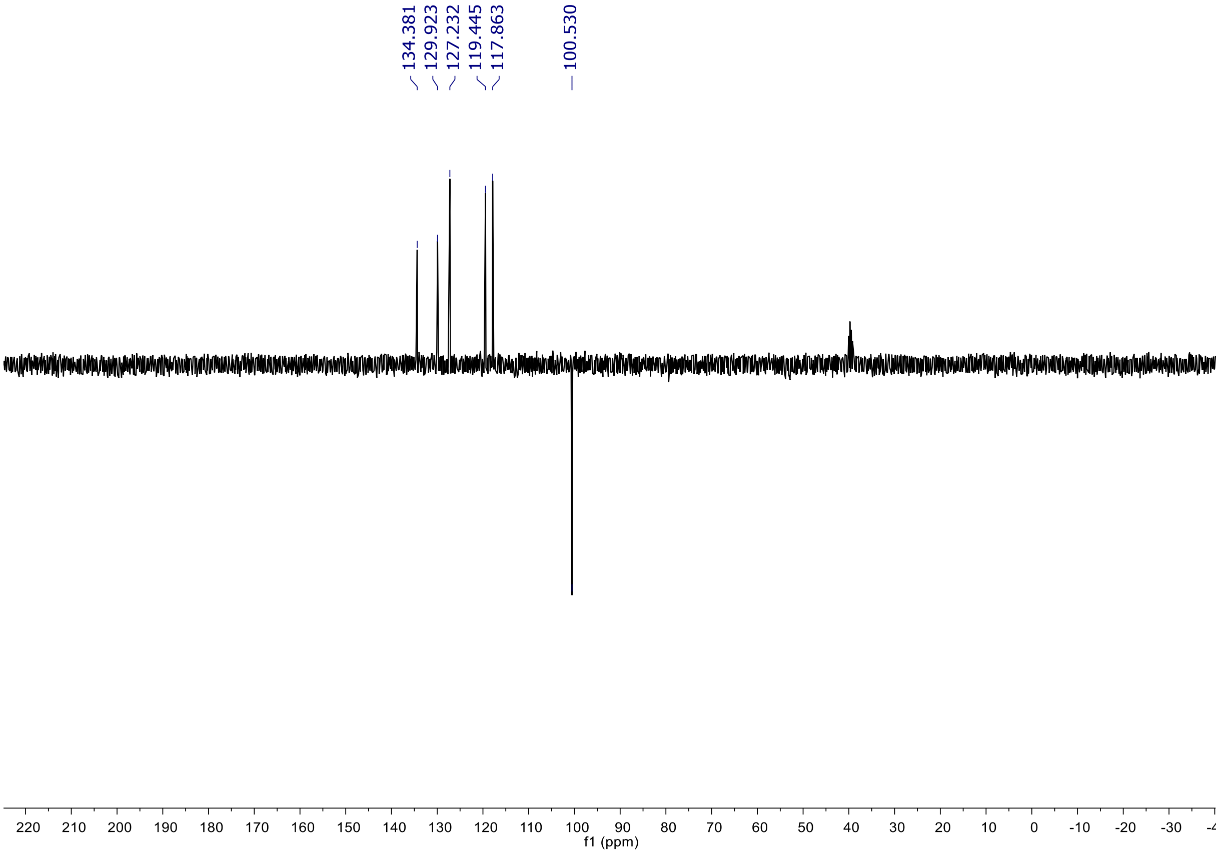

Figure S19. ^{13}C NMR DEPT 135 spectrum of compound **3a** in DMSO-d_6.

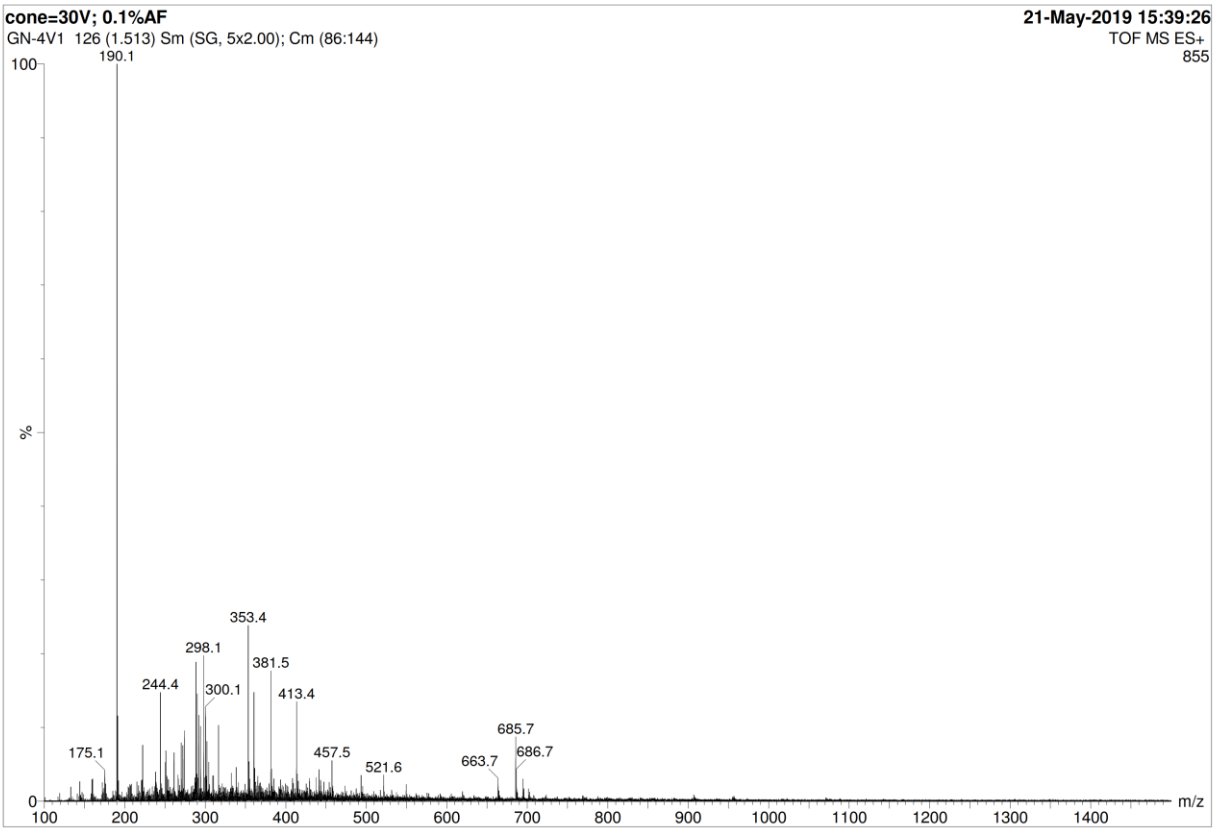

Figure S20. MS-ESI(+) spectrum of compound **3a**.

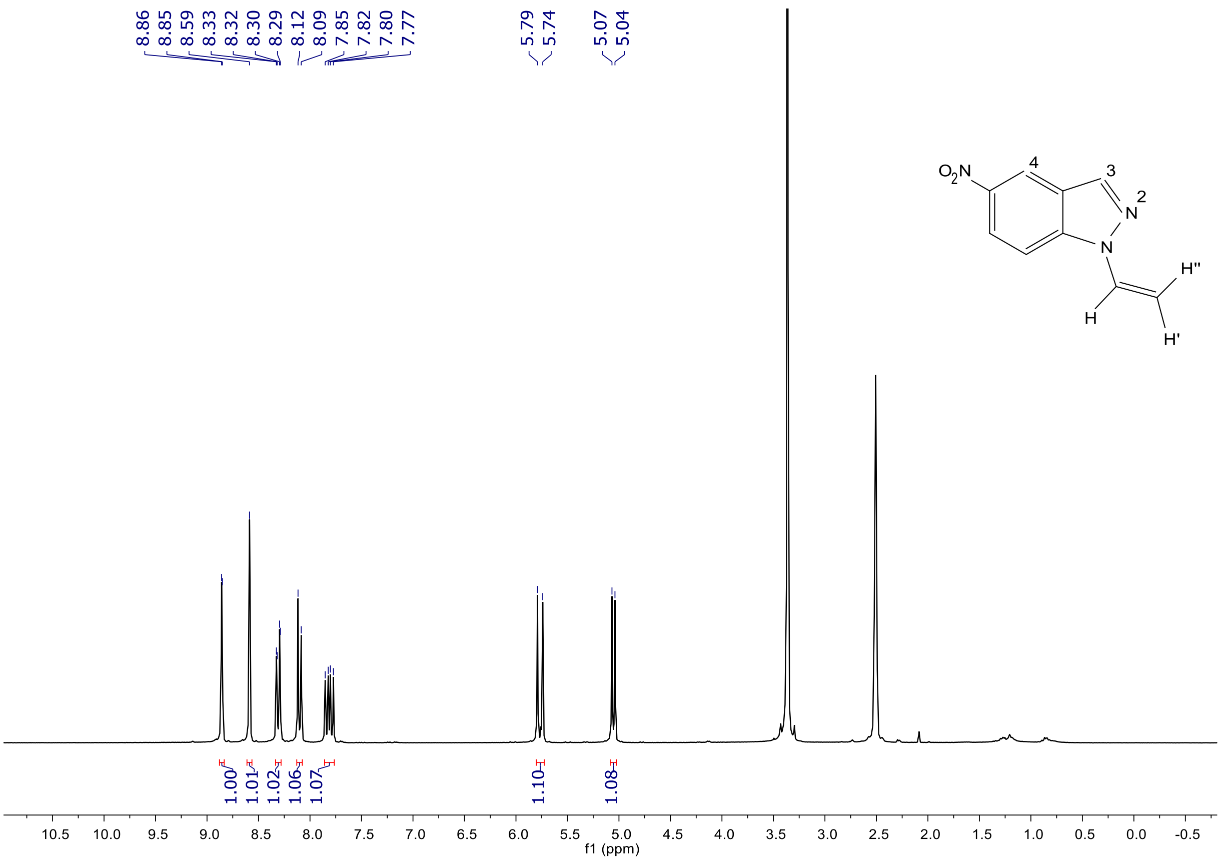

Figure S21. ^{1}H NMR spectrum of compound **3b** in DMSO-d_6.

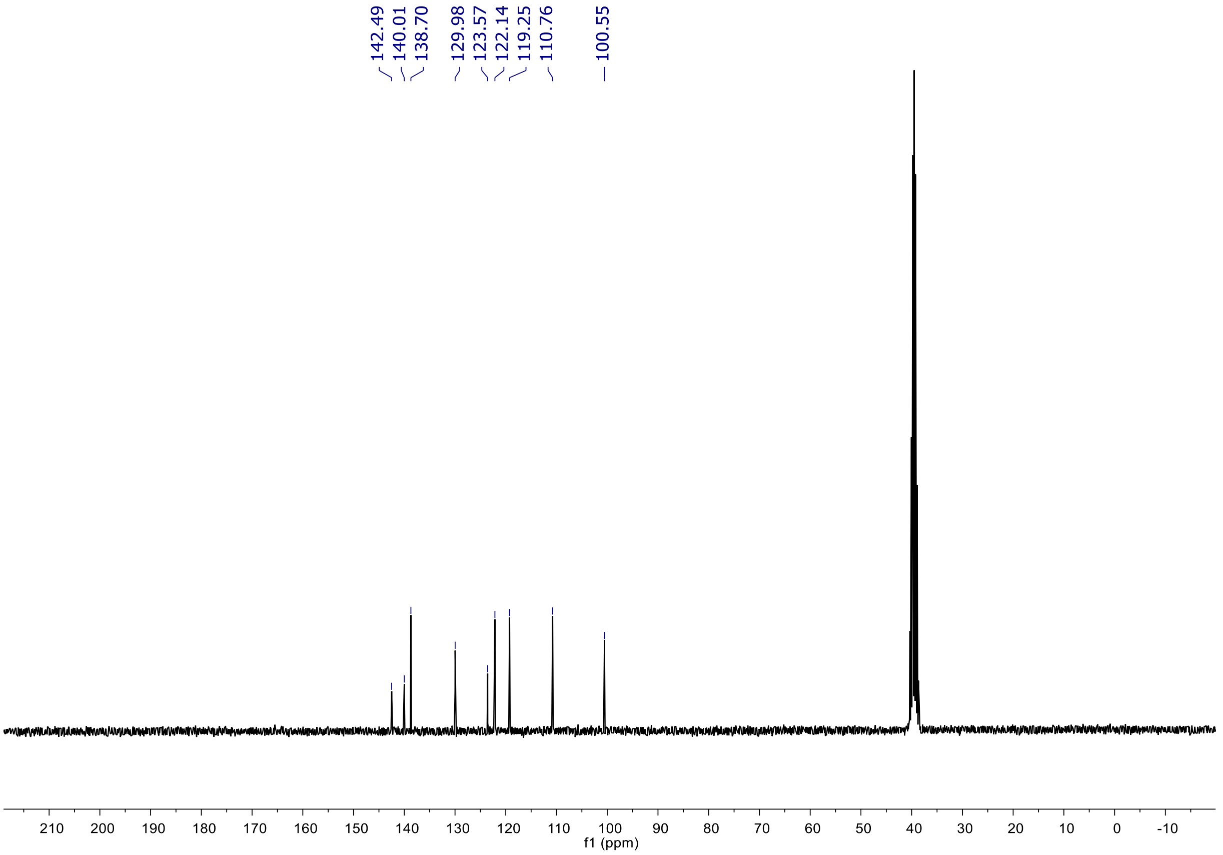

Figure S22. ^{13}C NMR spectrum of compound **3b** in DMSO-d_6.

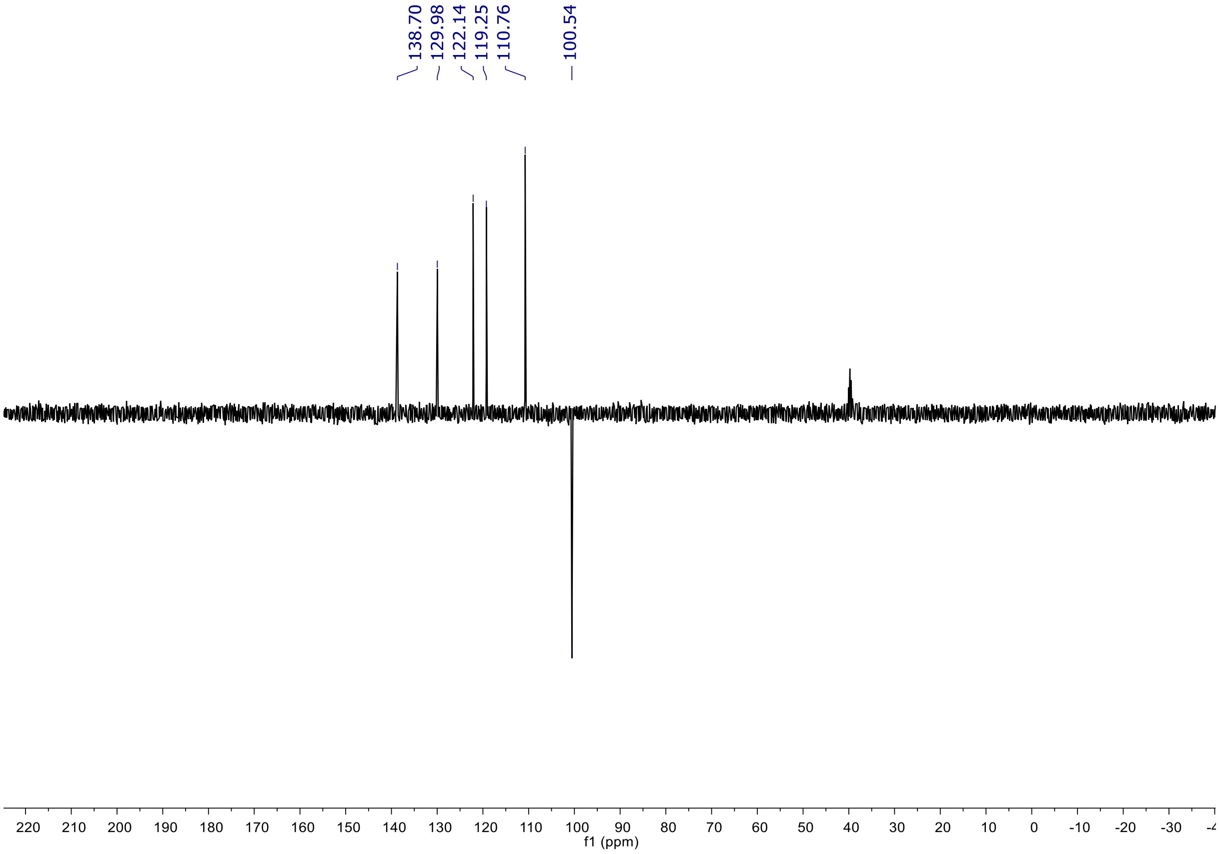

Figure S23. ^{13}C NMR DEPT 135 spectrum of compound **3b** in DMSO-d_6.

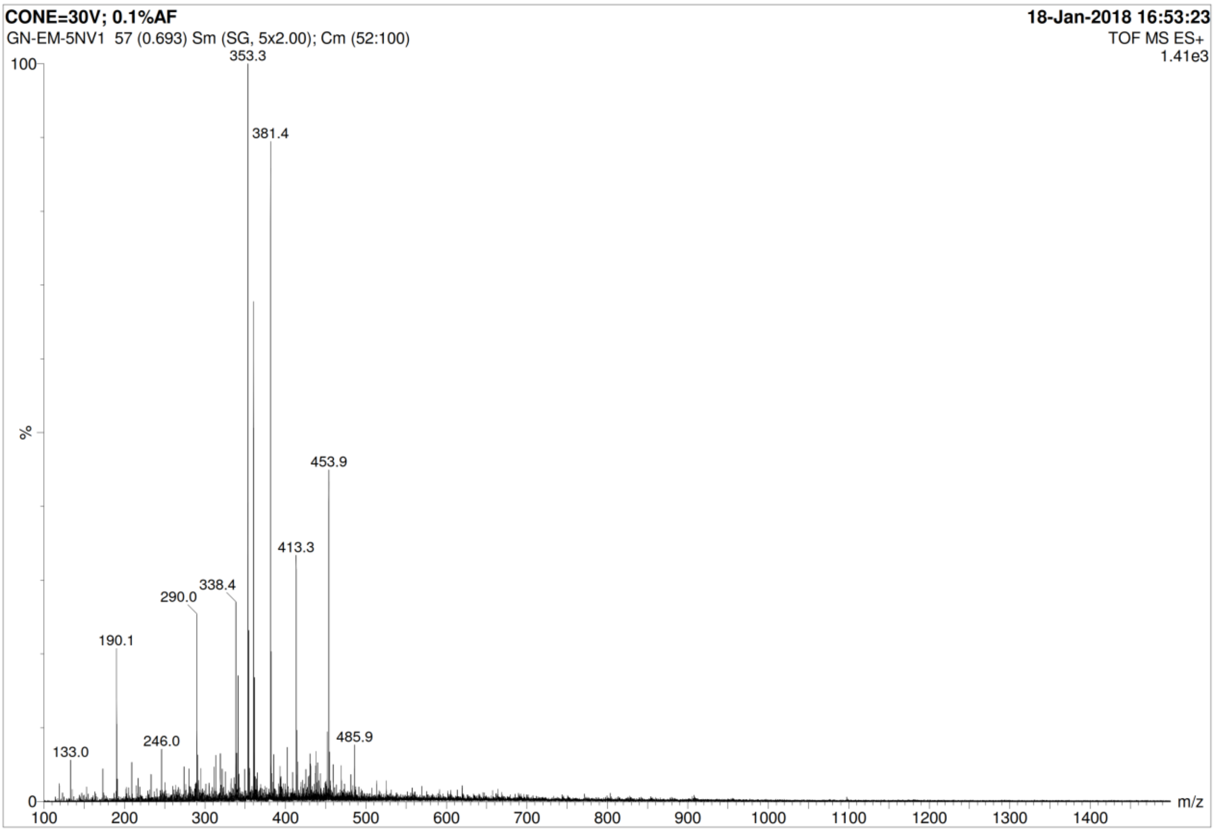

Figure S24. MS-ESI(+) spectrum of compound **3b**.

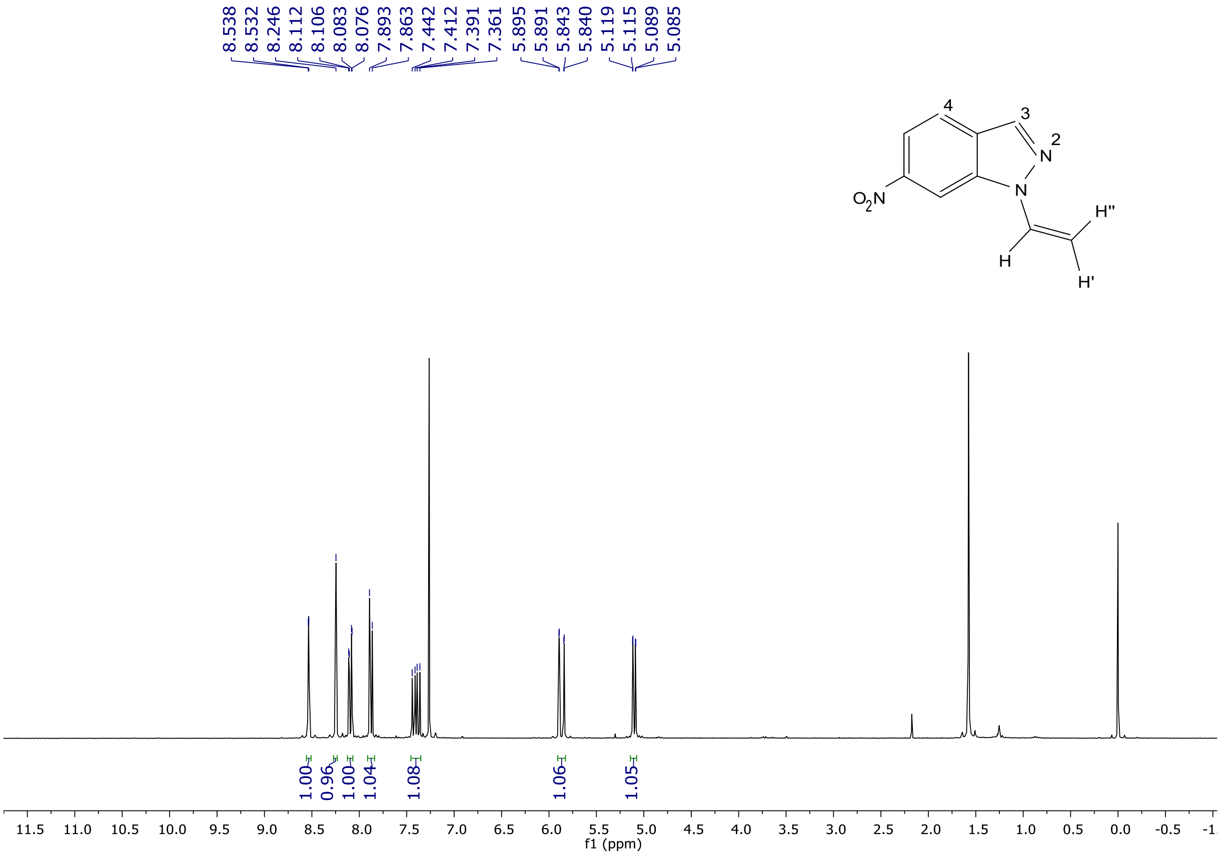

Figure S25. ^{1}H NMR spectrum of compound **3c** in $CDCl_3$.

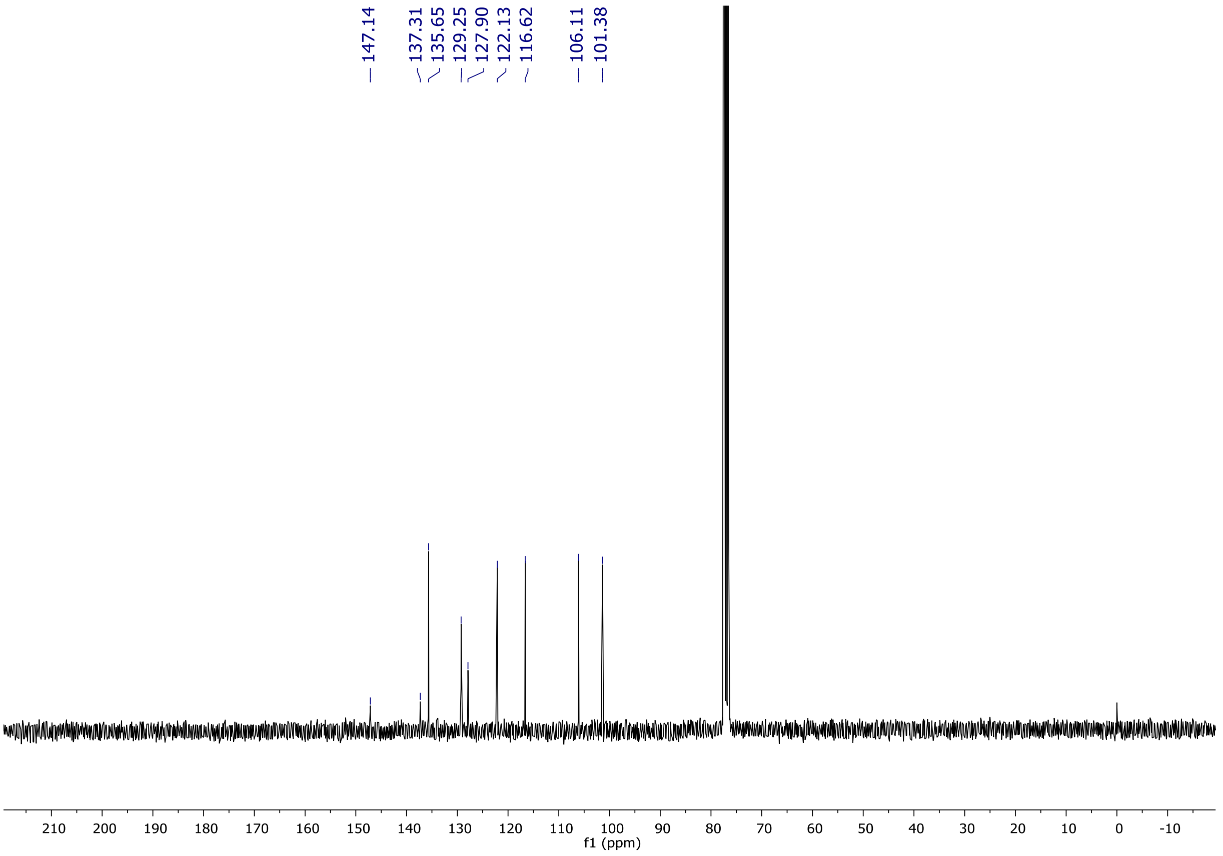

Figure S26. ^{13}C NMR spectrum of compound **3c** in $CDCl_3$.

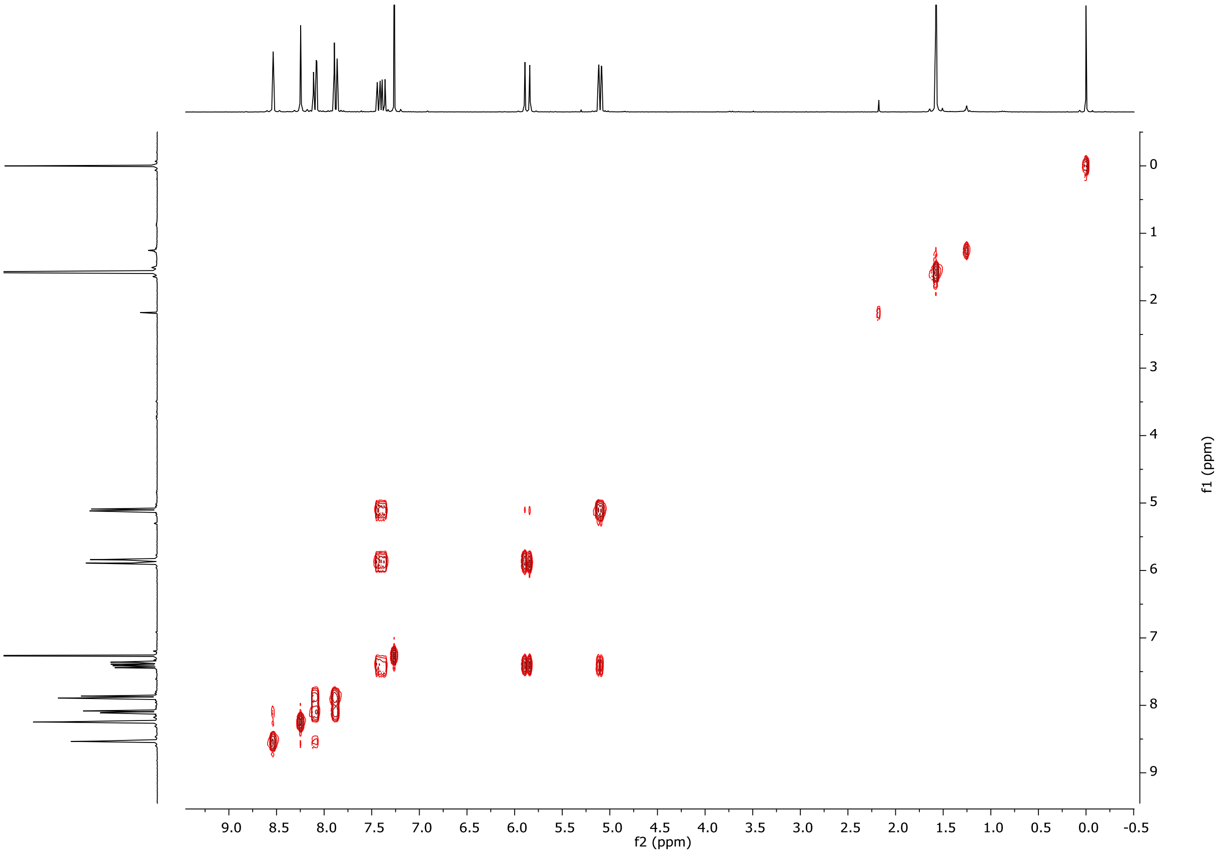

Figure S27. COSY ($^1H/^1H$) spectrum of compound **3c** in $CDCl_3$.

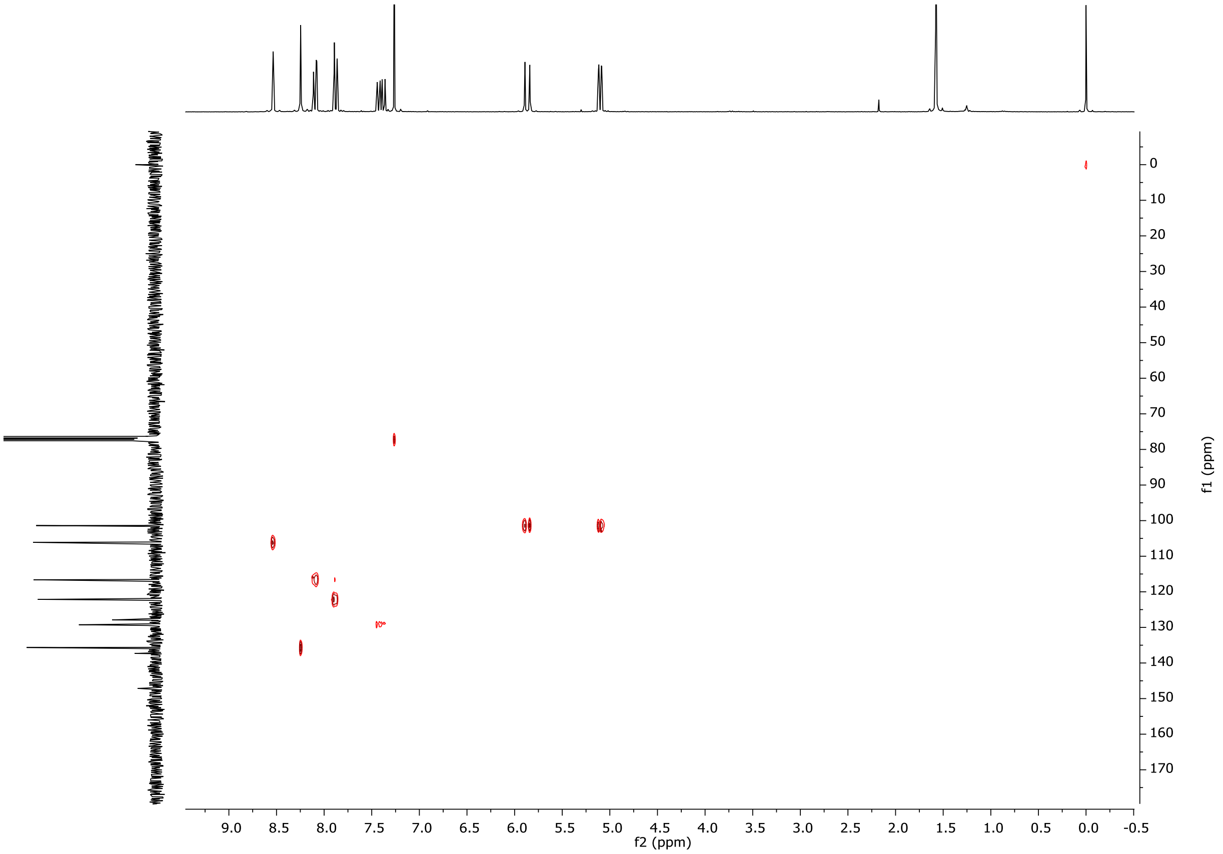

Figure S28. HSQC ($^1H/^{13}C$) spectrum of compound **3c** in $CDCl_3$.

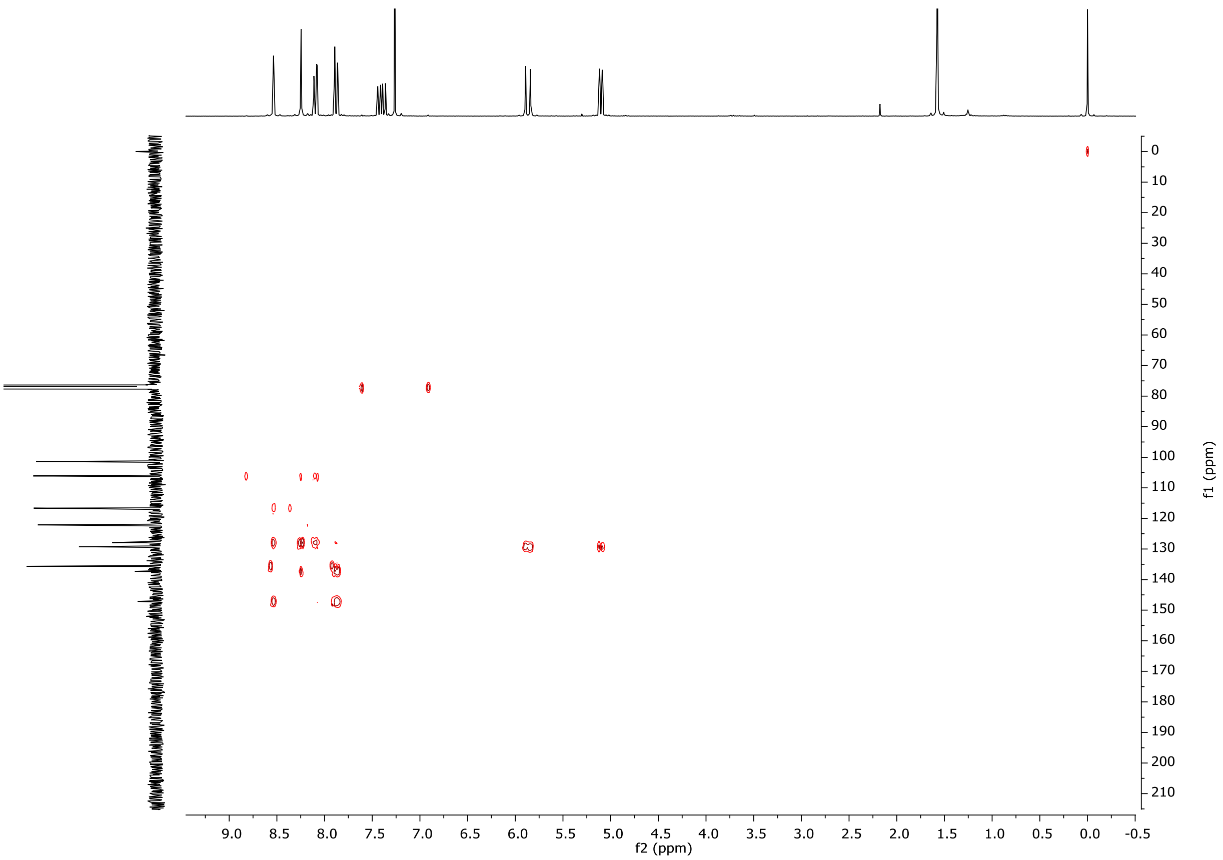

Figure S29. HMBC ($^{1}H/^{13}C$) spectrum of compound **3c** in $CDCl_3$.

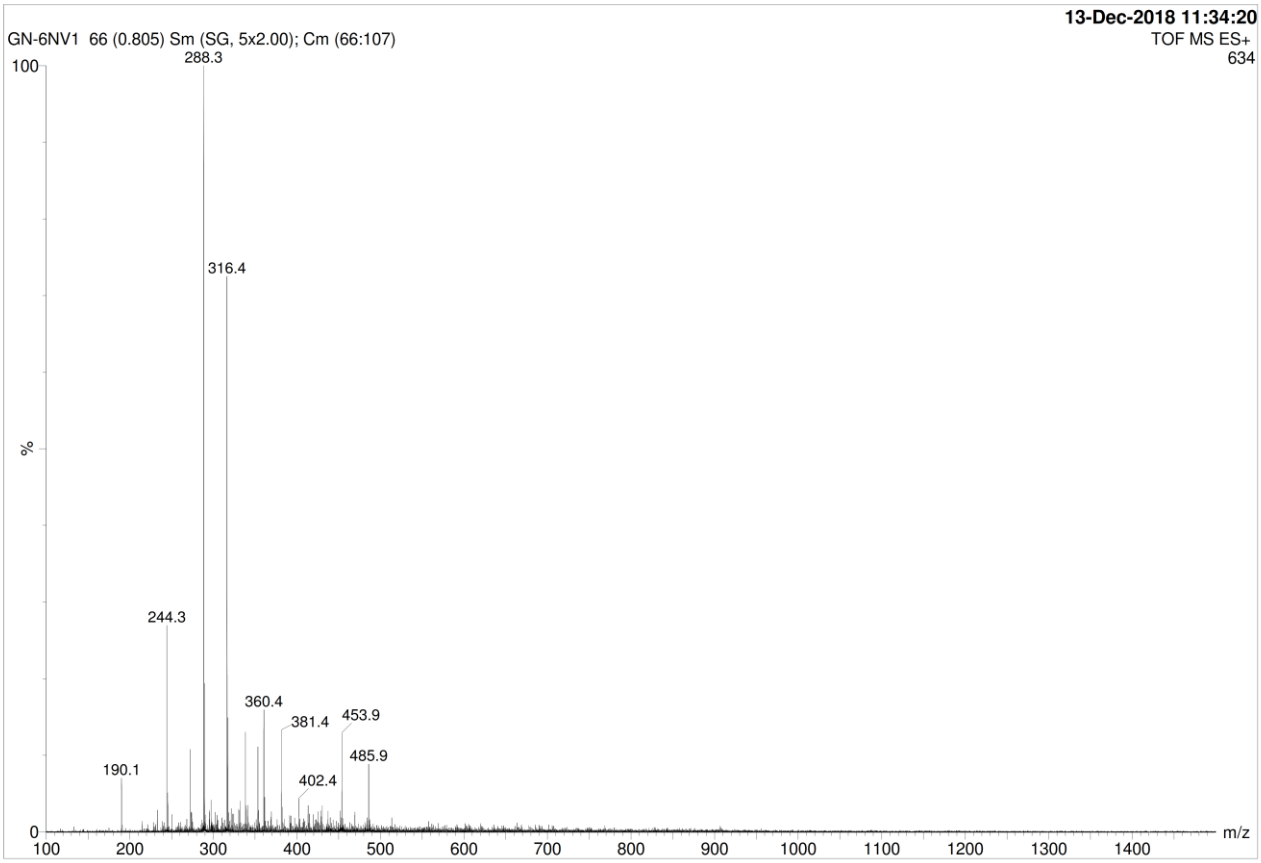

Figure S30. MS-ESI(+) spectrum of compound **3c**.

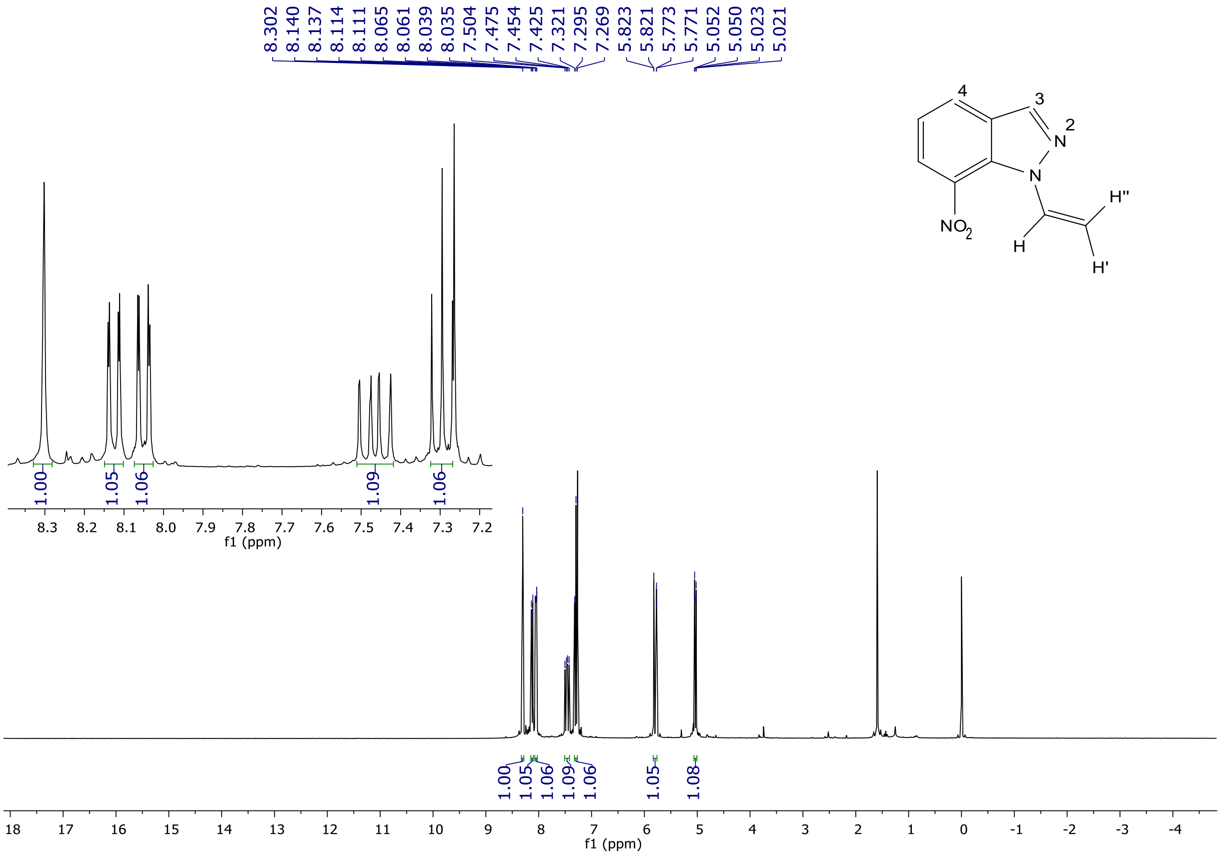

Figure S31. ^{1}H NMR spectrum of compound **3d** in $CDCl_3$.

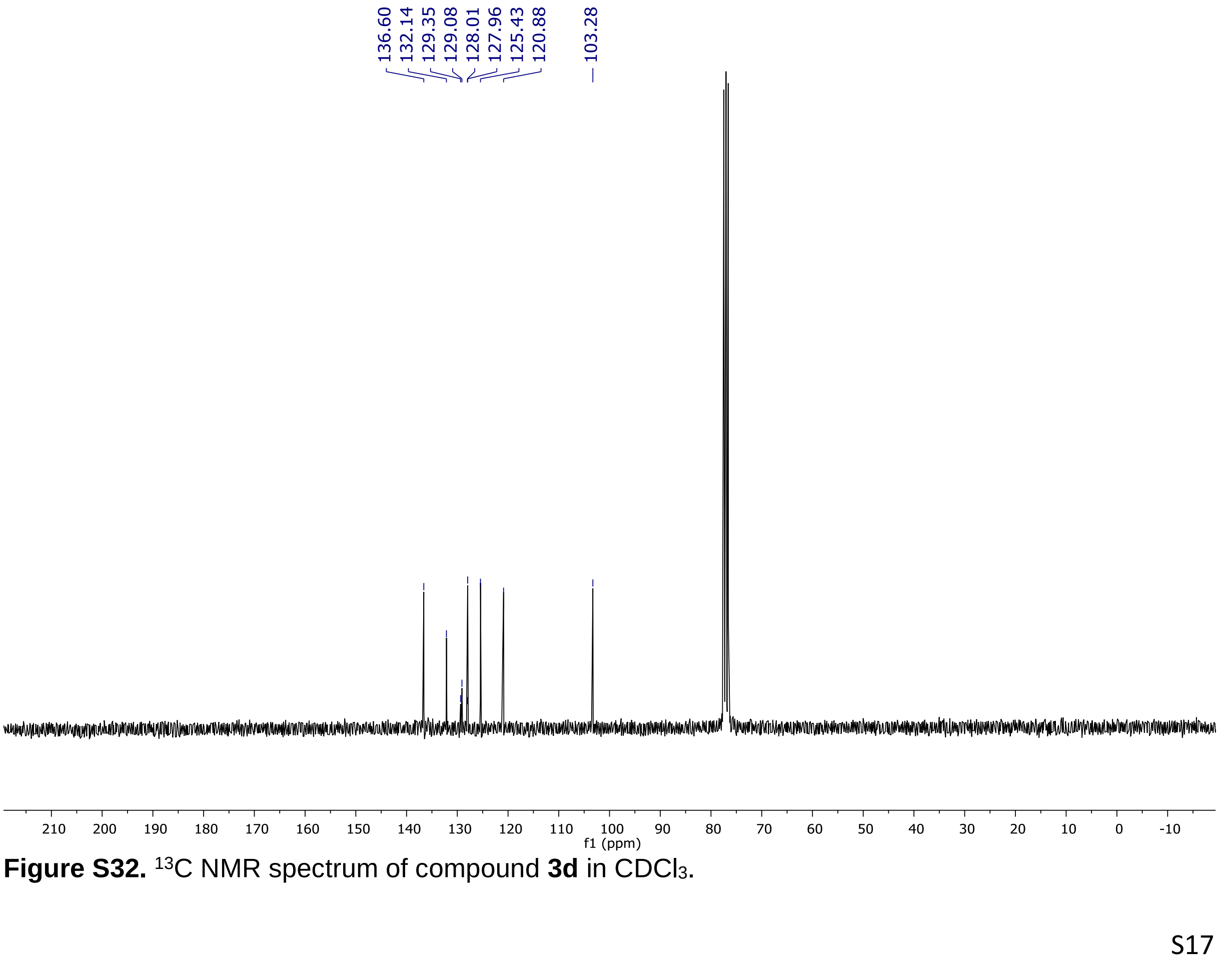

Figure S32. ^{13}C NMR spectrum of compound **3d** in $CDCl_3$.

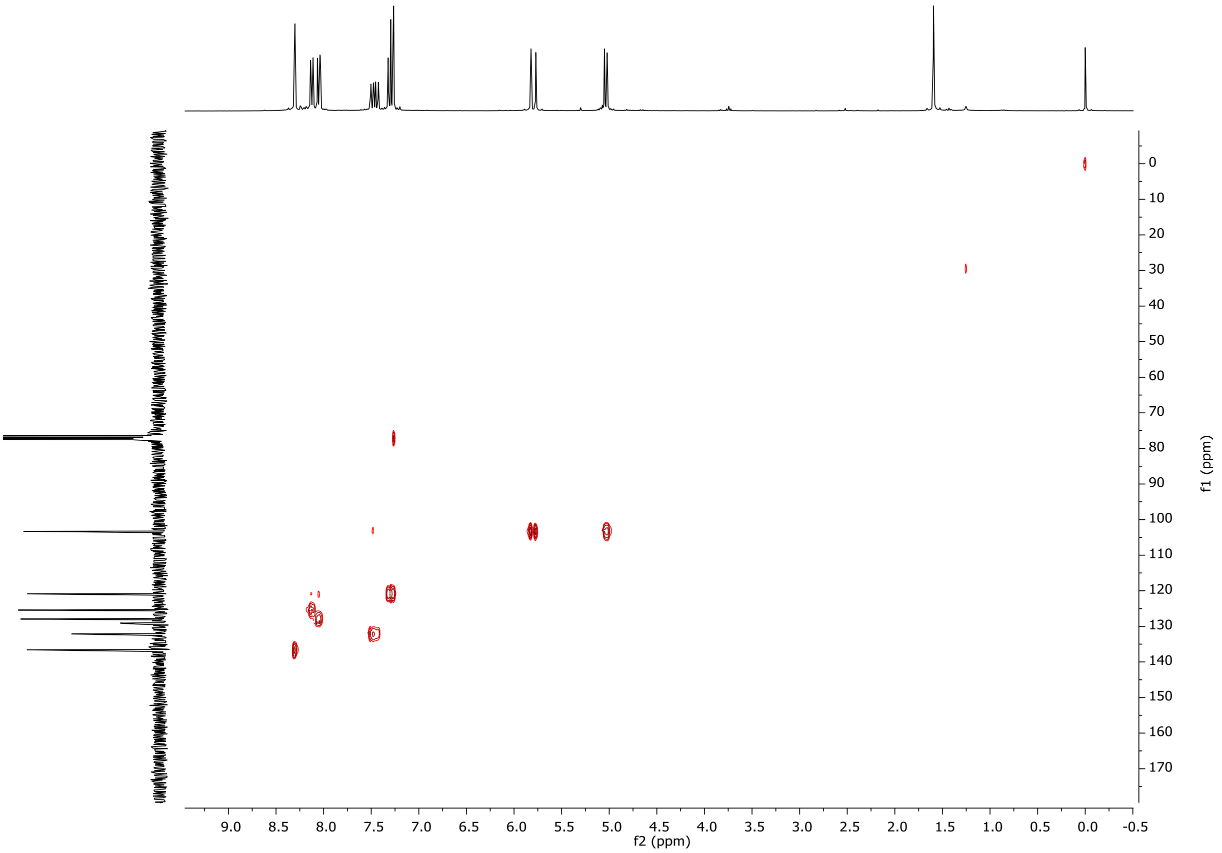

Figure S33. HSQC ($^{1}H/^{13}C$) spectrum of compound **3d** in $CDCl_3$.

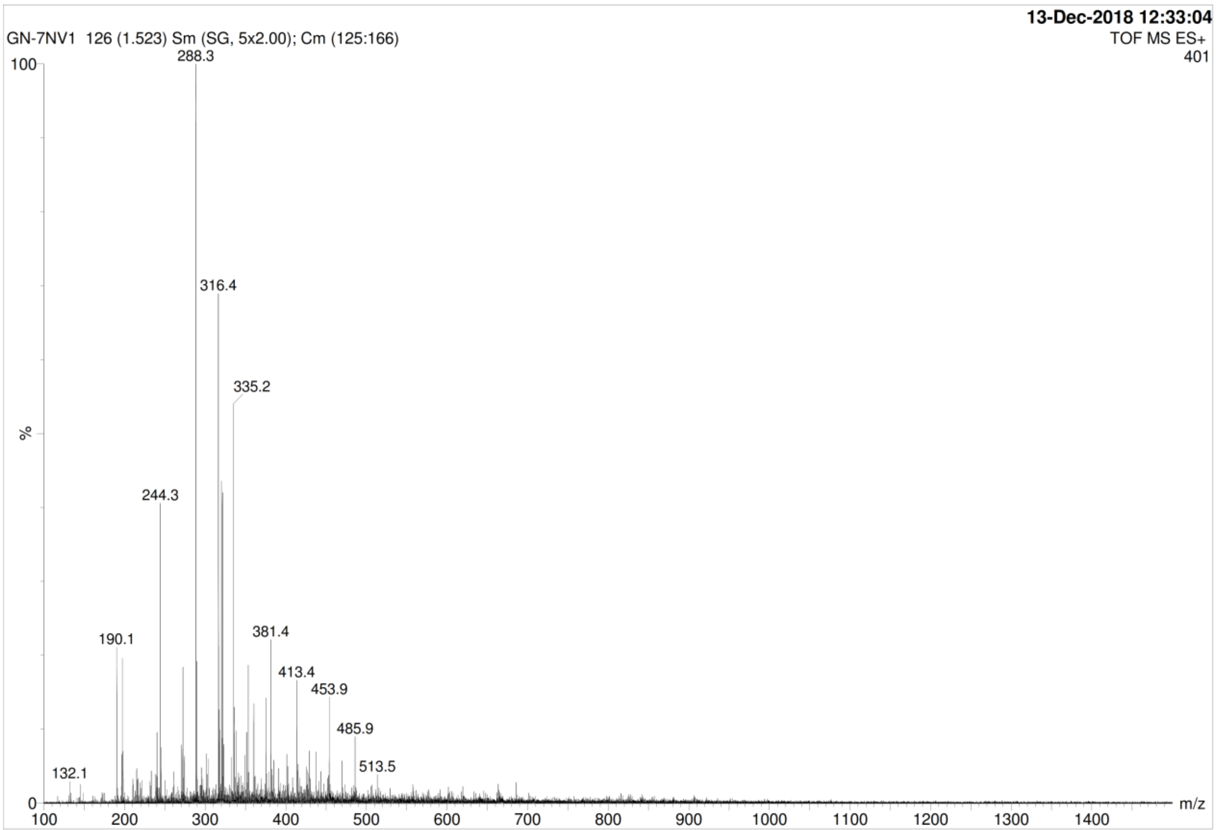

Figure S34. MS-ESI(+) spectrum of compound **3d**.

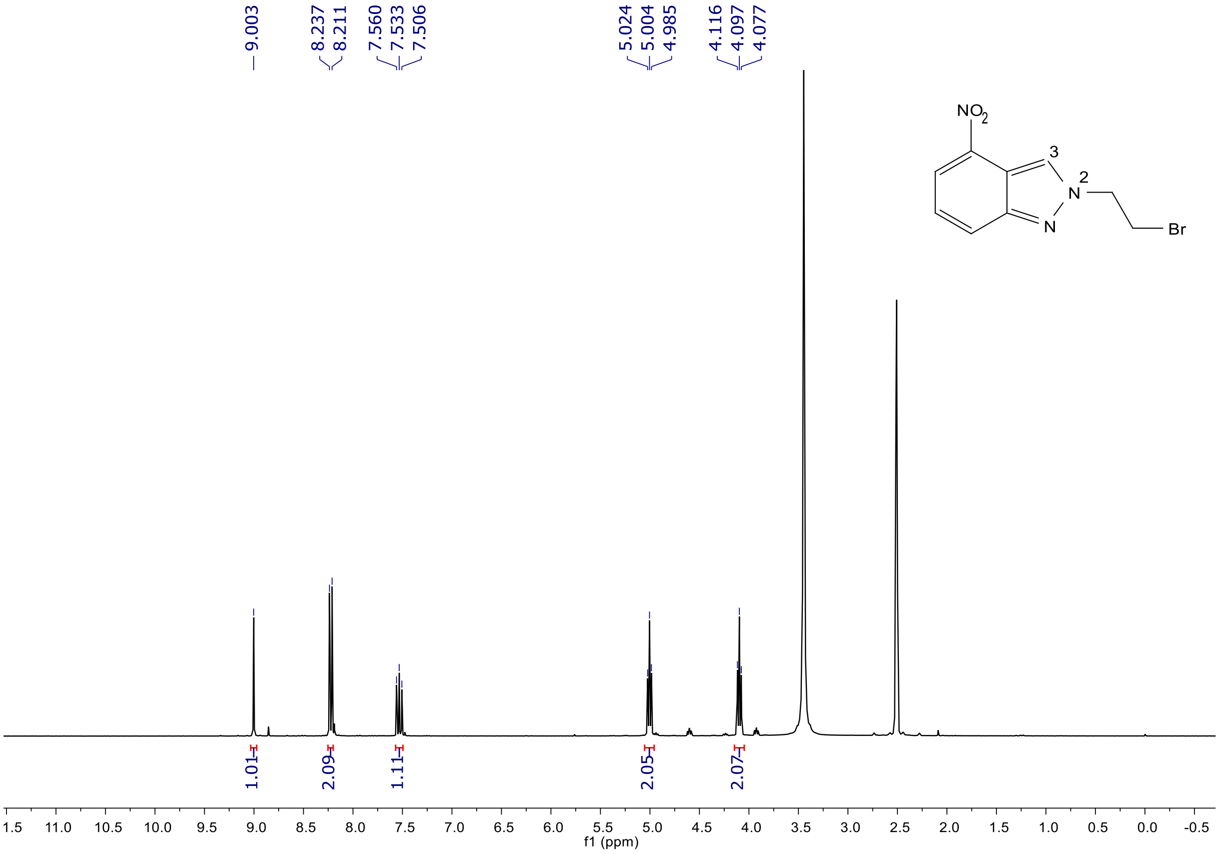

Figure S35. 1H NMR spectrum of compound **4a** in DMSO-d_6.

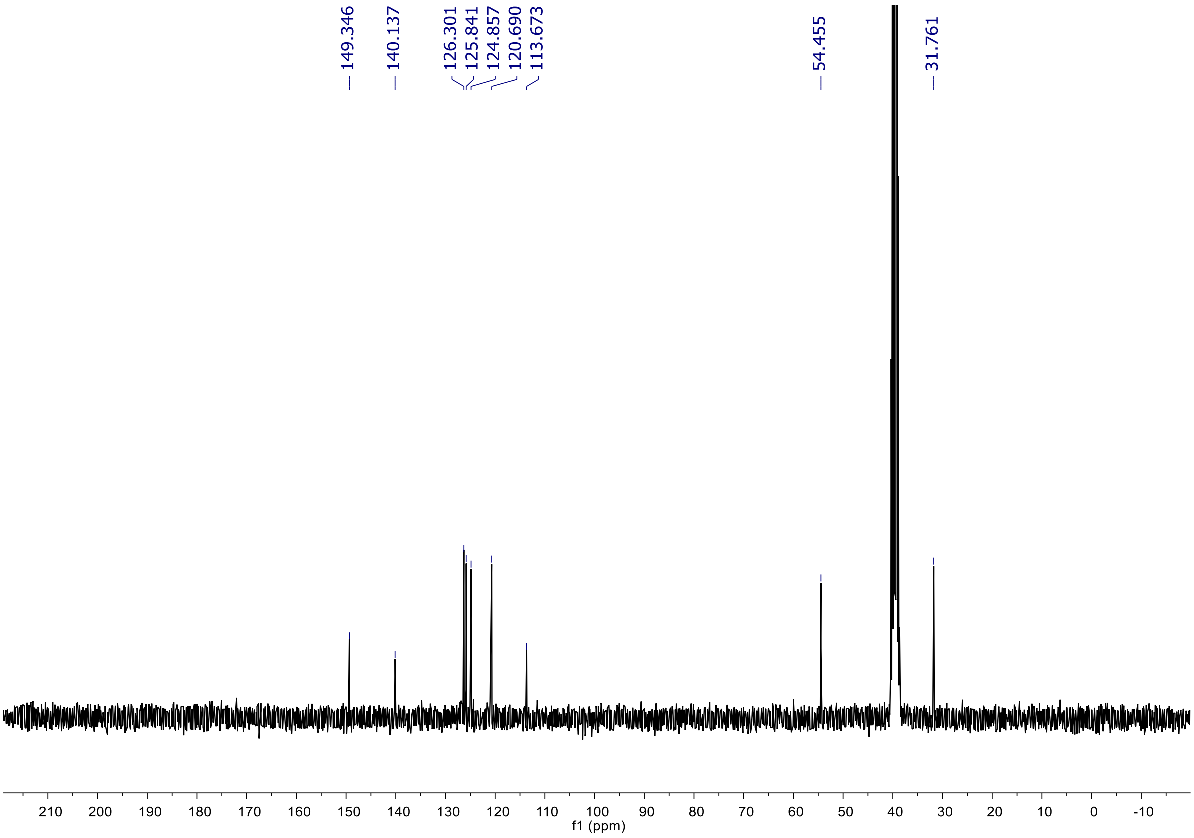

Figure S36. ^{13}C NMR spectrum of compound **4a** in DMSO-d_6.

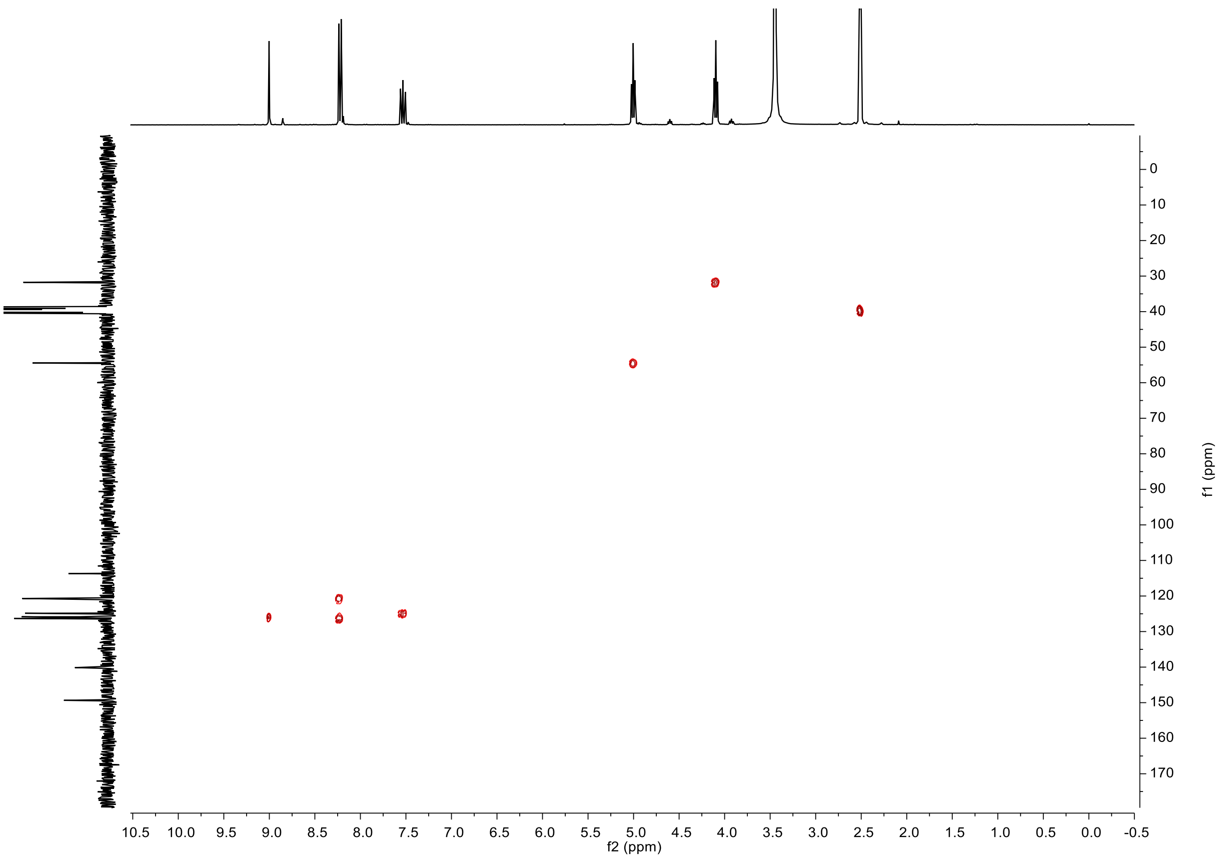

Figure S37. HSQC ($^{1}H/^{13}C$) spectrum of compound **4a** in DMSO-d_6.

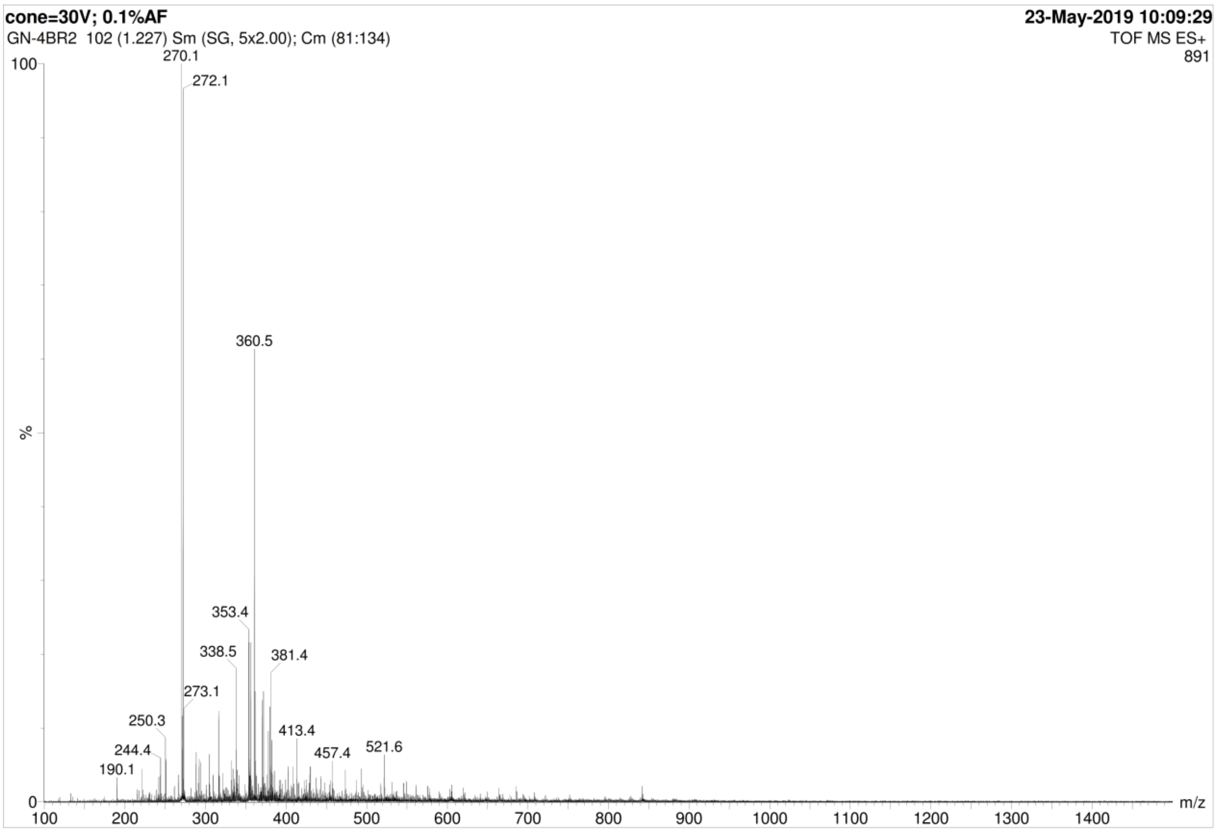

Figure S38. MS-ESI(+) spectrum of compound **4a**.

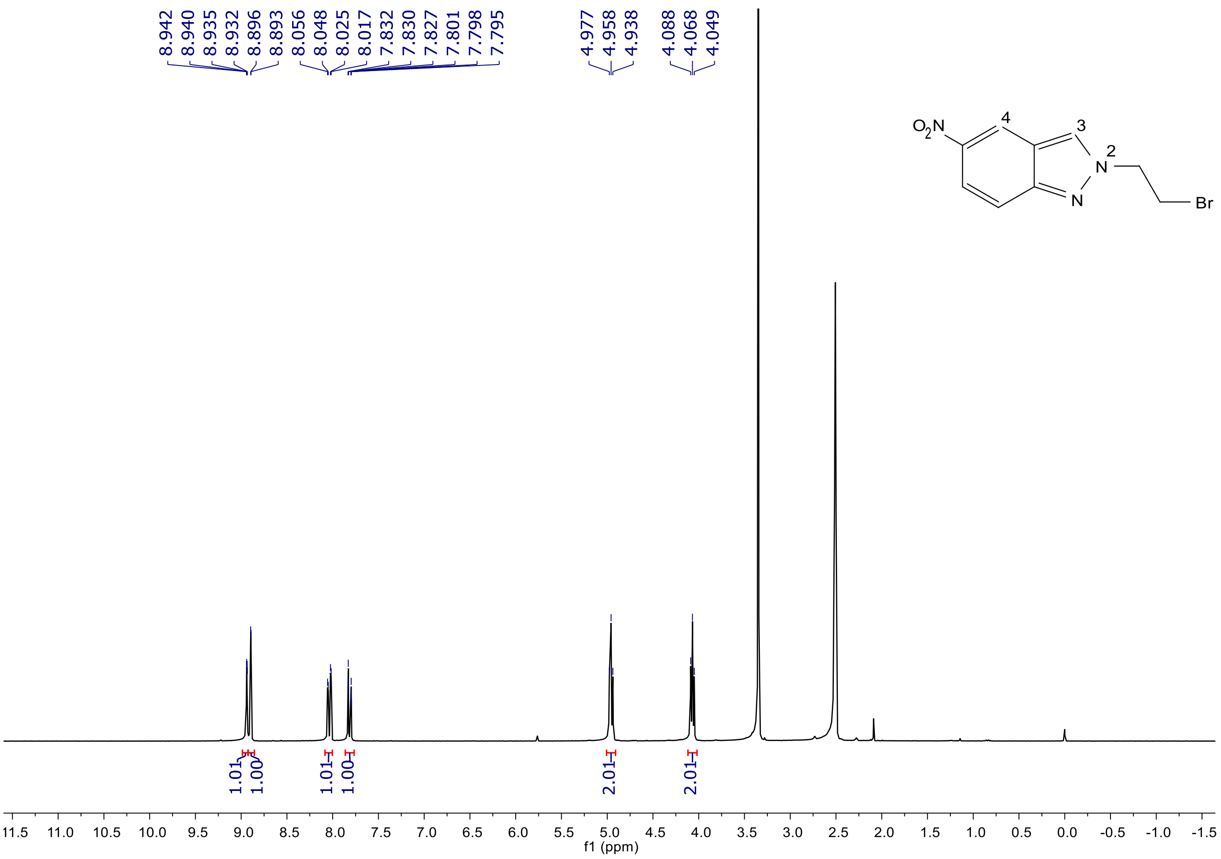

Figure S39. ^{1}H NMR spectrum of compound **4b** in DMSO-d6.

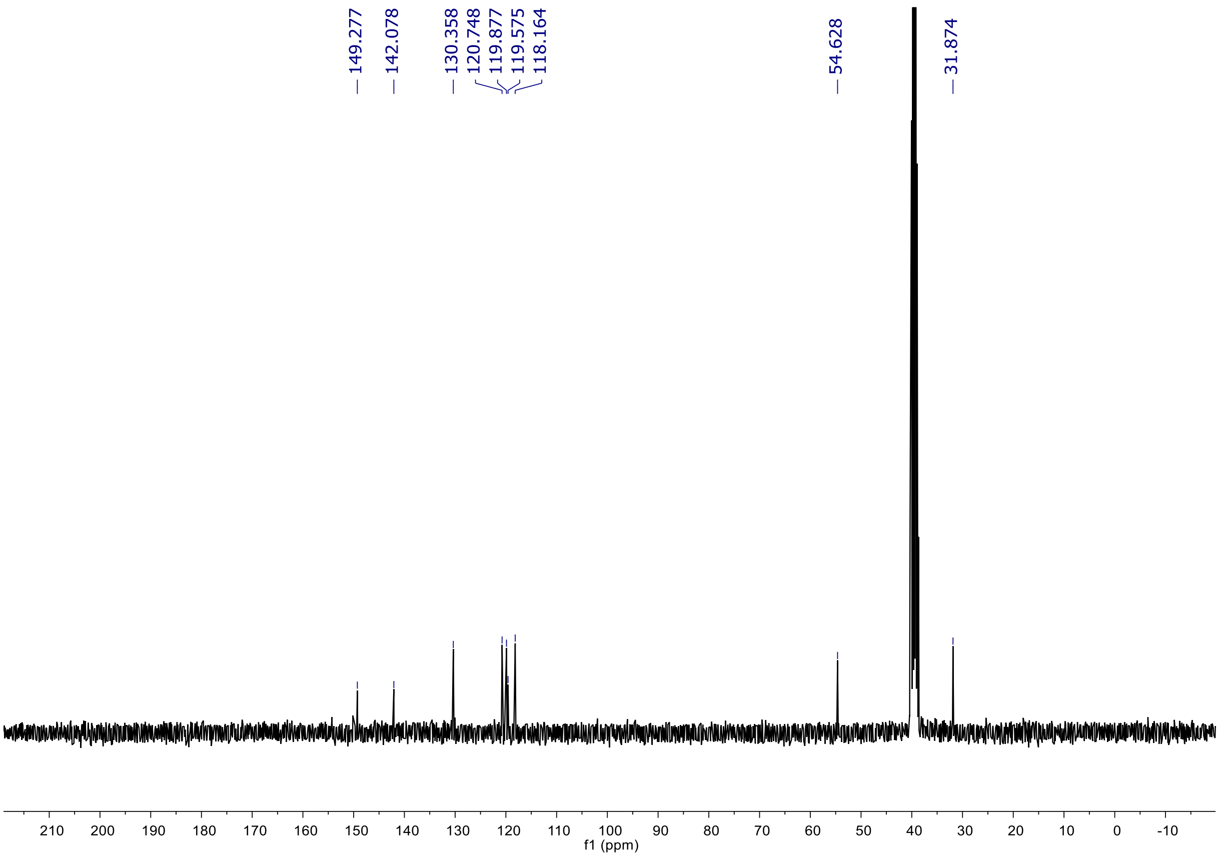

Figure S40. ^{13}C NMR spectrum of compound **4b** in DMSO-d6.

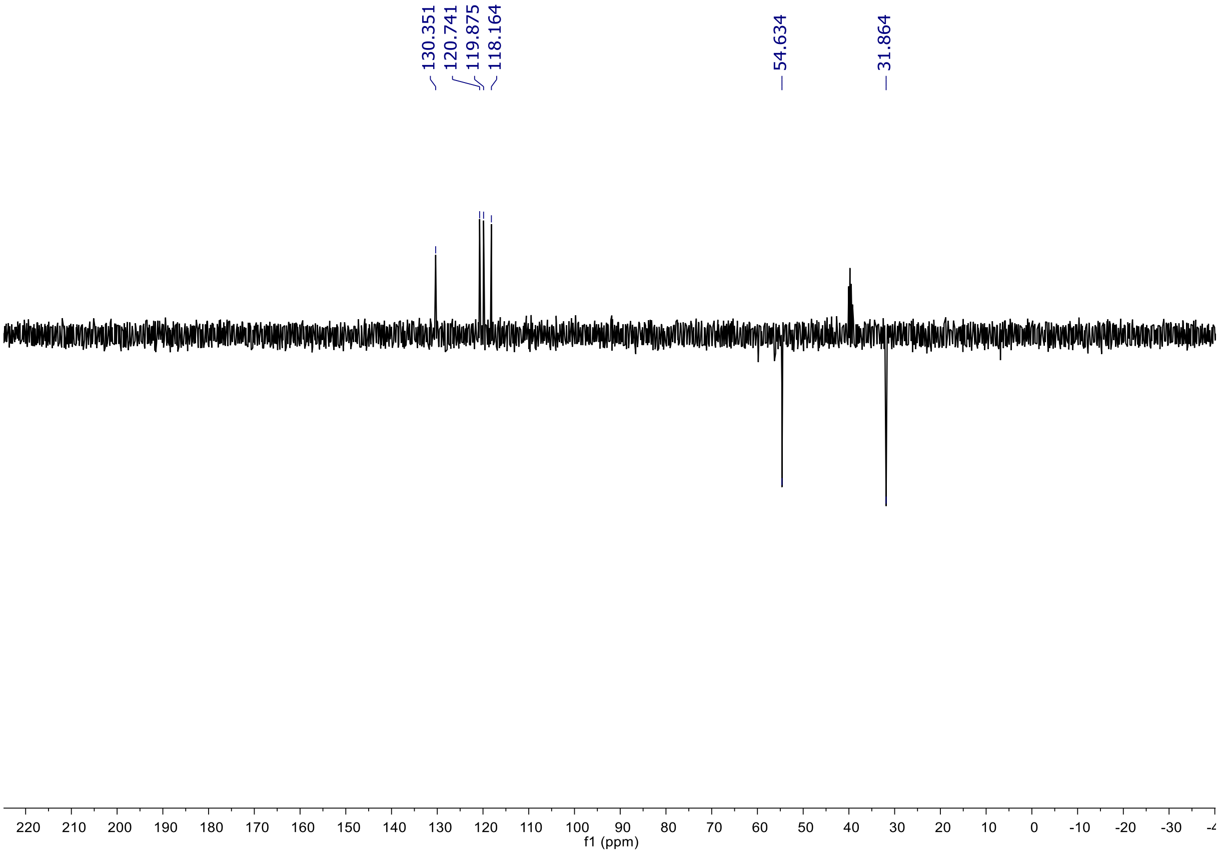

Figure S41. ^{13}C NMR DEPT 135 spectrum of compound **4b** in DMSO-d_6.

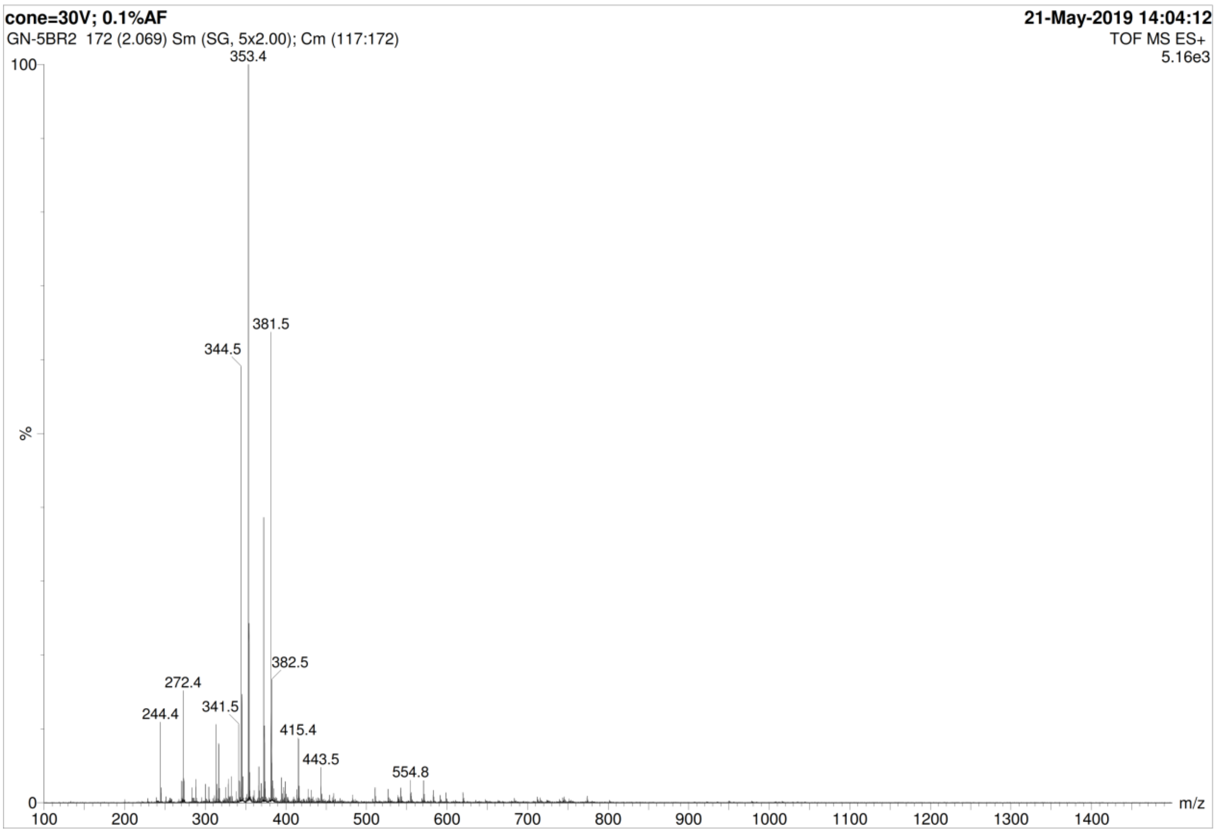

Figure S42. MS-ESI(+) spectrum of compound **4b.**

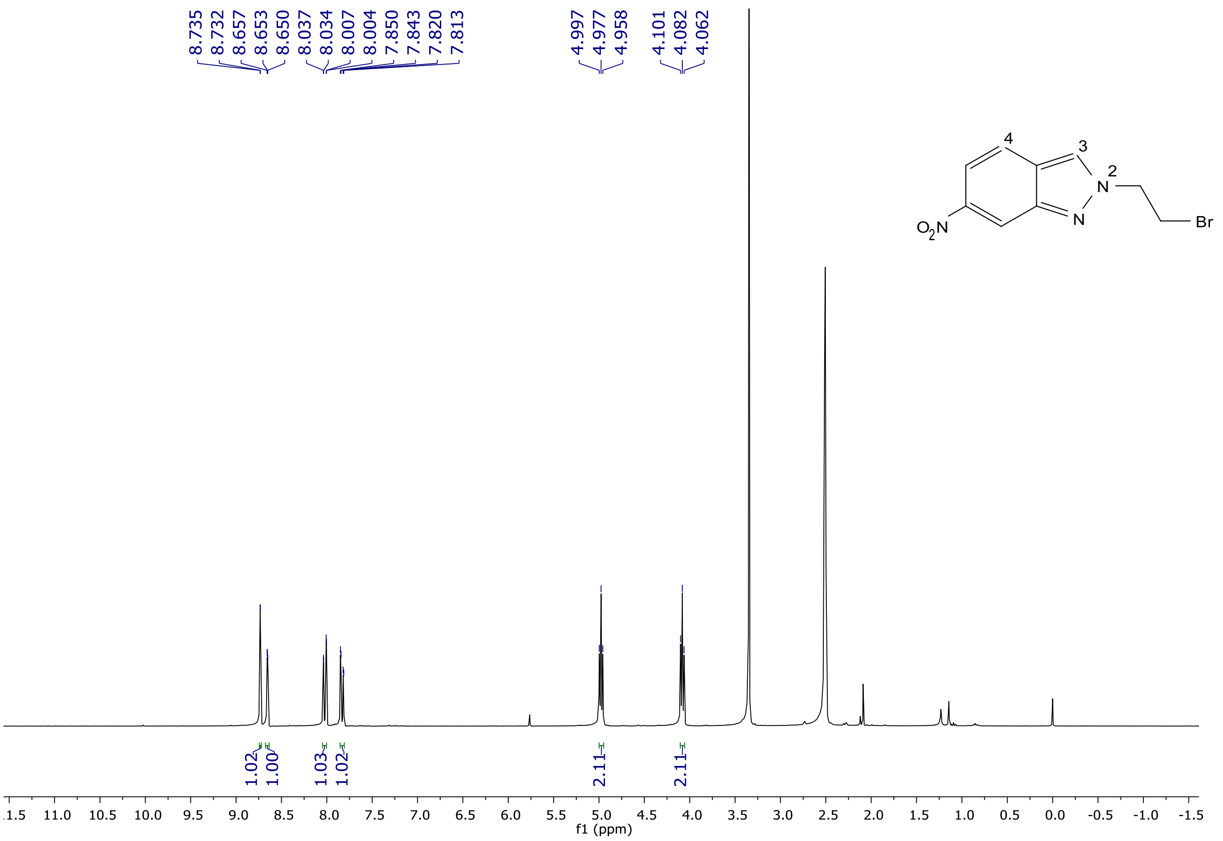

Figure S43. ^{1}H NMR spectrum of compound **4c** in DMSO-d6.

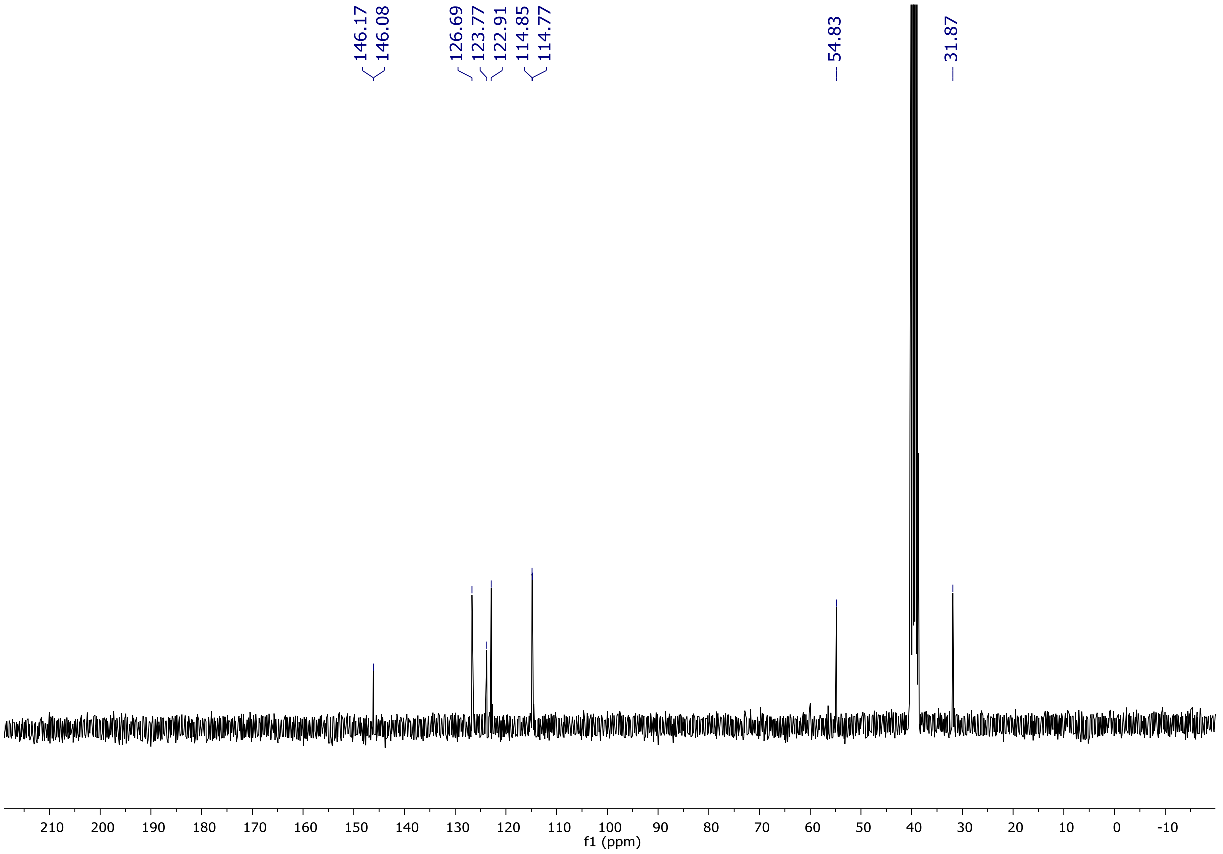

Figure S44. ^{13}C NMR spectrum of compound **4c** in DMSO-d6.

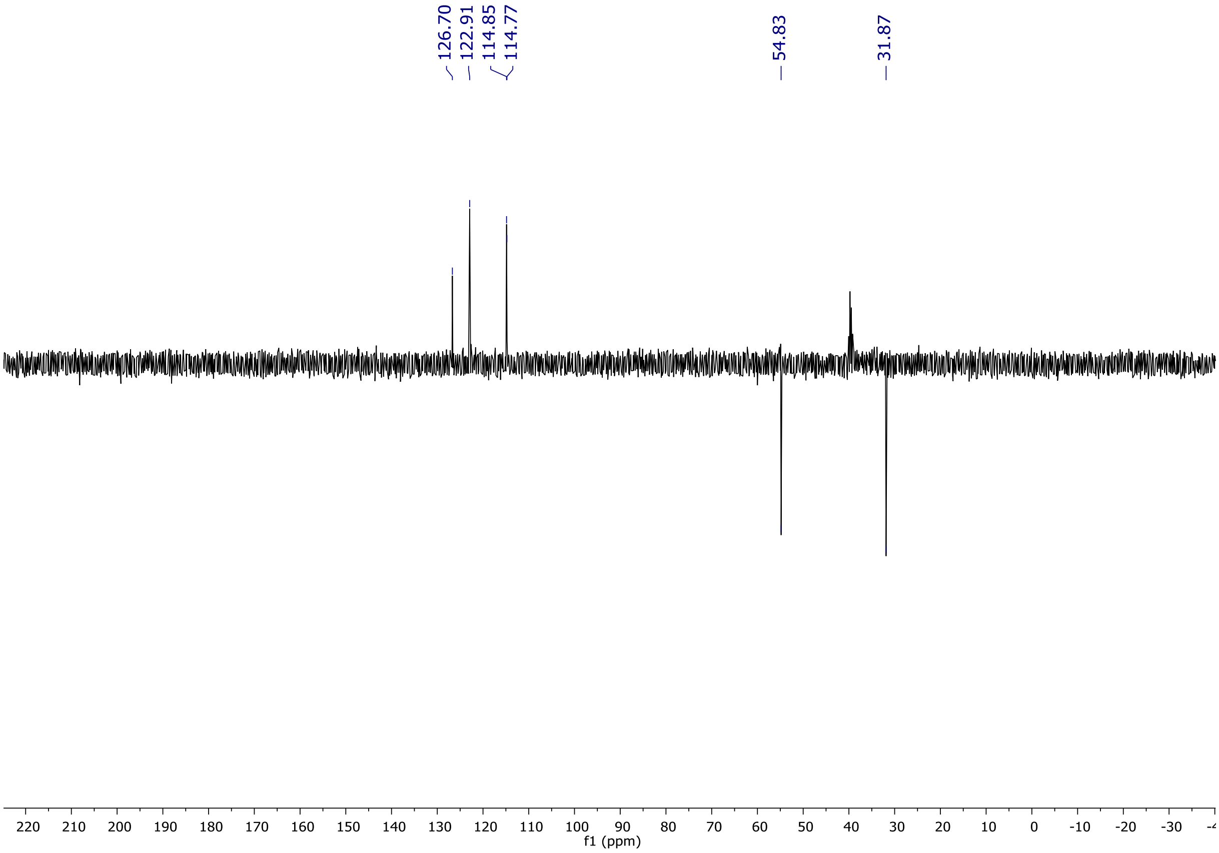

Figure S45. ^{13}C NMR DEPT 135 spectrum of compound **4c** in DMSO-d_6.

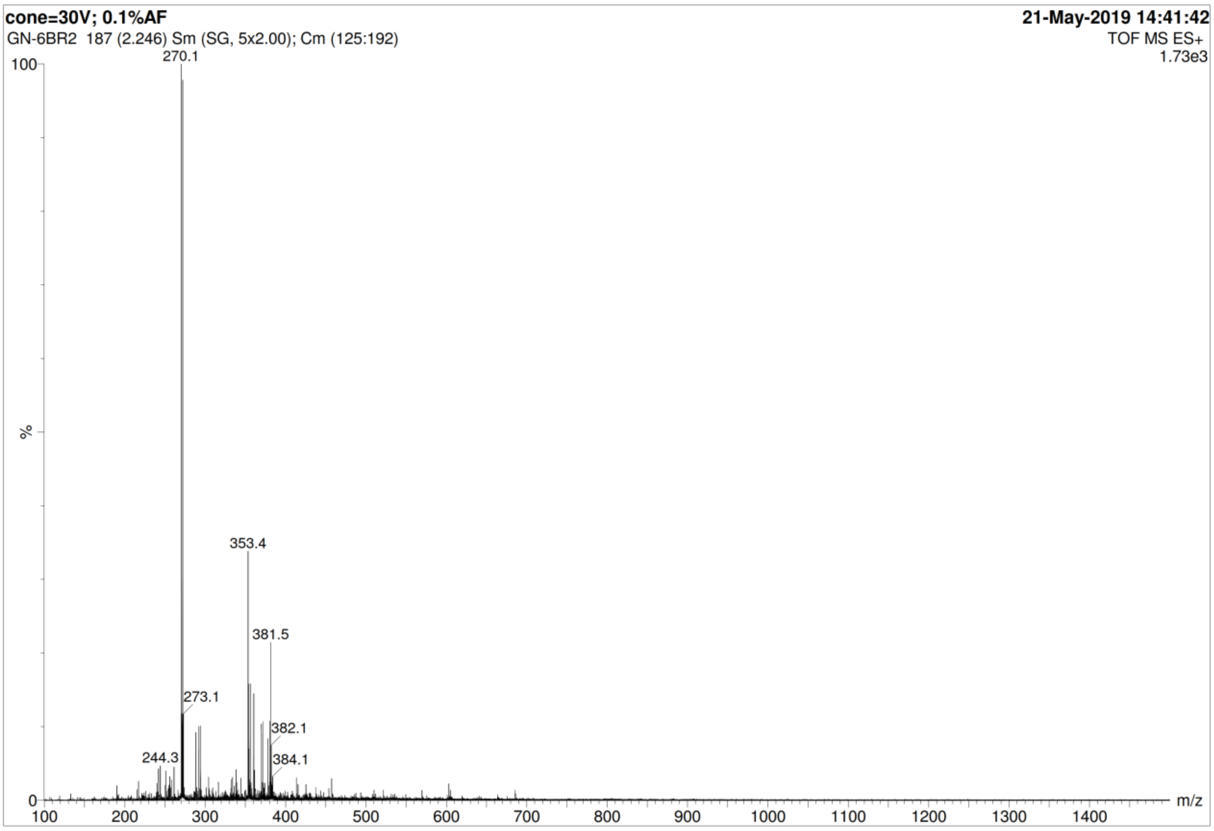

Figure S46. MS-ESI(+) spectrum of compound **4c**.

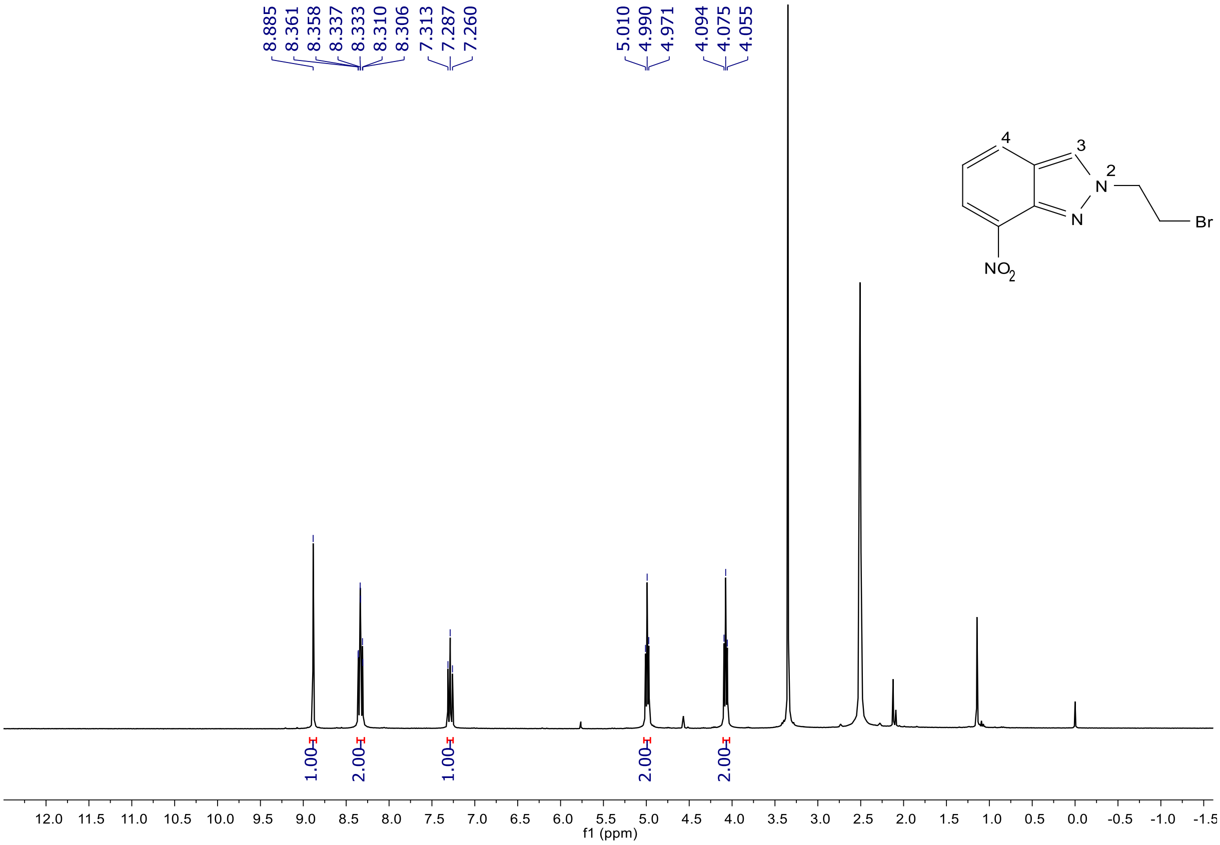

Figure S47. ^{1}H NMR spectrum of compound **4d** in DMSO-d_6.

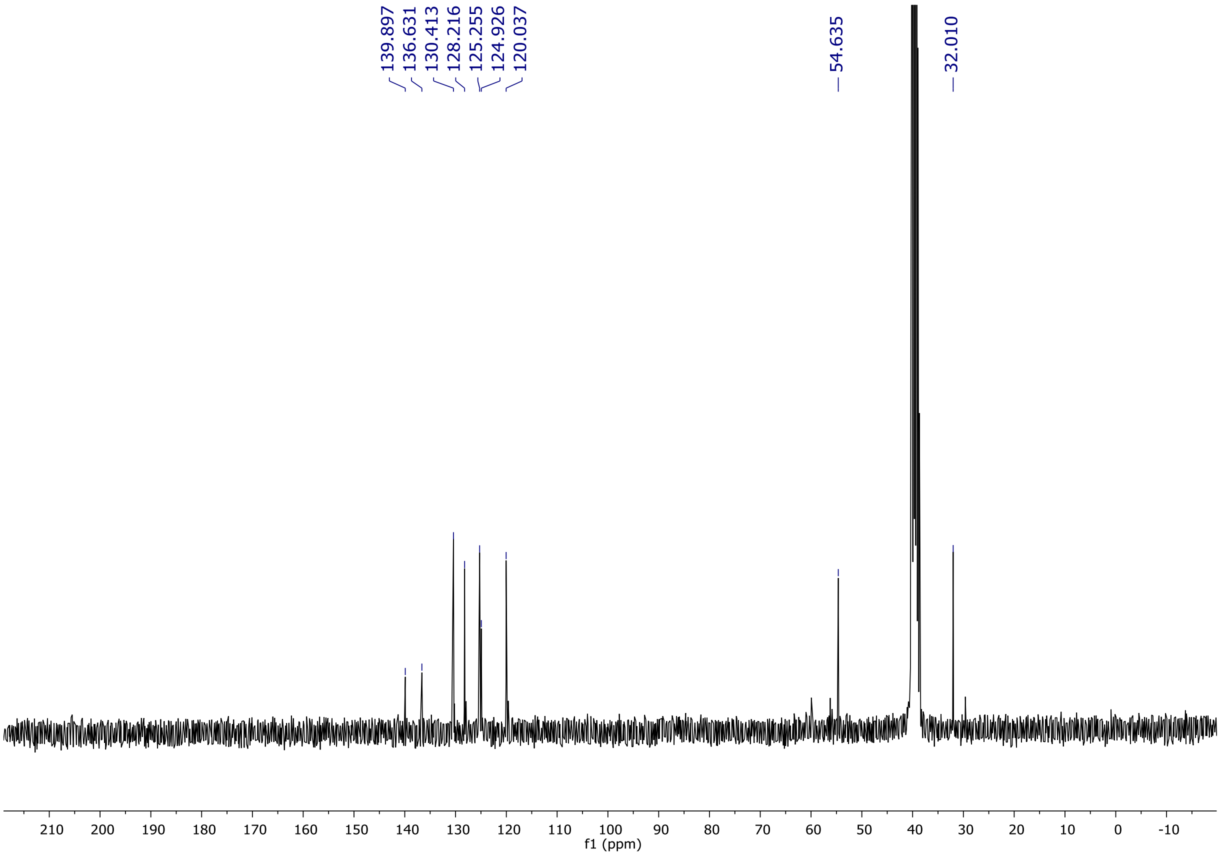

Figure S48. ^{13}C NMR spectrum of compound **4d** in DMSO-d_6.

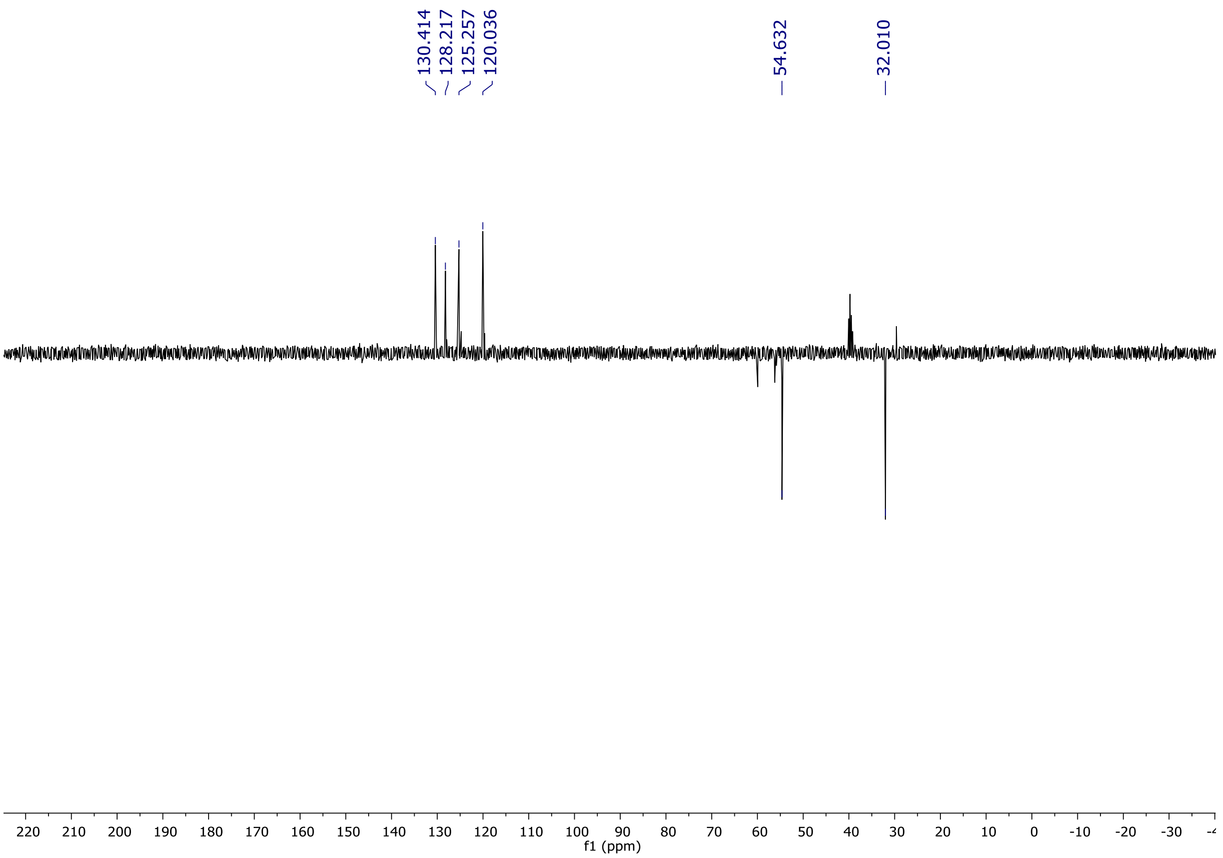

Figure S49. ^{13}C NMR DEPT 135 spectrum of compound **4d** in DMSO-d_6.

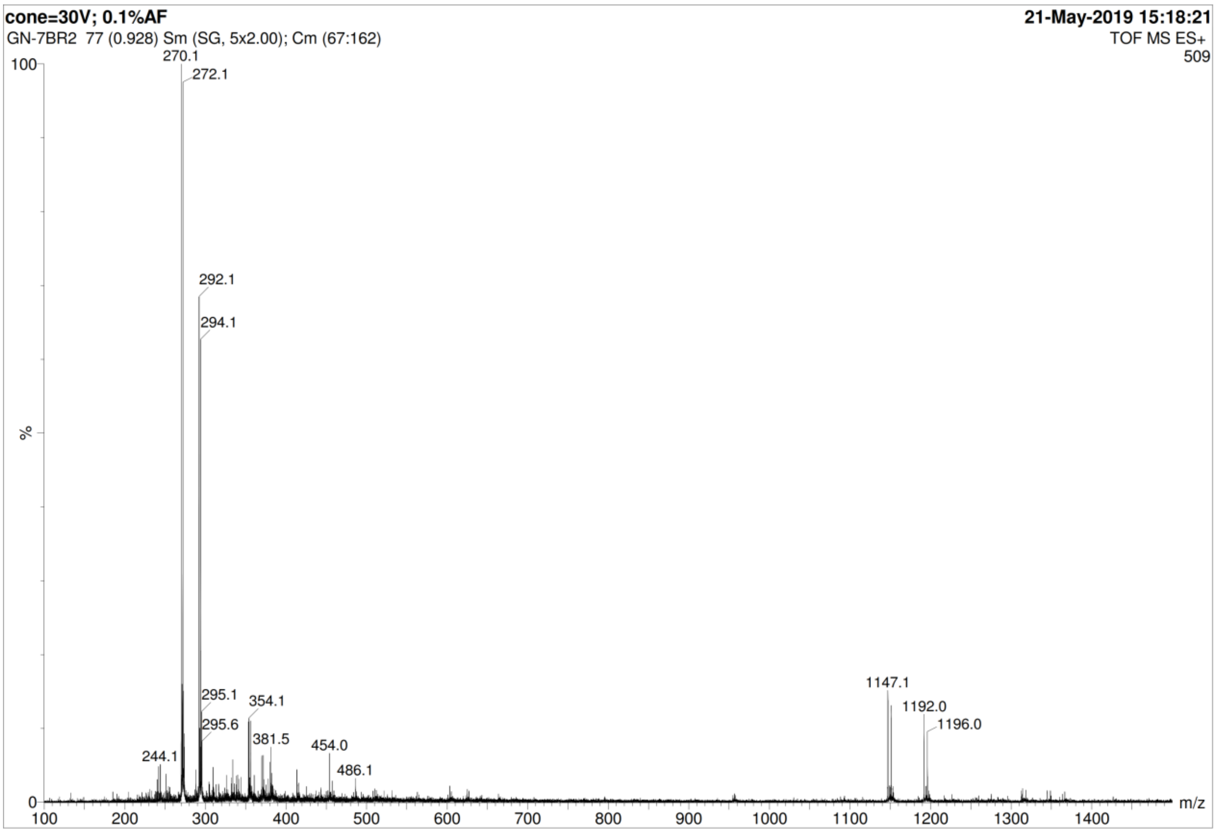

Figure S50. MS-ESI(+) spectrum of compound **4d**.

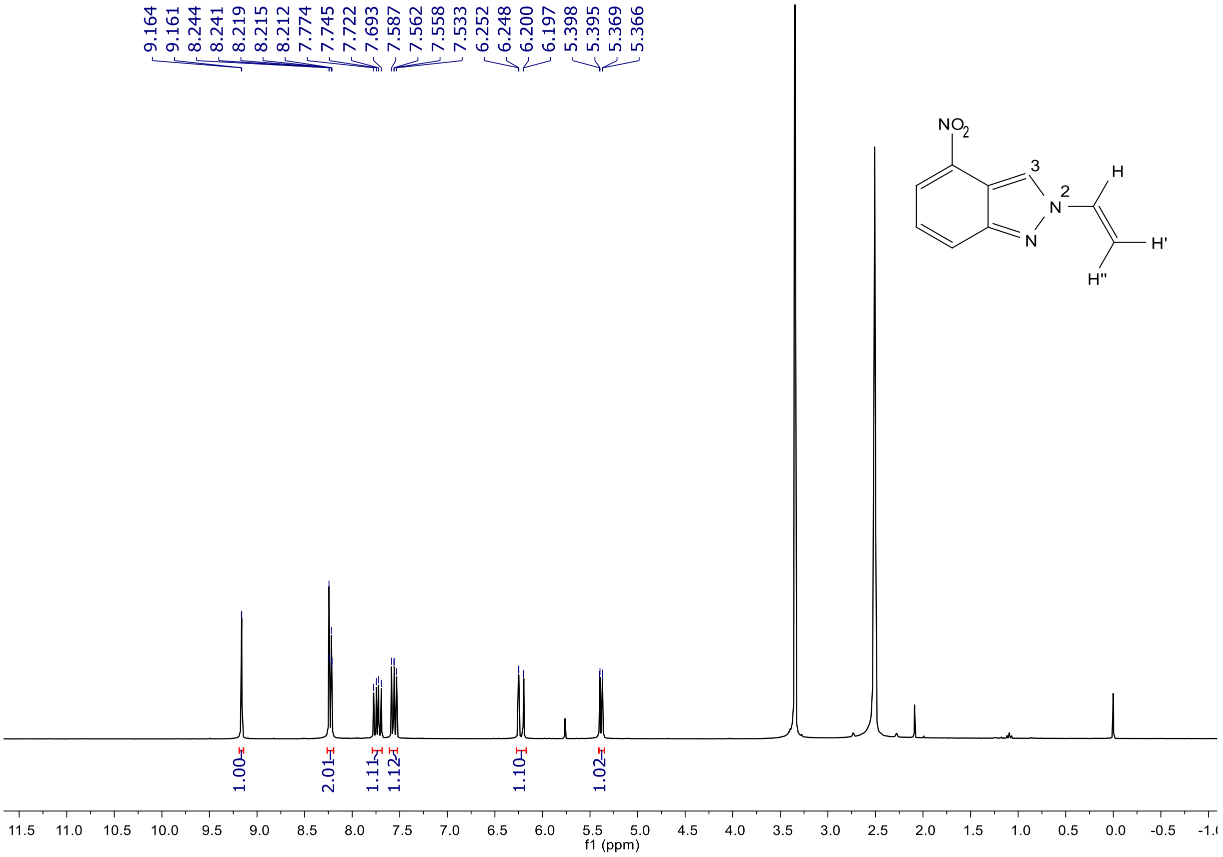

Figure S51. ^{1}H NMR spectrum of compound **5a** in DMSO-d_6.

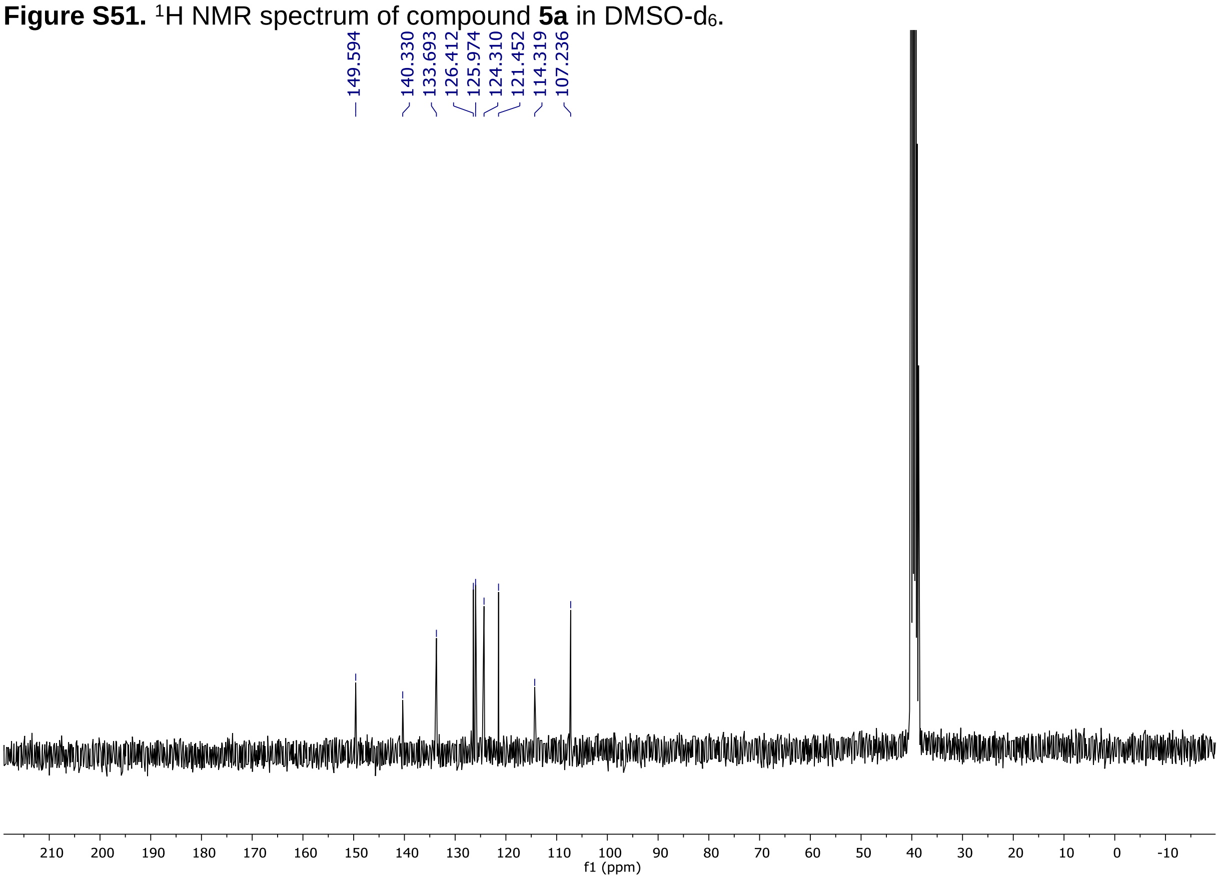

Figure S52. ^{13}C NMR spectrum of compound **5a** in DMSO-d_6.

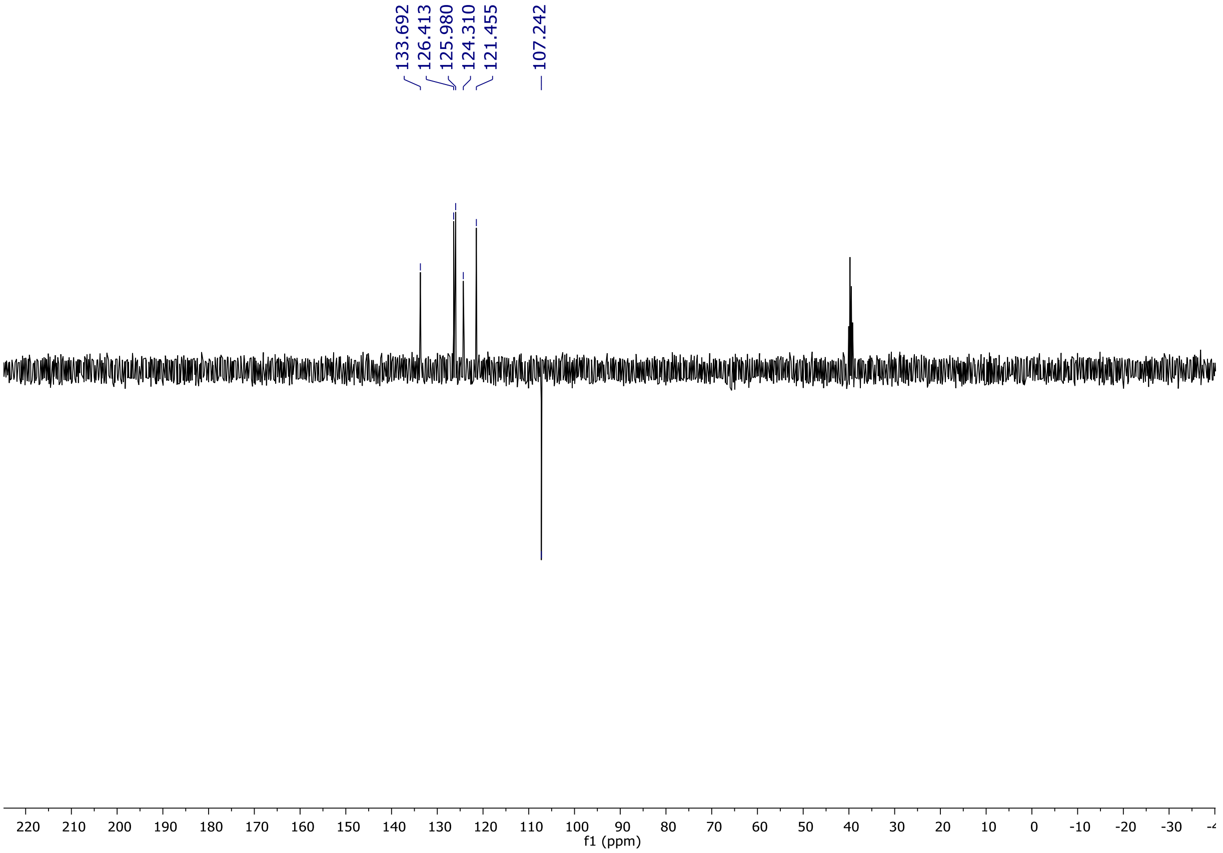

Figure S53. ^{13}C NMR DEPT 135 spectrum of compound **5a** in DMSO-d_6.

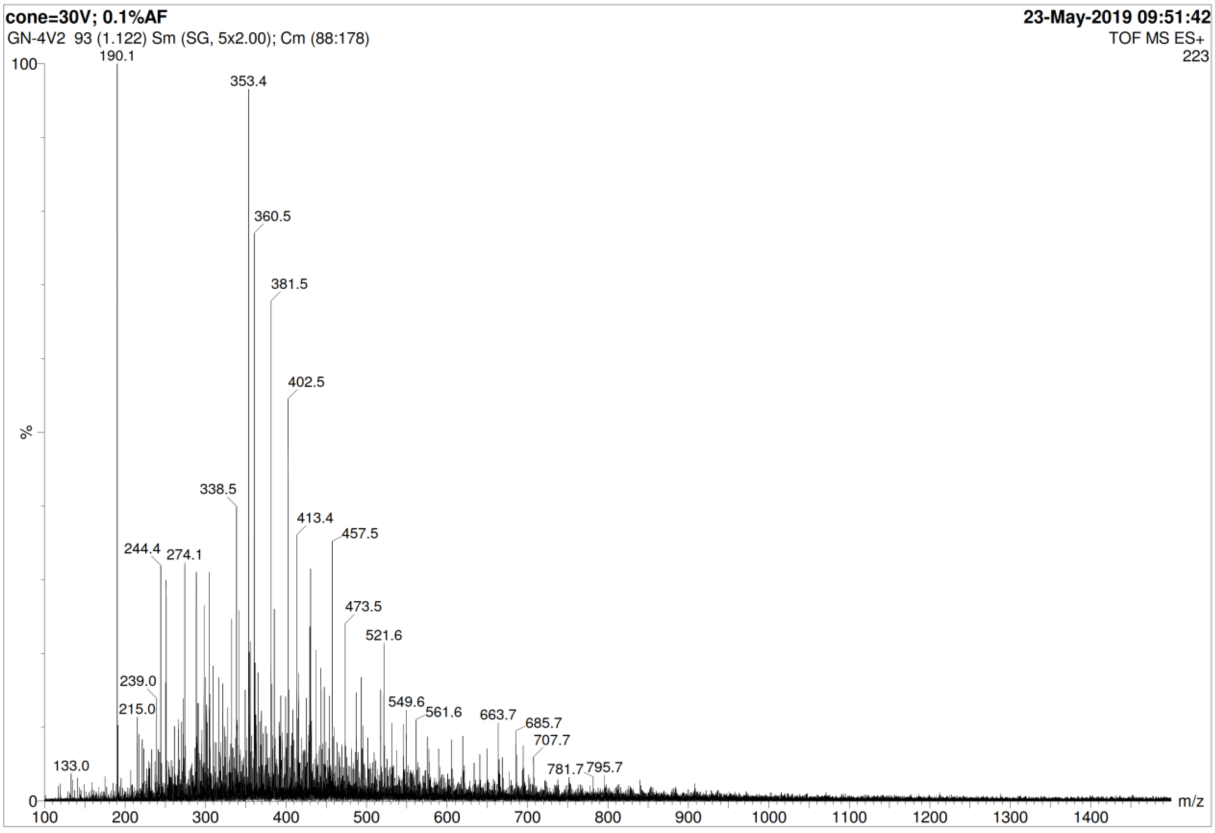

Figure S54. MS-ESI(+) spectrum of compound **5a**.

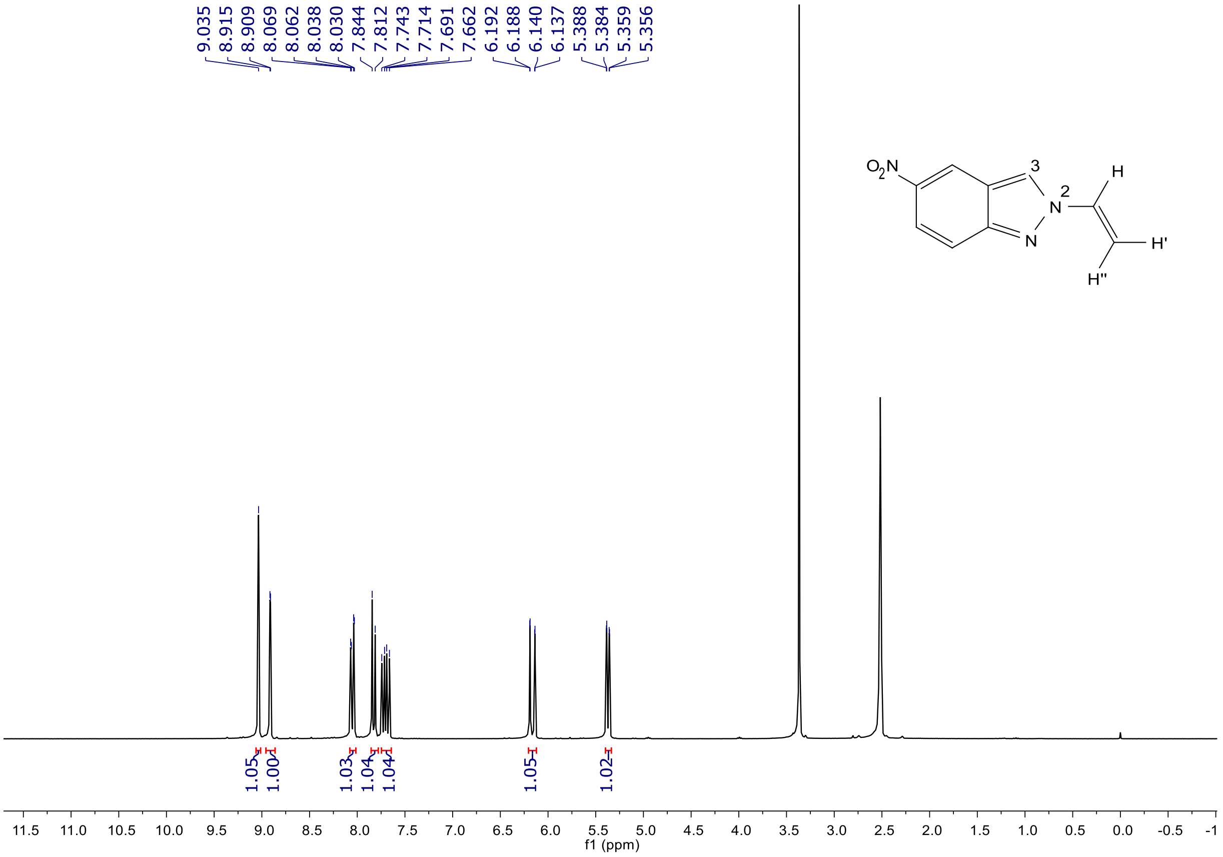

Figure S55. ^{1}H NMR spectrum of compound **5b** in DMSO-d$_6$.

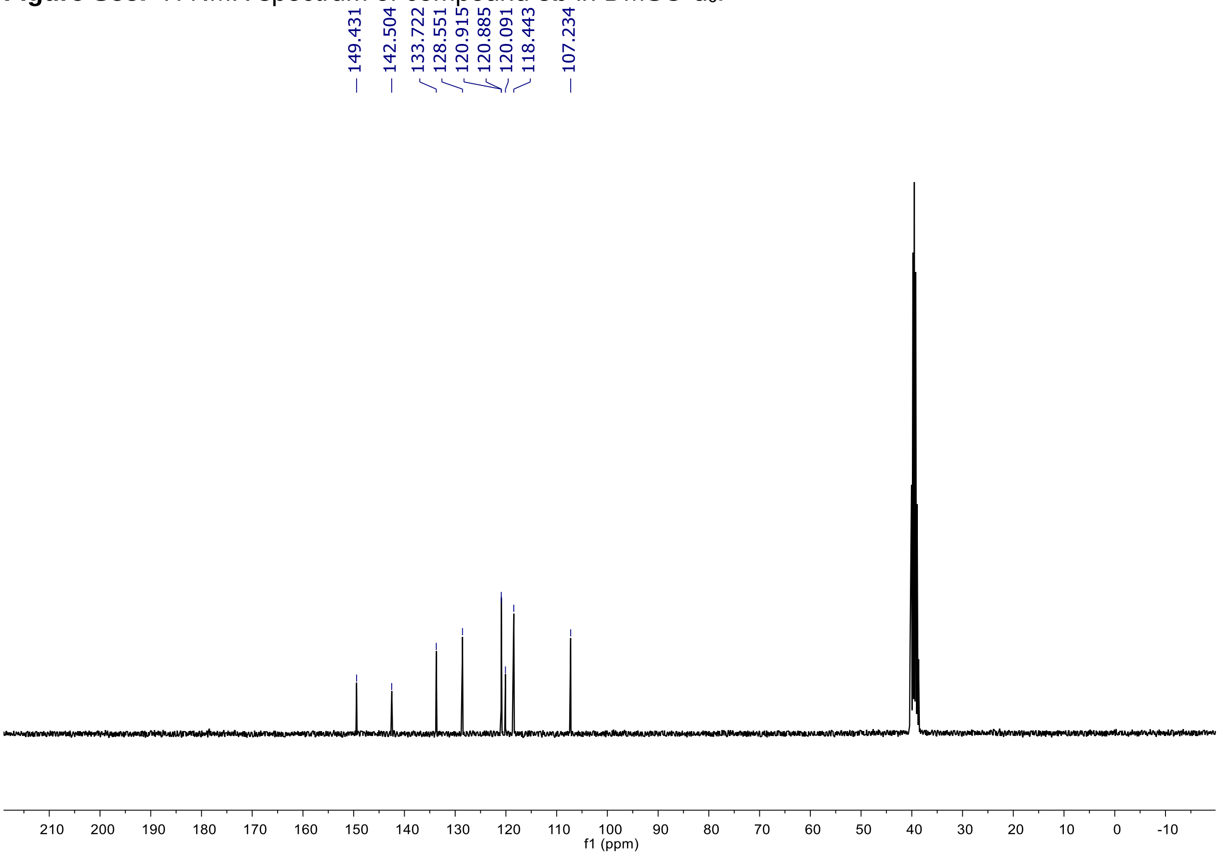

Figure S56. ^{13}C NMR spectrum of compound **5b** in DMSO-d$_6$.

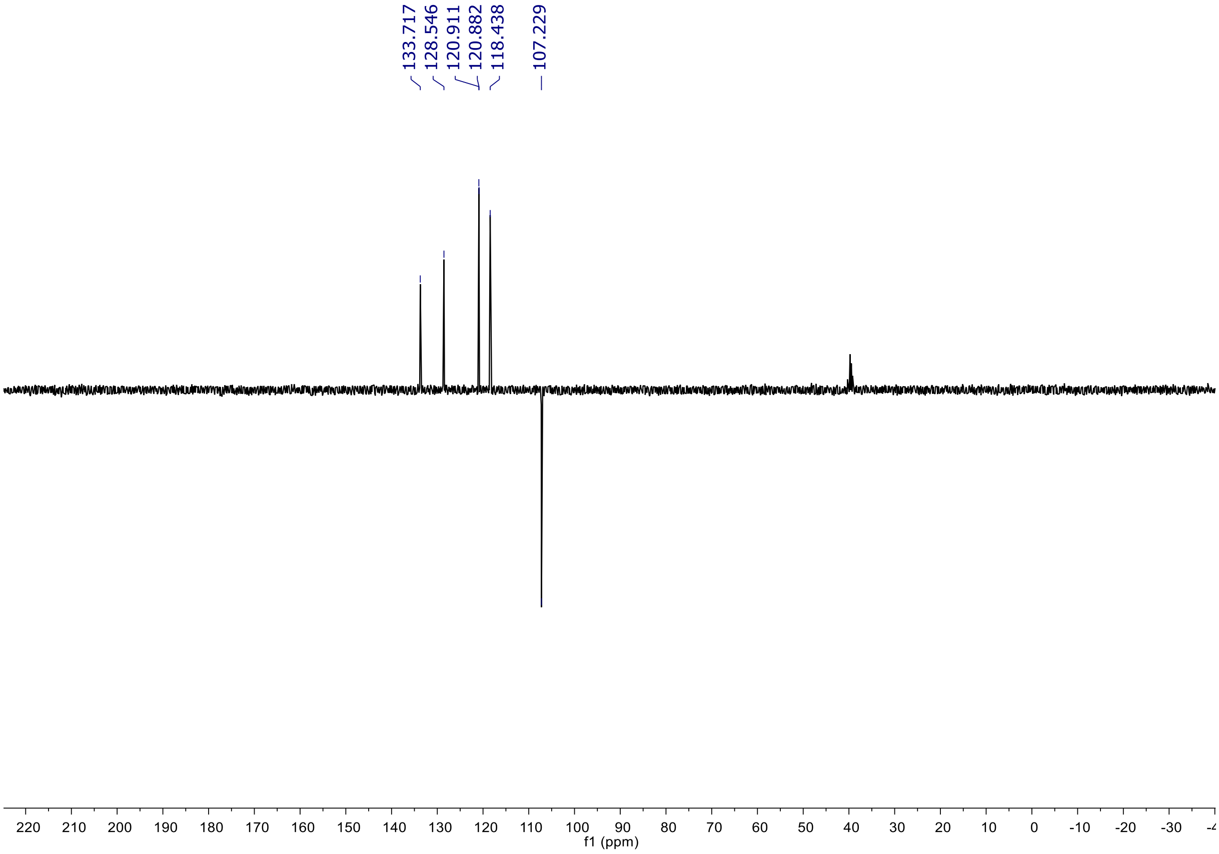

Figure S57. ^{13}C NMR DEPT 135 spectrum of compound **5b** in DMSO-d_6.

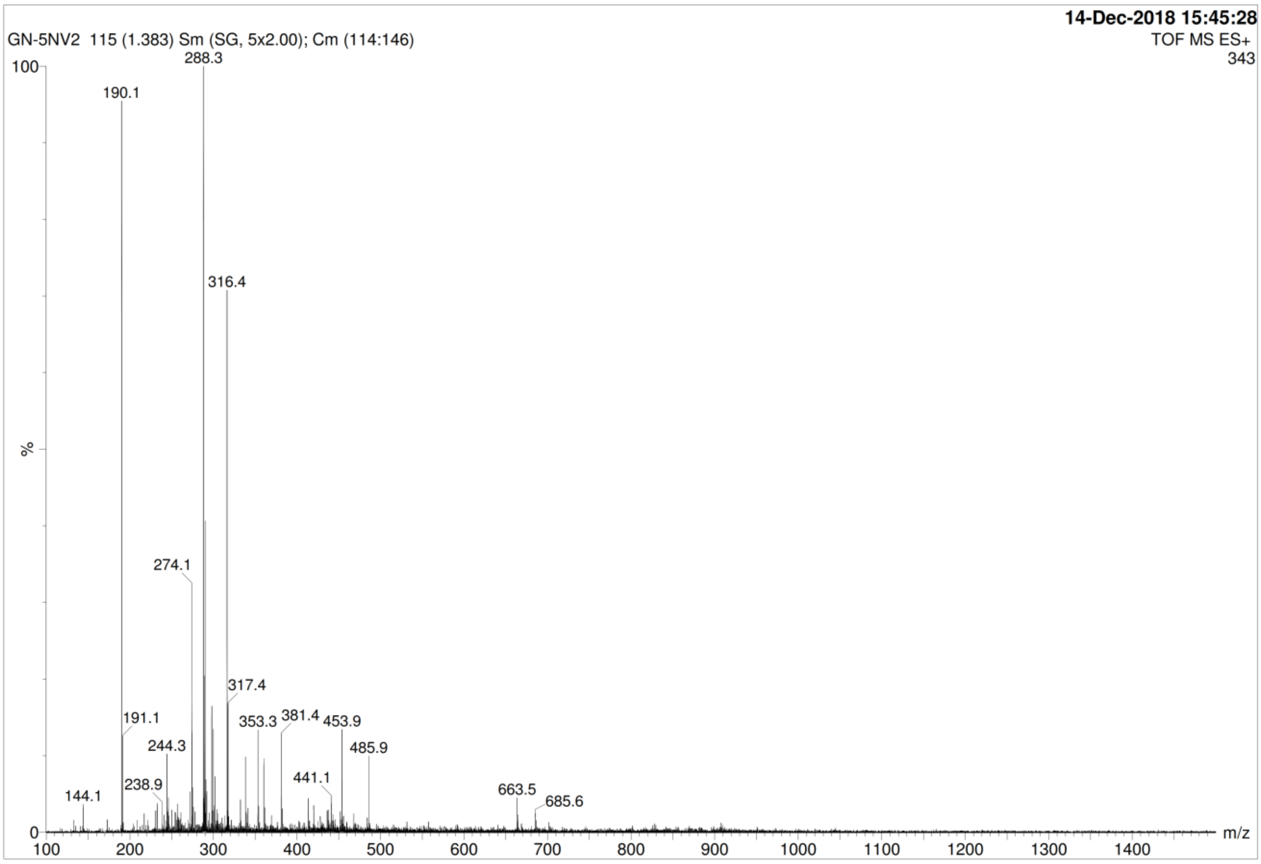

Figure S58. MS-ESI(+) spectrum of compound **5b**

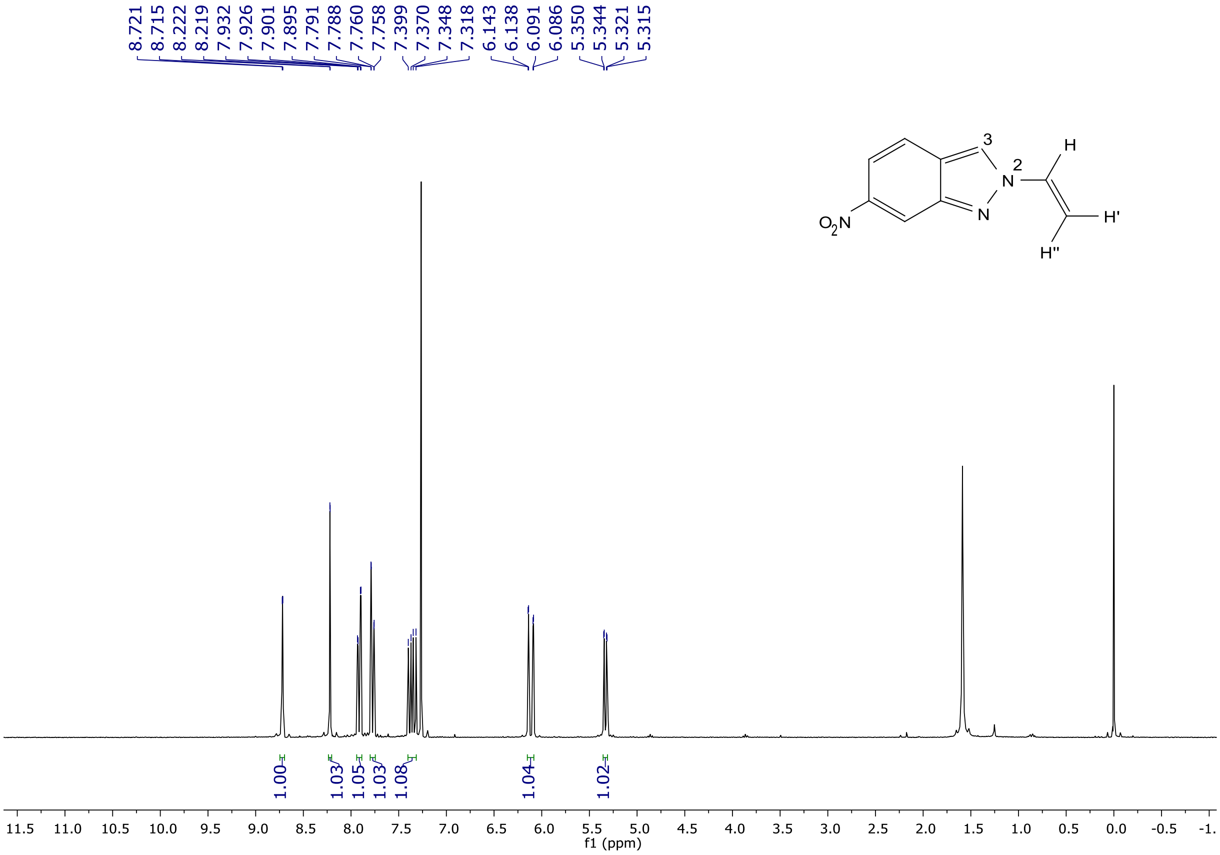

Figure S59. ^{1}H NMR spectrum of compound **5c** in $CDCl_3$.

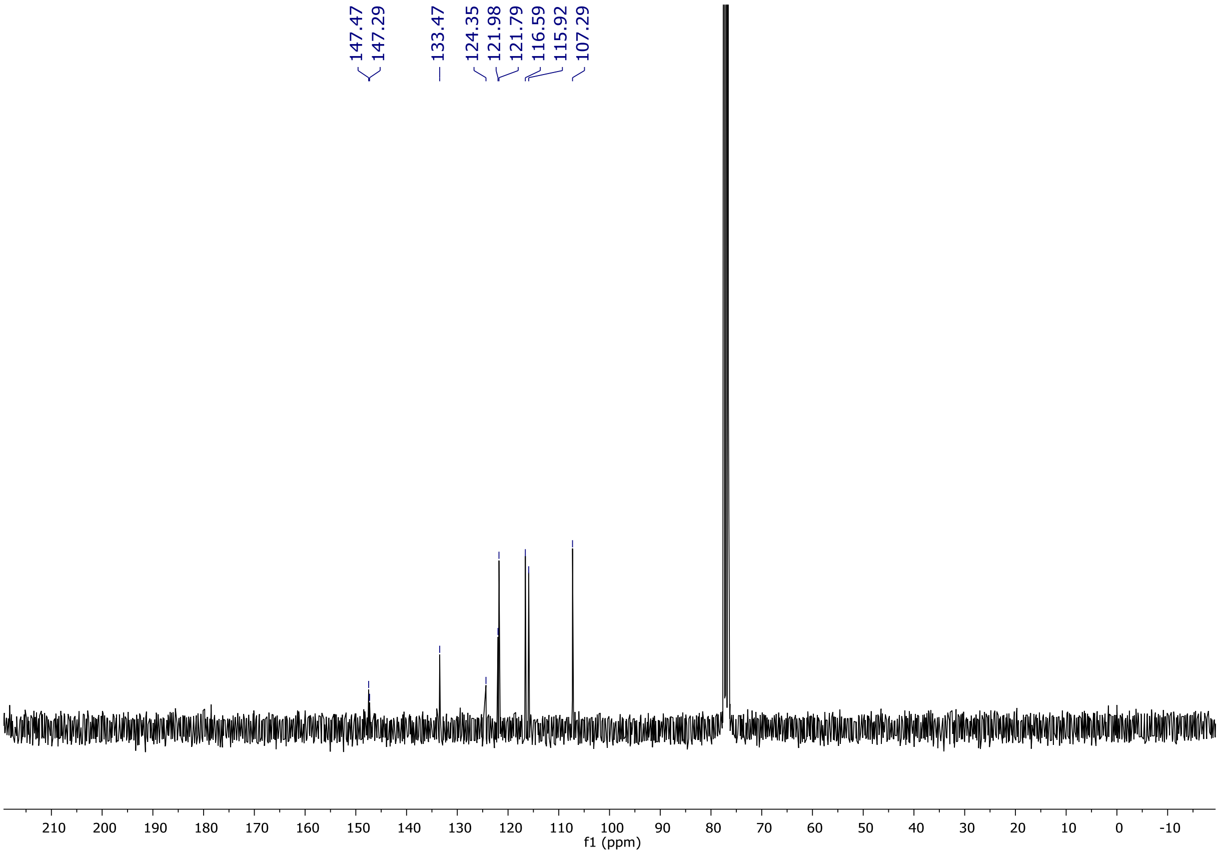

Figure S60. ^{13}C NMR spectrum of compound **5c** in $CDCl_3$.

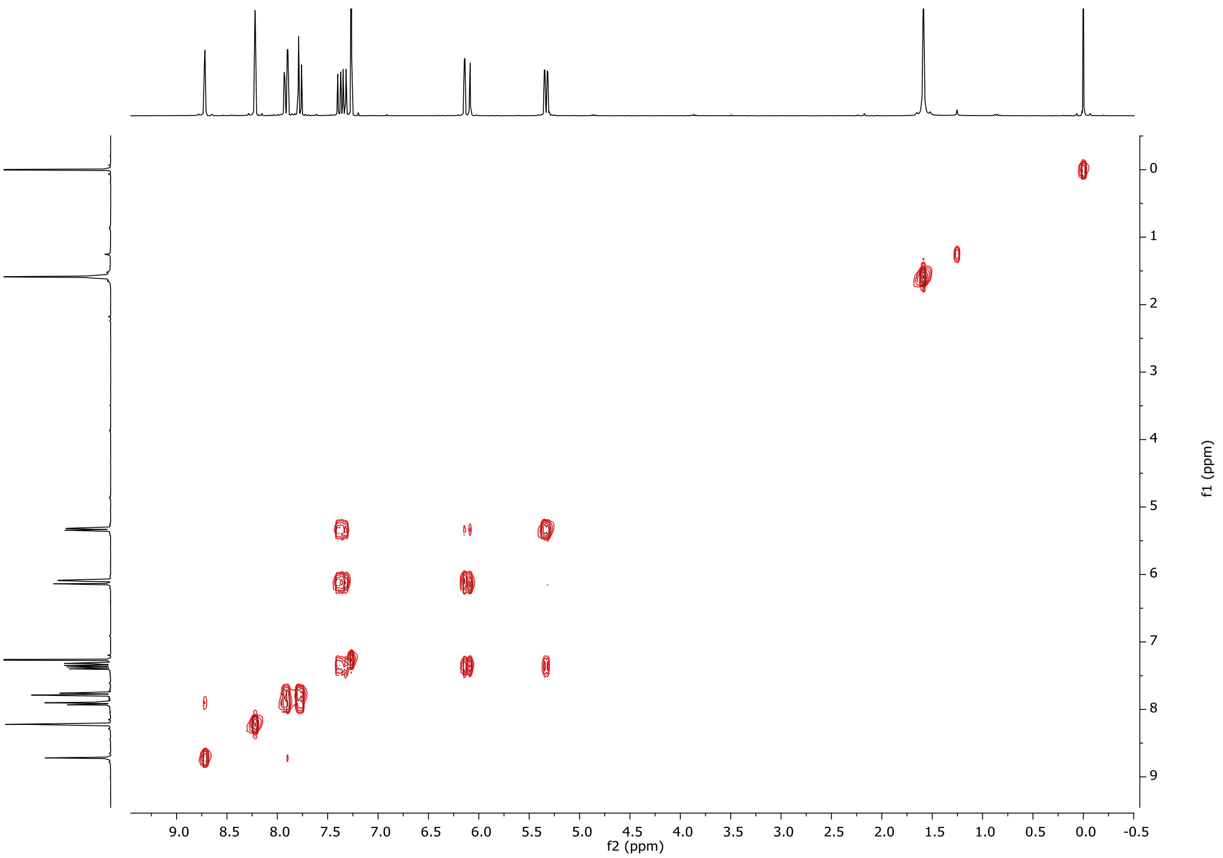

Figure S61. COSY ($^1H/^1H$) spectrum of compound **5c** in $CDCl_3$.

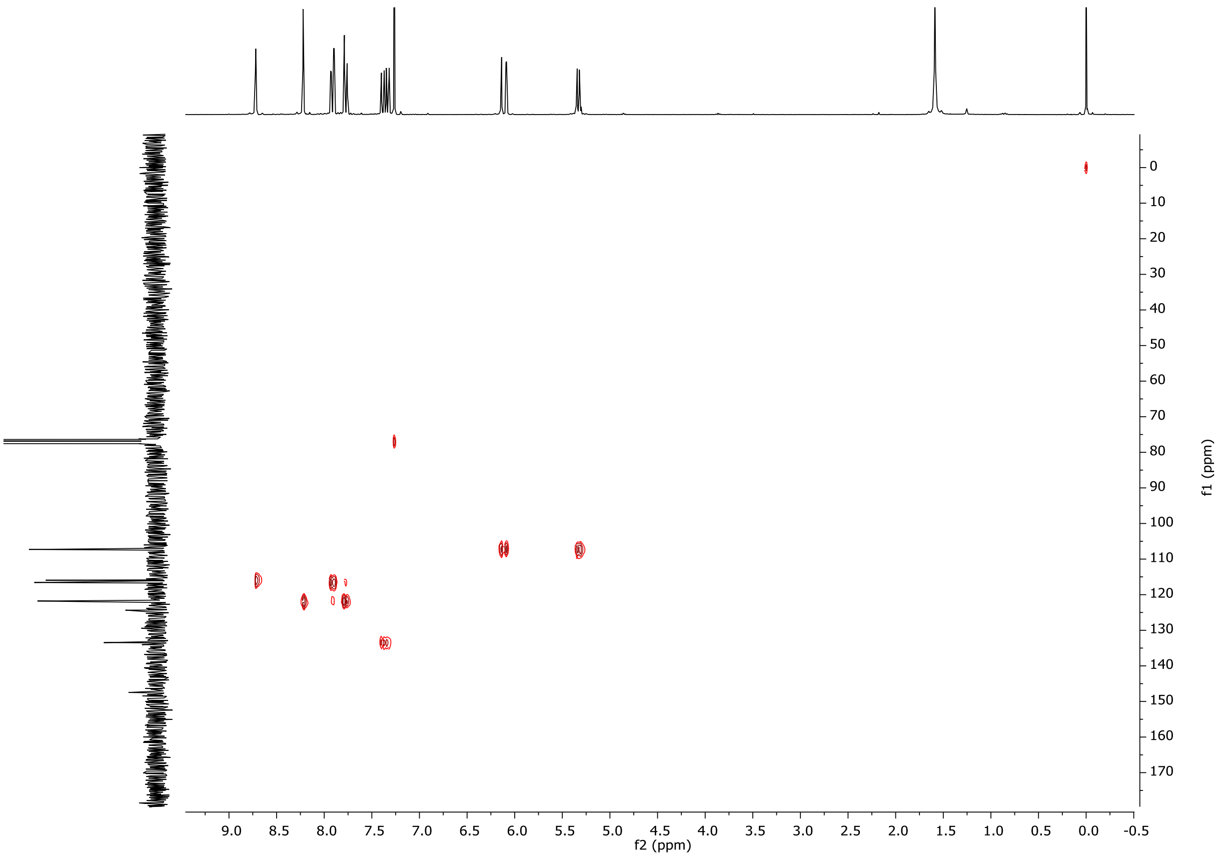

Figure S62. HSQC ($^1H/^{13}C$) spectrum of compound **5c** in $CDCl_3$.

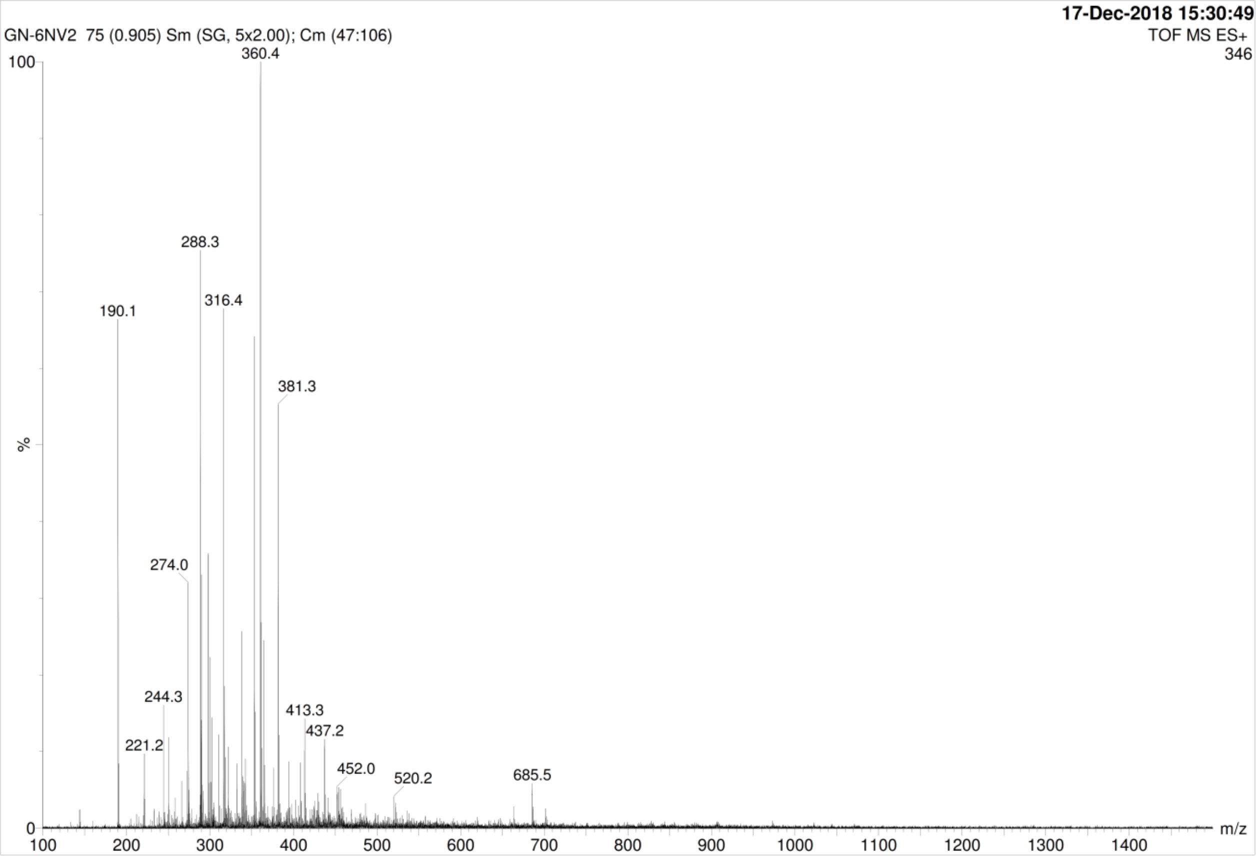

Figure S63. MS-ESI(+) spectrum of compound **5c**

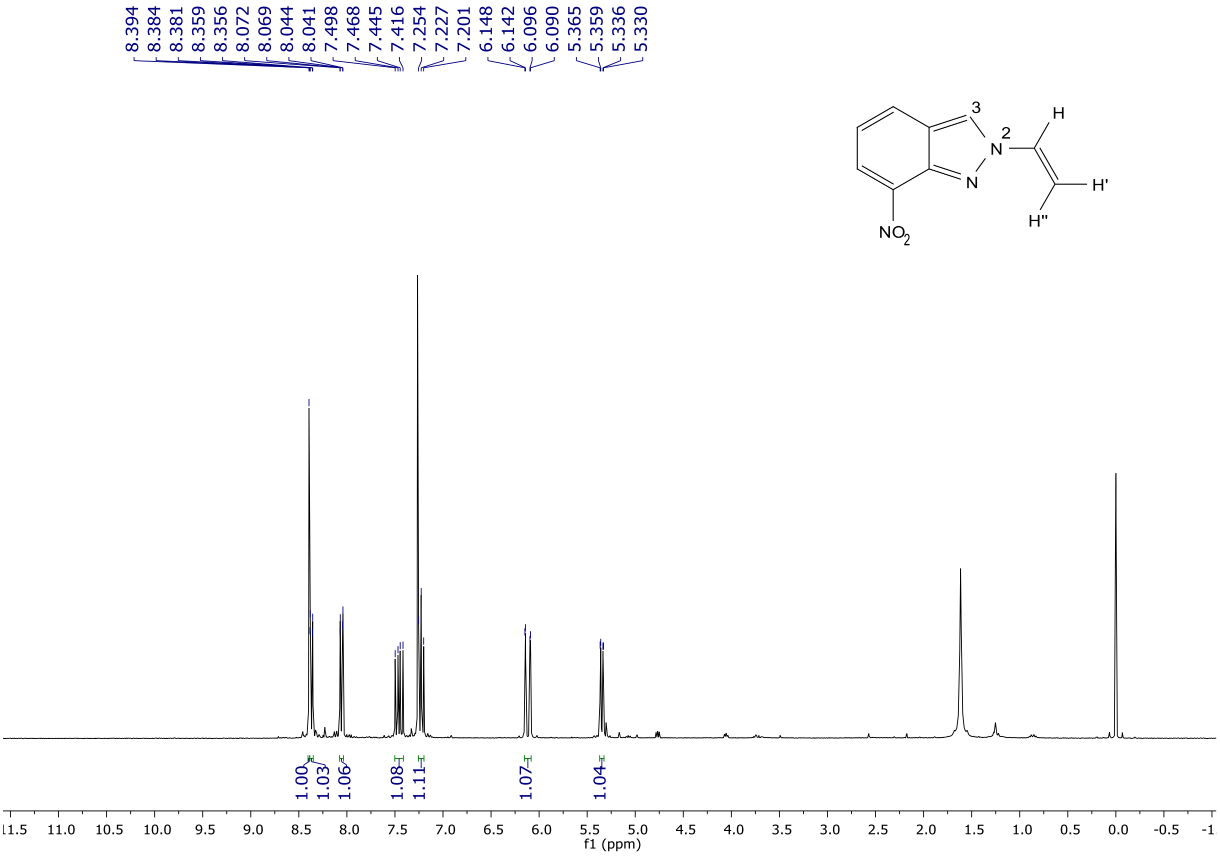

Figure S64. ^{1}H NMR spectrum of compound **5d** in $CDCl_3$.

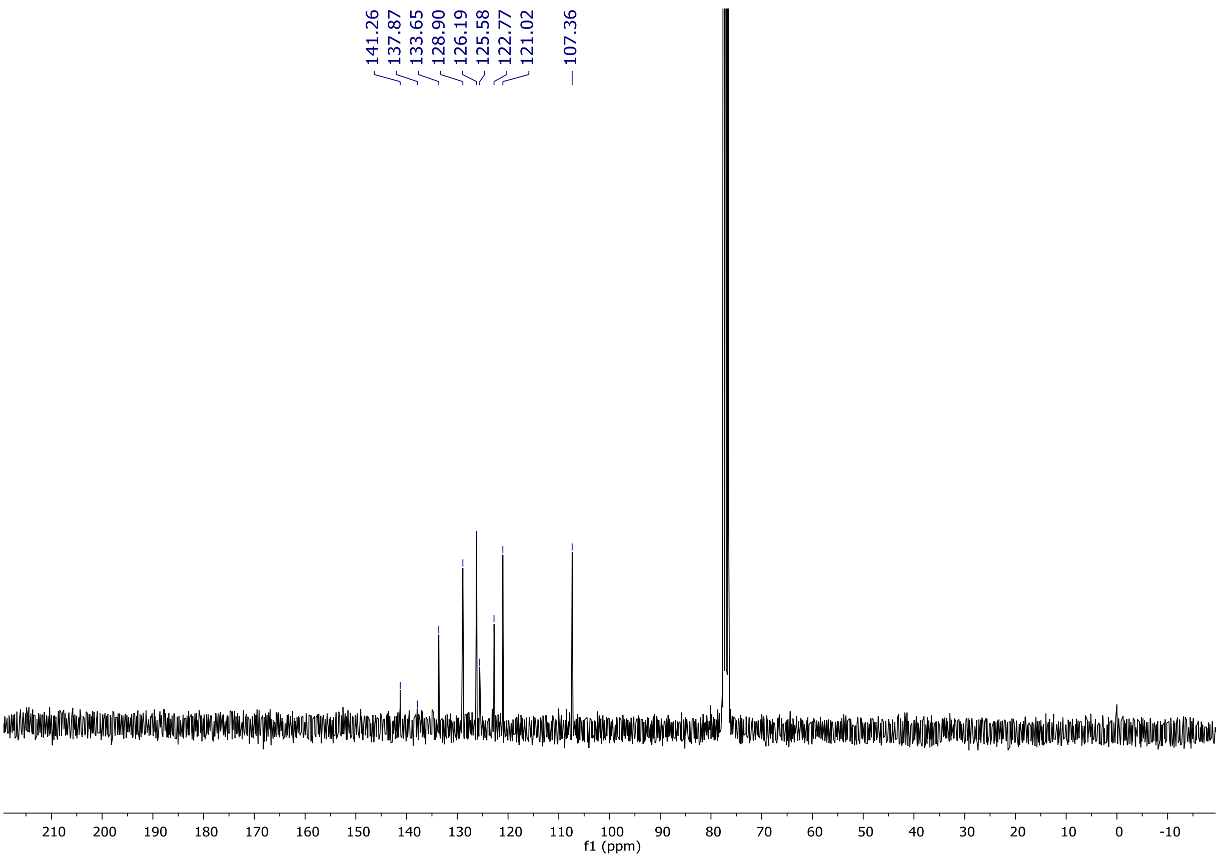

Figure S65. ^{13}C NMR spectrum of compound **5d** in $CDCl_3$.

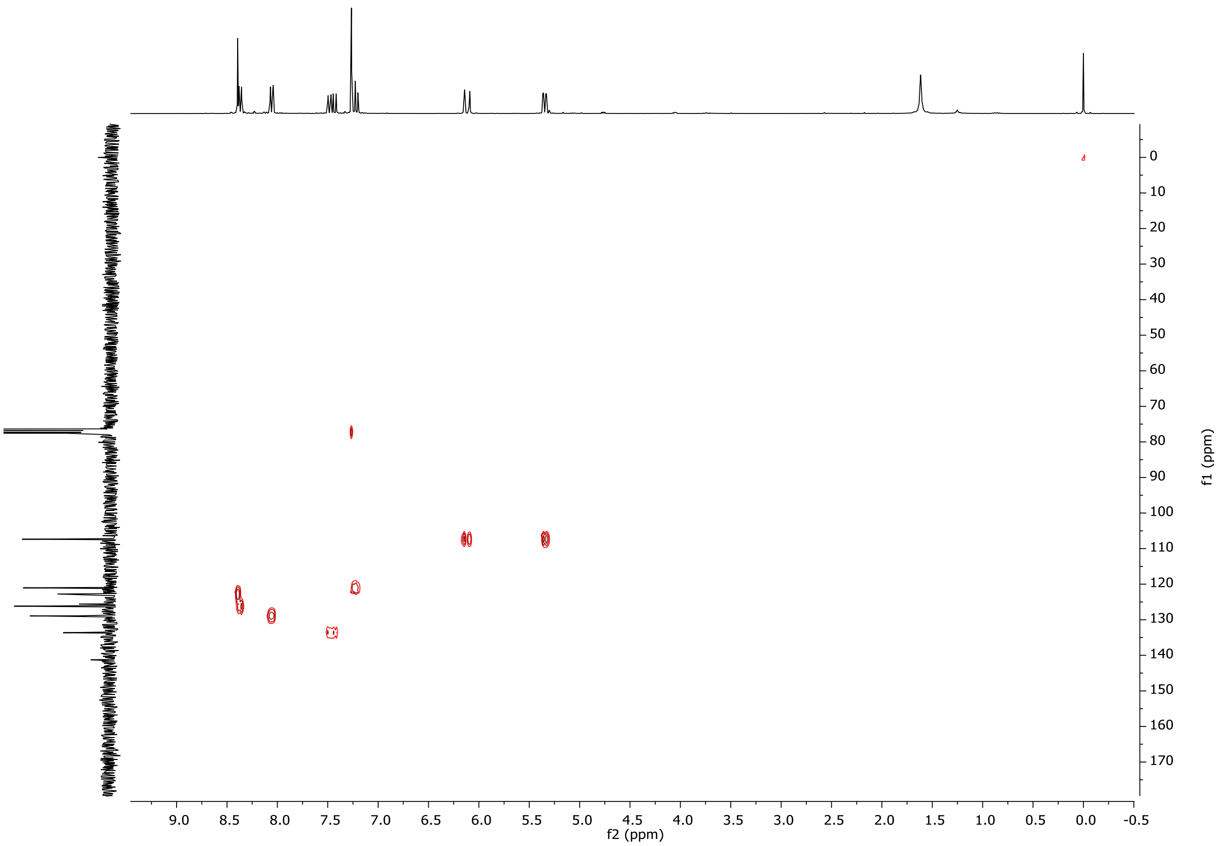

Figure S66. HSQC ($^{1}H/^{13}C$) spectrum of compound **5d** in $CDCl_3$.

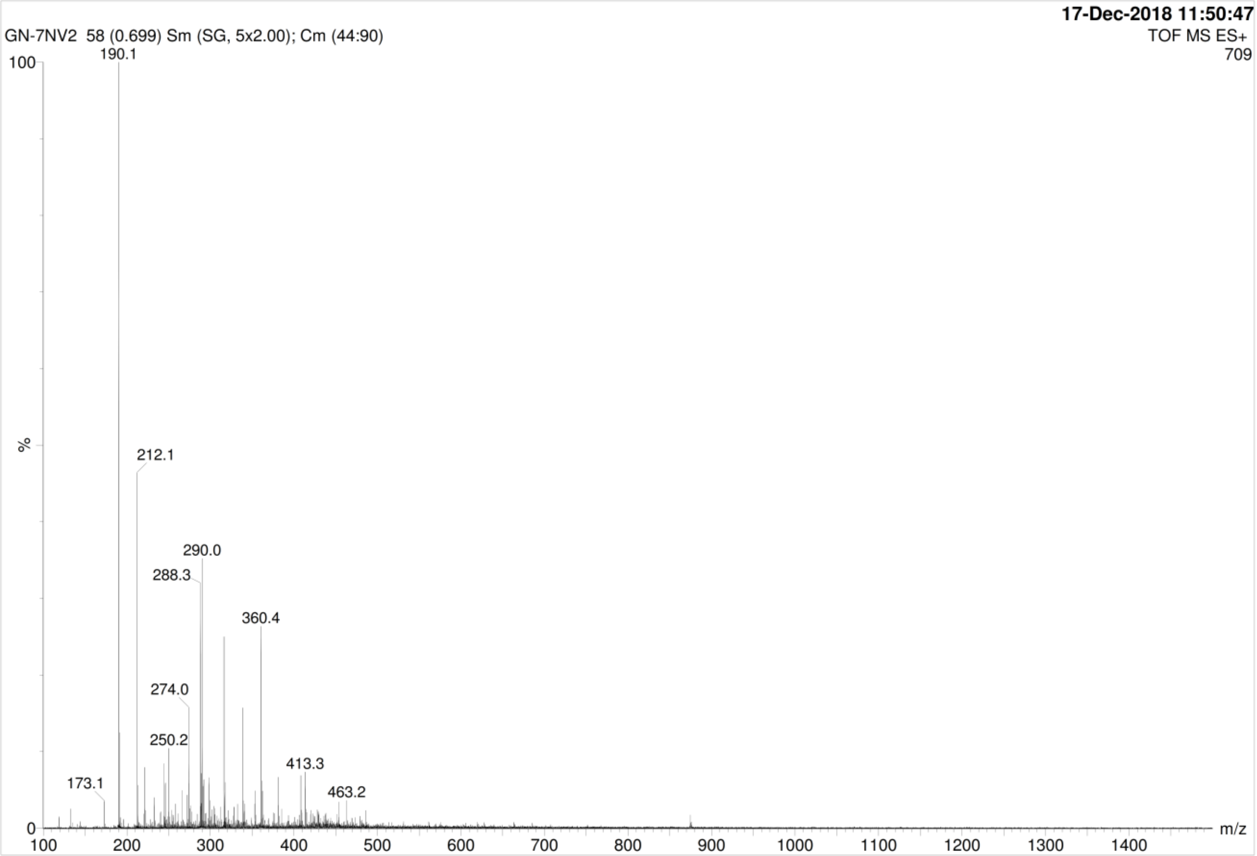

Figure S67. MS-ESI(+) spectrum of compound **5d**.

II - NMR and mass spectrum of compounds 6, 7a-d, 8 and 9

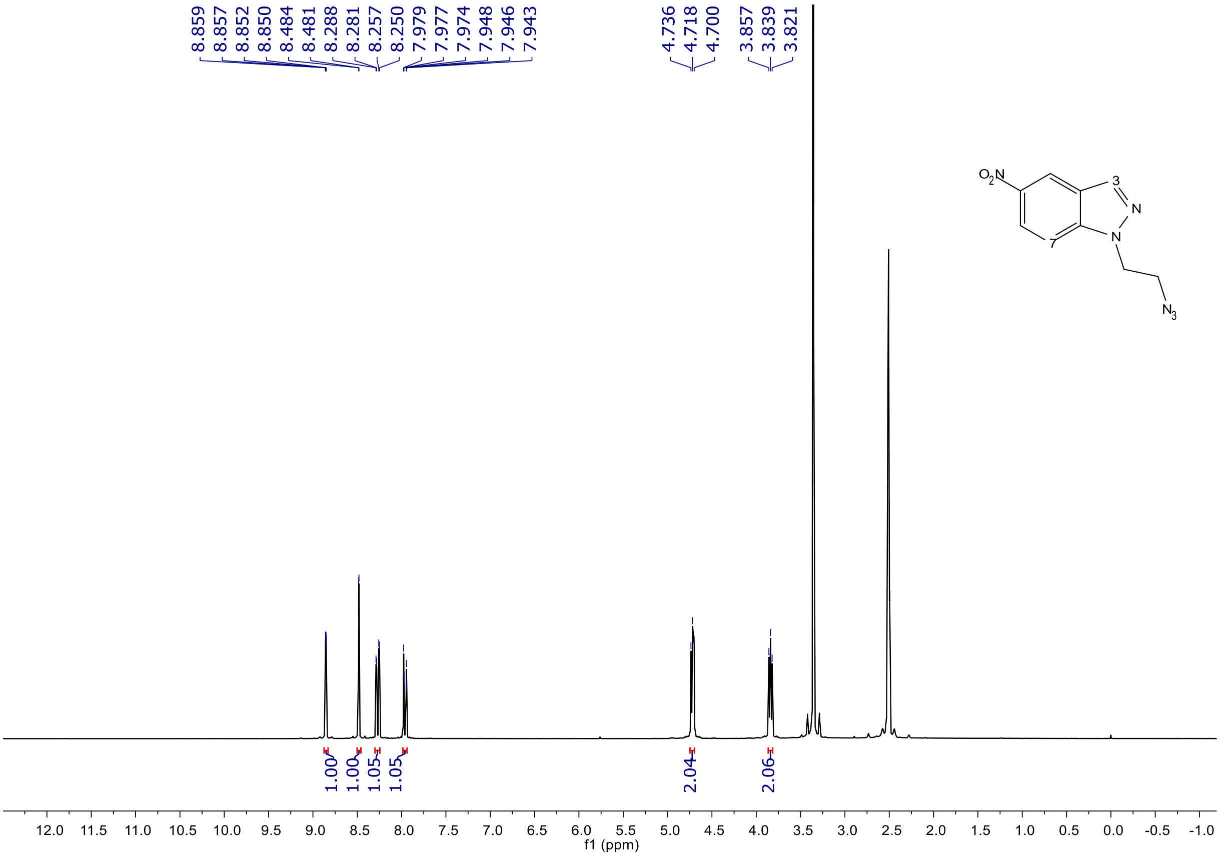

Figure S68. ^{1}H NMR spectrum of compound **6** in DMSO-d$_6$.

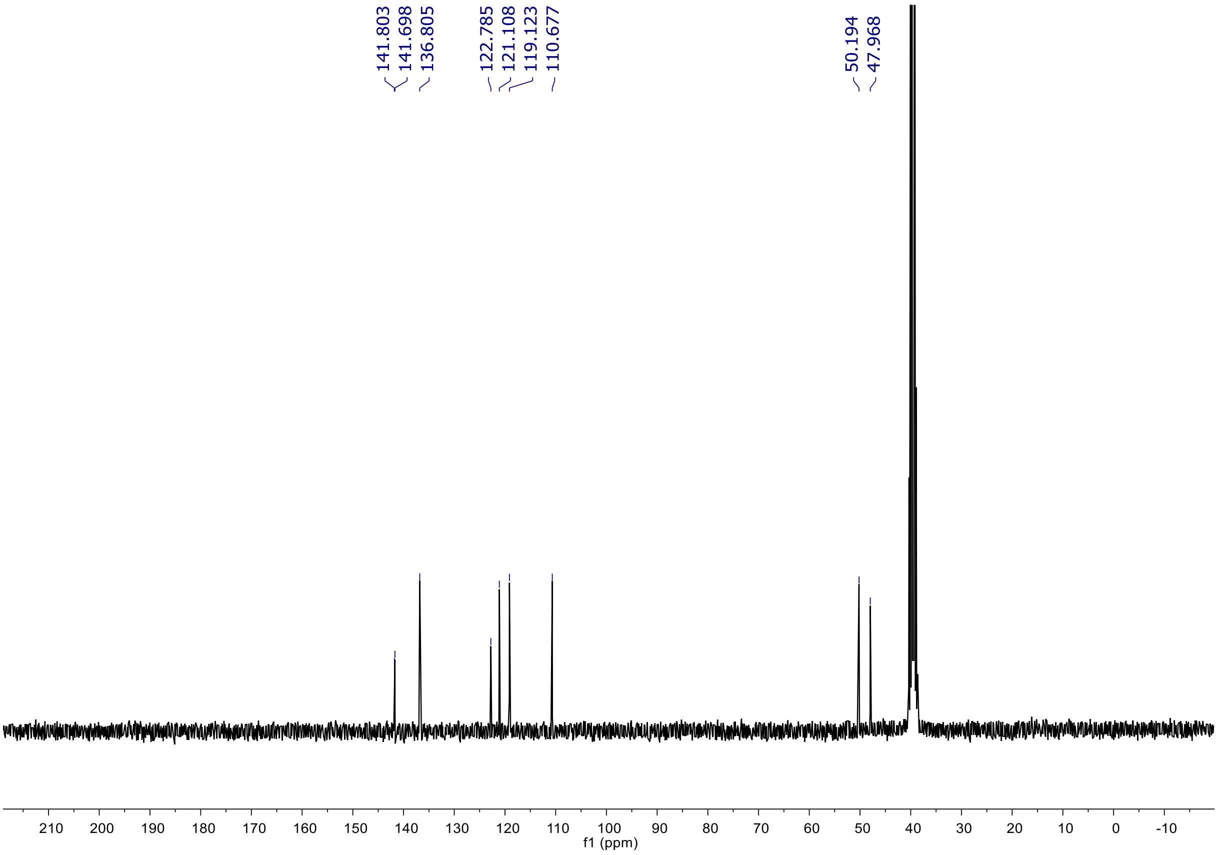

Figure S69. ^{13}C NMR spectrum of compound **6** in DMSO-d$_6$.

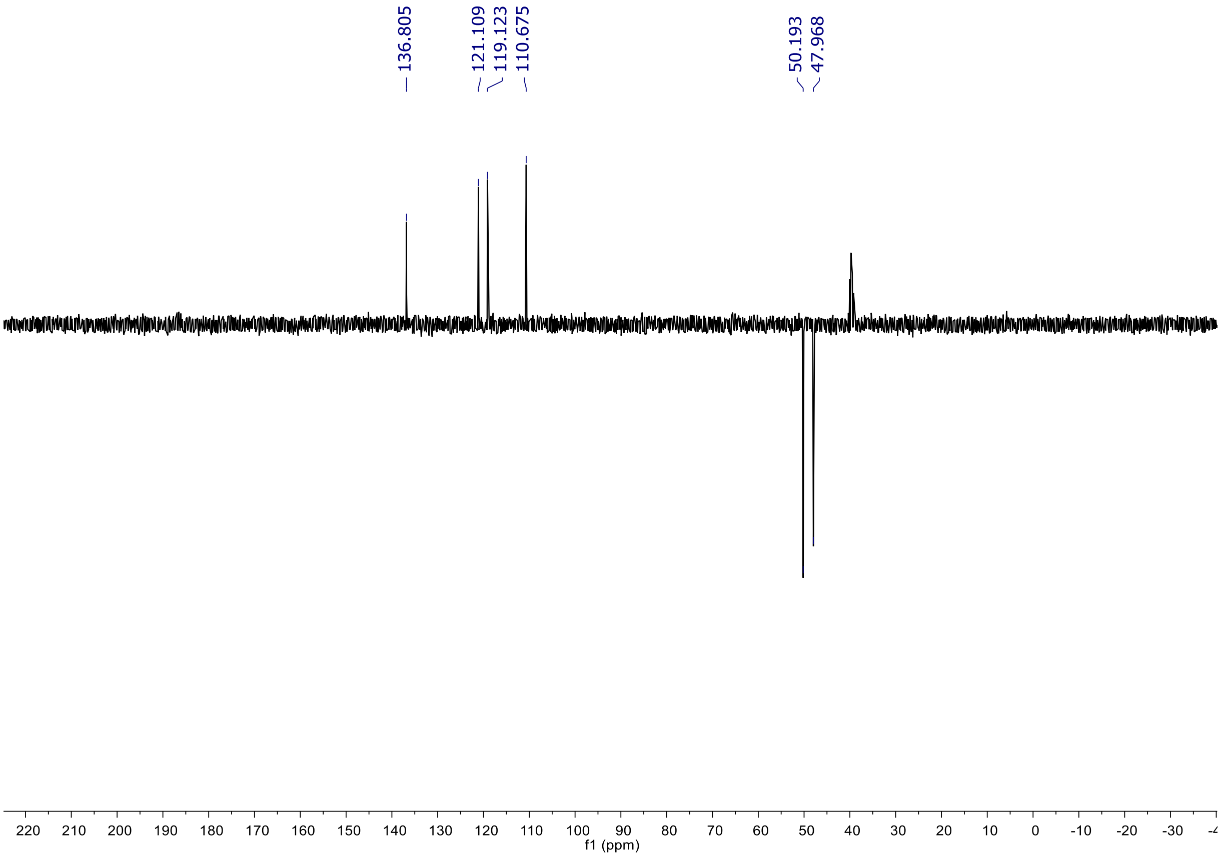

Figure S70. ^{13}C NMR DEPT 135 spectrum of compound **6** in DMSO-d_6.

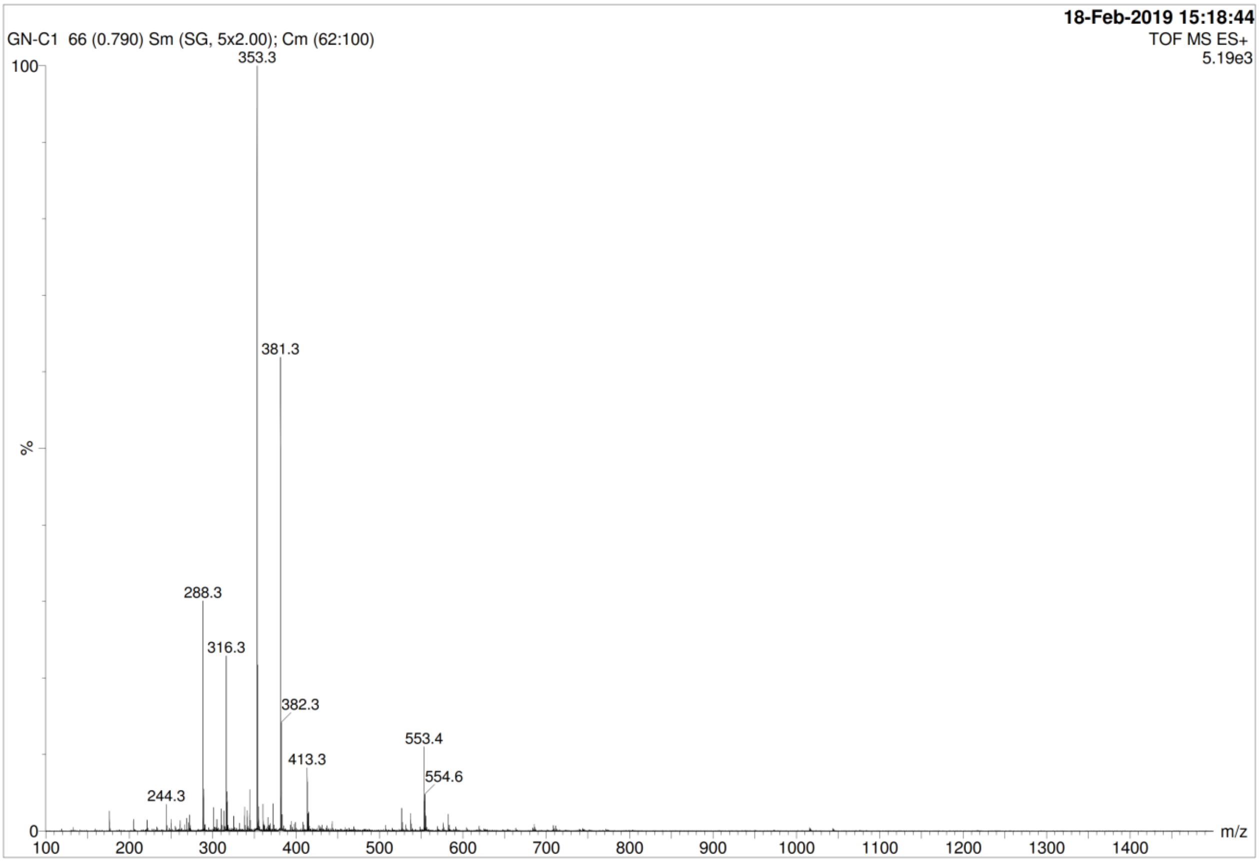

Figure S71. MS-ESI(+) spectrum of compound **6**.

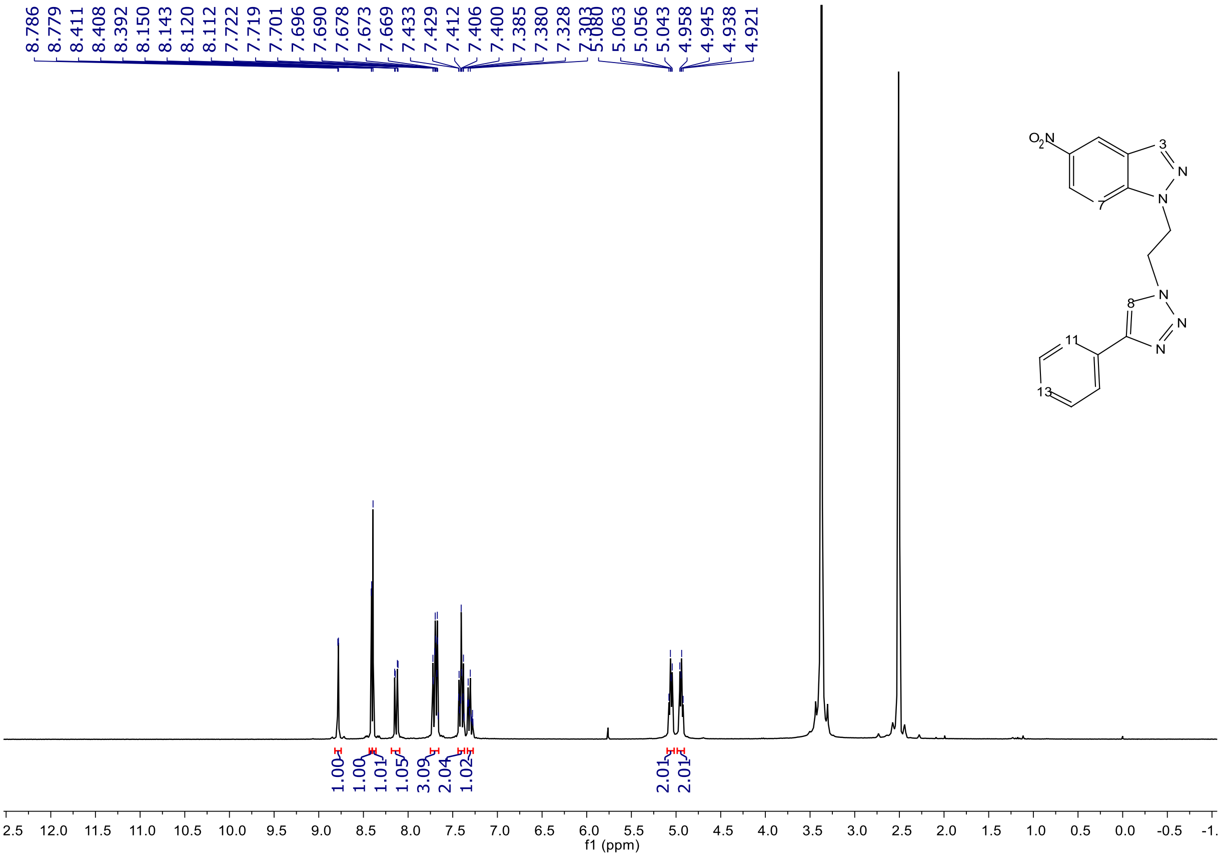

Figure S72. ^{1}H NMR spectrum of compound **7a** in DMSO-d_6.

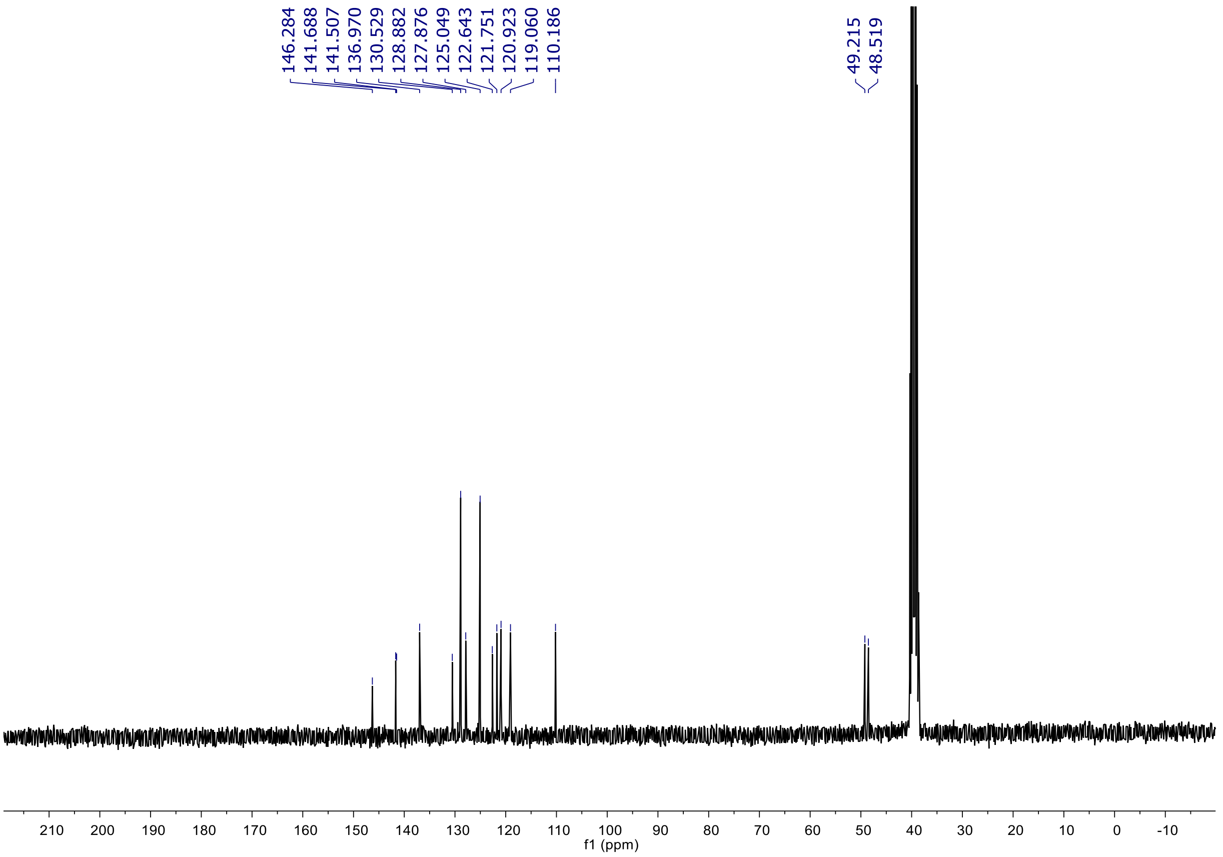

Figure S73. ^{13}C NMR spectrum of compound **7a** in DMSO-d_6.

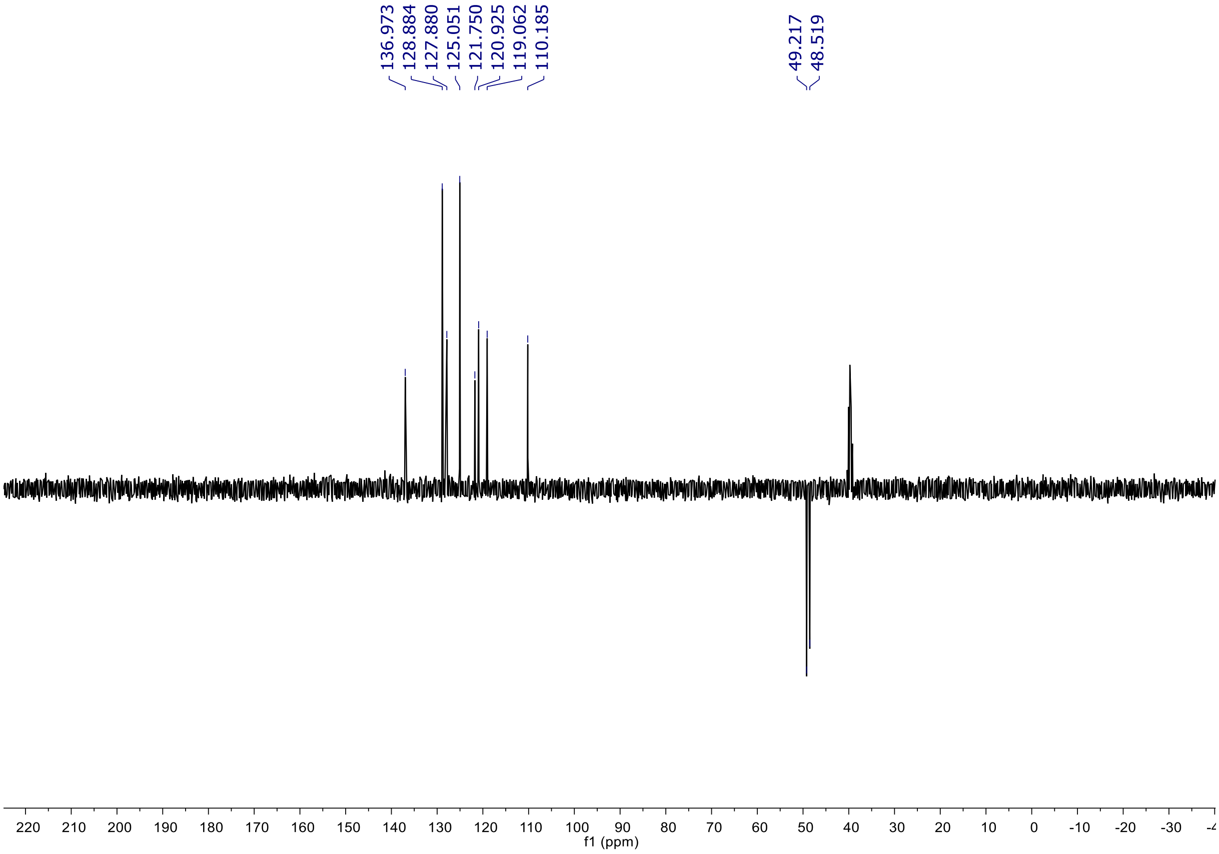

Figure S74. ^{13}C NMR DEPT 135 spectrum of compound **7a** in DMSO-d6.

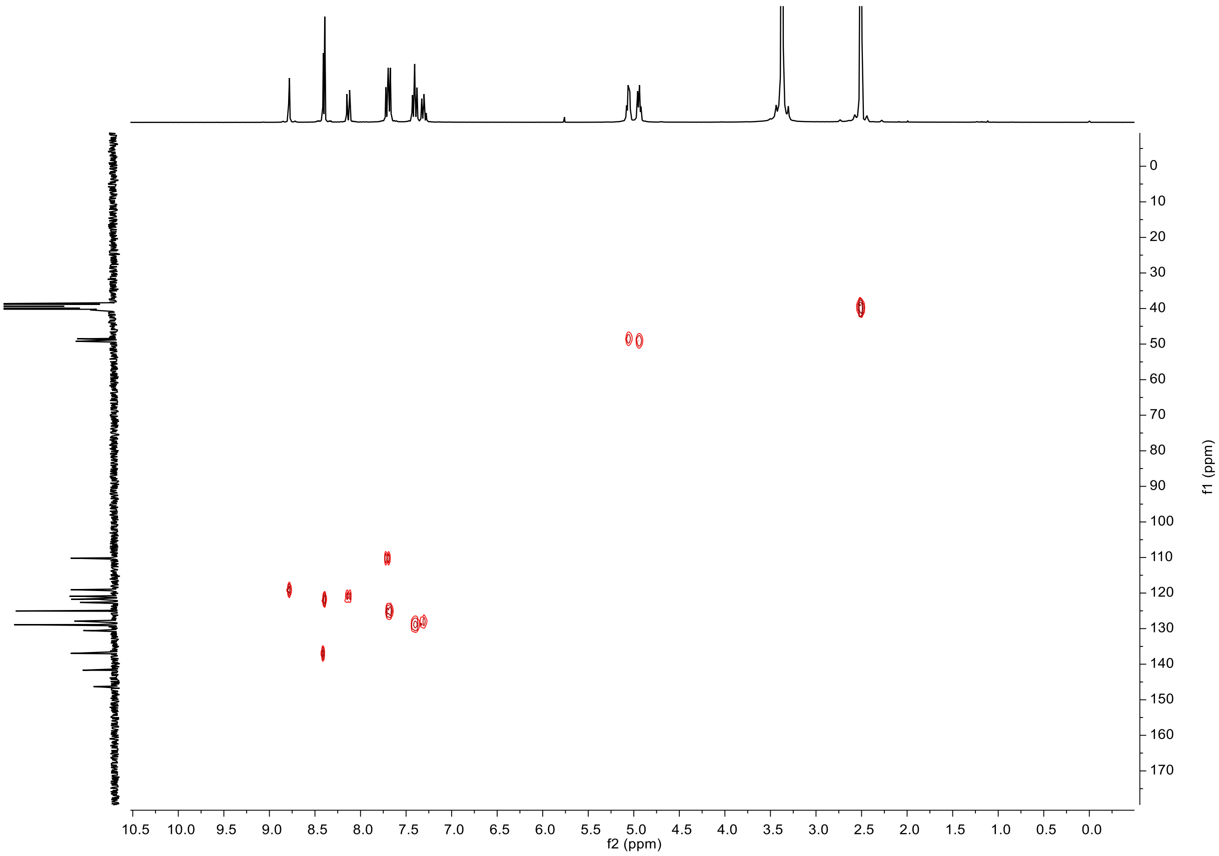

Figure S75. HSQC ($^{1}H/^{13}C$) spectrum of compound **7a** in DMSO-d6.

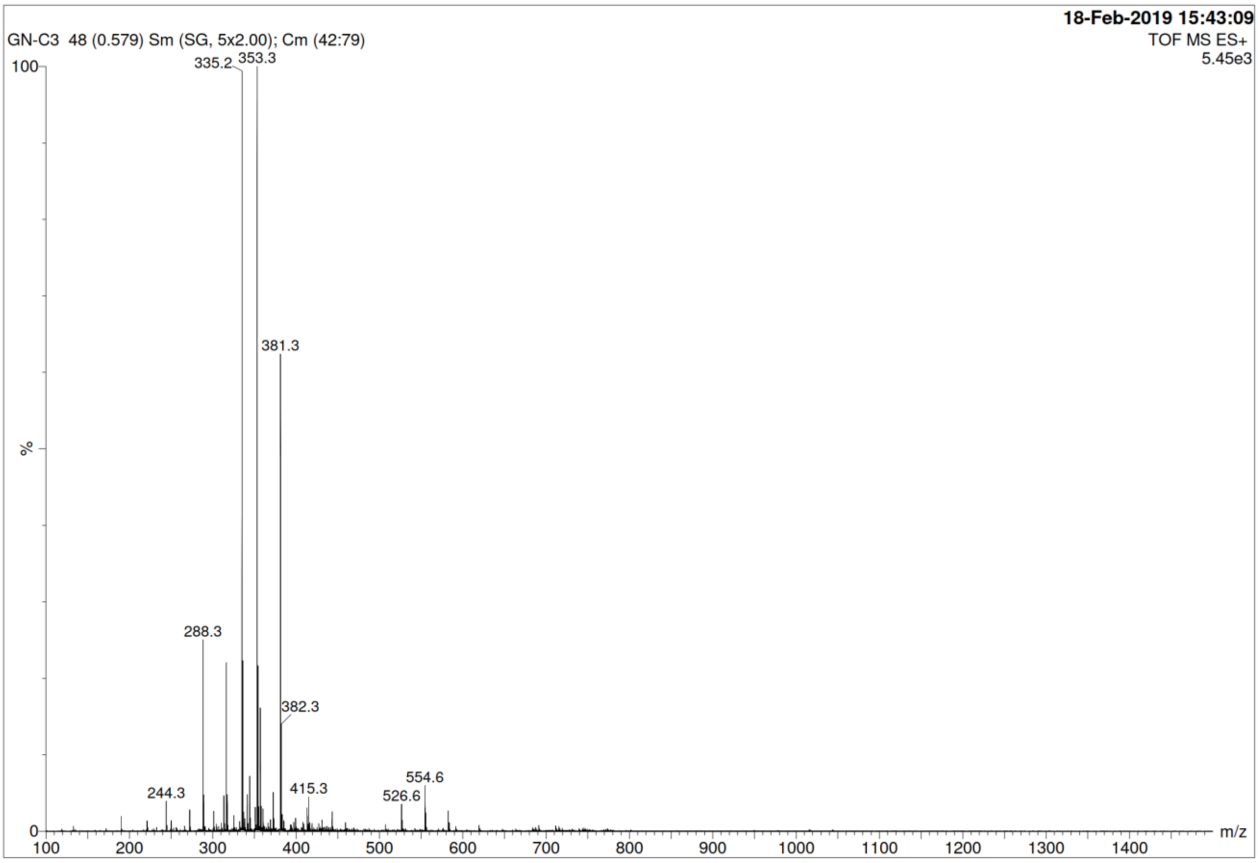

Figure S76. MS-ESI(+) spectrum of compound**7a**.

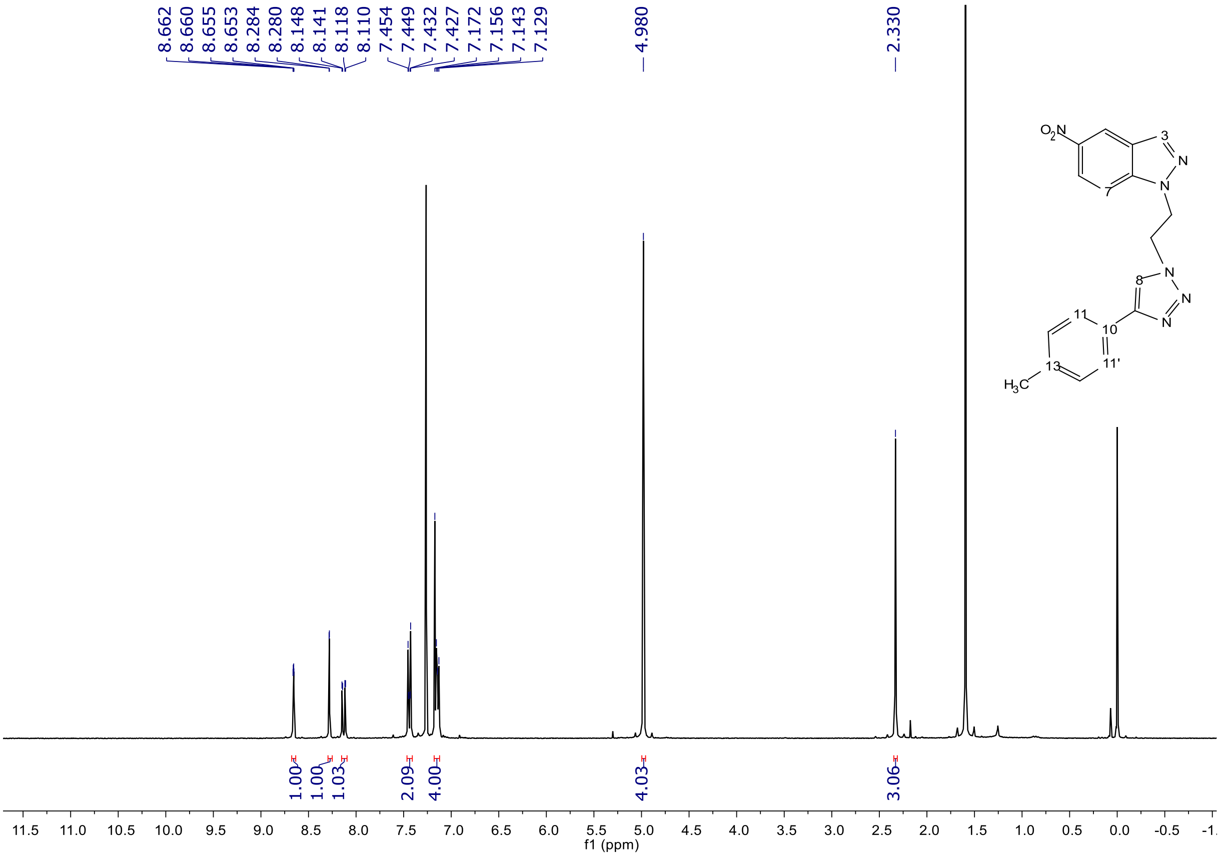

Figure S77. ^{1}H NMR spectrum of compound **7b** in $CDCl_3$.

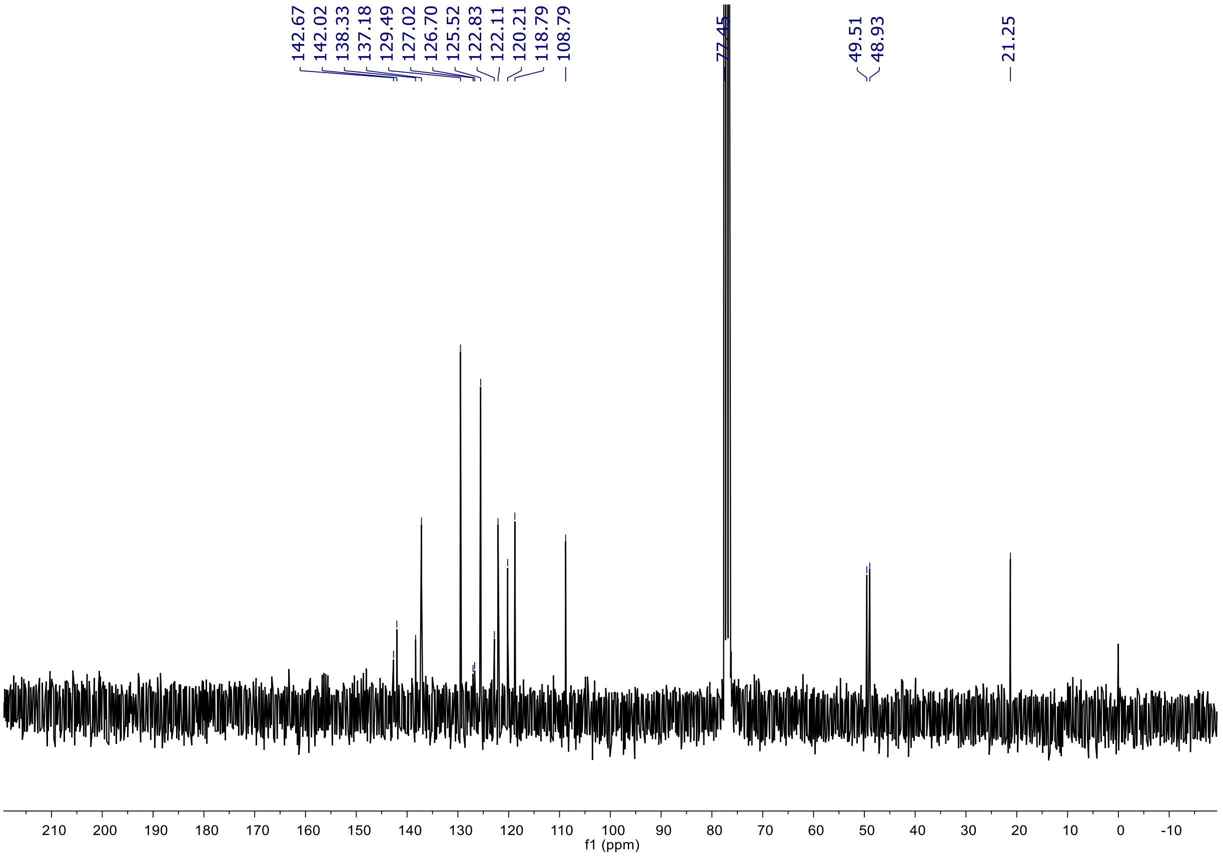

Figure S78. ^{13}C NMR spectrum of compound **7b** in $CDCl_3$.

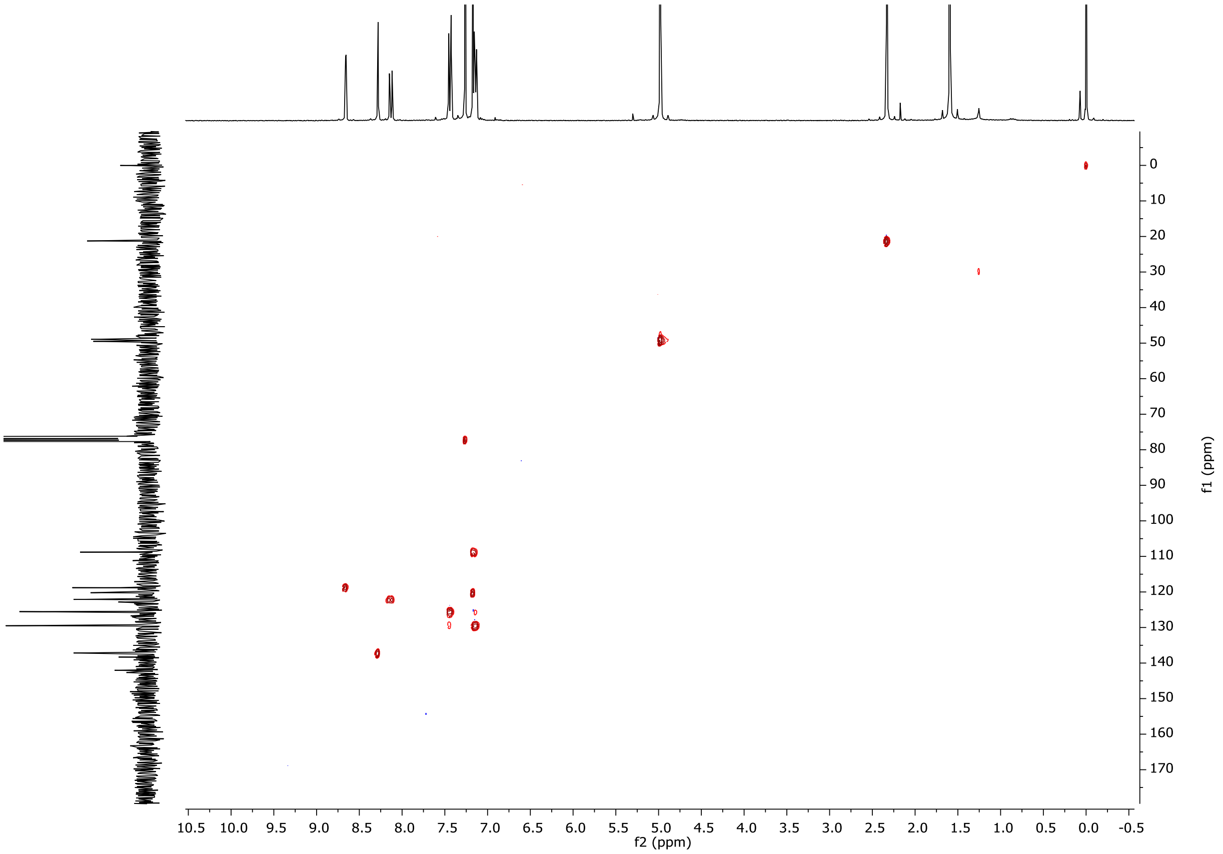

Figure S79. HSQC (^{1}H/^{13}C) spectrum of compound **7b** in $CDCl_3$.

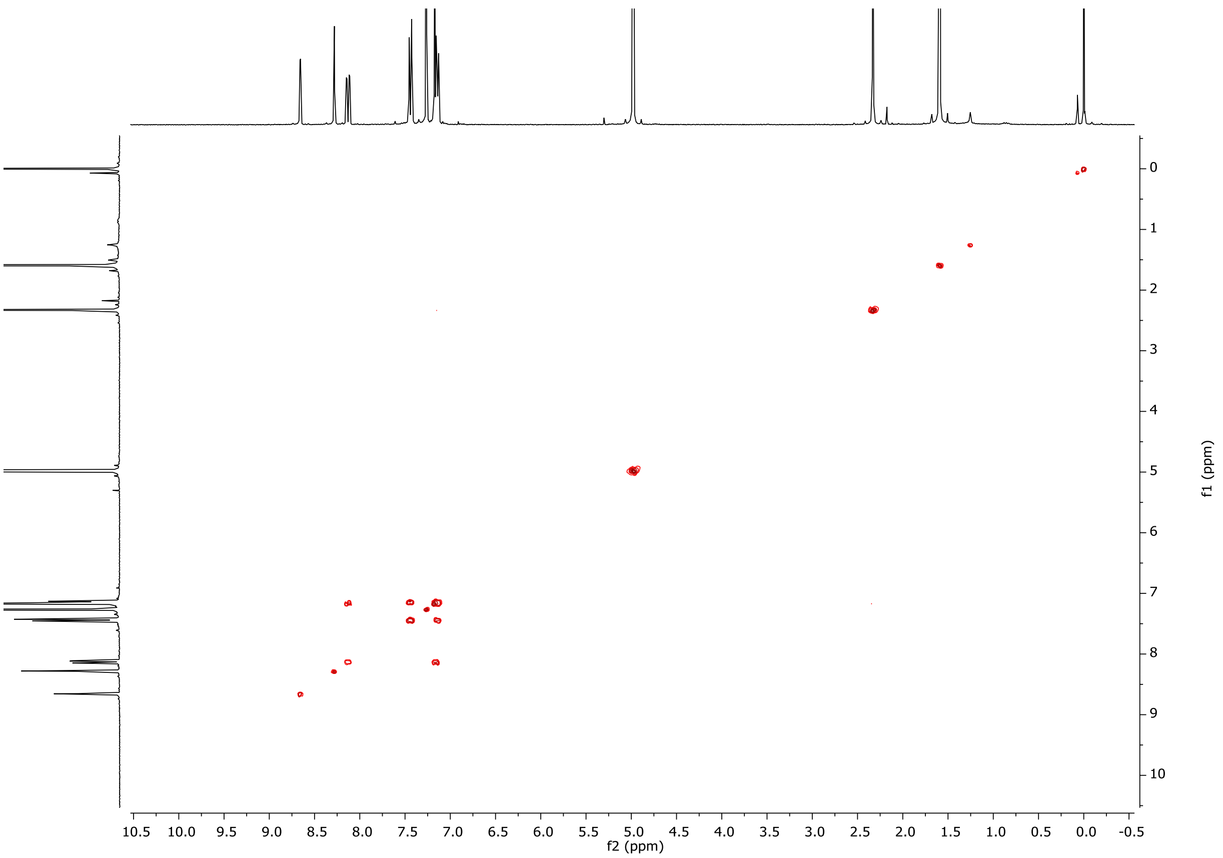

Figure S80. COSY ($^1H/^1H$) spectrum of compound **7b** in $CDCl_3$.

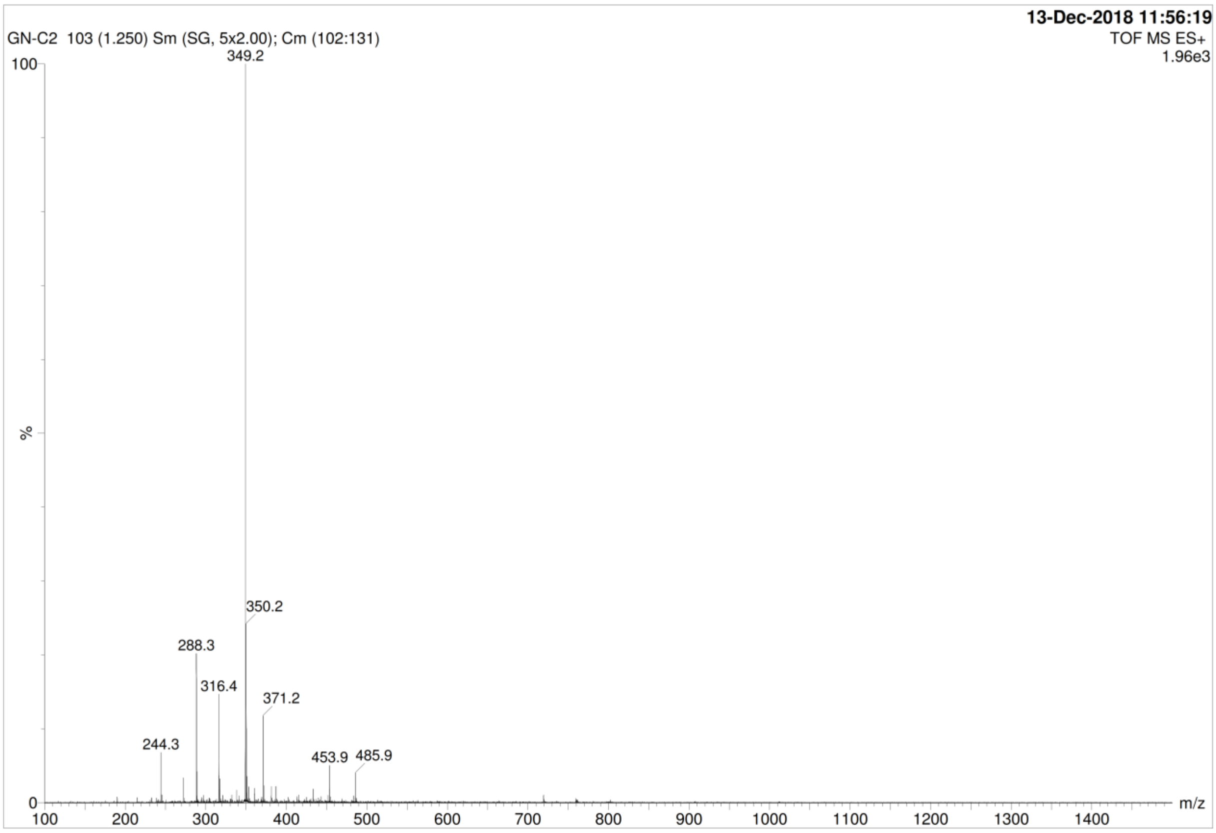

Figure S81. MS-ESI(+) spectrum of compound **7b**.

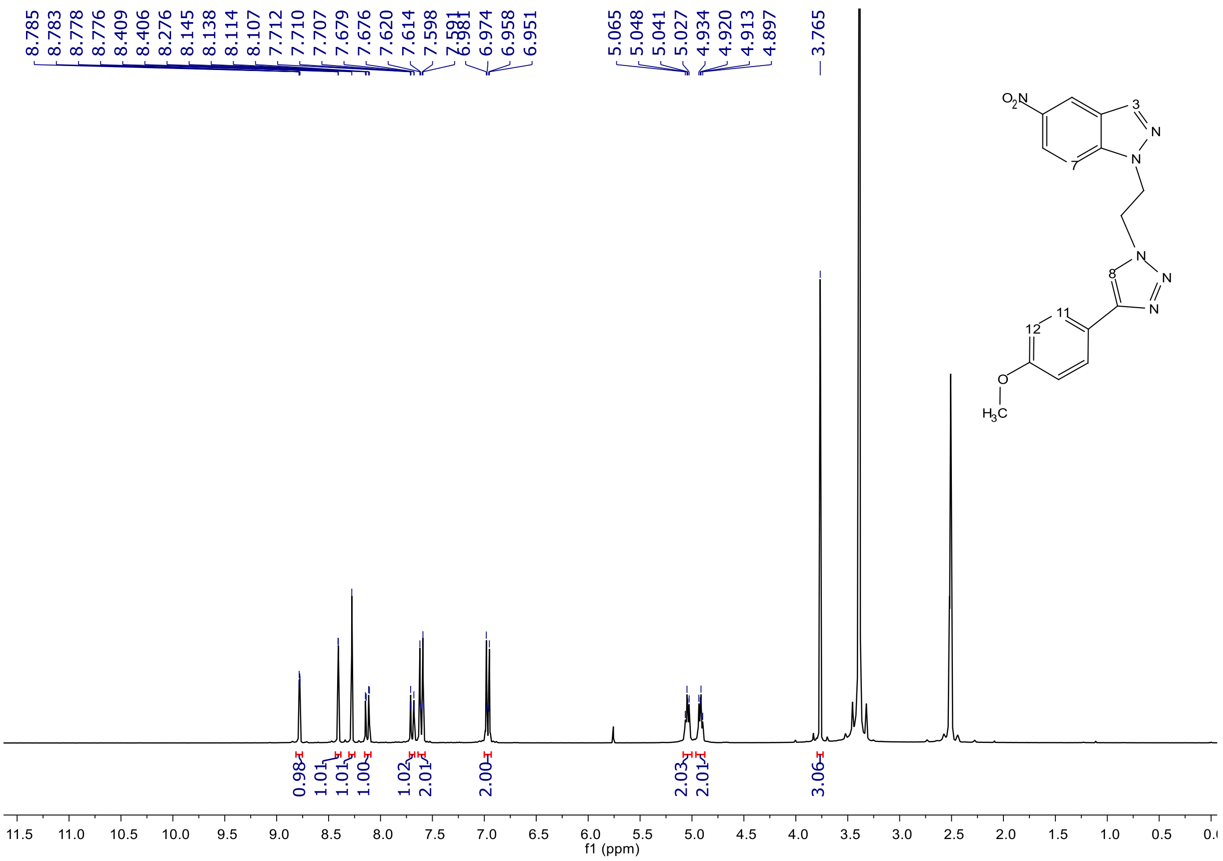

Figure S82. ^{1}H NMR spectrum of compound **7c** in DMSO-d_6.

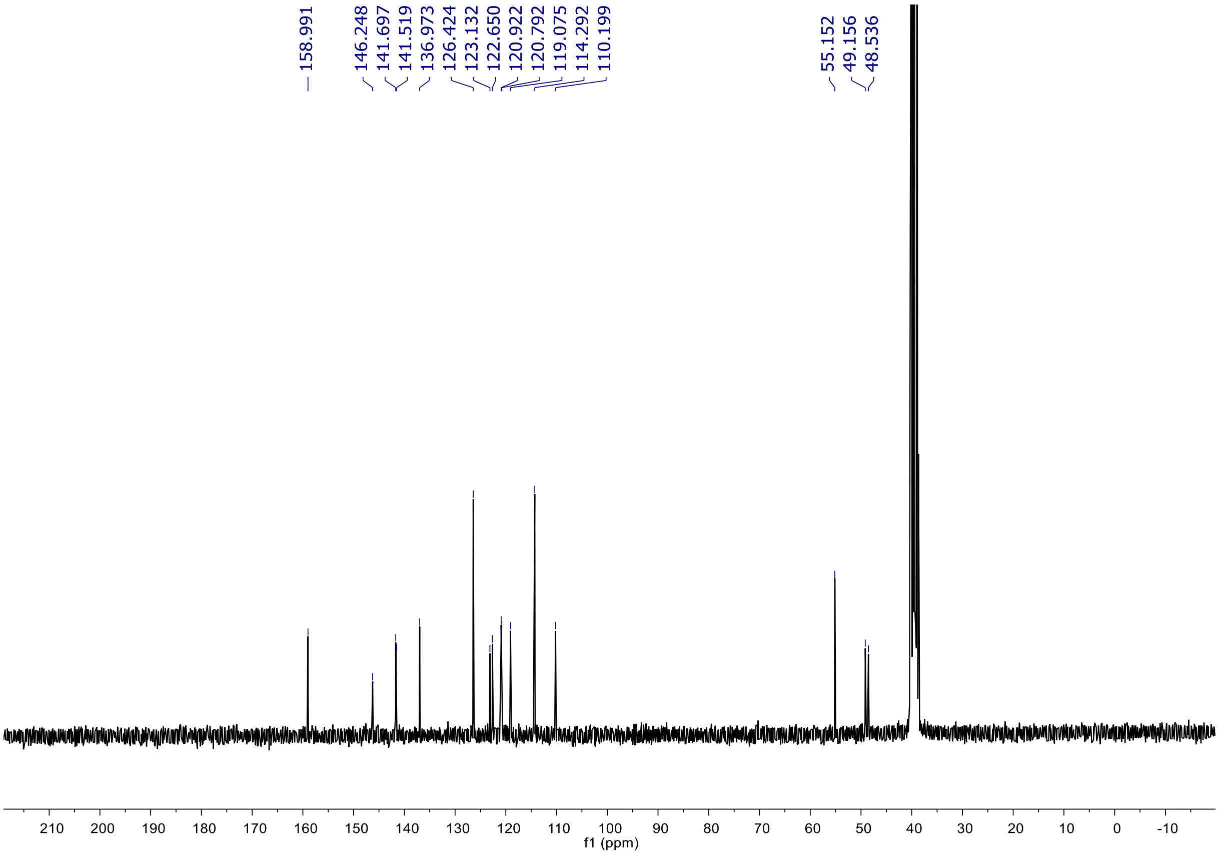

Figure S83. ^{13}C NMR spectrum of compound **7c** in DMSO-d_6.

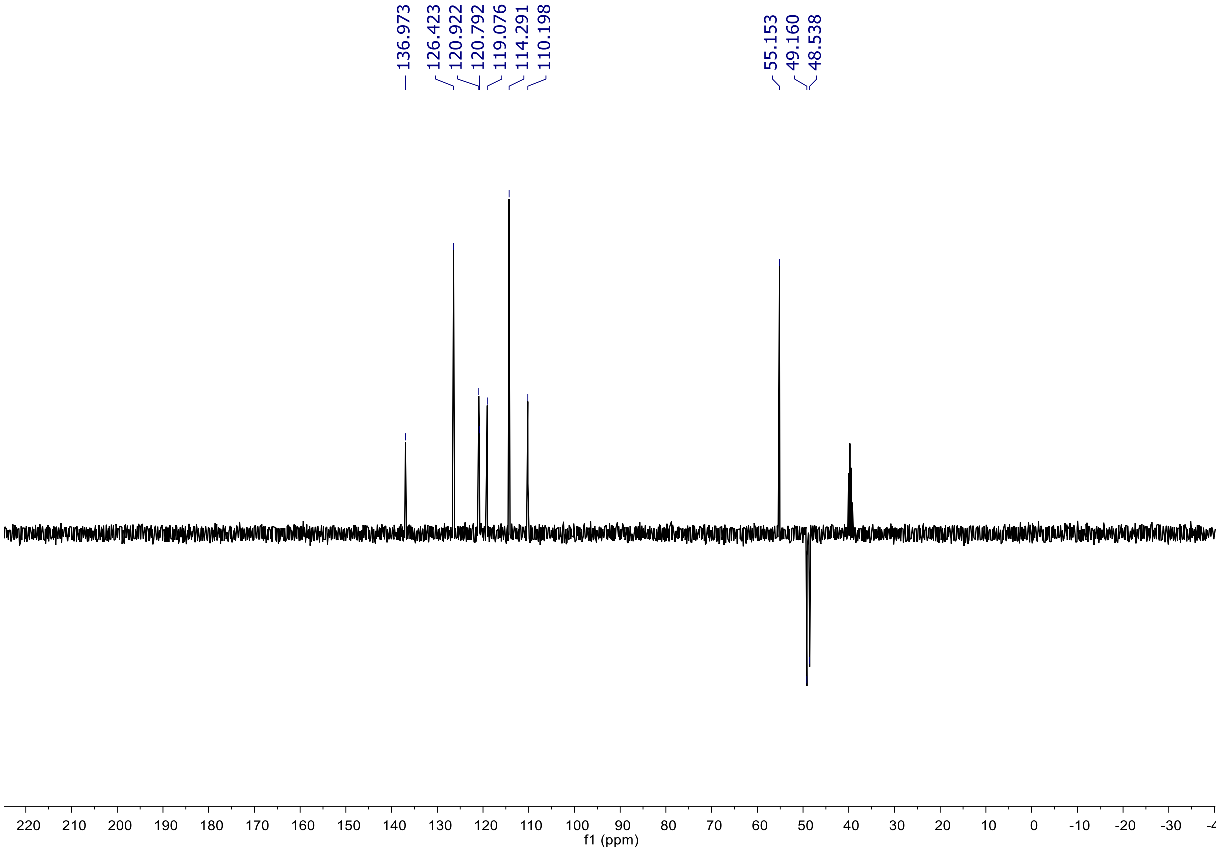

Figure S84. ^{13}C NMR DEPT 135 spectrum of compound **7c** in DMSO-d_6.

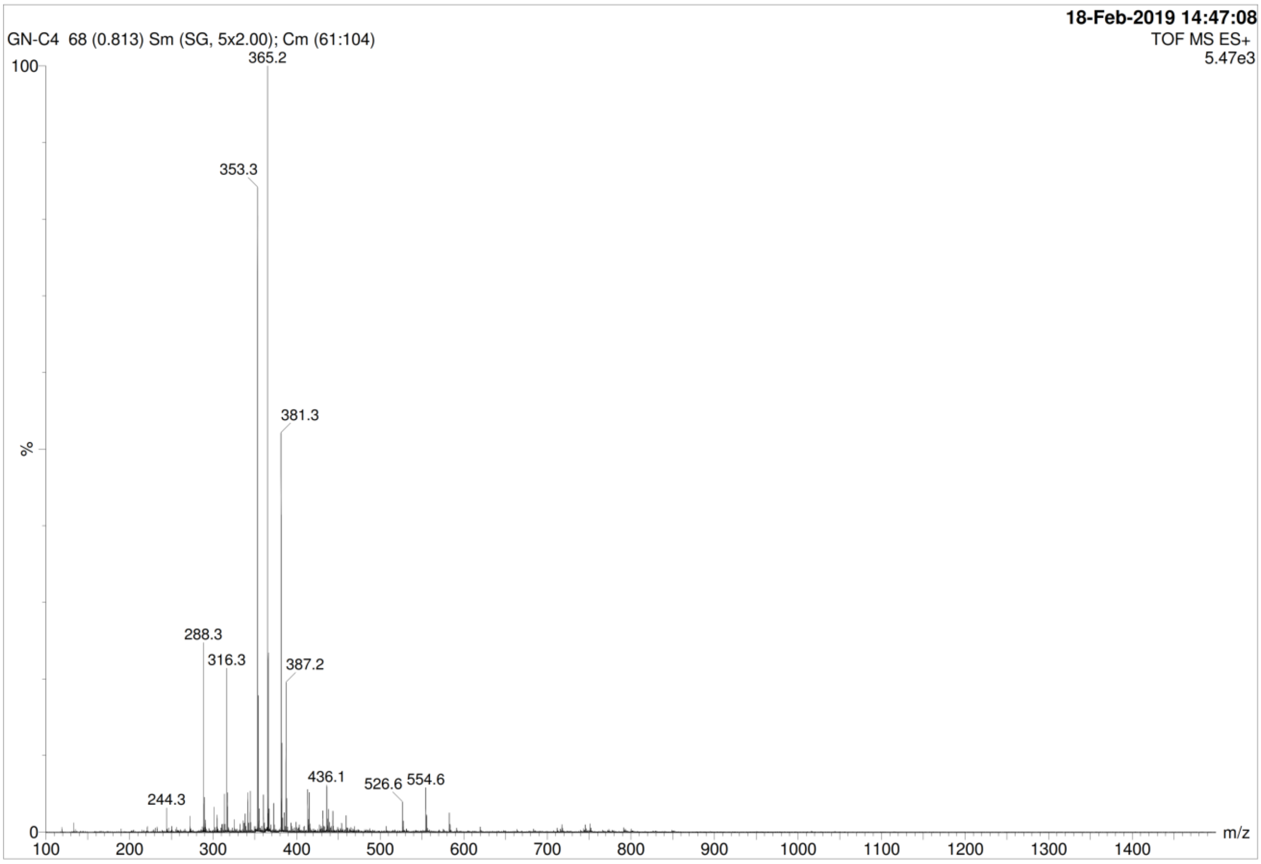

Figure S85. MS-ESI(+) spectrum of compound **7c**.

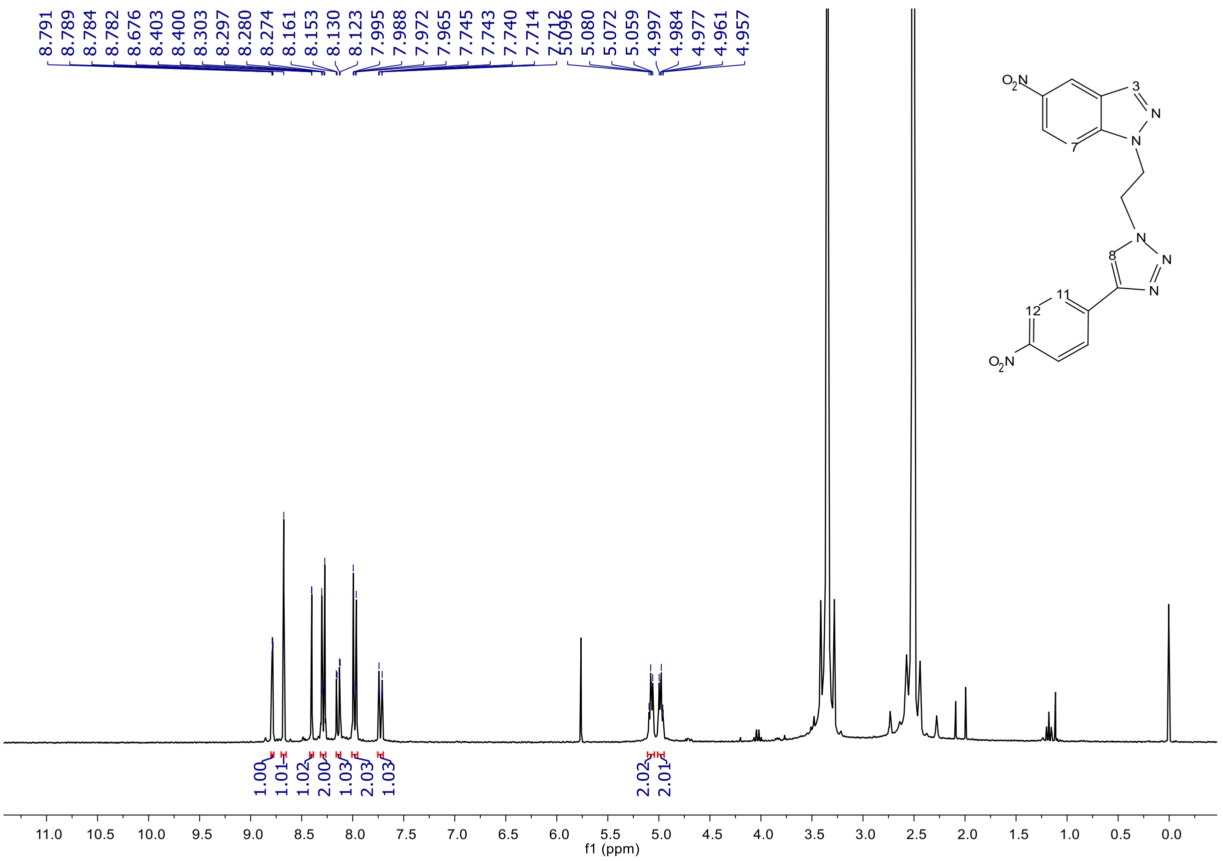

Figure S86. ^{1}H NMR spectrum of compound **7d** in DMSO-d6.

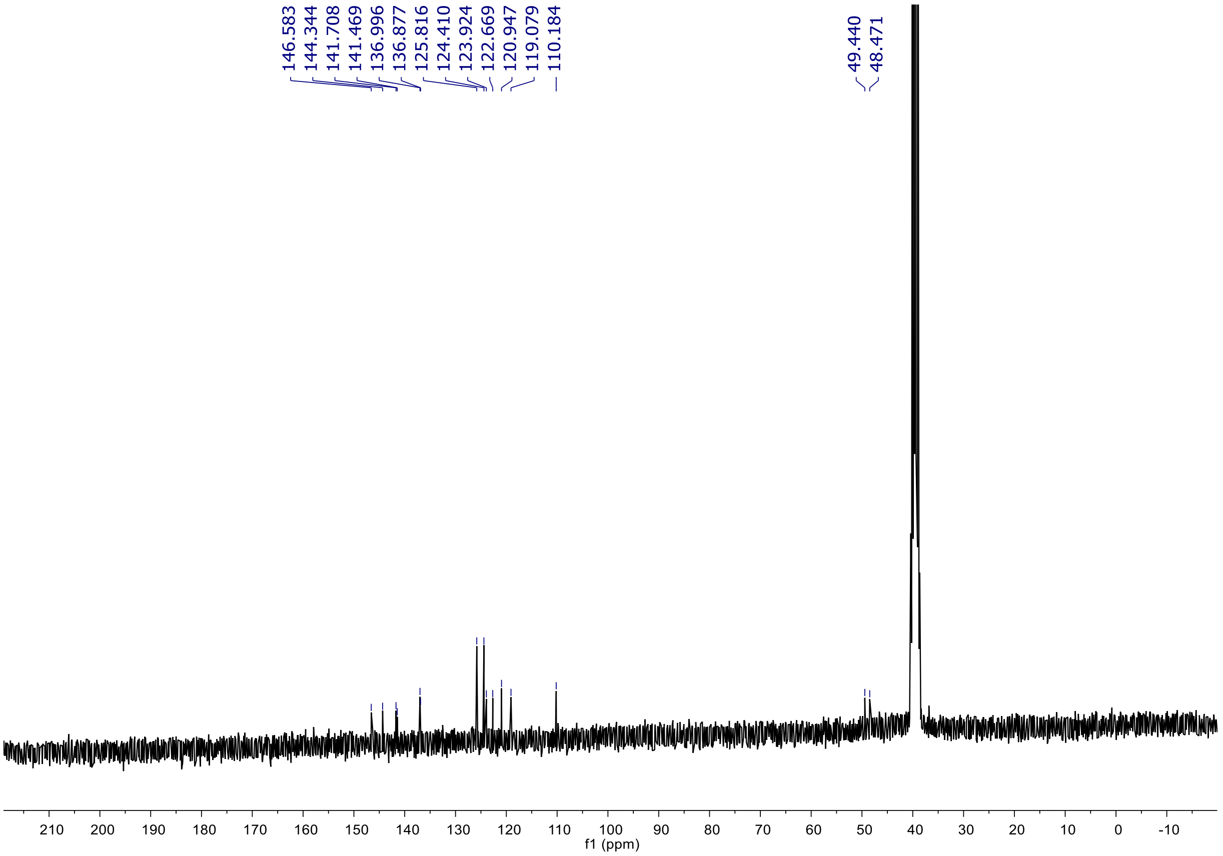

Figure S87. ^{13}C NMR spectrum of compound **7d** in DMSO-d6.

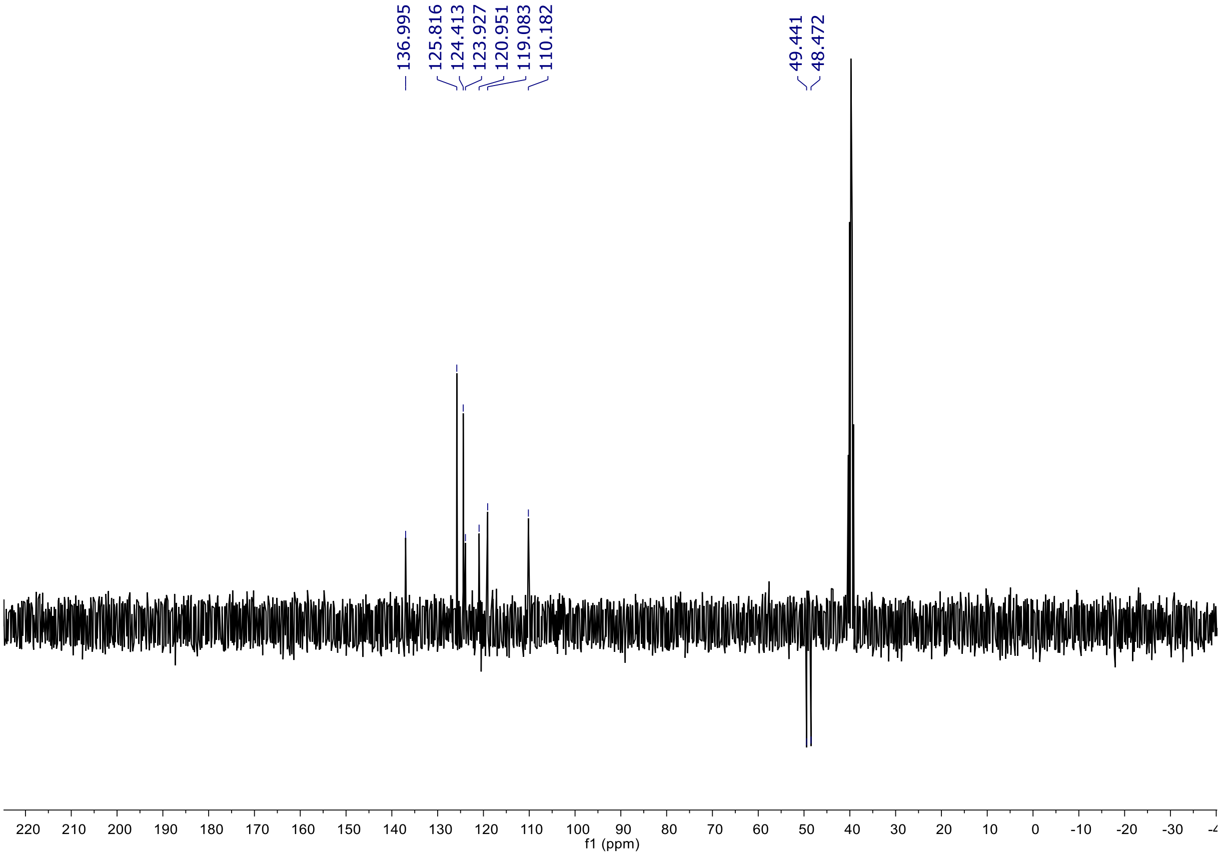

Figure S88. ^{13}C NMR DEPT 135 spectrum of compound **7d** in DMSO-d_6.

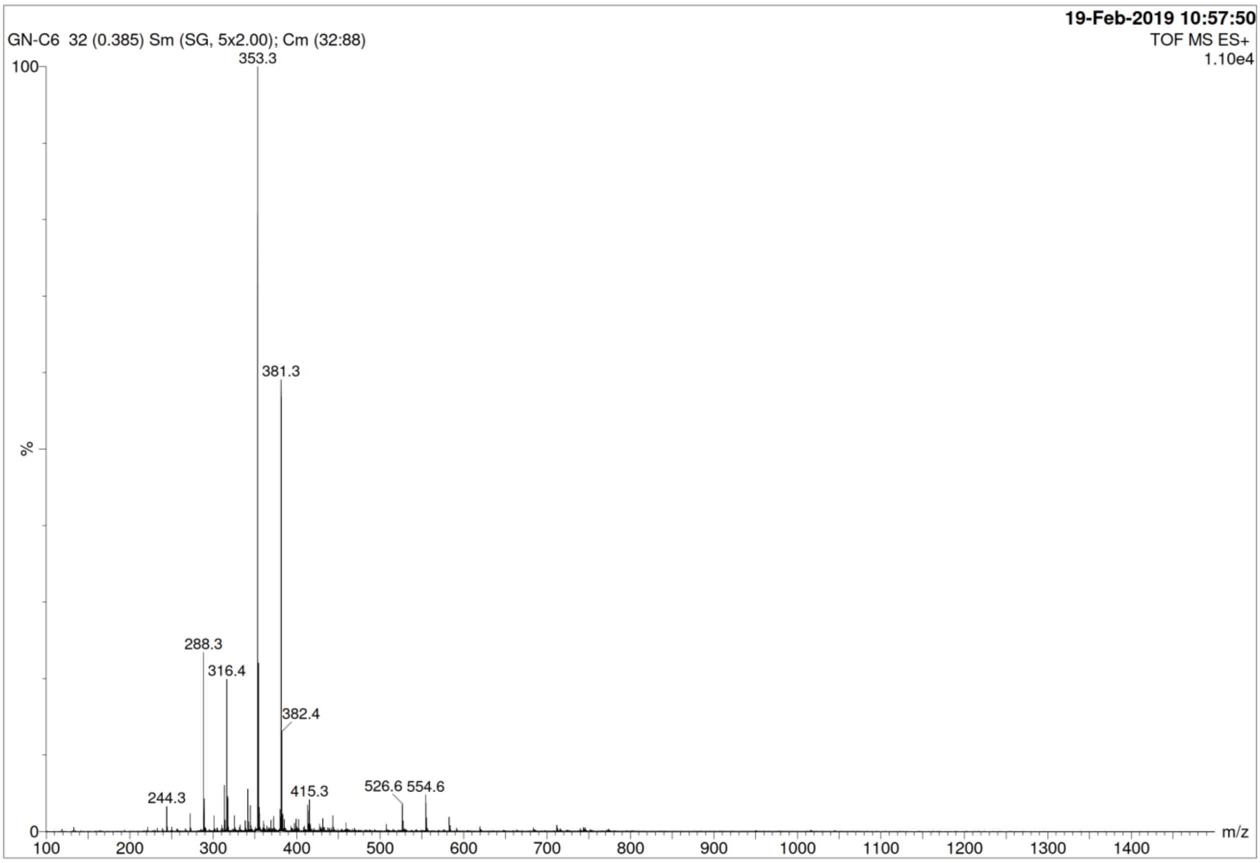

Figure S89. MS-ESI(+) spectrum of compound **7d**.

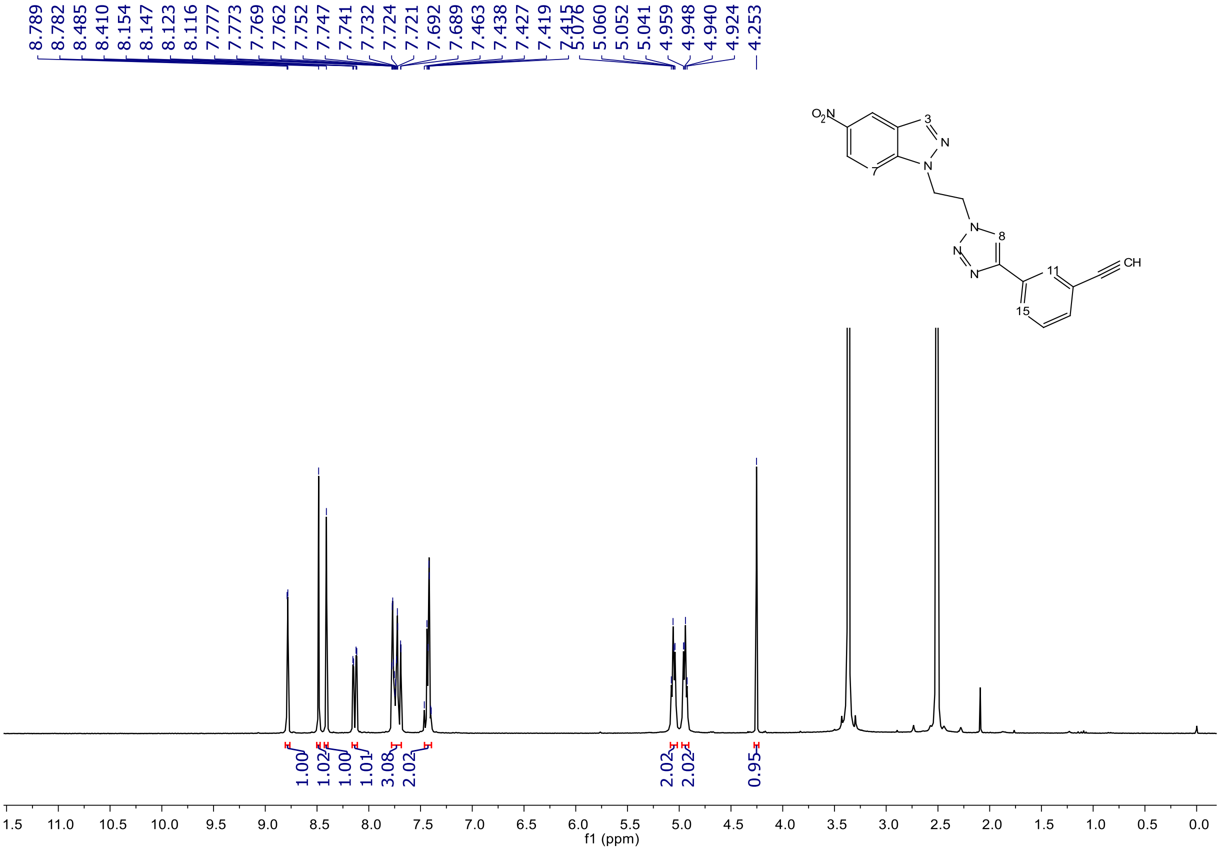

Figure S90. ^{1}H NMR spectrum of compound **8** in DMSO-d$_6$.

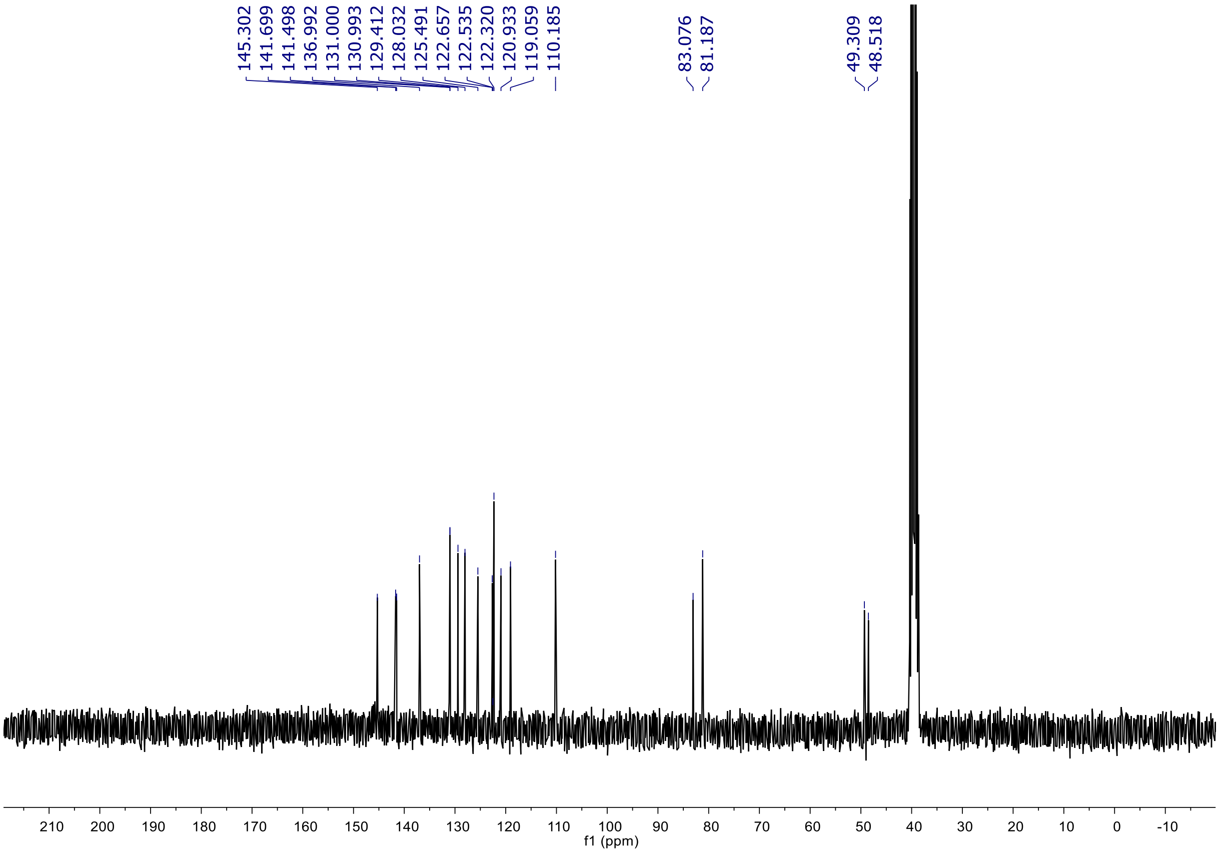

Figure S91. ^{13}C NMR spectrum of compound **8** in DMSO-d$_6$.

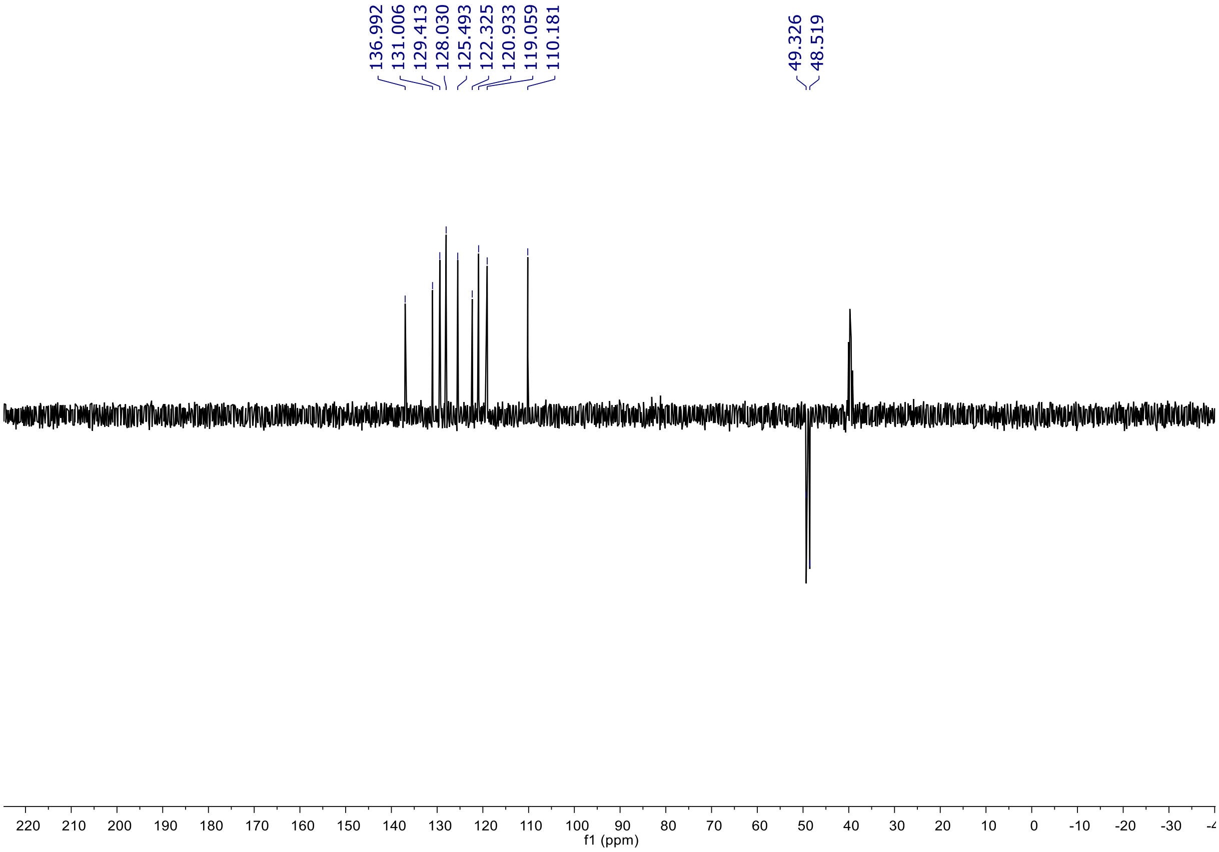

Figure S92. ^{13}C NMR DEPT 135 spectrum of compound **8** in DMSO-d_6.

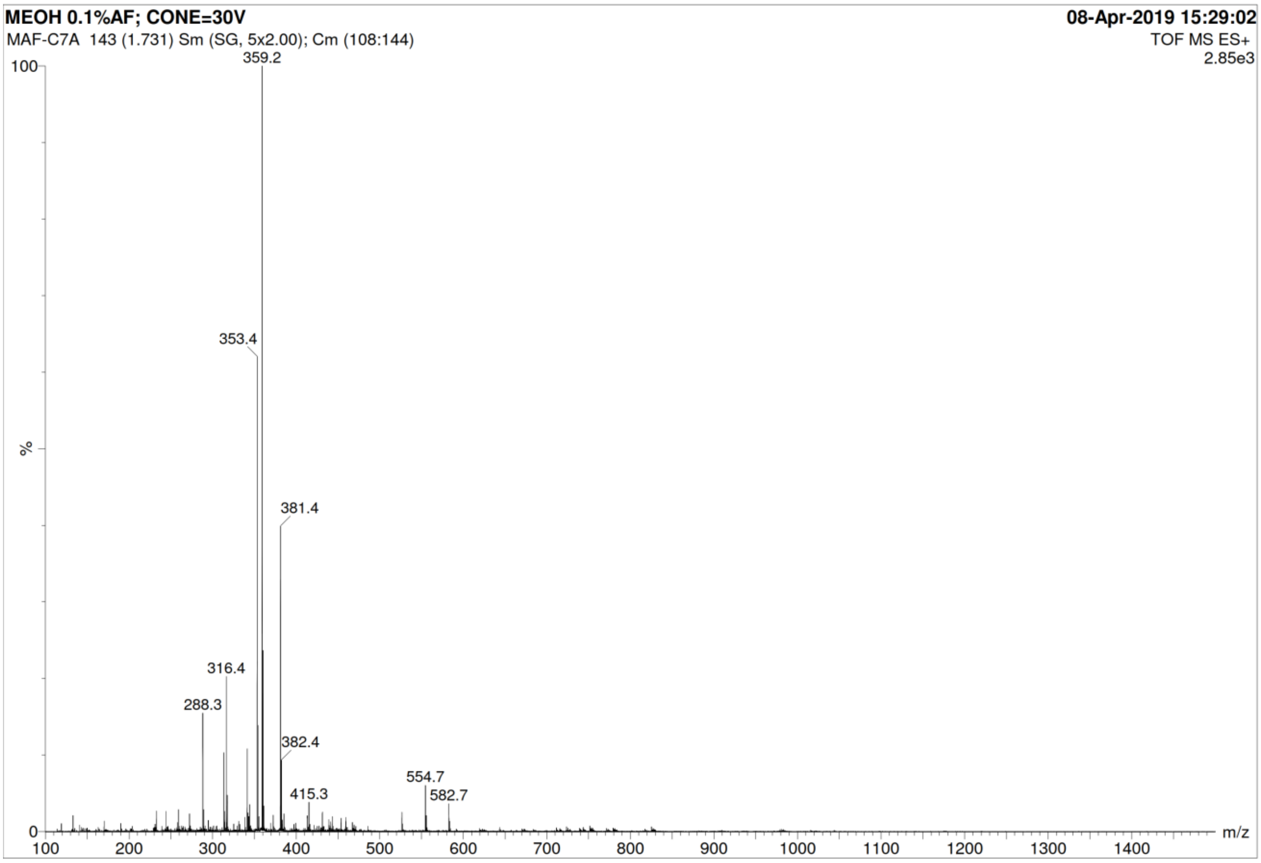

Figure S93. MS-ESI(+) spectrum of compound **8**.

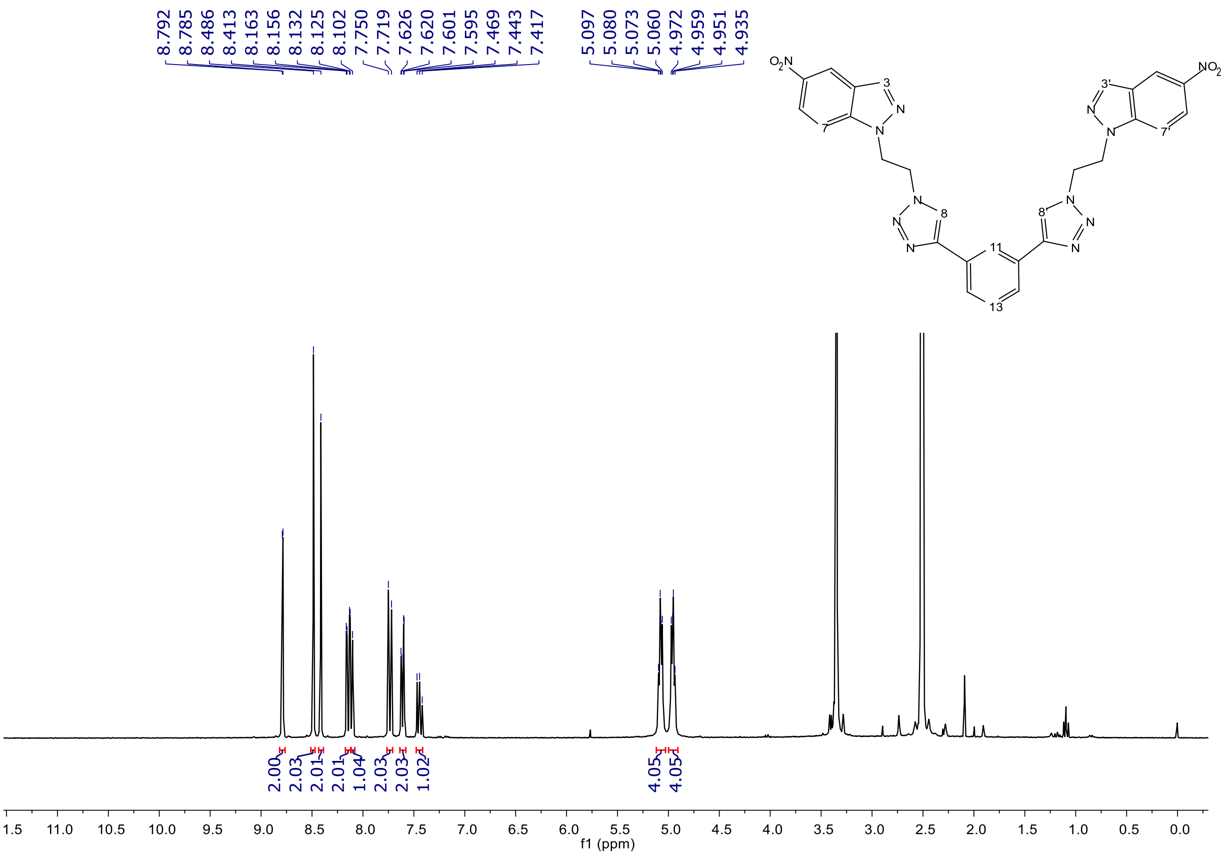

Figure S94. ^{1}H NMR spectrum of compound **9** in DMSO-d$_6$.

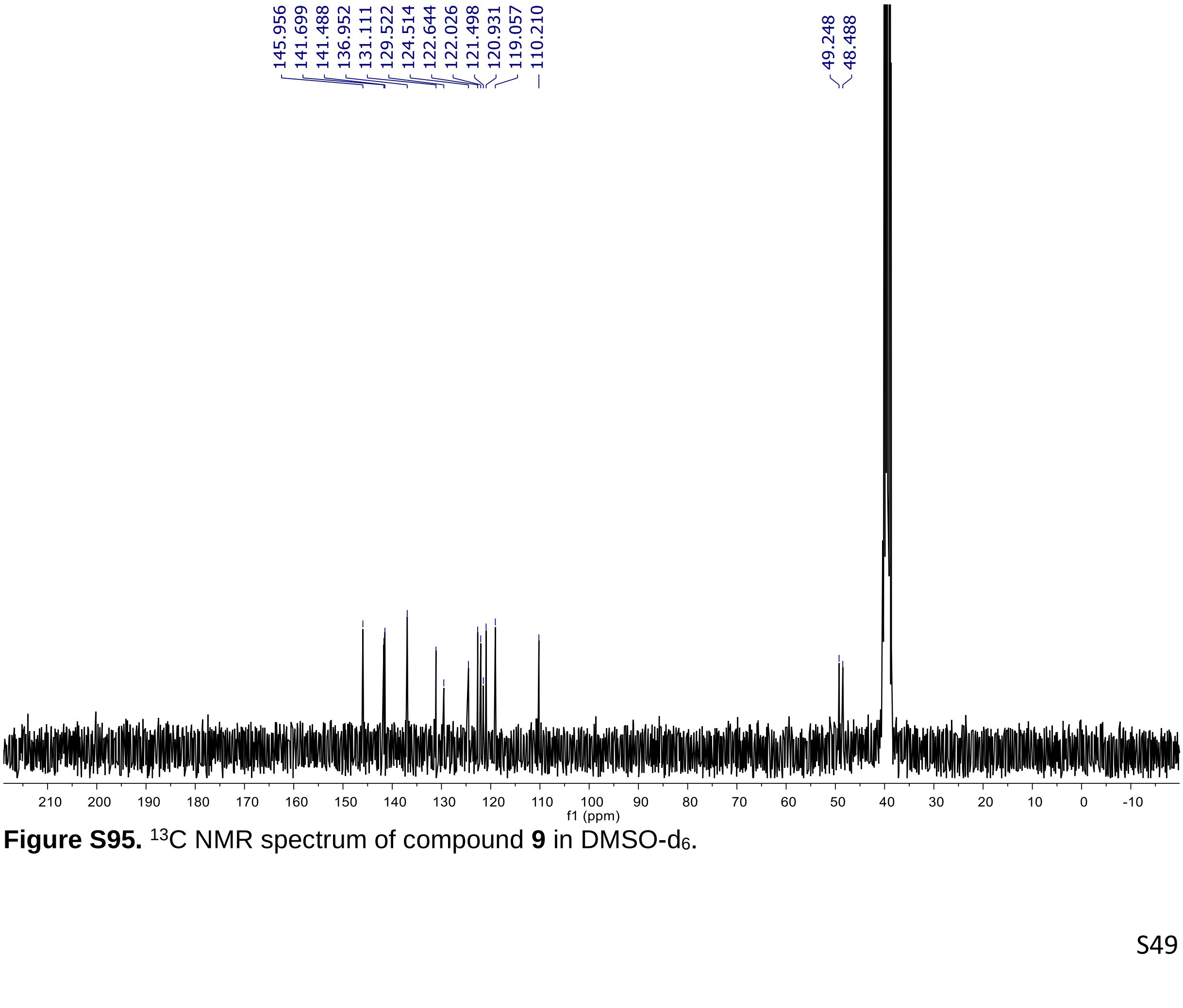

Figure S95. ^{13}C NMR spectrum of compound **9** in DMSO-d$_6$.

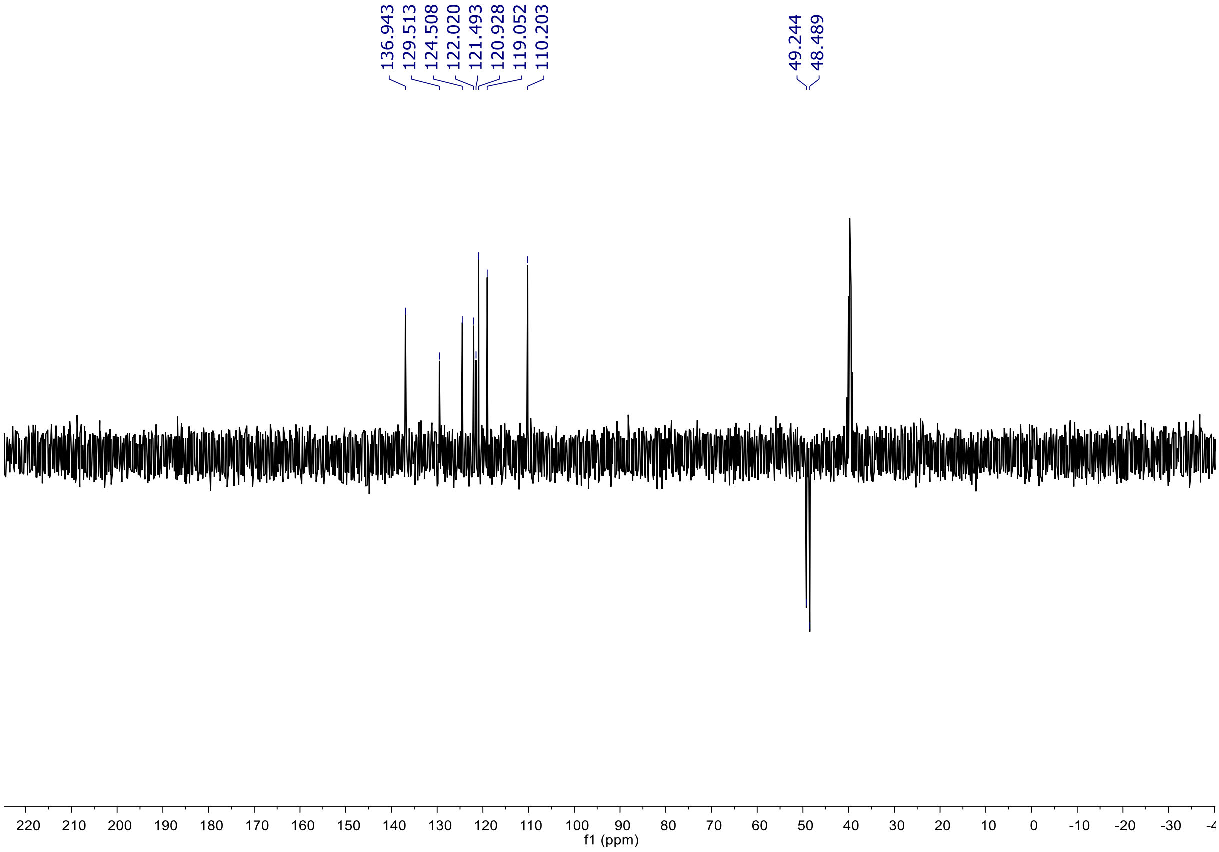

Figure S96. ^{13}C NMR DEPT 135 spectrum of compound **9** in DMSO-d_6.

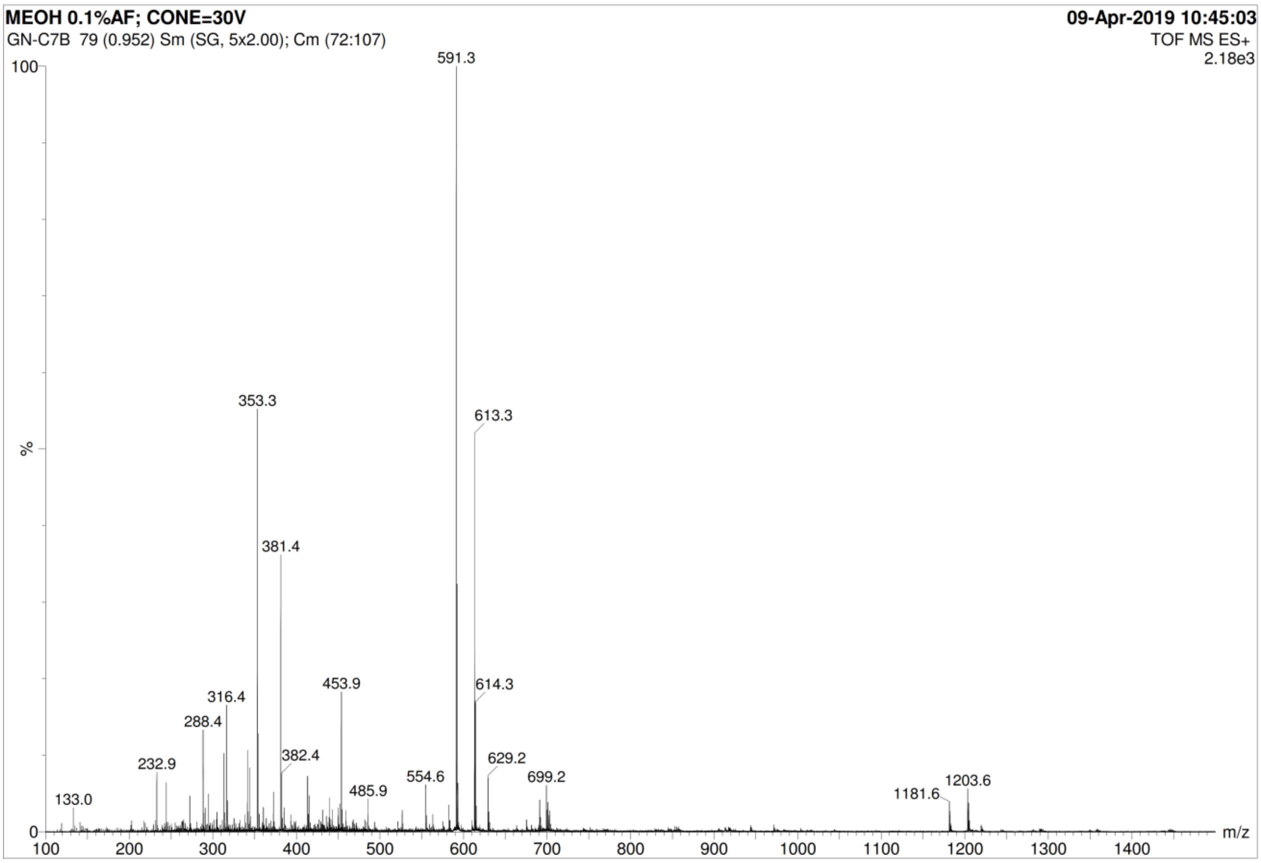

Figure S97. MS-ESI(+) spectrum of compound **9.**

III - NMR and mass spectrum of compounds 12a-e

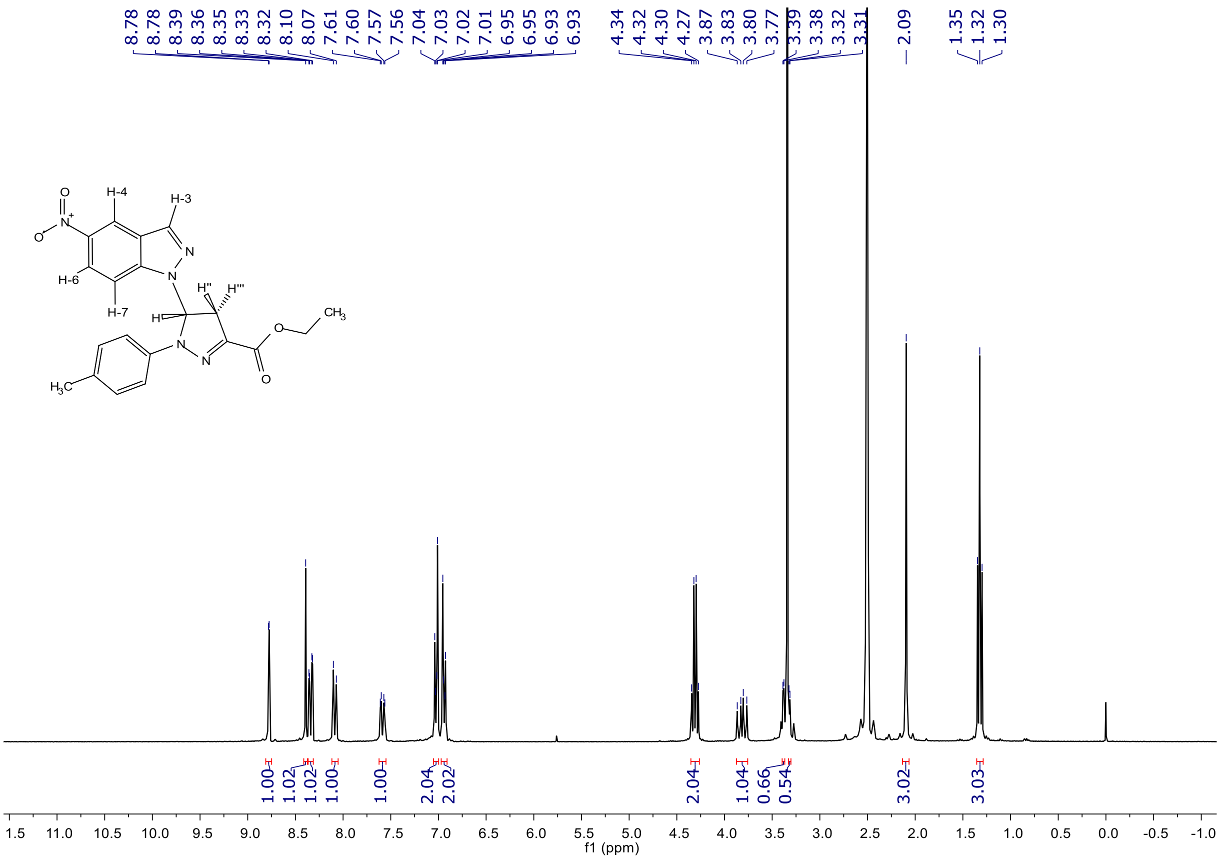

Figure S98. ^{1}H NMR spectrum of compound **12a** in DMSO-d_6.

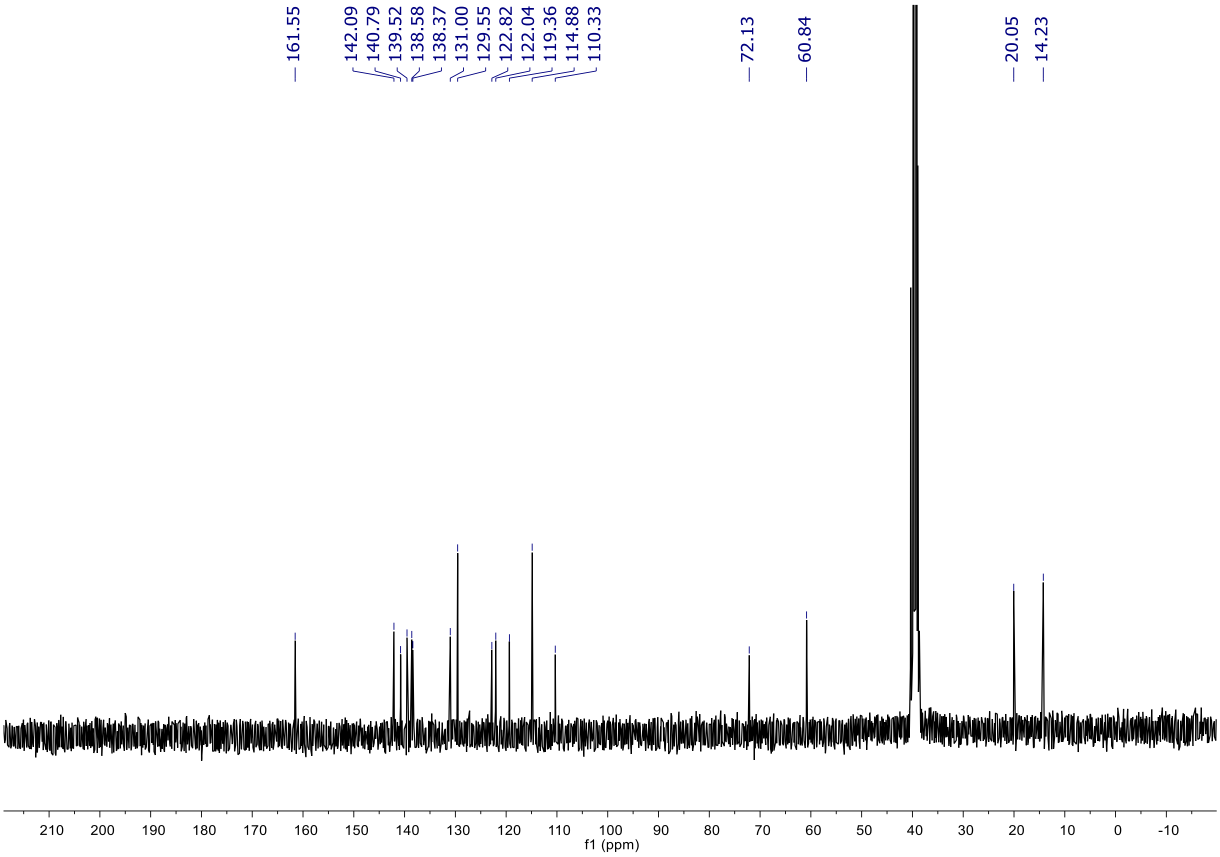

Figure S99. ^{13}C NMR spectrum of compound **12a** in DMSO-d_6.

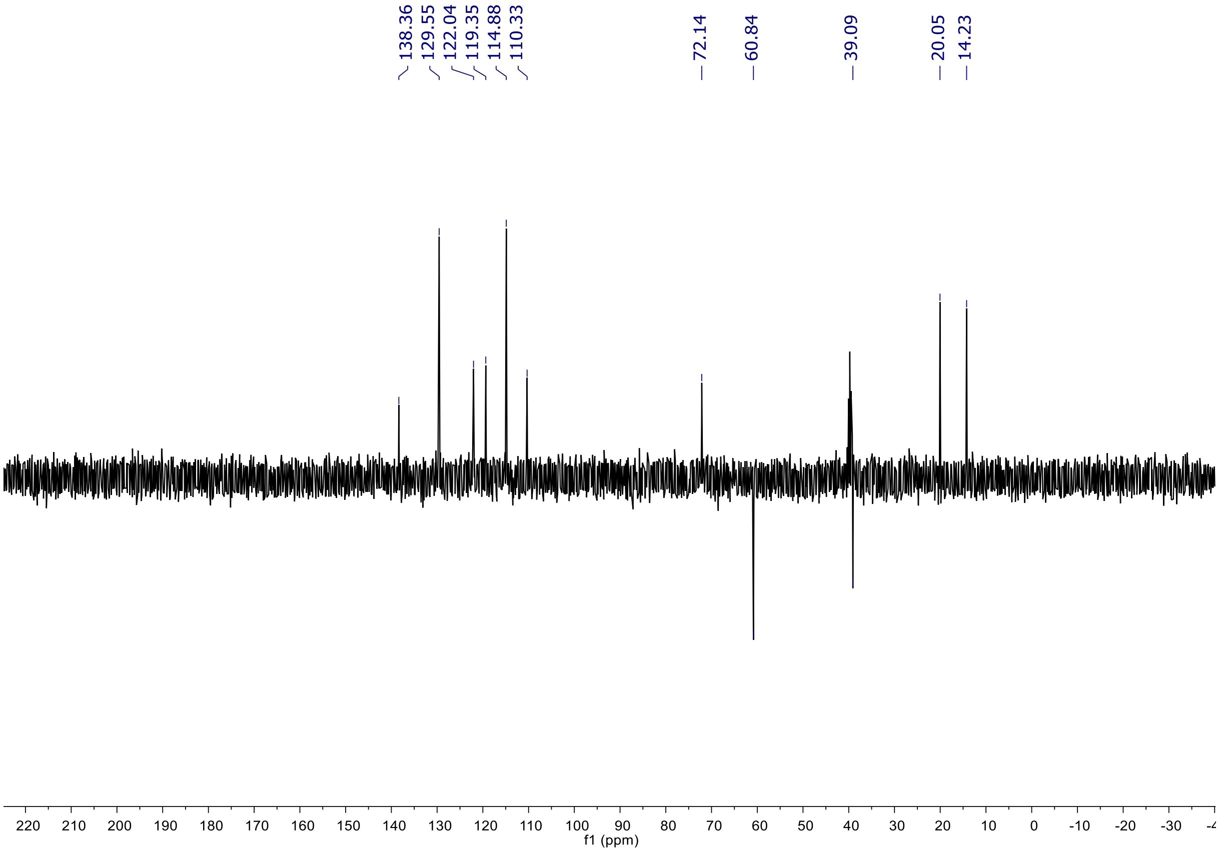

Figure S100. ^{13}C NMR DEPT 135 spectrum of compound **12a** in DMSO-d_6.

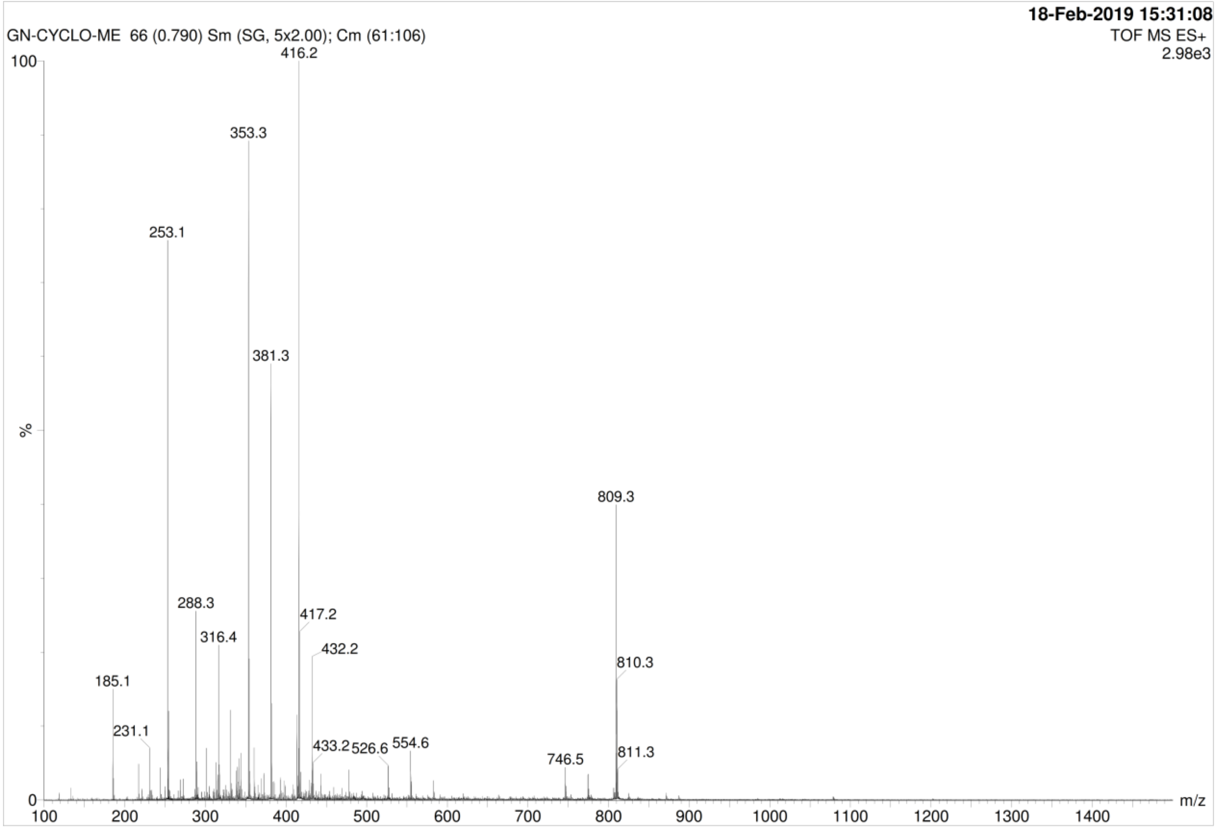

Figure S101. MS-ESI(+) spectrum of compound **12a**.

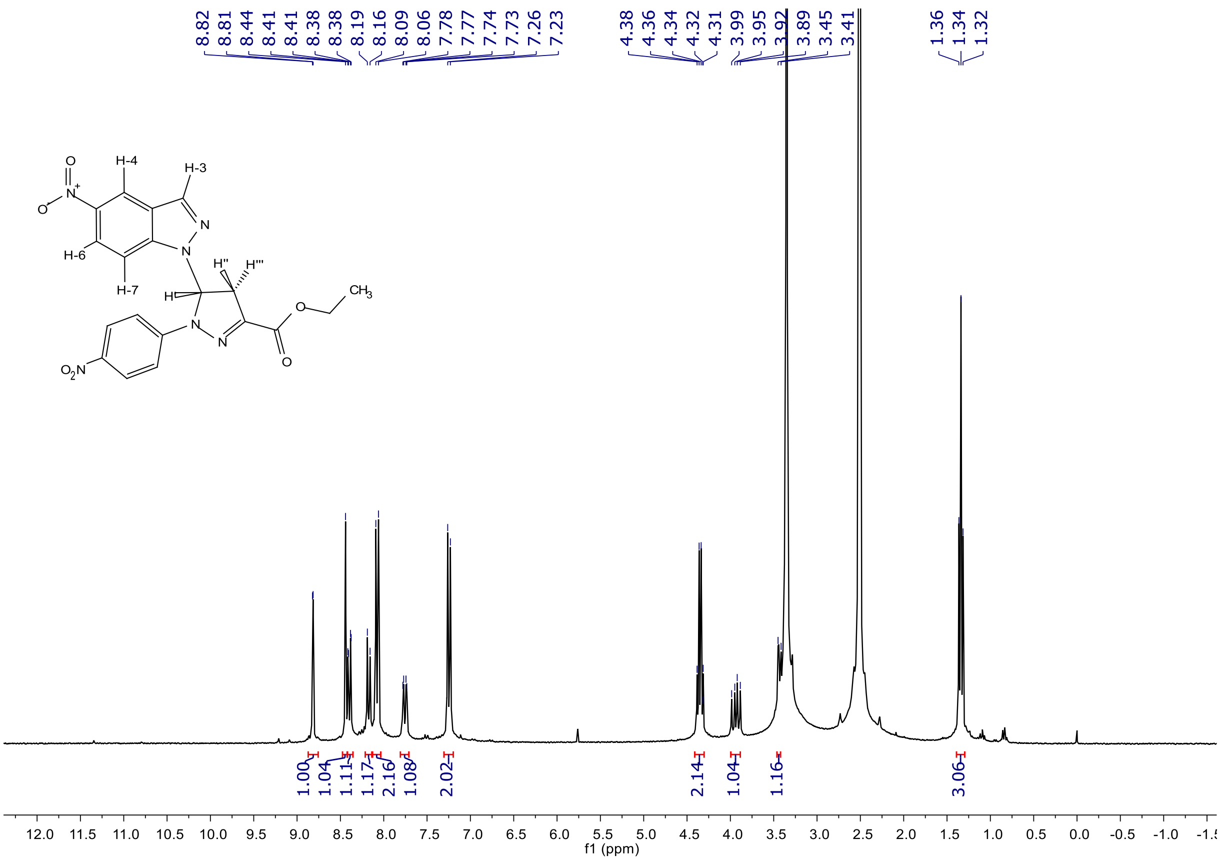

Figure S102. ^{1}H NMR spectrum of compound **12b** in DMSO-d6.

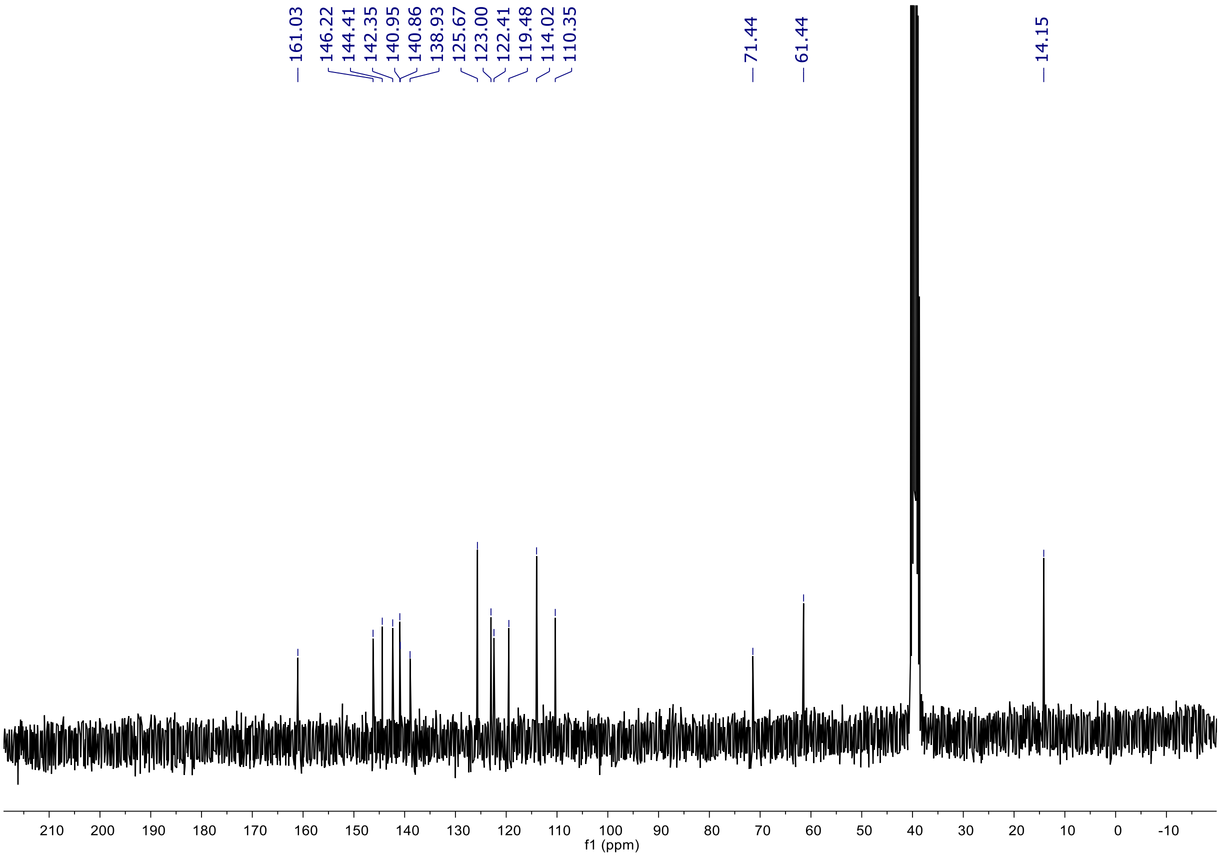

Figure S103. ^{13}C NMR spectrum of compound **12b** in DMSO-d6.

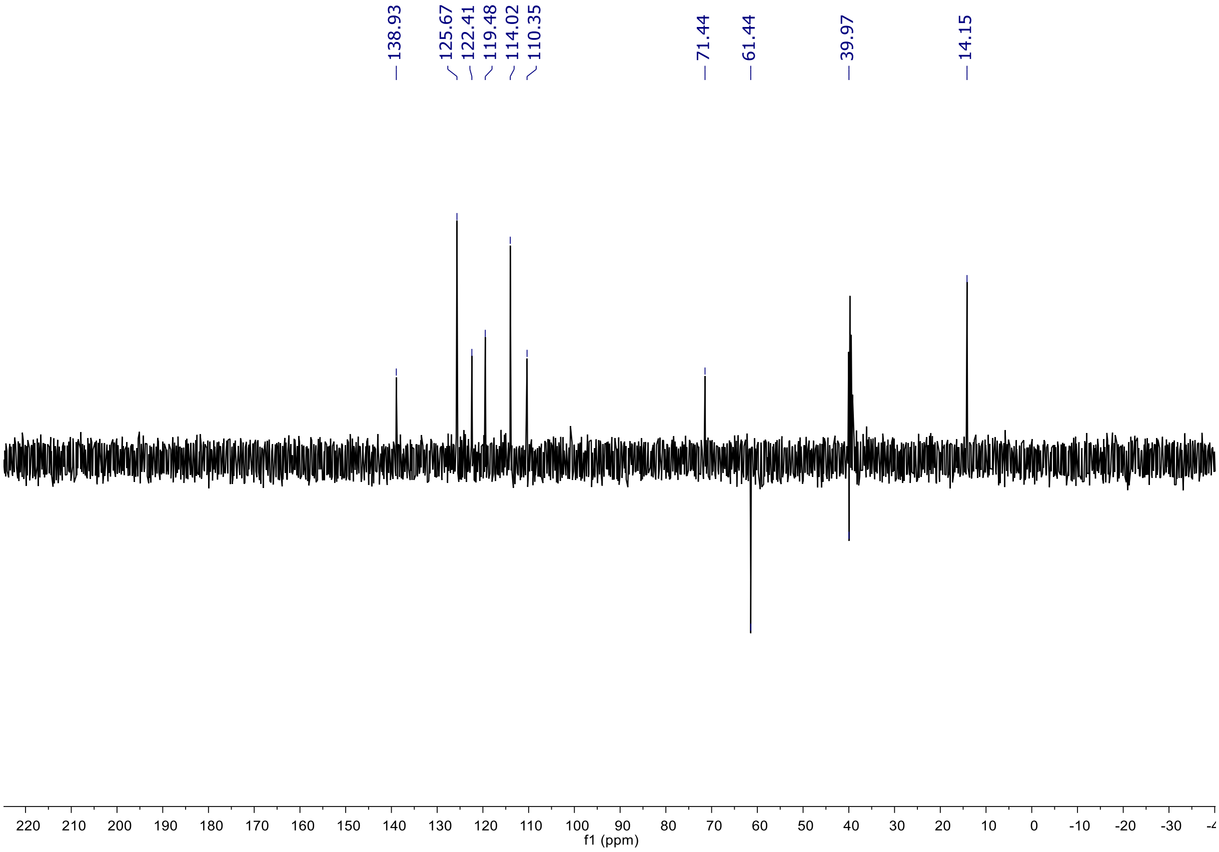

Figure S104. ^{13}C NMR DEPT 135 spectrum of compound **12b** in DMSO-d_6.

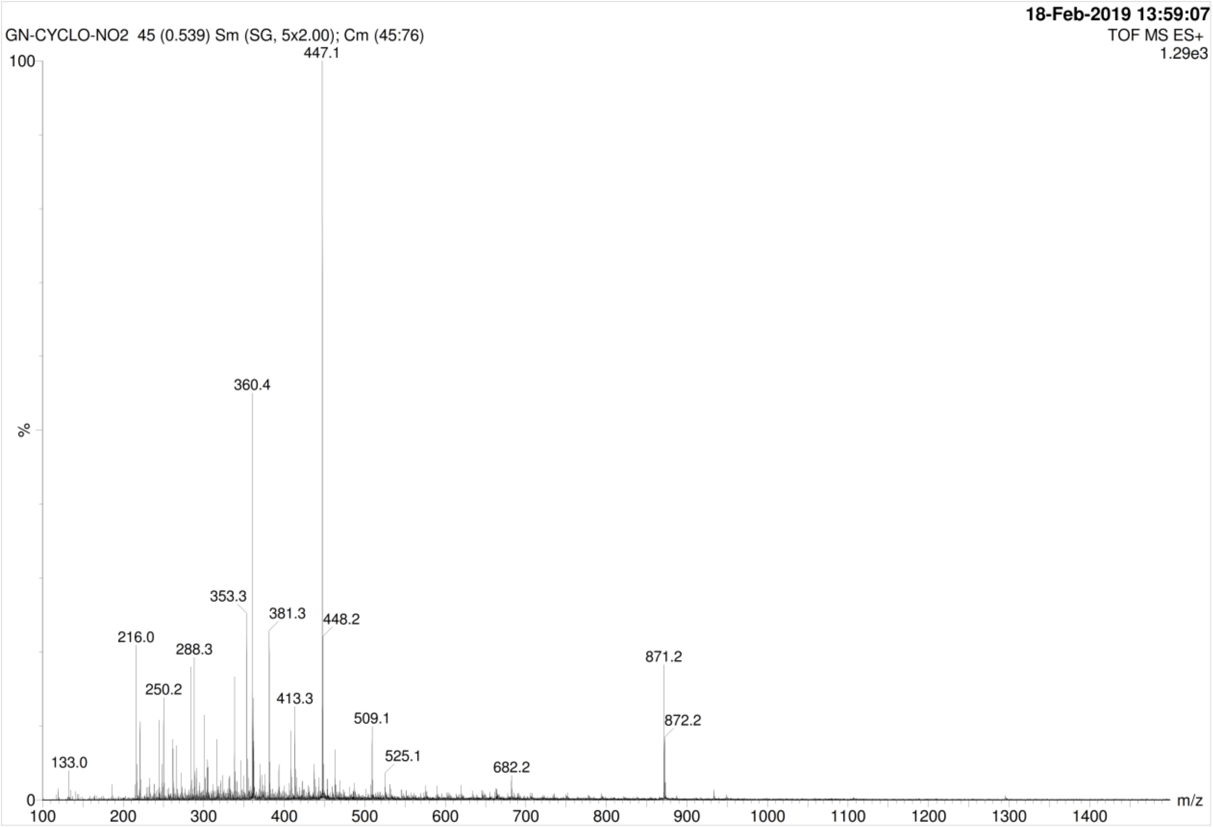

Figure S105. MS-ESI(+) spectrum of compound **12b**.

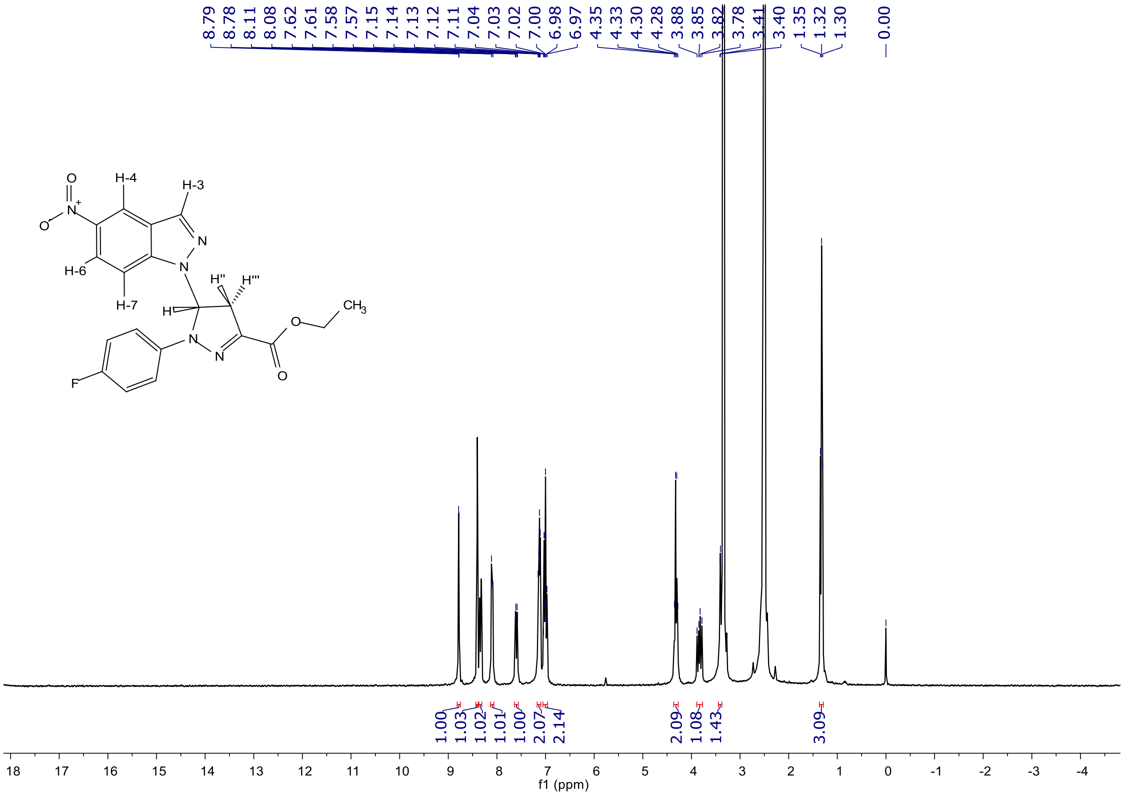

Figure S106. ^{1}H NMR spectrum of compound **12c** in DMSO-d_6.

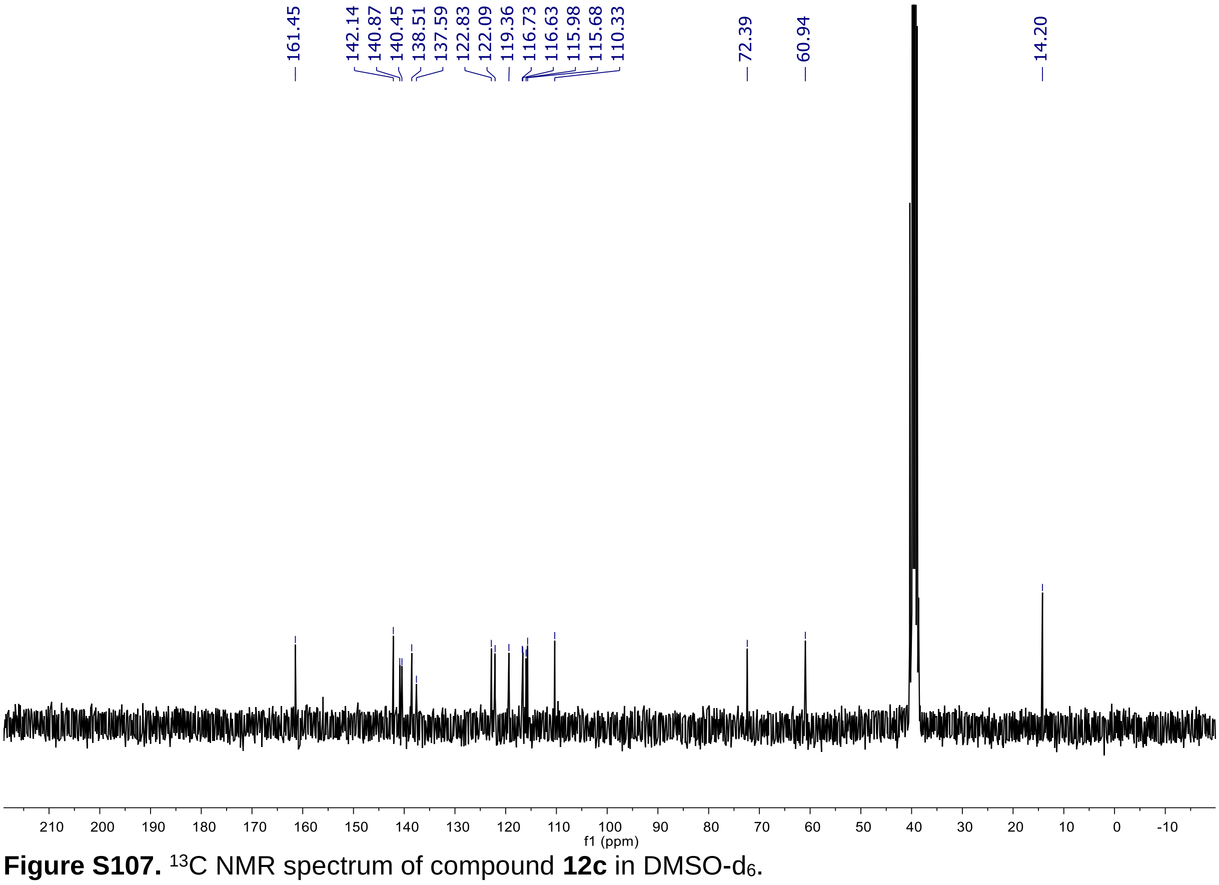

Figure S107. ^{13}C NMR spectrum of compound **12c** in DMSO-d_6.

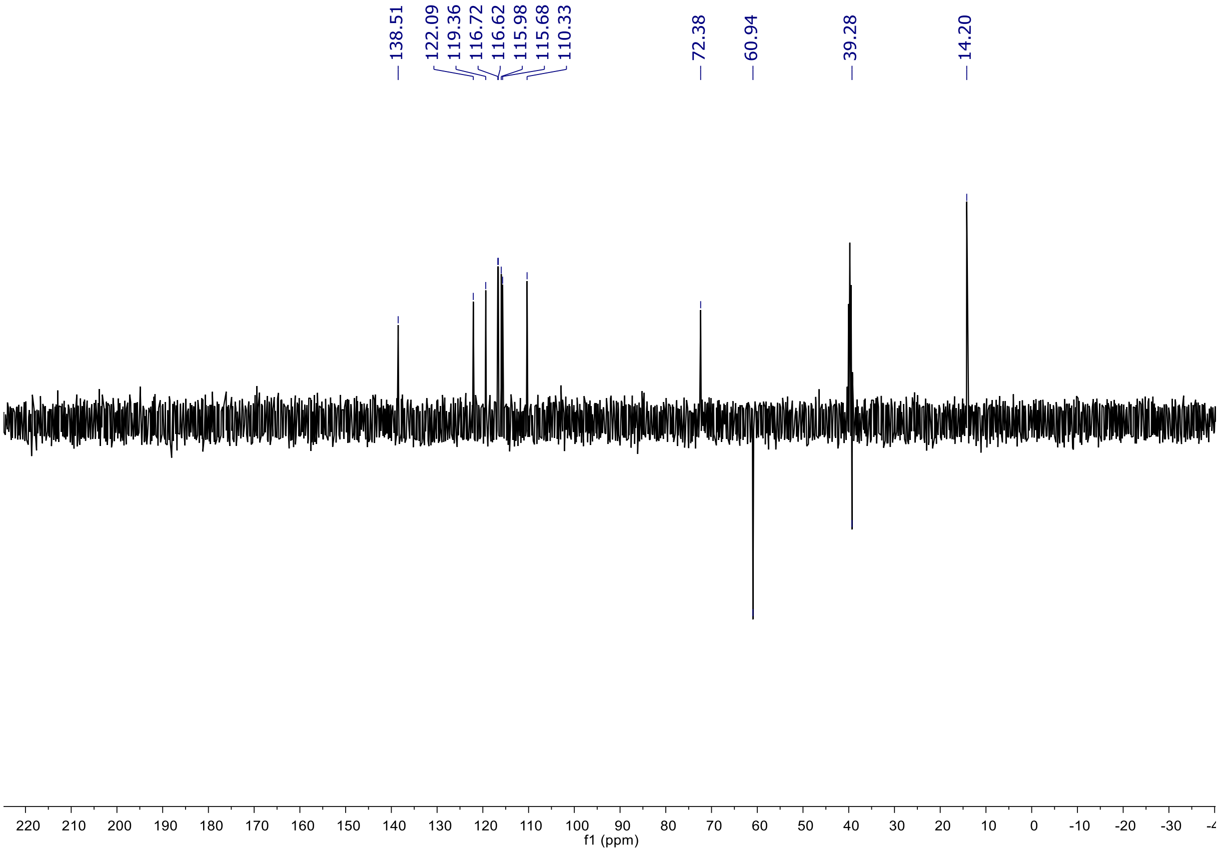

Figure S108. ^{13}C NMR DEPT 135 spectrum of compound **12c** in DMSO-d_6.

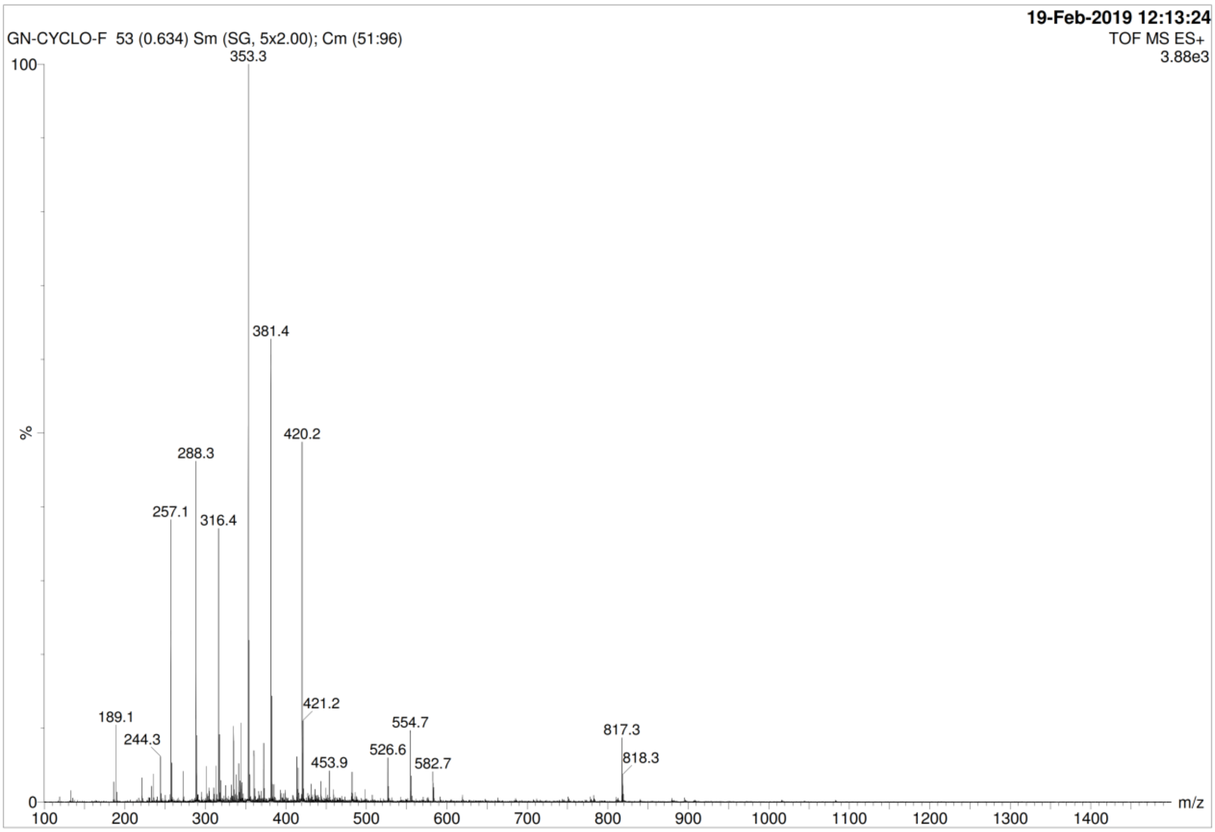

Figure S109. MS-ESI(+) spectrum of compound **12c**.

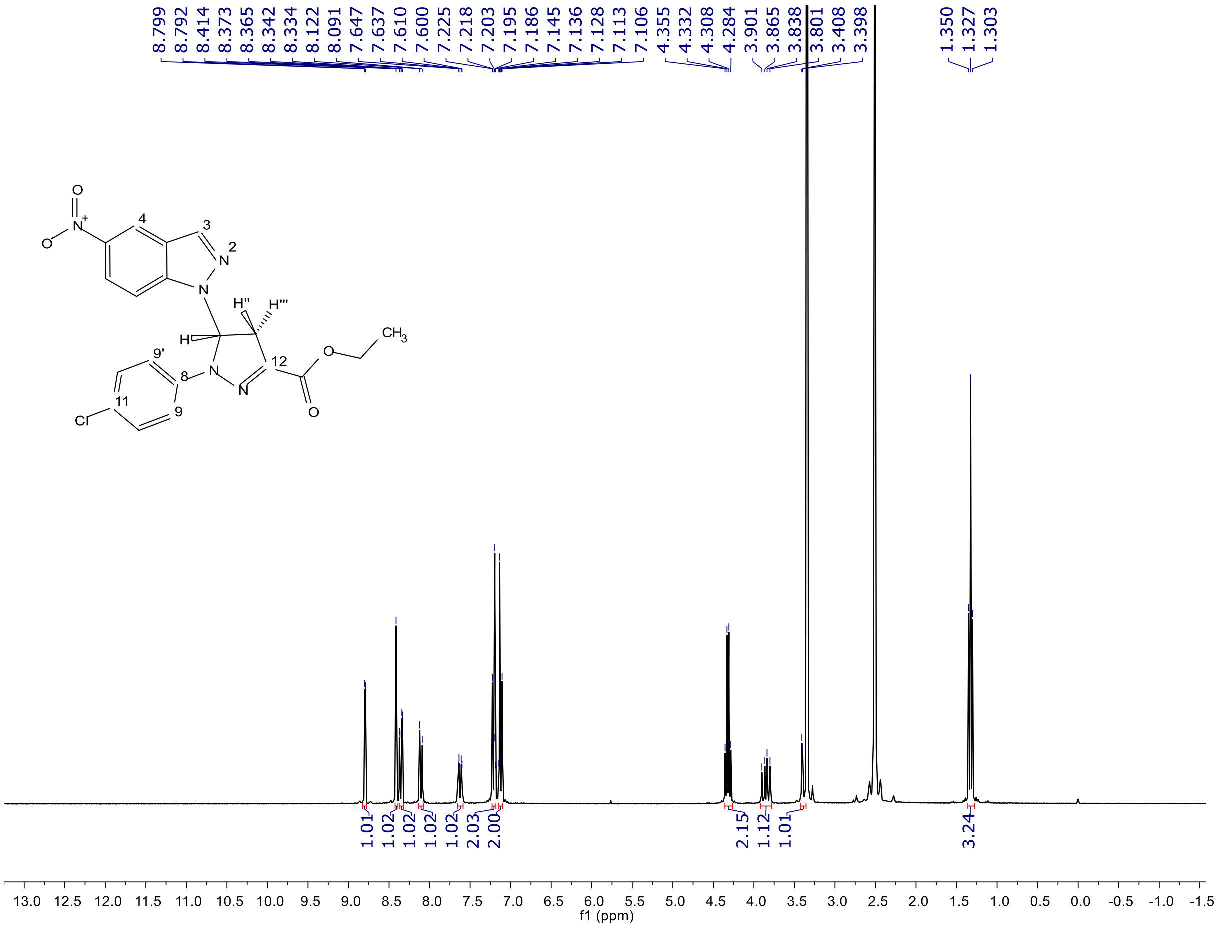

Figure S110. ^{1}H NMR spectrum of compound **12d** in DMSO-d_6.

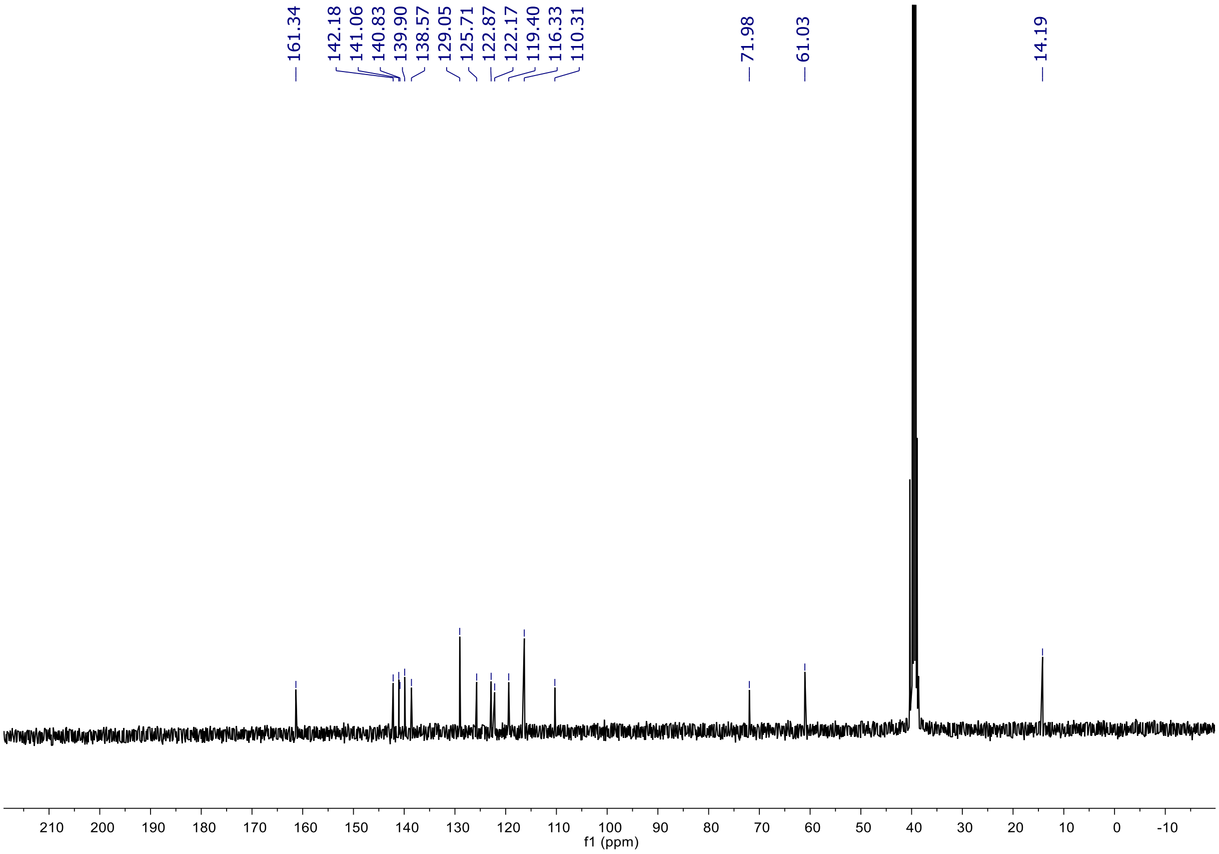

Figure S111. ^{13}C NMR spectrum of compound **12d** in DMSO-d_6.

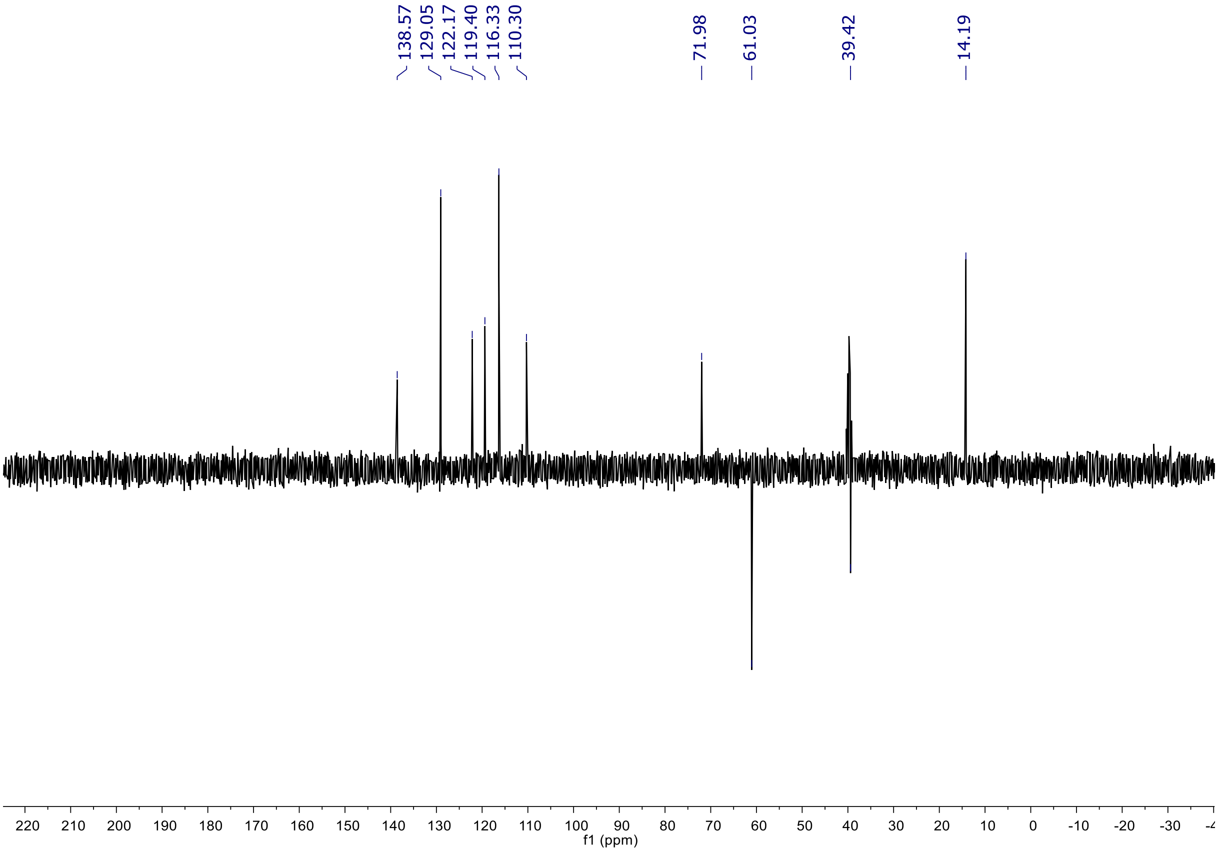

Figure S112. ^{13}C NMR DEPT 135 spectrum of compound **12d** in DMSO-d_6.

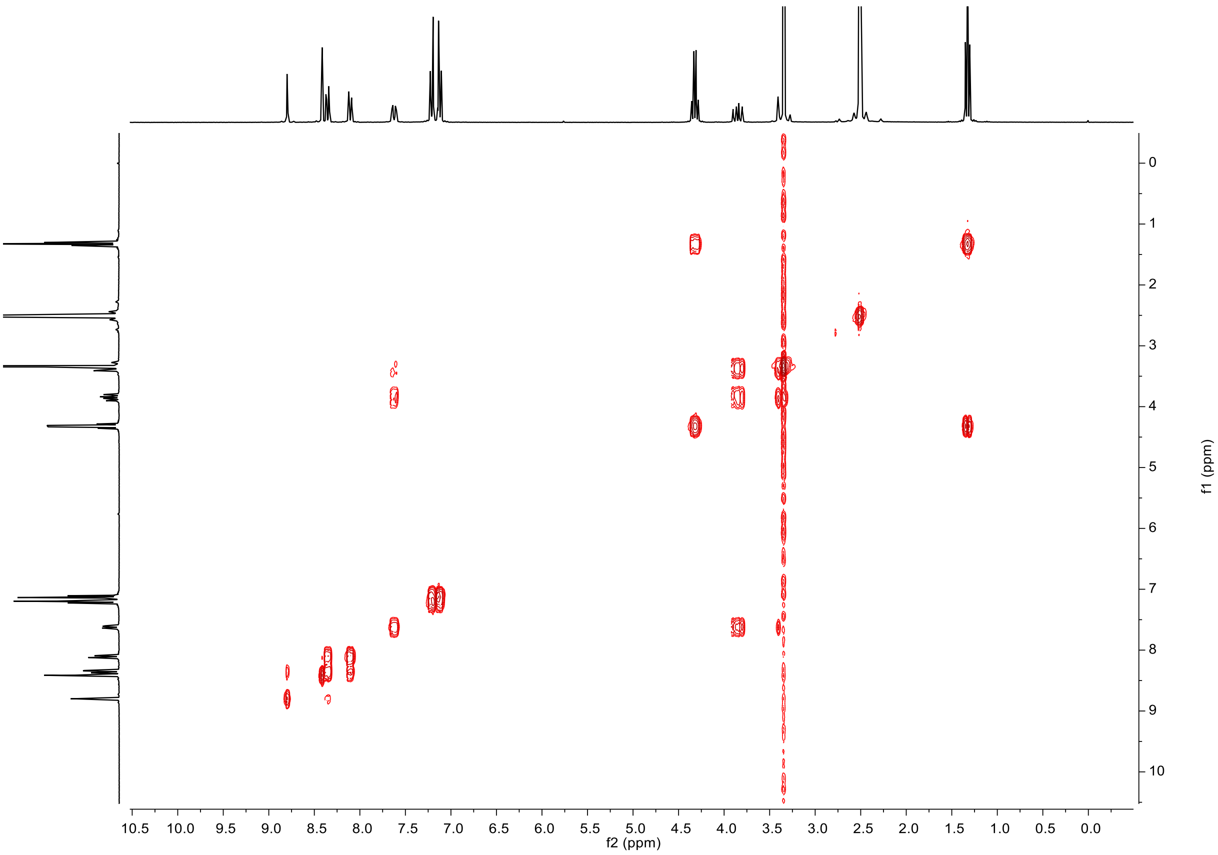

Figure S113. COSY ($^1H/^1H$) spectrum of compound **12d** in DMSO-d_6.

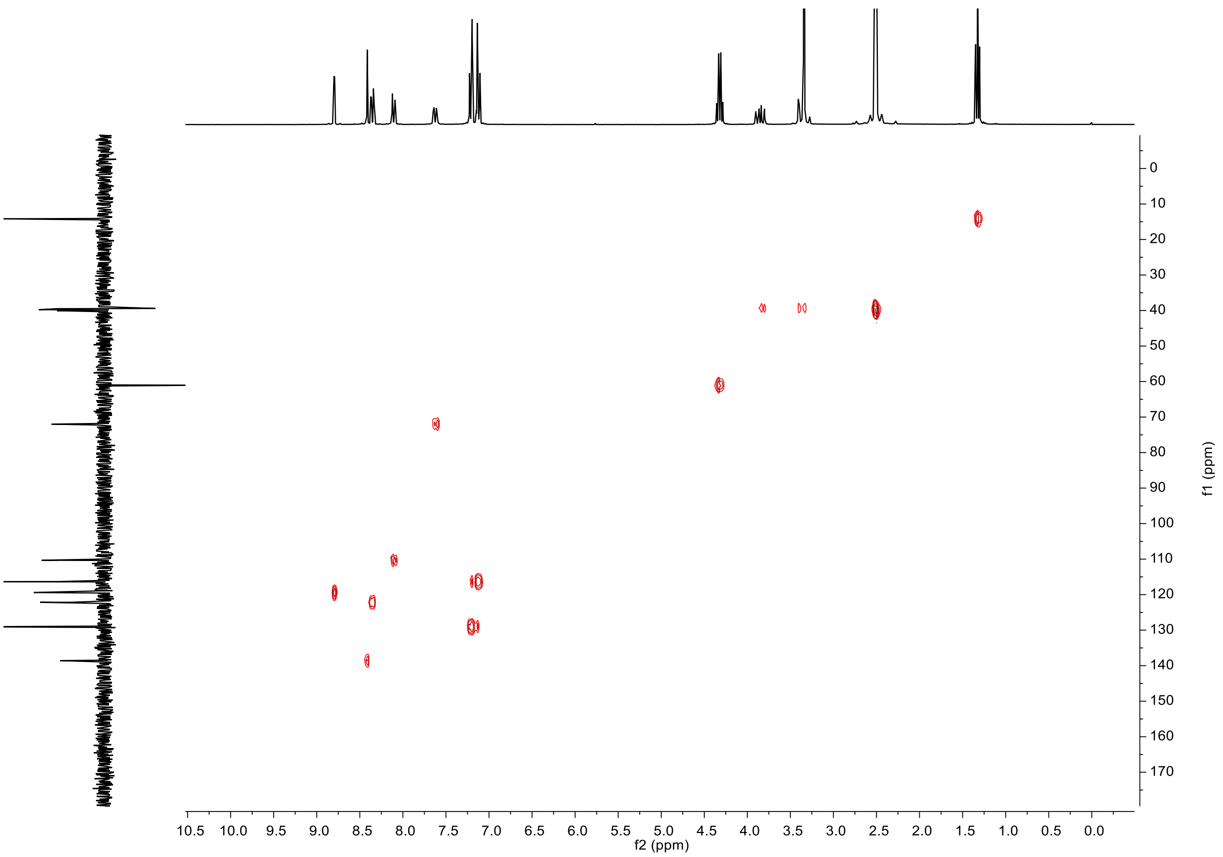

Figure S114. HSQC ($^{1}H/^{13}C$) spectrum of compound **12d** in DMSO-d_6.

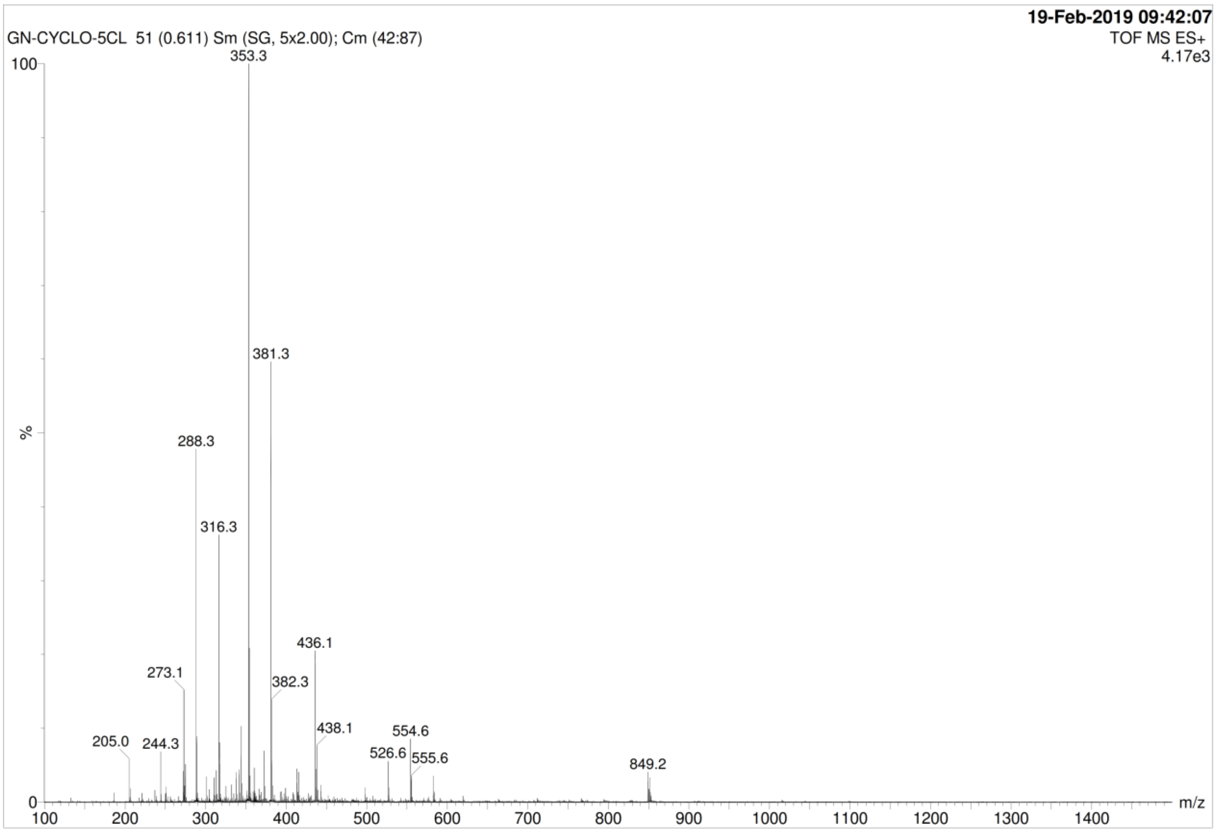

Figure S115. MS-ESI(+) spectrum of compound **12d**.

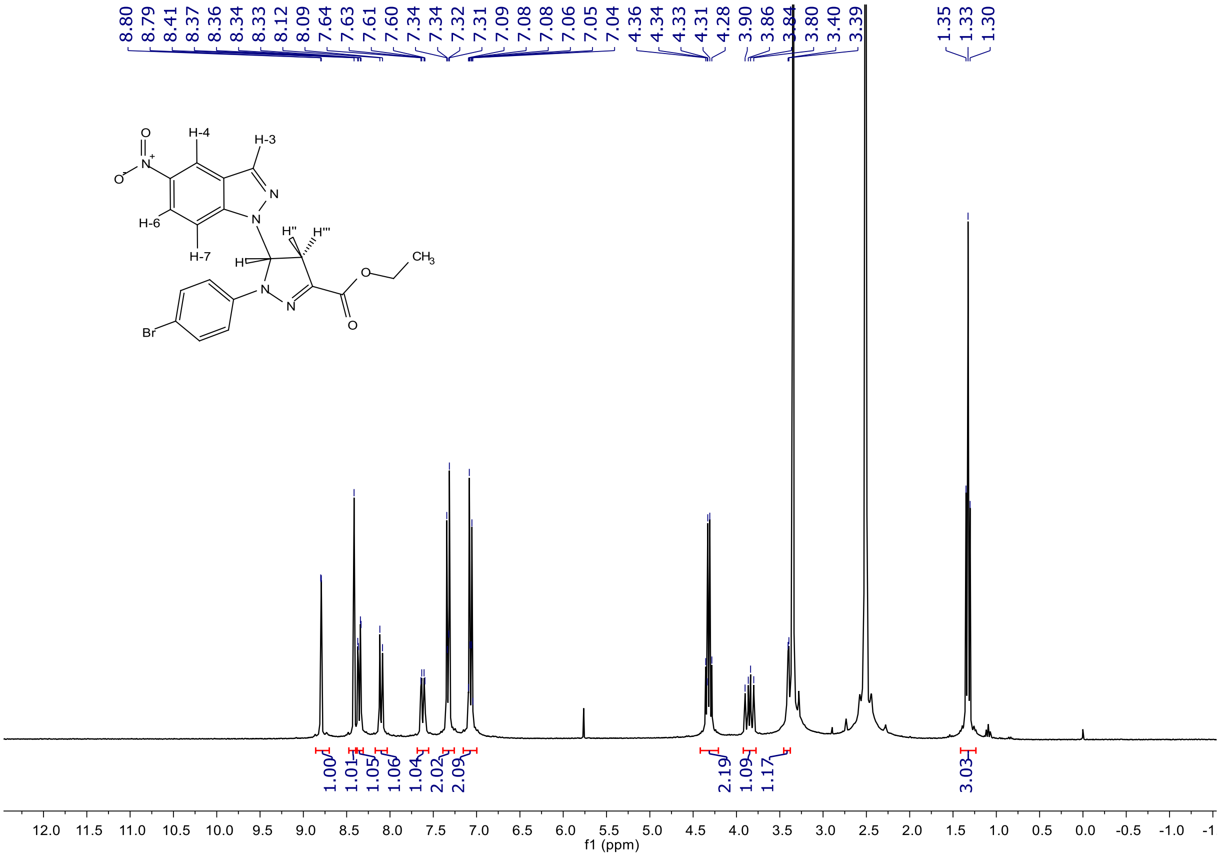

Figure S116. ^{1}H NMR spectrum of compound **12e** in DMSO-d$_6$.

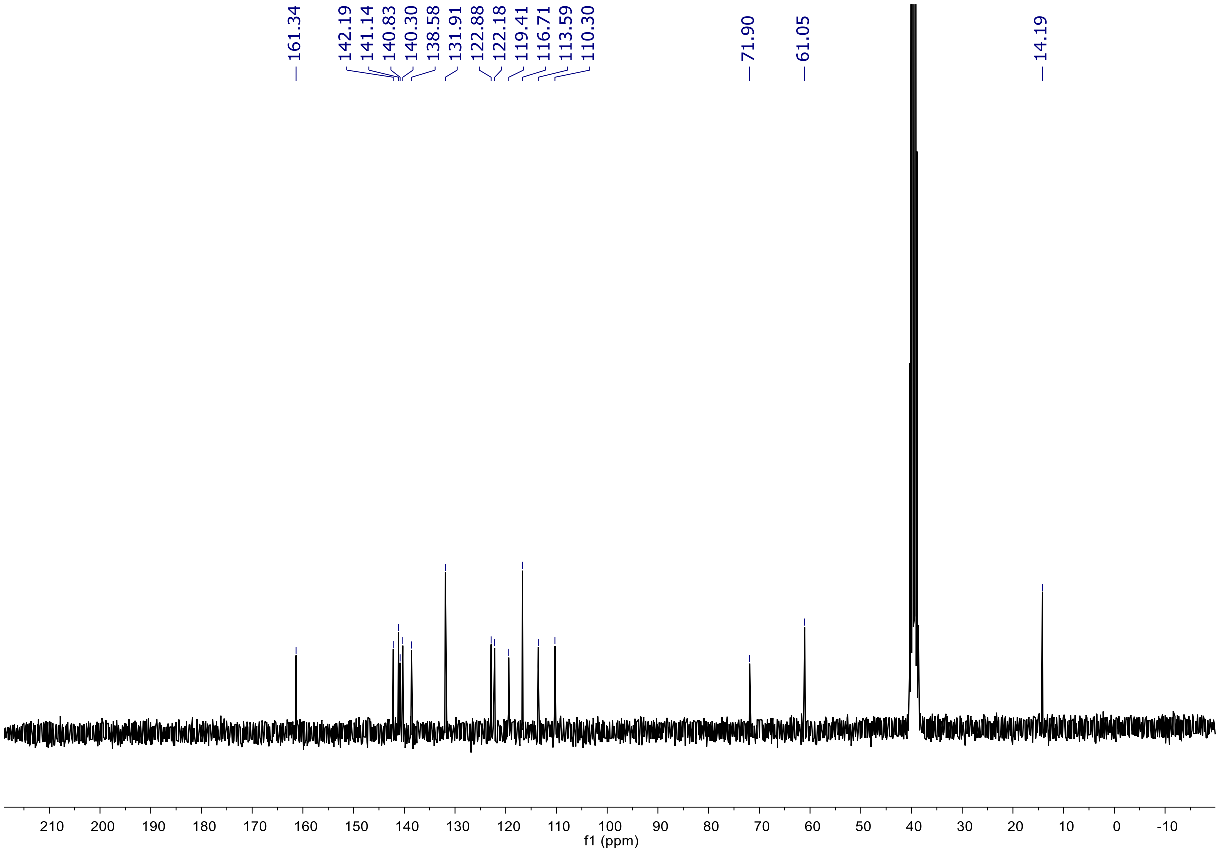

Figure S117. ^{13}C NMR spectrum of compound **12e** in DMSO-d$_6$.

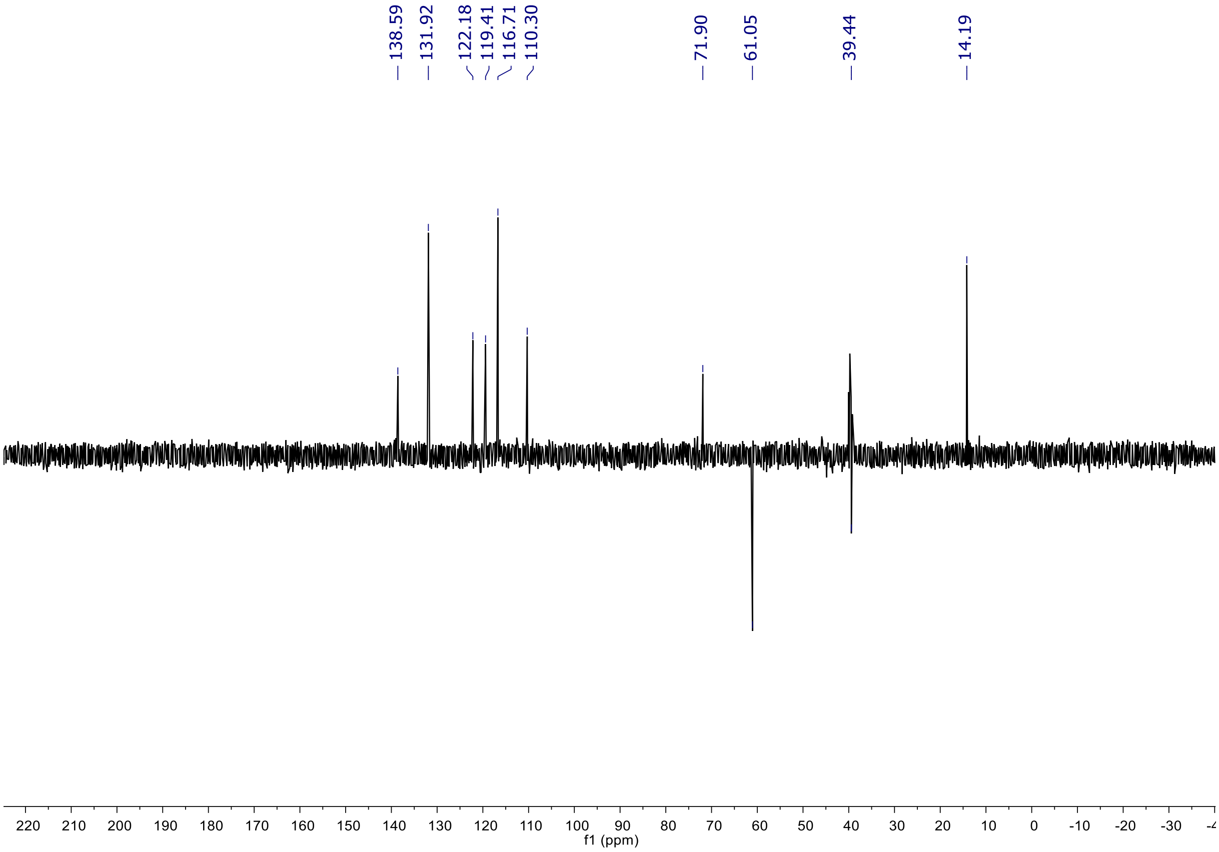

Figure S118. ^{13}C NMR DEPT 135 spectrum of compound **12e** in DMSO-d_6.

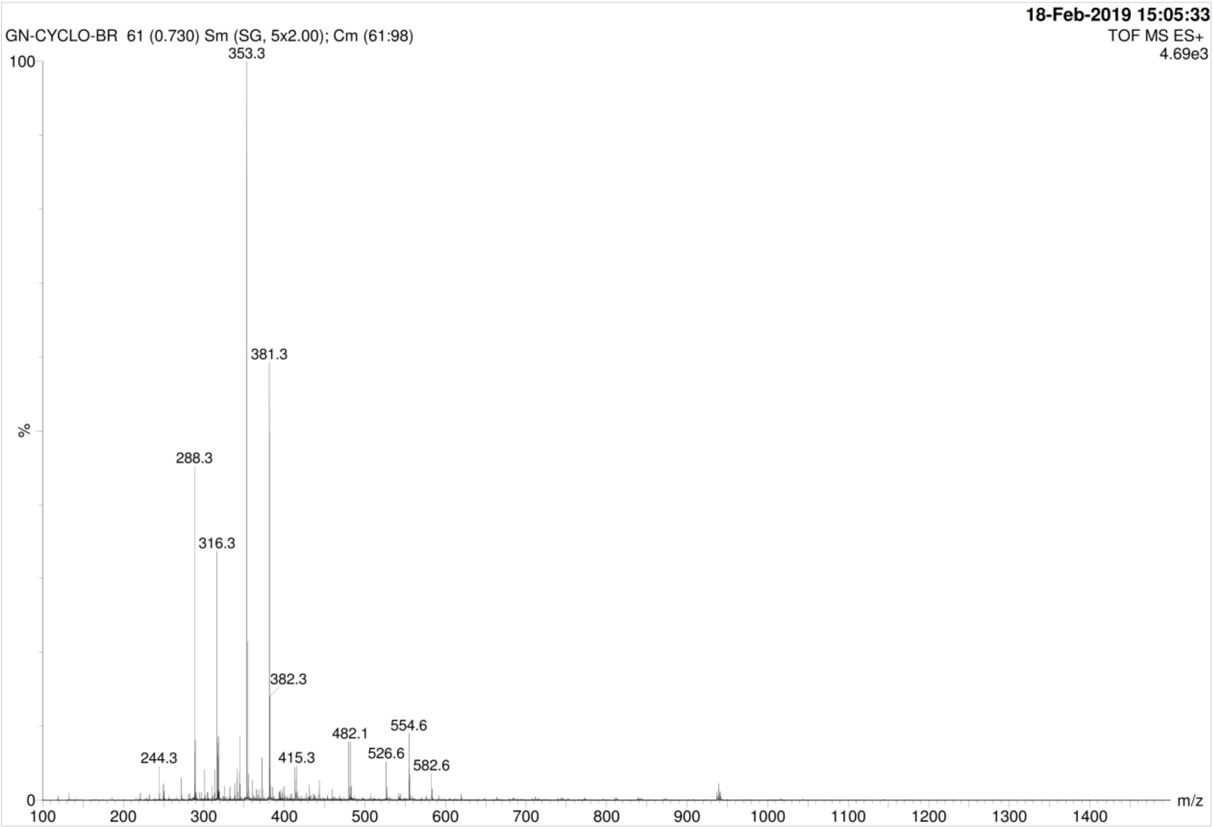

Figure S119. MS-ESI(+) spectrum of compound **12e**.

IV - NMR and mass spectrum of compounds 13

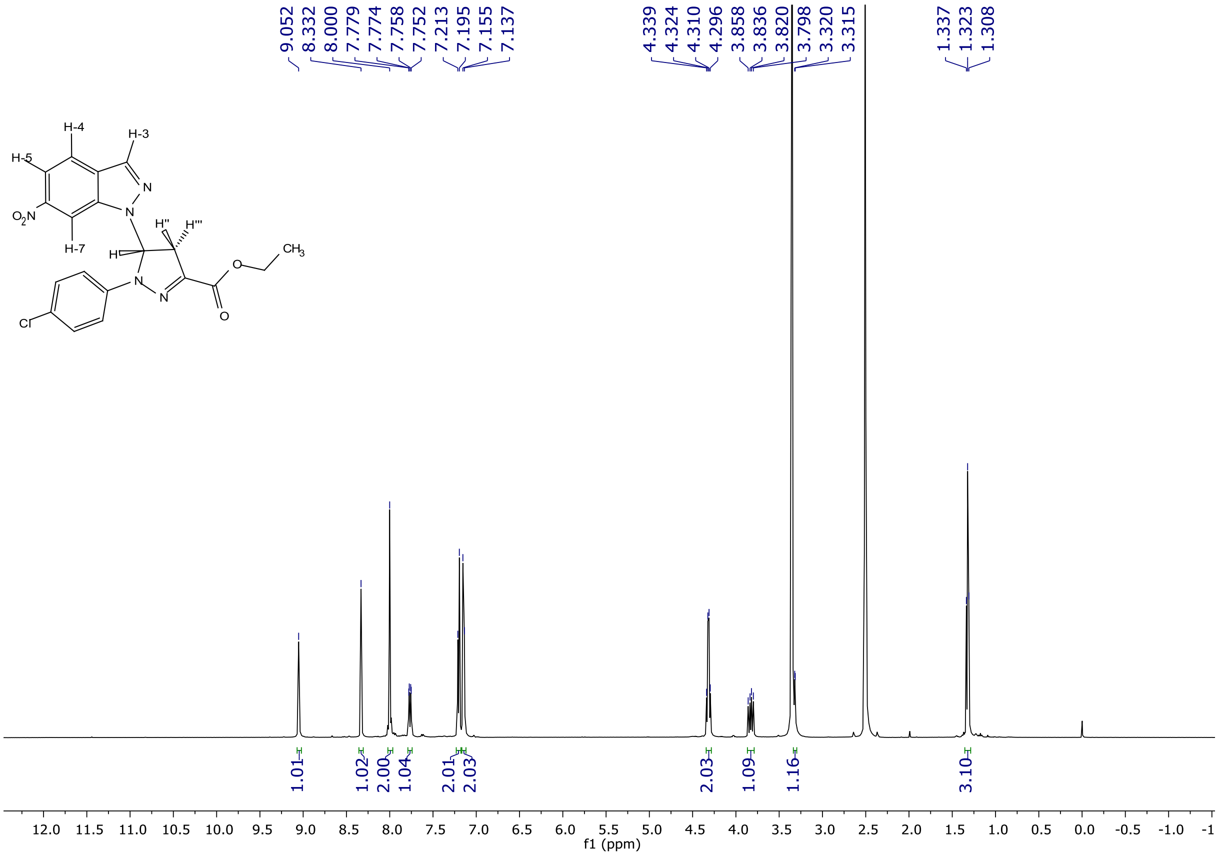

Figure S120. ^{1}H NMR spectrum of compound **13** in DMSO-d_6.

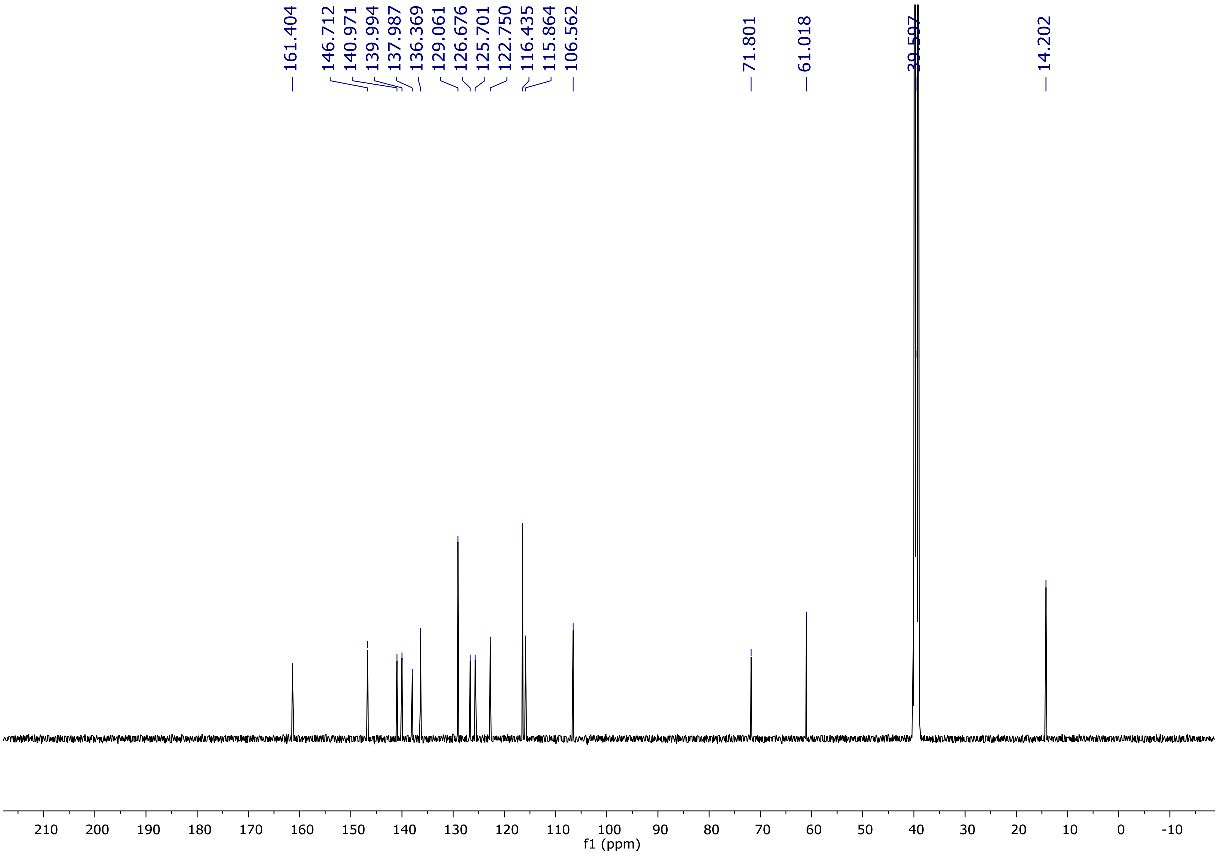

Figure S121. ^{13}C NMR spectrum of compound **13** in DMSO-d_6.

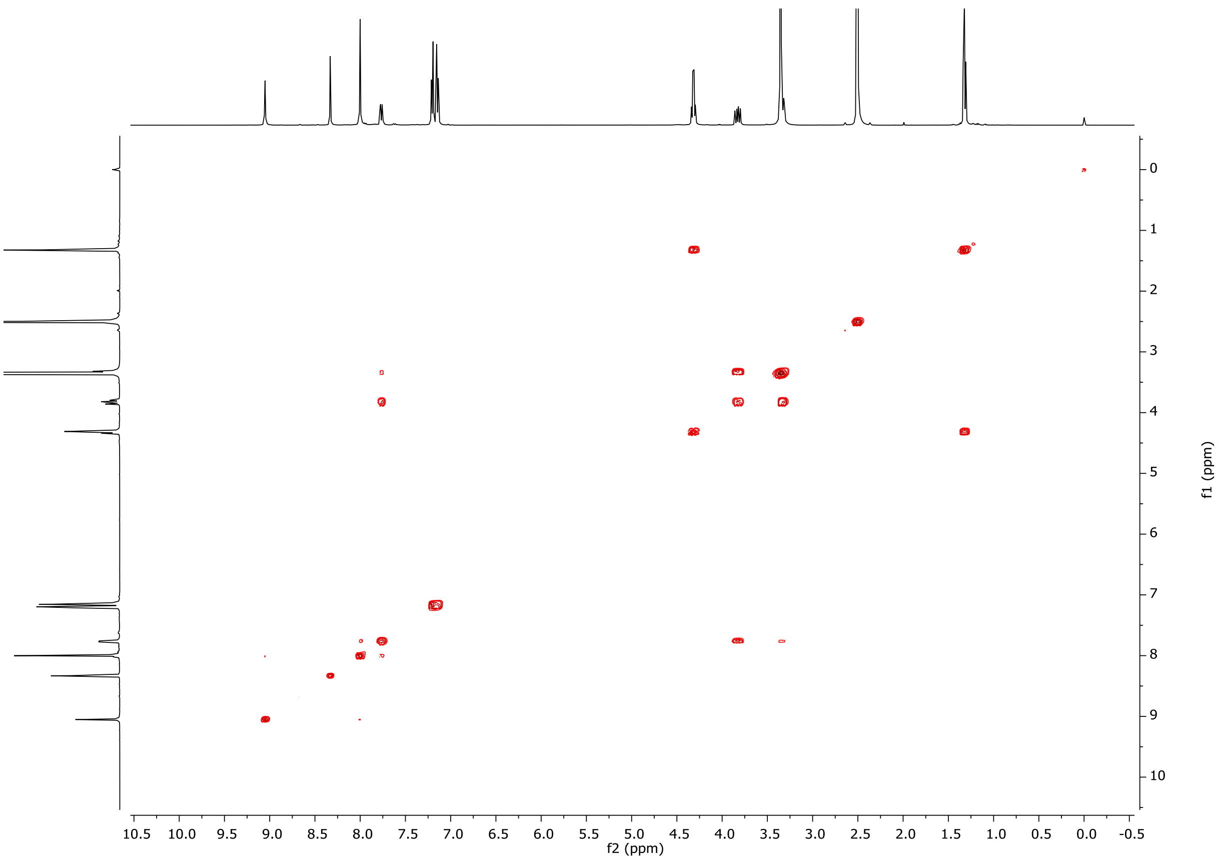

Figure S122. COSY ($^1H/^1H$) spectrum of compound **13** in DMSO-d_6.

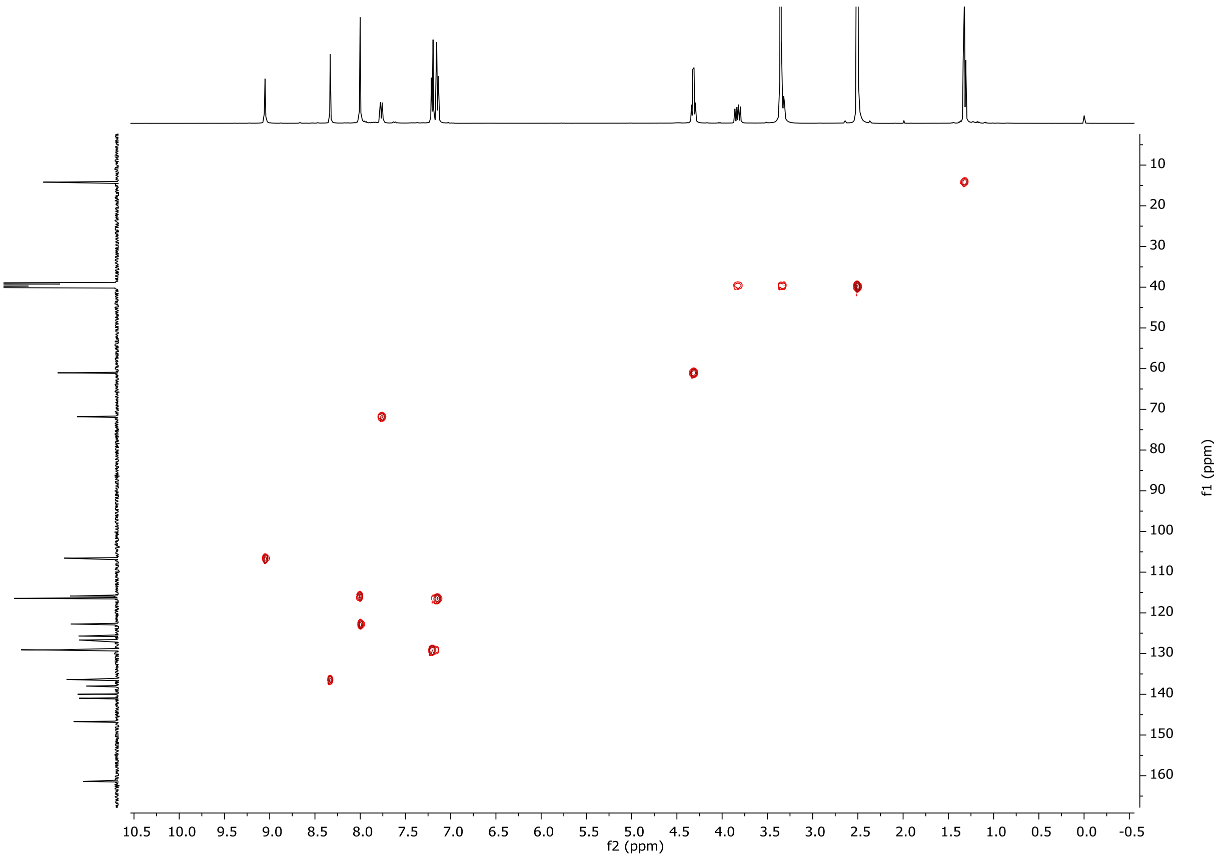

Figure S123. HSQC ($^1H/^{13}C$) spectrum of compound **13** in DMSO-d_6.

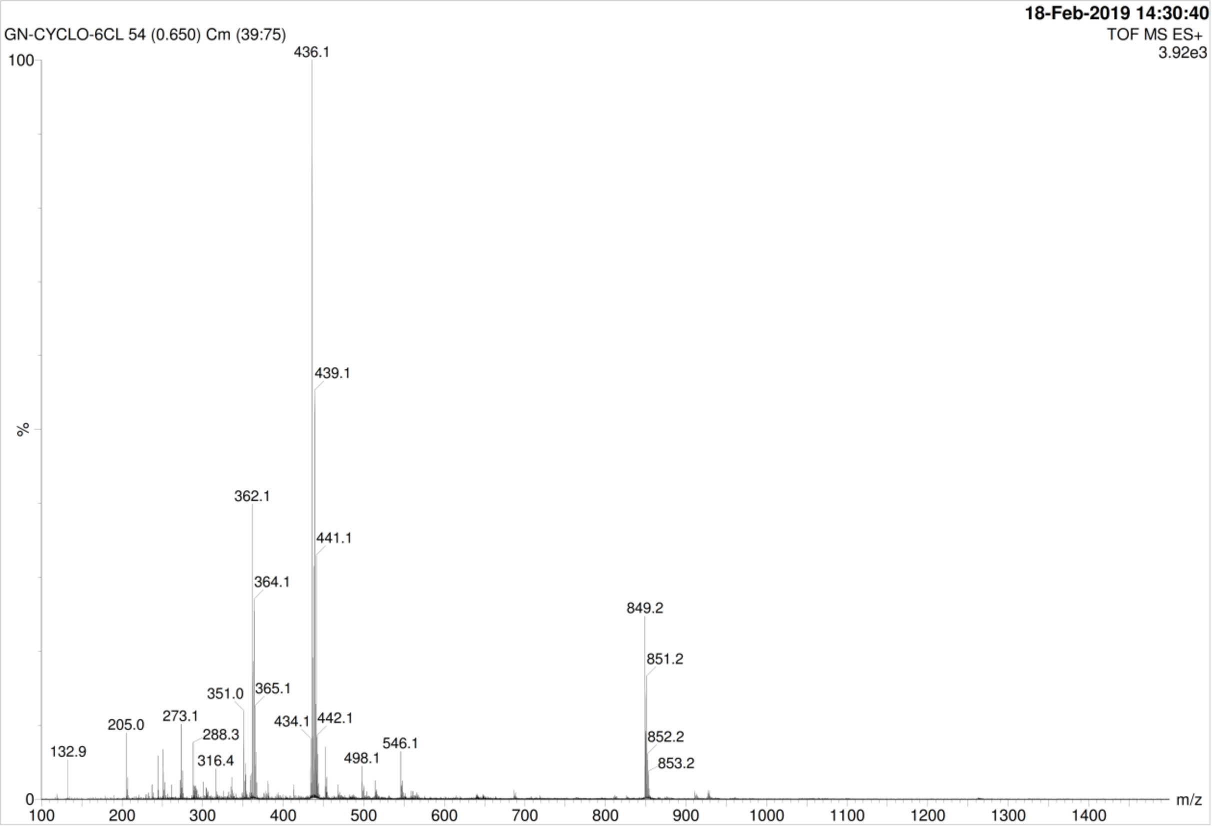

Figure S124. MS-ESI(+) spectrum of compound **13**.
